# Asymmetric [3 + 2] cycloaddition of donor–acceptor aziridines with aldehydes *via* carbon–carbon bond cleavage†
†Electronic supplementary information (ESI) available. CCDC 1057118. For ESI and crystallographic data in CIF or other electronic format see DOI: 10.1039/c5sc04151a


**DOI:** 10.1039/c5sc04151a

**Published:** 2016-02-23

**Authors:** Yuting Liao, Xiaohua Liu, Yu Zhang, Yali Xu, Yong Xia, Lili Lin, Xiaoming Feng

**Affiliations:** a Key Laboratory of Green Chemistry & Technology , Ministry of Education , College of Chemistry , Sichuan University , Chengdu 610064 , China . Email: liuxh@scu.edu.cn ; Email: xmfeng@scu.edu.cn ; Fax: +86 28 85418249 ; Tel: +86 28 85418249; b Collaborative Innovation Center of Chemical Science and Engineering (Tianjin) , China

## Abstract

An enantioselective [3 + 2] annulation of donor–acceptor aziridines with aldehydes has been realized using a Nd(OTf)_3_/*N*,*N*′-dioxide/LiNTf_2_ catalyst system, providing various chiral *cis*-1,3-oxazolidines in moderate to good yields with high levels of stereocontrol. A relay catalytic process is proposed where LiNTf_2_ promotes the formation of azomethine ylide intermediates, and a chiral Nd(iii)–*N*,*N*′-dioxide complex accelerates the asymmetric cycloaddition.

## Abstract 2

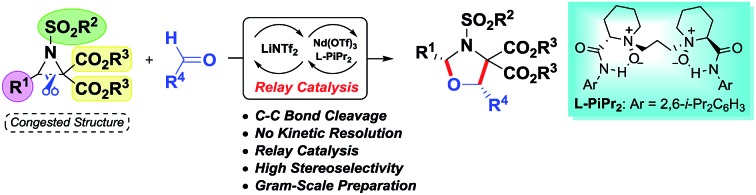
An enantioselective [3 + 2] annulation of donor–acceptor aziridines with aldehydes utilizing a relay catalyst system of Nd(OTf)_3_/*N*,*N*′-dioxide/LiNTf_2_ has been realized.

## Introduction

**Scheme 1:**
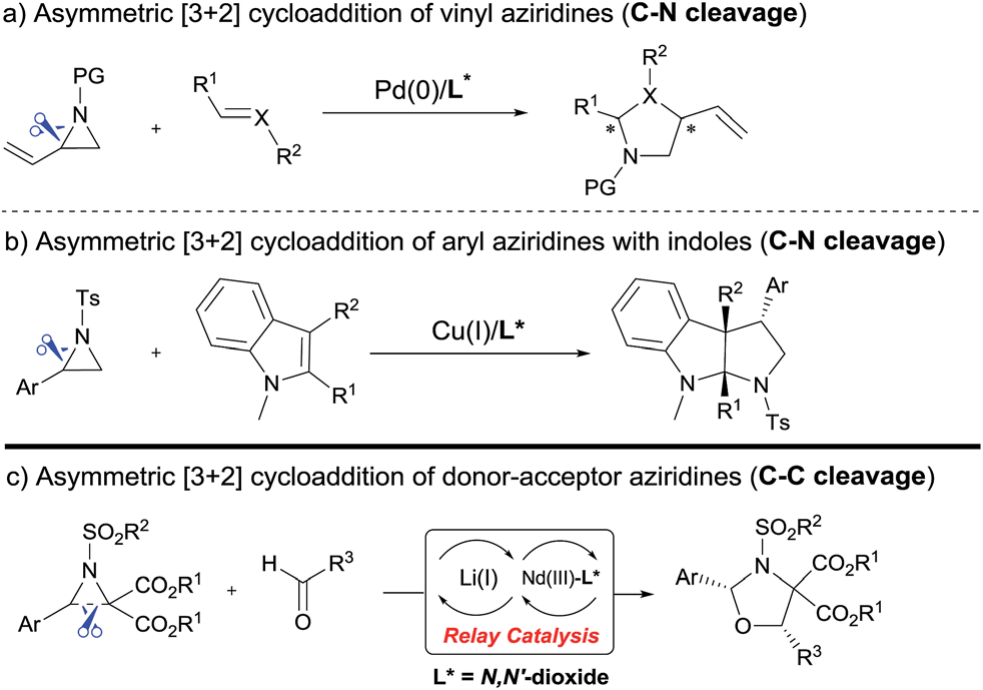
Asymmetric [3 + 2] cycloadditions of racemic aziridines.

The cycloaddition of aziridines is an attractive method for obtaining various nitrogen-containing heterocycles.^1^ Among them, highly enantioselective [3 + 2] cycloadditions *via* C–N bond cleavage of racemic aziridines have been realized. For example, the asymmetric cycloadditions of racemic vinyl aziridines with isocyanates and α,β-unsaturated ketones have been reported by Trost and Hou, respectively (Scheme 1a).^2^ Very recently, Wang realized the chiral copper(i) complex catalyzed [3 + 2] annulation of racemic 2-aryl-*N*-tosylaziridines with indoles (Scheme 1b).^3^ As a type of donor–acceptor (DA) variation, 2,3-diester aziridines favor C–C bond heterolytic cleavage, and the formed transient azomethine ylide intermediates can undergo cycloadditions with various dipolarophiles (Scheme 1c). These transformations can be promoted by Lewis acids under mild conditions in comparison to photochemically or thermally induced conditions.^4^ A pioneering example is the ZnCl_2_ catalyzed cycloaddition between *N*-aryl-2,3-diester aziridines and electron-rich alkenes developed by Johnson.5*a* The Zhang group and others have further explored Lewis acid accelerated cycloadditions of *N*-tosylaziridinedicarboxylates with aldehydes,5b,c imines,5c,d electron-rich alkenes,5*e* alkynes,5*f* 2,3-disubstituted indoles,5g,h donor–acceptor cyclopropanes,5*i* isocyanides5*j* and heterocumulenes.5*k*

Despite these important racemic examples, catalytic asymmetric [3 + 2] cycloadditions of DA aziridines are rare, with the results yielding no more than 70% ee.5b,d–f Compared with their cousins, DA cyclopropanes^6^ and DA oxiranes,^7^ enantioselective [3 + 2] cycloadditions of aziridines are more difficult with considerable challenges: (1) the azomethine ylide *via* C–C bond cleavage is transient, being further converted to 2-amino malonate and aldehyde in the presence of unavoidable trace amounts of water that prevent cycloadditions.5a,e (2) DA aziridine has a relatively congested structure tethering four substituents on a three-membered ring, which hampers its interaction with a chiral catalyst. Additionally, the competitive coordination of aldehydes to the chiral catalyst is also disadvantageous to the yield and stereocontrol. Therefore, asymmetric cycloadditions of DA aziridines require a powerful catalytic system enabling both C–C bond cleavage and enantiocontrol. Here we report a relay catalyst system of Nd(OTf)_3_/*N*,*N*′-dioxide/LiNTf_2_ for the asymmetric cycloaddition of DA aziridines with aldehydes (Scheme 1c).^8^ The relay approach is to use an achiral metal salt to accelerate the formation of the azomethine ylide intermediate, which is then transformed into a chiral catalytic environment to undergo asymmetric cycloaddition. Chiral *cis*-1,3-oxazolidines, which have emerged as crucial structural units in chiral catalysts and biologically active compounds,^9^ could be formed in moderate to high yield with good enantioselectivity.

## Results and discussion

We selected the model [3 + 2] annulation of DA aziridine **1a** with benzaldehyde **2a** as our starting point (see Table 1). In the presence of 10 mol% *N*,*N*′-dioxide **L-PiPr_2_** and 4 Å MS, metal salts that have previously been established as efficient Lewis acids for catalytic racemic transformations were tested (entries 1–4). Disappointingly, low yields and enantioselectivities were obtained with Sc(OTf)_3_, Ni(ClO_4_)_2_, Zn(OTf)_2_ and La(OTf)_3_ due to the decomposition of aziridine **1a**. La(OTf)_3_/**L-PiPr_2_** provided the *cis*-1,3-oxazolidines **3aa** in 14% yield with 36% ee in toluene at 35 °C. We surmised whether extra Lewis acid could assist in furnishing the desired transformation.^7^ Encouragingly, the addition of LiNTf_2_ (10 mol%) was indeed beneficial for both the reactivity and enantioselectivity (entry 5). After examining a series of lanthanide metal salts (see the ESI†), Nd(OTf)_3_ was selected as the best one, resulting in 30% yield and 71% ee (entry 6). Next, the systematic modification of *N*,*N*′-dioxides through alteration of their amino acid backbones and amide moieties, as well as the length of the linkage, showed **L-PiPr_2_** was the optimal ligand (see the ESI†). Empirically changing the solvent to CHCl_3_ instead of toluene improved the enantioselectivity to 85% ee (entry 7). Optimization of other reaction conditions by adjusting the ratio of Nd(OTf)_3_ to **L-PiPr_2_** (2 : 1) and increasing the amount of LiNTf_2_, 4 Å MS and aldehyde to speed up the reaction provided the product **3aa** in a good yield of 65% with 87% ee (entry 8). It should be considered that the azomethine ylide intermediate derived from DA aziridine was very unstable, thereby a 2-fold excess of benzaldehyde **2a** was necessary to achieve a satisfying outcome. Furthermore, chromatography purification using basic Al_2_O_3_ as the stationary phase instead of silica gel, led to an encouraging outcome with 68% yield and 91% ee (entry 9). Basic Al_2_O_3_ might prevent the partial racemization of chiral *cis*-1,3-oxazolidines that occurs in silica gel. Notably, **L-PiPr_2_** at a loading of 2.5 mol% was able to catalyse the reaction efficiently without any changes to the outcome (entry 10).

**Table 1:** Optimization of the reaction conditions^*a*^

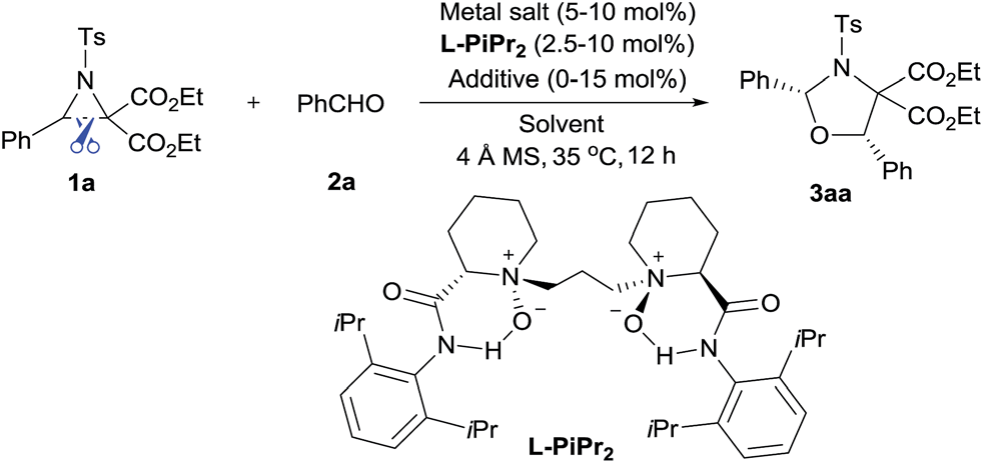					
Entry	Metal salt	Additive	Solvent	Yield^*b*^ (%)	ee^*c*^ (%)
1	Sc(OTf)_3_	—	Toluene	45	0
2	Ni(ClO_4_)_2_·6H_2_O	—	Toluene	20	–22
3	Zn(OTf)_2_	—	Toluene	Trace	—
4	La(OTf)_3_	—	Toluene	14	36
5^*d*^	La(OTf)_3_	LiNTf_2_	Toluene	24	58
6^*d*^	Nd(OTf)_3_	LiNTf_2_	Toluene	30	71
7^*d*^	Nd(OTf)_3_	LiNTf_2_	CHCl_3_	35	85
8^*e*^	Nd(OTf)_3_	LiNTf_2_	CHCl_3_	65	87
9^*e*^ ^,^^*f*^	Nd(OTf)_3_	LiNTf_2_	CHCl_3_	68	91
10^*e*^ ^–^^*g*^	Nd(OTf)_3_	LiNTf_2_	CHCl_3_	68	91

^*a*^Unless otherwise noted, the reactions were performed with metal salt/L* (10 mol%, 1 : 1), 4 Å MS (20 mg), aziridine **1a** (0.1 mmol) and PhCHO **2a** (0.15 mmol) in solvent (1.0 mL) under nitrogen at 35 °C for 12 h.

^*b*^Isolated yield by silica gel chromatography.

^*c*^The ratio of *cis*/*trans* was >19 : 1 determined by ^1^H NMR spectroscopy and ee values were determined by chiral HPLC analysis.

^*d*^LiNTf_2_ (10 mol%) was used.

^*e*^Nd(OTf)_3_/**L-PiPr_2_** (5 mol%, 2 : 1), LiNTf_2_ (15 mol%), 4 Å MS (100 mg), aziridine **1a** (0.1 mmol) and PhCHO **2a** (0.2 mmol) in CHCl_3_ (0.75 mL).

^*f*^Isolation by flash chromatography using basic Al_2_O_3_.

^*g*^Nd(OTf)_3_/**L-PiPr_2_** (2.5 mol%, 2 : 1), LiNTf_2_ (15 mol%).

Examples of asymmetric [3 + 2] annulations of DA aziridines **1** with benzaldehyde **2a** promoted by the Nd(OTf)_3_/LiNTf_2_/**L-PiPr_2_** catalyst system are summarized in Table 2. For the ester moieties, the methyl group had less influence on the outcome, while the isopropyl group was detrimental to both the reactivity and enantioselectivity (entries 1–3). Other benzenesulfonyl motifs were well tolerated except for those with 2-methyl and 2-nitro substituents, which slowed down the reaction and diminished the yield and ee value, perhaps as a result of the steric hindrance at the *ortho*-position (entries 4–8). It was noteworthy that the methanesulfonyl substituent could make the result rise up to 77% yield and 95% ee (entry 9). Moreover, the 2-trimethylsilylethanesulfonyl group, which proved to be readily cleaved under mild conditions,^10^ was also a tolerable substituent for the catalyst system (entry 10). Subsequently, a variety of aryl aziridines with electron-withdrawing substituents provided the cycloadducts in 66–98% yields and 87–94% ee (entries 11–17). The position of the substituent had some influence on the enantioselectivity, and the *ortho*-substituted analogue required an elongated reaction time and gave a reduced ee value (entries 11–13). The aryl substituent could be replaced by biphenyl or naphthalene-2-yl groups and the desired products were obtained in high yields and enantioselectivities, albeit with the need for a 3-fold excess of benzaldehyde **2a** (entries 18–19). This large excess of benzaldehyde was beneficial for accomplishing the cycloadditions with more reactive azomethine ylide intermediates of DA aziridines **1r** and **1s**. Also, it could avoid the background reactions of DA aziridines **1r** and **1s** with the aldehydes from the decomposition. The absolute configuration of the product **3sa** was determined to be (2*R*, 5*S*) by X-ray crystal analysis.^11^ The alkyl substituted aziridine was inert and none of the corresponding 1,3-oxazolidine was generated (entry 20). This result should be caused by the poor stability of its azomethine ylide intermediate compared to the aryl substituted ones. Overall, only *cis*-1,3-oxazolidines were detected.

**Table 2:** Substrate scope of donor–acceptor aziridines^*a*^

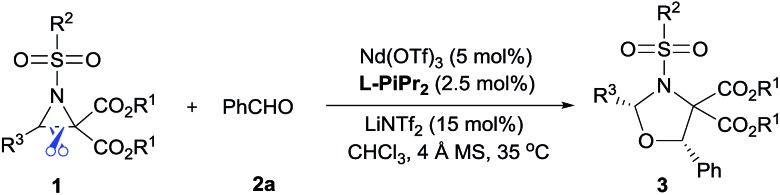					
Entry		**3**	*t* (h)	Yield^*b*^ (%)	ee^*c*^ (%)
1	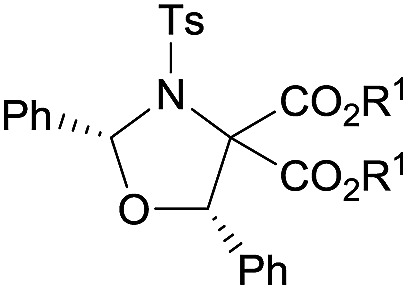	Et (**3aa**)	12	68	91
2^*d*^		Me (**3ba**)	12	62	90
3^*d*^		iPr (**3ca**)	14	40	72
4^*d*^	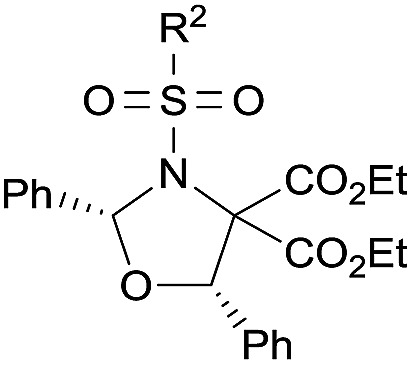	4-ClC_6_H_4_ (**3da**)	12	74	89
5		C_6_H_5_ (**3ea**)	12	70	90
6		4-MeOC_6_H_4_ (**3fa**)	12	60	90
7		2-MeC_6_H_4_ (**3ga**)	38	54	76
8		2-O_2_NC_6_H_4_ (**3ha**)	92	6^*e*^	62
9^*d*^		Me (**3ia**)	12	77	95
10^*d*^		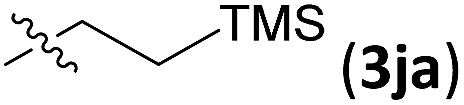	12	66	93
11^*d*^	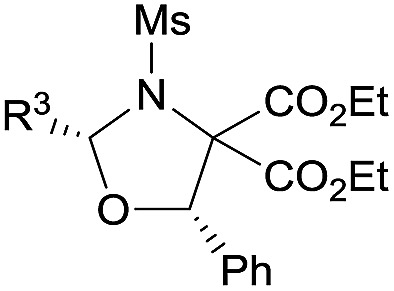	4-ClC_6_H_4_ (**3ka**)	24	80	91
12^*d*^		3-ClC_6_H_4_ (**3la**)	24	78	92
13^*d*^		2-ClC_6_H_4_ (**3ma**)	40	71	88
14^*d*^		4-BrC_6_H_4_ (**3na**)	24	70	93
15^*d*^		4-FC_6_H_4_ (**3oa**)	24	66	94
16^*d*^ ^,^^*f*^		4-F_3_CC_6_H_4_ (**3pa**)	40	98	91
17		4-O_2_NC_6_H_4_ (**3qa**)	36	84	87
18^*g*^		4-PhC_6_H_4_ (**3ra**)	12	70	93
19^*d*^ ^,^^*g*^		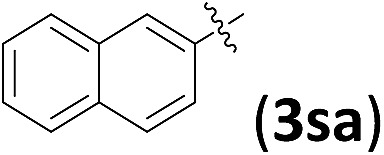	12	94	93
20		Cyclohexyl (**3ua**)	36	0	—

^*a*^Unless otherwise noted, the reaction conditions were the same as entry 10, Table 1.

^*b*^Isolated yield by flash chromatography using basic Al_2_O_3_.

^*c*^Determined by chiral HPLC analysis.

^*d*^The absolute configuration was assigned as (2*R*, 5*S*) by CD analysis and inferred from **3sa** whose absolute configuration was determined by X-ray analysis.^11^

^*e*^The yield was determined by ^1^H NMR.

^*f*^Nd(OTf)_3_/**L-PiPr_2_** (5 mol%, 2 : 1).

^*g*^PhCHO (0.3 mmol, 3 equiv.).

The reaction also tolerated diverse aromatic aldehydes (see Table 3). In order to reach satisfactory reactivity and enantioselectivity, the amount of Nd(OTf)_3_, **L-PiPr_2_** and aldehyde needed to be adjusted. A variety of benzaldehydes with electron withdrawing or donating substituents provided *cis*-1,3-oxazolidines in moderate to high yields (38–73%) with good enantioselectivities (84–94% ee) (entries 1–6). Remarkably, heteroaromatic aldehydes (such as furan-2-carbaldehyde and thiophene-3-carbaldehyde) delivered the desired products in high yields (84–93%) and enantioselectivities (91–94% ee) (entries 7–8). Additionally, cinnamaldehyde displayed high reactivity but gave only a moderate ee value (entry 9). Only trace amounts of the expected adduct were detected when using an aliphatic aldehyde (entry 10). Overall, only *cis*-1,3-oxazolidines were attained. To evaluate the synthetic utility of this catalyst system, a gram-scale preparation of **3kh** was undertaken, resulting in an outcome of 93% yield, >19 : 1 dr and 93% ee.

**Table 3:** Substrate scope of aldehydes^*a*^

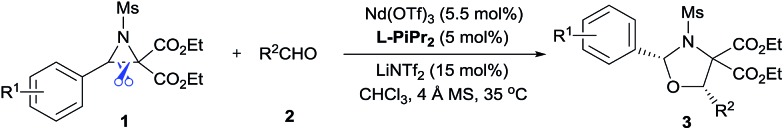						
Entry	R^1^	R^2^	**1** : **2**	*t* (h)	Yield^*b*^ (%)	ee^*c*^ (%)
1^*d*^	H	4-ClC_6_H_4_	1 : 3	24	70 (**3ib**)	90
2^*e*^	3-Cl	3-ClC_6_H_4_	1 : 2	66	38 (**3lc**)	89
3^*f*^	4-Cl	4-MeC_6_H_4_	1 : 2	24	51 (**3kd**)	84
4^*f*^	4-Cl	3-MeC_6_H_4_	1 : 1.5	24	73 (**3ke**)	94
5^*f*^	3-Me	3-MeC_6_H_4_	1 : 2	12	51 (**3te**)	92
6^*f*^	4-Cl	3-MeOC_6_H_4_	1 : 2	24	70 (**3kf**)	93
7^*f*^	4-Cl	3-Thienyl	1 : 1.5	27	84 (**3kg**)	91
8^*g*^	4-Cl	2-Furyl	1 : 1.5	27	93 (**3kh**)	94
9^*f*^	4-Cl	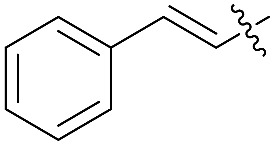	1 : 2	30	84 (**3ki**)	55
10	4-Cl	Cyclohexyl	1 : 2	40	Trace	—

^*a*^Unless otherwise noted, the reactions were performed with Nd(OTf)_3_ (5.5 mol%), **L-PiPr_2_** (5 mol%), LiNTf_2_ (15 mol%), 4 Å MS (100 mg), aziridine **1** (0.1 mmol) and aldehyde **2** in CHCl_3_ (0.75 mL) under nitrogen at 35 °C for the indicated time.

^*b*^Isolated yield by flash chromatography using basic Al_2_O_3_.

^*c*^Determined by chiral HPLC analysis.

^*d*^Nd(OTf)_3_/**L-PiPr_2_** (2.5 mol%, 2 : 1) was used.

^*e*^Nd(OTf)_3_/**L-PiPr_2_** (5 mol%, 2 : 1) was used.

^*f*^The absolute configuration was assigned as (2*R*, 5*S*) by CD analysis referred to **3sa**.

^*g*^With the absolute configuration of (2*R*, 5*R*).

**Fig. 1:**
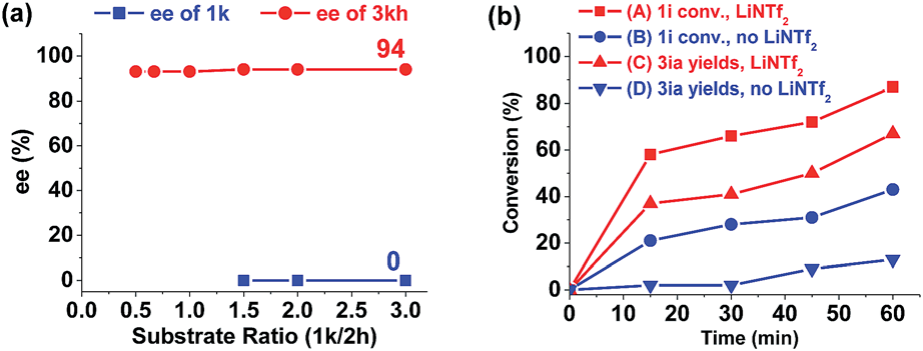
(a) ee traces of the product **3kh** and the substrate **1k** of the reaction. (b) Reaction time profile of the reactions with or without LiNTf_2_.

To gain insights into the reaction process, several control experiments were conducted. In the standard reaction conditions, excess aldehyde **2h** was used and the desired *cis*-product **3kh** was given in 93% yield and 94% ee (Table 3, entry 8). When the ratio of aziridine **1k** to aldehyde **2h** was increased, the enantioselectivity of the product **3kh** was maintained and the remaining aziridine **1k** was racemic (Fig. 1a),12*a* indicating that the reaction occurs through the formation of an azomethine ylide intermediate.^13^

To better understand the catalyst system, HRMS experiments were further explored. ESI-MS species assigned to [**L-PiPr_2_** + Li^+^]^+^ and [**L-PiPr_2_** + Nd^3+^ + OTf^–^]^2+^ were detected from separate mixtures of the ligand with each of the metal salts. However, upon mixing the three components together (**L-PiPr_2_**/Nd(OTf)_3_/LiNTf_2_ = 1 : 1.1 : 3), only signals related to the complexes of **L-PiPr_2_**/Nd(iii) were observed (see the ESI†), suggesting the high stereocontrol might originate from the chiral Nd(iii) complex of **L-PiPr_2_**.

**Scheme 2:**
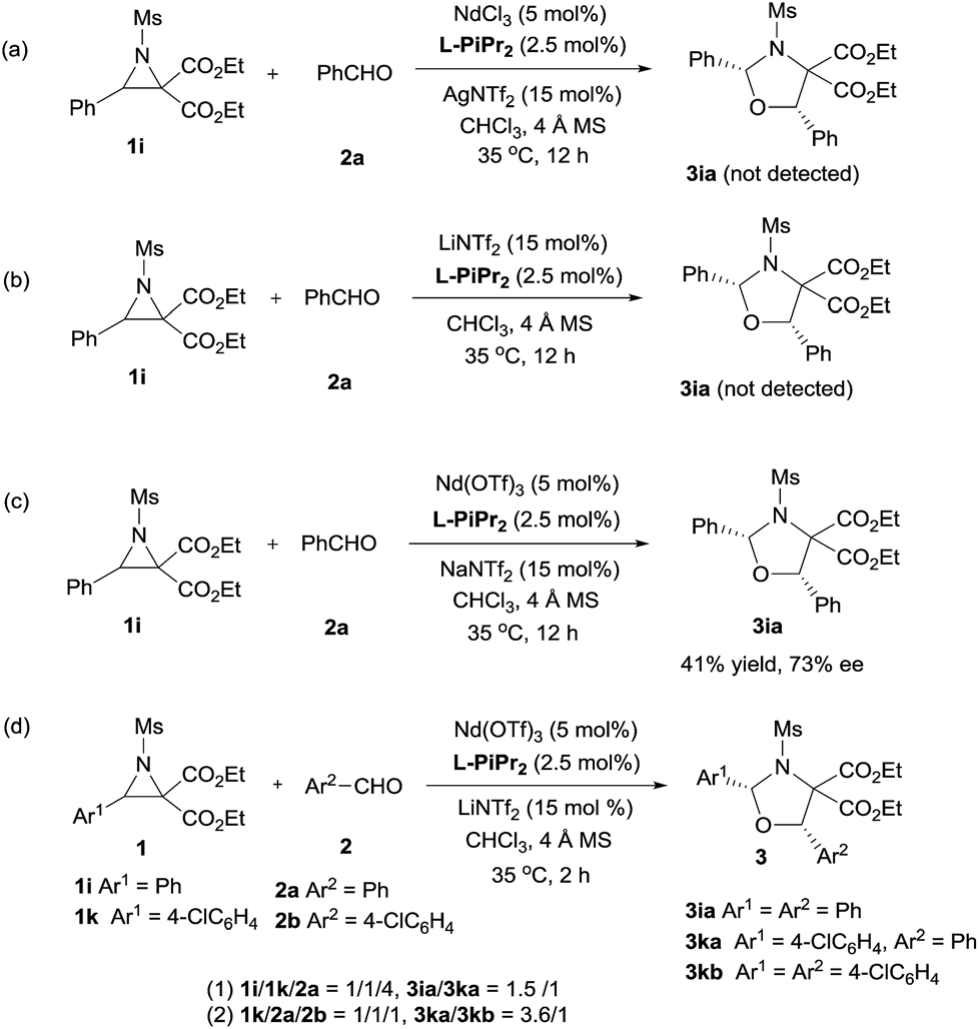
Control experiments.

Next, the primary actions of the catalyst components were studied. When mixtures of NdCl_3_/AgNTf_2_/**L-PiPr_2_** or LiNTf_2_/**L-PiPr_2_** were used, the desired [3 + 2] cycloadducts were not observed but byproducts were detected (Scheme 2a and b). In addition, the utilization of NaNTf_2_ in place of LiNTf_2_ deteriorated the results (41% yield/73% ee *vs.* 77% yield/95% ee, Scheme 2c), which implies that the lithium salt does not merely provide a counter-anion and participate in the cycloaddition step. A reaction time profile of the transformation between aziridine **1i** and benzaldehyde **2a** shows that the *cis*-1,3-oxazolidine **3ia** formed gradually accompanied by the consumption of aziridine **1i**. It is also evident from Fig. 1b that the reaction rate was faster in the presence of, rather than the absence of, LiNTf_2_. A ^1^H NMR spectroscopy study of aziridine **1r** revealed that LiNTf_2_ could obviously accelerate the generation of 4-PhC_6_H_4_CHO and MsNHCH(CO_2_Et)_2_, which forms when the azomethine ylide is trapped by water. Therefore, it is reasonable to conclude that LiNTf_2_ could promote the cleavage of the aziridine to generate the azomethine ylide intermediate (see the ESI†).^14^ Moreover, competition reactions show that the rate of cycloaddition was more sensitively influenced by the electronic nature of the aldehydes (Scheme 2d).12b,^15^ This implies that the aldehydes function as electron rich dipolarphiles and the cycloaddition step is more likely to be the rate-determining step in comparison with the carbon–carbon cleavage in this case.

**Scheme 3:**
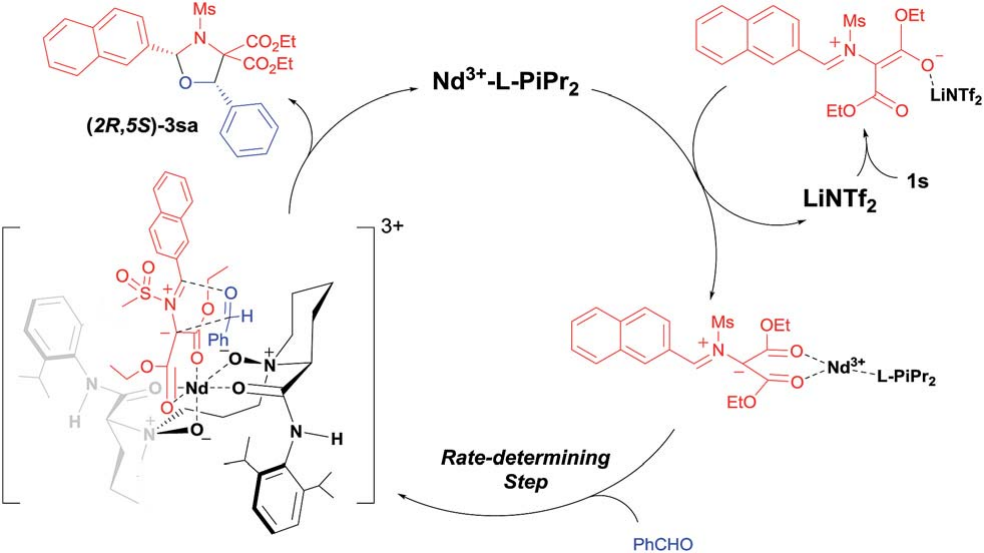
A plausible catalytic cycle.

Based on above mentioned results and our previous study on *N*,*N*′-dioxide–metal complex catalysis,^8^ we propose a dual Lewis acids relay catalysis process (Scheme 3).^16^ Firstly, with the assistance of LiNTf_2_, the carbon–carbon bond of the DA aziridine is cleaved to form a dipolar intermediate. It is then caught by the chiral Nd(iii)/**L-PiPr_2_** complex due to the strong bidentate coordination of the two ester groups to the metal center. A concerted [3 + 2] cycloaddition occurs enantioselectively to give *cis*-(2*R*,5*S*)-1,3-oxazolidine **3sa**, liberating the catalysts.

## Conclusions

In summary, we have disclosed an enantioselective [3 + 2] annulation of donor–acceptor aziridines with aldehydes through C–C bond cleavage. LiNTf_2_ and a chiral *N*,*N*′-dioxide/Nd(OTf)_3_ complex worked as relay catalysts to promote the reaction under mild reaction conditions. The protocol allowed the efficient production of a variety of enantiomerically enriched *cis*-1,3-oxazolidines. Additional research on expanding the asymmetric transformations of DA aziridines to other dipolarphiles is under way.

## Supplementary Material

Supplementary information

Crystal structure data

## References

[cit1] (c) Callebaut G. Meiresonne T. Kimpe N. D. Mangelinckx S., Chem. Rev., 2014, 114 , 7954 , . For recent examples . 24773209 10.1021/cr400582d

[cit2] Trost B. M., Fandrick D. R. (2003). J. Am. Chem. Soc..

[cit3] Chai Z., Zhu Y.-M., Yang P.-J., Wang S., Wang S., Liu Z., Yang G. (2015). J. Am. Chem. Soc..

[cit4] Huisgen R., Scheer W., Huber H. (1967). J. Am. Chem. Soc..

[cit5] Pohlhaus P. D., Bowman R. K., Johnson J. S. (2004). J. Am. Chem. Soc..

[cit6] (b) de Nanteuil F. De Simone F. Frei R. Benfatti F. Serrano E. Waser J., Chem. Commun., 2014, 50 , 10912 , . For selected examples . 10.1039/c4cc03194f 24970590

[cit7] Chen W. L., Lin L. L., Cai Y. F., Xia Y., Cao W. D., Liu X. H., Feng X. M. (2014). Chem. Commun..

[cit8] (b) Liu X. H. Lin L. L. Feng X. M., Org. Chem. Front., 2014, 1 , 298 , . Selected examples .

[cit9] Hirayama S., Wada N., Nemoto T., Iwai T., Fujii H., Nagase H. (2014). ACS Med. Chem. Lett..

[cit10] Caldwell J. J., Craig D. (2007). Angew. Chem., Int. Ed..

[cit11] CCDC 1057118 ((2*R*, 5*S*)-**3sa**)

[cit12] Jin M., Adak L., Nakamura M. (2015). J. Am. Chem. Soc..

[cit13] The computation revealed that the DA aziridine went through the azomethine ylide intermediate during the [3 + 2] cycloaddition, see: Paasche A. Arnone M. Fink R. F. Schirmeister T. Engels B., J. Org. Chem., 2009, 74 , 5244 . 19719251 10.1021/jo900505q

[cit14] The azomethine ylide intermediate has been detected by NMR through mixing LiClO_4_ with donor–acceptor *N*-arylaziridines, see: Vaultier M. Carrie R., Tetrahedron Lett., 1978, 19 , 1195 .

[cit15] The desired yield sharply decreased in 100% aziridine consumption when the electron density of aryl groups on aldehydes was reduced (Table 2, entries 9 and 12 *vs.*Table 3, entry 2)

[cit16] (a) Chen D.-F. Han Z.-Y. Zhou X.-L. Gong L.-Z., Acc. Chem. Res., 2014, 47 , 2365 , . For selected examples . 24911184 10.1021/ar500101a

