# Supplementary material for: Asymmetric [3 + 2] cycloaddition of donor–acceptor aziridines with aldehydes via carbon–carbon bond cleavage
Source: Chem Sci. 2016 Feb 23;7(6):3775–9. doi: 10.1039/c5sc04151a (PMC6013814; doi:10.1039/c5sc04151a)
Supplement: Supplementary file 1 [file SC-007-C5SC04151A-s001.pdf]

Electronic Supplementary Material (ESI) for Chemical Science.
This journal is © The Royal Society of Chemistry 2016

Supporting Information

CONTENTS:

(A) General remarks ……………………………………………………………S2

(B) Preparation of aziridines …………………………………………………...S2

(C) The analytical and spectral characterization data of aziridines …………………S3

(D) General procedure for chiral *N,N'*-dioxides preparation ………….………...S11

(E) General procedure for the preparation of the racemic products ……………….S11

(F) General procedure for the catalytic asymmetric transformation ………………S11

(G) Experimental procedure for the scale-up reaction ……………………………..S12

(H) Control experiments ……………………………………………………...S12

(I) A plausible catalytic cycle ……..…………………………………………S17

(J) Optimization of conditions ………………………………………………S18

(K) The analytical and spectral characterization data of products …………………S20

(L) References ………………………………………………………………..S46

(M) The X-ray data for **3sa** …………….……………………………….......S47

(N) Copies of NMR spectra ………………………………………………..S49

(O) Copies of CD spectra …………………………………………………S126

(A) General remarks

^1H NMR spectra were recorded on commercial instruments (400 MHz). Chemical shifts were recorded in ppm relative to tetramethylsilane and with the solvent resonance as the internal standard ($CDCl_3$, $\delta = 7.26$). Data were reported as follows: chemical shift, multiplicity (s = singlet, d = doublet, t = triplet, q = quartet, m = multiplet), coupling constants (Hz), integration. ^{13}C NMR data were collected on commercial instruments (100 MHz) with complete proton decoupling. Chemical shifts were reported in ppm from the tetramethylsilane with the solvent resonance as internal standard ($CDCl_3$, $\delta = 77.0$; DMSO-d_6, $\delta = 39.5$). Enantiomeric excesses were determined by chiral HPLC analysis on Daicel Chiralcel IE, ID and IA at 23 °C with UV detector at 210 nm in comparison with the authentic racemates. Optical rotations were reported as follows: $[\alpha]_\lambda^T$ (c: g/100 mL, in solvent, λ). HRMS was recorded on a commercial apparatus (ESI source). All the reactions were carried out under an atmosphere of nitrogen in oven-dried apparatus. All the solvents were purified by usual methods before use. Molecular sieves were activated at 500 °C for 5 h before use. All the liquid aldehydes were freshly distilled prior to use. All the solid aldehydes were used after recrystallization with petroleum ether. All the imines were prepared according to literature.[1] Chromatography: Silica gel (HG/T2354-2010) made in Qingdao Haiyang Chemical Co., Ltd; Basic aluminum oxide (pH = 9-10) made in Shanghai Ludu Chemical Co., Ltd.

(B) Preparation of aziridines

Method A[2a]

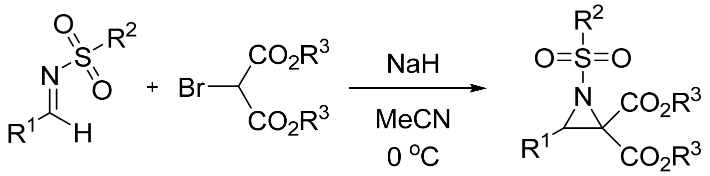

General Procedure: Under N_2 atmosphere, to a solution of imine (5.0 mmol) and 2-bromomalonate (5.5 mmol) in dry MeCN (50 mL) were added NaH (5.5 mmol) at 0 °C. After 20 min, the mixture was filtrated through a thin layer of silica gel with

CH_2Cl_2. The filtrates were concentrated and quickly purified by flash chromatography (Eluent: Ethyl acetate:Petroleum ether = 1:10 - 3:7) to afford the corresponding aziridines. (Ease for gram-scale preparation)

Method B[2b]

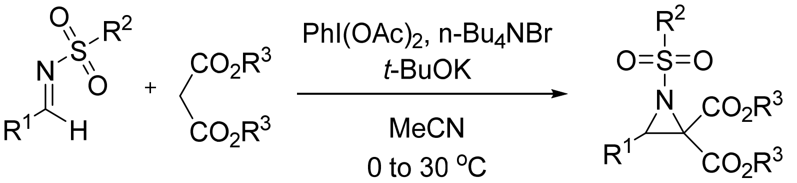

General Procedure: To a solution of imine (5.0 mmol) and $CH_2(CO_2R^3)_2$ (6.0 mmol) in anhydrous MeCN (45 mL) were added $PhI(OAc)_2$ (10.0 mmol), n-Bu_4NBr (10.0 mmol) and t-BuOK (2.5 mmol) at 0 °C. The reaction mixture was warmed up to 30 °C and continuously stirred for approximately 3 h. Then the resultant suspensions were filtered, concentrated, directly purified by flash column chromatography (Eluent: Ethyl acetate:Petroleum ether = 1:10 - 3:7) to provide the corresponding aziridines.

(C) The analytical and spectral characterization data of aziridines

Diethyl 3-phenyl-1-tosylaziridine-2, 2′-dicarboxylate (1a)

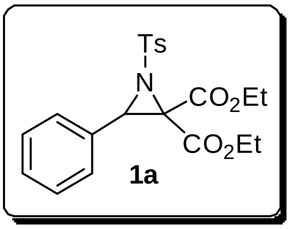

Prepared by *Method A*. Colorless oil, 86% yield. ^{1}H NMR (400 MHz, $CDCl_3$) δ = 7.96 (d, J = 8.4 Hz, 2H), 7.35 (d, J = 8.0 Hz, 2H), 7.28 - 7.21 (m, 5H), 4.88 (s, 1H), 4.43 - 4.35 (m, 2H), 3.95 (dd, J = 7.2 Hz, 14.0 Hz, 2H), 2.44 (s, 3H), 1.37 (t, J = 7.2 Hz, 3H), 0.88 (t, J = 7.2 Hz, 3H); ^{13}C NMR (101 MHz, $CDCl_3$) δ = 163.1, 162.5, 144.7, 136.6, 131.0, 129.7, 128.8, 128.4, 127.7, 127.0, 63.4, 62.1, 57.5, 49.7, 21.7, 13.8, 13.6. HRMS (ESI-TOF) calcd for $C_{21}H_{23}KNO_6S^+$ ([M+K$^+$]) = 456.0878, Found 456.0870.

Dimethyl 3-phenyl-1-tosylaziridine-2, 2′-dicarboxylate (1b)

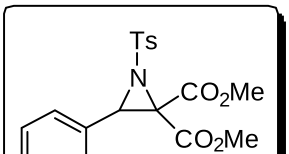

1b

Prepared by *Method A*. Colorless oil, 80% yield. ^{1}H NMR (400 MHz, $CDCl_3$) δ = 7.94 (d, J = 8.0 Hz, 2H), 7.35 (d, J = 8.0 Hz, 2H), 7.29 - 7.20 (m, 5H), 4.89 (s, 1H), 3.92 (s, 3H), 3.47 (s, 3H), 2.43 (s, 3H); ^{13}C NMR (101 MHz, $CDCl_3$) δ = 163.6, 163.0, 145.0, 136.2, 130.9, 129.8, 129.0, 128.5, 127.7, 126.9, 57.3, 54.1, 53.0, 49.8, 21.7. HRMS (ESI-TOF) calcd for $C_{19}H_{19}NNaO_6S^+$ ([M+Na$^+$]) = 412.0826, Found 412.0835.

Diisopropyl 3-phenyl-1-tosylaziridine-2, 2′-dicarboxylate (1c)

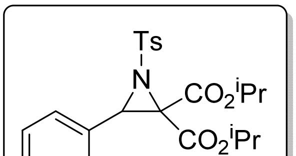

1c

Prepared by *Method B*. Colorless oil, 42% yield. ^{1}H NMR (400 MHz, $CDCl_3$) δ = 7.99 - 7.95 (m, 2H), 7.36 - 7.32 (m, 2H), 7.27 - 7.20 (m, 5H), 5.28 - 5.20 (m, 1H), 4.88 (s, 1H), 4.83 - 4.75 (m, 1H), 2.44 (s, 3H), 1.37 (d, J = 6.4 Hz, 3H), 1.33 (d, J = 6.0 Hz, 3H), 1.05 (d, J = 6.0 Hz, 3H), 0.72 (d, J = 6.4 Hz, 3H); ^{13}C NMR (101 MHz, $CDCl_3$) δ = 162.6, 161.9, 144.6, 136.9, 131.2, 129.7, 128.8, 128.3, 127.6, 127.0, 71.4, 69.9, 57.8, 49.8, 21.7, 21.4, 21.1. HRMS (ESI-TOF) calcd for $C_{23}H_{27}NNaO_6S^+$ ([M+Na$^+$]) = 468.1452, Found 468.1454.

Diethyl 3-phenyl-1-(4-chlorobenzenesulfonyl)aziridine-2, 2′-dicarboxylate (1d)

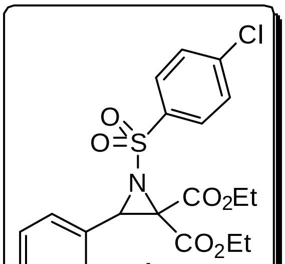

1d

Prepared by *Method A*. Colorless oil, 66% yield. ^{1}H NMR (400 MHz, $CDCl_3$) δ = 8.03 (d, J = 8.4 Hz, 2H), 7.54 (d, J = 8.8 Hz, 2H), 7.30 - 7.25 (m, 3H), 7.25 - 7.20 (m, 2H), 4.93 (s, 1H), 4.40 (dd, J = 6.4 Hz, 13.6 Hz, 2H), 3.96 (dd, J = 6.8 Hz, 14 Hz, 2H), 1.37 (t, J = 7.2 Hz, 3H), 0.89 (t, J = 7.2 Hz, 3H); ^{13}C NMR (101 MHz, $CDCl_3$) δ = 163.0, 162.3, 140.4, 138.2, 130.8, 129.5, 129.1, 128.5, 126.9, 63.6, 62.3, 57.7, 50.1, 13.8, 13.6. HRMS (ESI-TOF) calcd for $C_{20}H_{21}{}^{34.9689}ClNO_6S^+$ ([M+H$^+$]) = 438.0773, Found 438.0774. HRMS (ESI-TOF) calcd for $C_{20}H_{21}{}^{36.9659}ClNO_6S^+$ ([M+H$^+$]) = 440.0744, Found 440.0765.

Diethyl 3-phenyl-1-benzenesulfonylaziridine-2, 2′-dicarboxylate (1e)

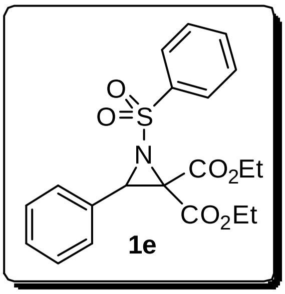

Prepared by *Method A*. Colorless oil, 91% yield. ^{1}H NMR (400 MHz, $CDCl_3$) δ = 8.09 (d, *J* = 7.6 Hz, 2H), 7.65 (t, *J* = 7.2 Hz, 1H), 7.56 (t, *J* = 7.2 Hz, 2H), 7.29 - 7.20 (m, 5H), 4.92 (s, 1H), 4.40 (dd, *J* = 6.8 Hz, 14.0 Hz, 2H), 3.95 (dd, *J* = 7.2 Hz, 14.0 Hz, 2H), 1.37 (t, *J* = 6.8 Hz, 3H), 0.88 (t, *J* = 7.2 Hz, 3H); ^{13}C NMR (101 MHz, $CDCl_3$) δ = 163.1, 162.5, 139.6, 133.8, 130.9, 129.1, 128.9, 128.4, 127.6, 127.0, 63.5, 62.2, 57.5, 49.9, 13.8, 13.6. HRMS (ESI-TOF) calcd for $C_{20}H_{21}NNaO_6S^+$ ([M+Na$^+$]) = 426.0982, Found 426.0987.

Diethyl 3-phenyl-1-(4-methoxylbenzenesulfonyl)aziridine-2, 2′-dicarboxylate (1f)

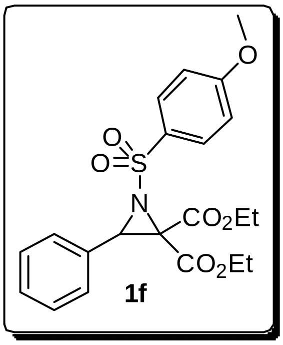

Prepared by *Method A*. Colorless oil, 98% yield. ^{1}H NMR (400 MHz, $CDCl_3$) δ = 8.00 (d, *J* = 9.2 Hz, 2H), 7.30 - 7.20 (m, 5H), 7.01 (d, *J* = 8.8 Hz, 2H), 4.86 (s, 1H), 4.44 - 4.33 (m, 2H), 3.94 (dd, *J* = 7.2 Hz, 14.4 Hz, 2H), 3.85 (s, 3H), 1.36 (t, *J* = 7.2 Hz, 3H), 0.87 (t, *J* = 6.8 Hz, 3H); ^{13}C NMR (101 MHz, $CDCl_3$) δ = 163.8, 163.1, 162.5, 131.0, 130.8, 130.0, 128.8, 128.4, 127.0, 114.3, 63.3, 62.1, 57.4, 55.7, 49.6, 13.8, 13.6. HRMS (ESI-TOF) calcd for $C_{21}H_{23}NNaO_7S^+$ ([M+Na$^+$]) = 456.1088, Found 456.1088.

Diethyl 3-phenyl-1-(2-methylbenzenesulfonyl)aziridine-2, 2′-dicarboxylate (1g)

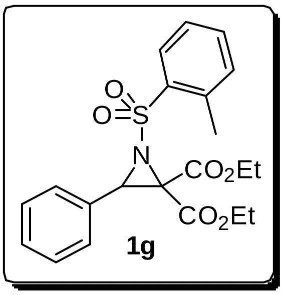

Prepared by *Method A*. Colorless oil, 46% yield. ^{1}H NMR (400 MHz, $CDCl_3$) δ = 8.00 (d, *J* = 7.6 Hz, 1H), 7.48 (t, *J* = 7.2 Hz, 1H), 7.37 (d, *J* = 7.2 Hz, 1H), 7.30 - 7.18 (m, 6H), 4.97 (s, 1H), 4.44 - 4.27 (m, 2H), 3.98 - 3.84 (m, 2H), 2.93 (s, 3H), 1.32 (t, *J* = 7.2 Hz, 3H), 0.85 (t, *J* = 7.2 Hz, 3H); ^{13}C NMR (101 MHz, $CDCl_3$) δ = 163.0, 162.6, 139.1, 137.4, 134.0, 132.7, 131.1, 129.1, 129.0, 128.4, 126.9, 126.1, 63.3, 62.1, 57.4, 49.9, 20.5, 13.7, 13.6. HRMS (ESI-TOF) calcd for $C_{21}H_{24}NO_6S^+$ ([M+H$^+$]) = 418.1319, Found 418.1321.

Diethyl 3-phenyl-1-(2-nitrobenzenesulfonyl)aziridine-2, 2′-dicarboxylate (1h)

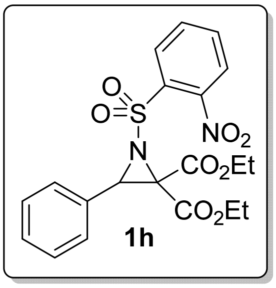

Prepared by *Method A*. White solid (Crystallized by Et_2O/petroleum ether), 74% yield. ^1H NMR (400 MHz, $CDCl_3$) δ = 8.48 - 8.32 (m, 1H), 7.94 - 7.76 (m, 3H), 7.38 - 7.27 (m, 5H), 5.12 (s, 1H), 4.42 (dd, J = 7.2, 14.4 Hz, 2H), 4.08 - 3.92 (m, 2H), 1.39 (t, J = 7.2 Hz, 3H), 0.90 (t, J = 7.2 Hz, 3H); ^{13}C NMR (101 MHz, $CDCl_3$) δ = 163.2, 162.3, 147.9, 134.5, 133.9, 132.91, 131.0, 130.9, 129.0, 128.4, 126.9, 125.1, 63.7, 62.3, 58.4, 52.5, 13.8, 13.6. HRMS (ESI-TOF) calcd for $C_{20}H_{20}N_2NaO_8S^+$ ([M+Na$^+$]) = 471.0833, Found 471.0832.

Diethyl 3-phenyl-1-methylsulfonylaziridine-2, 2′-dicarboxylate (1i)

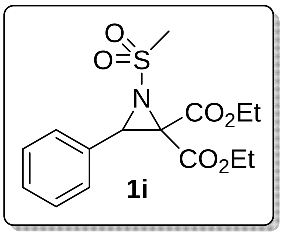

Prepared by *Method A*. White solid (Crystallized by Et_2O/petroleum ether), 76% yield. ^1H NMR (400 MHz, $CDCl_3$) δ = 7.42 - 7.31 (m, 5H), 4.78 (s, 1H), 4.41 - 4.32 (m, 2H), 4.02 (dd, J = 7.2 Hz, 14.4 Hz, 2H), 3.34 (s, 3H), 1.36 (t, J = 7.2 Hz, 3H), 0.93 (t, J = 7.2 Hz, 3H); ^{13}C NMR (101 MHz, $CDCl_3$) δ = 163.0, 162.4, 130.7, 129.1, 128.5, 127.1, 63.5, 62.4, 57.3, 48.3, 41.9, 13.7. HRMS (ESI-TOF) calcd for $C_{15}H_{19}NNaO_6S^+$ ([M+Na$^+$]) = 364.0826, Found 364.0829.

Diethyl 3-phenyl-1-(2-trimethylsilylethanesulfonyl)aziridine-2, 2′-dicarboxylate (1j)

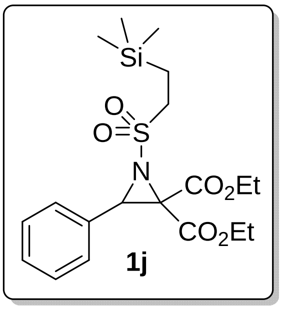

Prepared by *Method A*. Light yellow oil, 22% yield. ^1H NMR (400 MHz, $CDCl_3$) δ = 7.40 - 7.29 (m, 5H), 4.81 (s, 1H), 4.41 - 4.31 (m, 2H), 4.06 - 3.95 (m, 2H), 3.39 - 3.23 (m, 2H), 1.35 (t, J = 7.2 Hz, 3H), 1.33 - 1.20 (m, 2H), 0.94 (t, J = 7.2 Hz, 3H), 0.09 (s, 9H); ^{13}C NMR (101 MHz, $CDCl_3$) δ = 163.2, 162.6, 133.4, 131.3, 130.7, 129.2, 63.4, 62.2, 57.1, 51.5, 48.7, 13.7, 13.6, 9.8, -2.0. HRMS (ESI-TOF) calcd for $C_{19}H_{29}NNaO_6SSi^+$ ([M+Na$^+$]) = 450.1377, Found 450.1385.

Diethyl 3-(4-chlorophenyl)-1-methylsulfonylaziridine-2, 2′-dicarboxylate (1k)

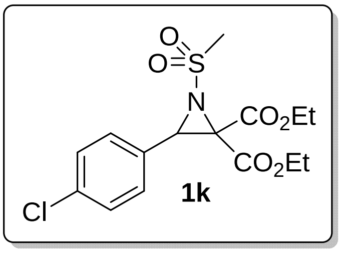

Prepared by *Method A*. White solid (Crystallized by Et_2O/petroleum ether), 74% yield. ^{1}H NMR (400 MHz, $CDCl_3$) δ = 7.39 - 7.29 (m, 4H), 4.72 (s, 1H), 4.36 (dd, *J* = 7.2 Hz, 14.4 Hz, 2H), 4.11 - 3.97 (m, 2H), 3.33 (s, 3H), 1.36 (t, *J* = 7.2 Hz, 3H), 1.00 (t, *J* = 7.2 Hz, 3H); ^{13}C NMR (101 MHz, $CDCl_3$) δ = 162.8, 162.2, 135.1, 129.3, 128.8, 128.6, 63.6, 62.6, 57.4, 47.4, 41.8, 13.8. HRMS (ESI-TOF) calcd for $C_{15}H_{18}{}^{34.9689}ClNNaO_6S^+$ ([M+Na$^+$]) = 398.0436, Found 398.0436. HRMS (ESI-TOF) calcd for $C_{15}H_{18}{}^{36.9659}ClNNaO_6S^+$ ([M+Na$^+$]) = 400.0407, Found 400.0399.

Diethyl 3-(3-chlorophenyl)-1-methylsulfonylaziridine-2, 2′-dicarboxylate (1l)

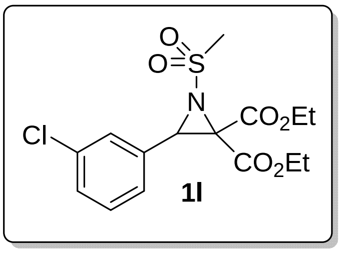

Prepared by *Method A*. Colorless oil, 54% yield. ^{1}H NMR (400 MHz, $CDCl_3$) δ = 7.40 - 7.37 (m, 1H), 7.35 - 7.27 (m, 3H), 4.72 (s, 1H), 4.42 - 4.30 (m, 2H), 4.06 (dd, *J* = 7.2 Hz, 14.4 Hz, 2H), 3.34 (s, 3H), 1.36 (t, *J* = 7.2 Hz, 3H), 0.99 (t, *J* = 7.2 Hz, 3H); ^{13}C NMR (101 MHz, $CDCl_3$) δ = 162.7, 162.2, 134.5, 132.8, 129.9, 129.3, 127.1, 125.6, 63.6, 62.6, 57.2, 47.2, 41.8, 13.8, 13.7. HRMS (ESI-TOF) calcd for $C_{15}H_{18}{}^{34.9689}ClNKO_6S^+$ ([M+K$^+$]) = 414.0175, Found 414.0173. HRMS (ESI-TOF) calcd for $C_{15}H_{18}{}^{36.9659}ClNKO_6S^+$ ([M+K$^+$]) = 416.0146, Found 416.0148.

Diethyl 3-(2-chlorophenyl)-1-methylsulfonylaziridine-2, 2′-dicarboxylate (1m)

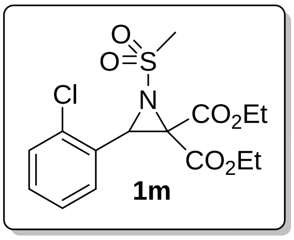

Prepared by *Method A*. Colorless oil, 69% yield. ^{1}H NMR (400 MHz, $CDCl_3$) δ = 7.45 (d, *J* = 7.2 Hz, 1H), 7.37 (d, *J* = 7.6 Hz, 1H), 7.32 - 7.27 (m, 1H), 7.26 - 7.22 (m, 1H), 4.94 (s, 1H), 4.45 - 4.30 (m, 2H), 4.08 - 3.94 (m, 2H), 3.37 (s, 3H), 1.37 (t, *J* = 7.2 Hz, 3H), 0.92 (t, *J* = 7.2 Hz, 3H); ^{13}C NMR (101 MHz, $CDCl_3$) δ = 162.7, 162.2, 134.1, 130.2, 129.2, 129.1, 128.8, 126.7, 63.5, 62.5, 56.5, 46.3, 41.5, 13.7, 13.6. HRMS (ESI-TOF) calcd for

$C_{15}H_{18}{}^{34.9689}ClNNaO_6S^+$ ([M+Na$^+$]) = 398.0436, Found 398.0442. HRMS (ESI-TOF) calcd for $C_{15}H_{18}Na^{36.9659}ClNNaO_6S^+$ ([M+Na$^+$]) = 400.0407, Found 400.0418.

Diethyl 3-(4-bromophenyl)-1-methylsulfonylaziridine-2, 2′-dicarboxylate (1n)

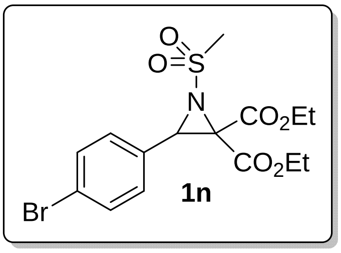

Prepared by *Method A*. White solid (Crystallized by Et_2O/petroleum ether), 58% yield. ^1H NMR (400 MHz, $CDCl_3$) δ = 7.48 (d, J = 8.8 Hz, 2H), 7.28 (d, J = 8.0 Hz, 2H), 4.70 (s, 1H), 4.36 (dd, J = 7.2 Hz, 14.4 Hz, 2H), 4.11 - 3.97 (m, 2H), 3.33 (s, 3H), 1.36 (t, J = 7.2 Hz, 3H), 1.00 (t, J = 7.2 Hz, 3H); ^{13}C NMR (101 MHz, $CDCl_3$) δ = 162.8, 162.2, 131.7, 129.8, 128.8, 123.3, 63.6, 62.6, 57.3, 47.4, 41.8, 13.8. HRMS (ESI-TOF) calcd for $C_{15}H_{18}{}^{78.9183}BrKNO_6S^+$ ([M+K$^+$]) = 457.9670, Found 457.9674. HRMS (ESI-TOF) calcd for $C_{15}H_{18}{}^{80.9163}BrKNO_6S^+$ ([M+K$^+$]) = 459.9650, Found 459.9631.

Diethyl 3-(4-fluorophenyl)-1-methylsulfonylaziridine-2, 2′-dicarboxylate (1o)

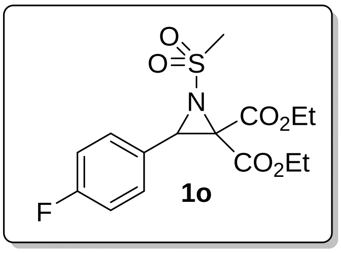

Prepared by *Method A*. White solid (Crystallized by Et_2O/petroleum ether), 66% yield. ^1H NMR (400 MHz, $CDCl_3$) δ = 7.38 (dd, J = 5.2 Hz, 8.4 Hz, 2H), 7.04 (t, J = 8.8 Hz, 2H), 4.73 (s, 1H), 4.36 (dd, J = 7.2 Hz, 14.4 Hz, 2H), 4.04 (dd, J = 7.2 Hz, 14.4 Hz, 2H), 3.33 (s, 3H), 1.36 (t, J = 7.2 Hz, 3H), 0.98 (t, J = 7.2 Hz, 3H); ^{13}C NMR (101 MHz, $CDCl_3$) δ = 163.1 (d, J = 249.4 Hz), 162.9, 162.3, 129.0 (d, J = 8.5 Hz), 126.6 (d, J = 3.1 Hz), 115.6 (d, J = 22.0 Hz), 63.6, 62.5, 57.4, 47.4, 41.8, 13.8; ^{19}F NMR (376 MHz, $CDCl_3$) δ = -112.0. HRMS (ESI-TOF) calcd for $C_{15}H_{18}FNNaO_6S^+$ ([M+Na$^+$]) = 382.0732, Found 382.0735.

Diethyl 3-(4-trifluoromethylphenyl)-1-methylsulfonylaziridine-2, 2′-dicarboxylate (1p)

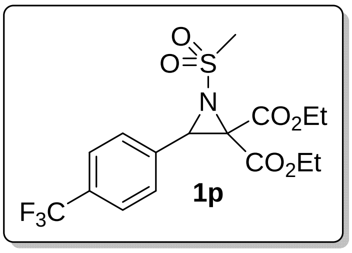

Prepared by *Method A*. White solid (Crystallized by Et_2O/petroleum ether), 77% yield. ^{1}H NMR (400 MHz, $CDCl_3$) δ = 7.61 (d, *J* = 8.0 Hz, 2H), 7.54 (d, *J* = 8.0 Hz, 2H), 4.79 (s, 1H), 4.38 (dd, *J* = 7.2 Hz, 14.4 Hz, 2H), 4.10 - 3.96 (m, 2H), 3.35 (s, 3H), 1.37 (t, *J* = 7.2 Hz, 3H), 0.95 (t, *J* = 7.2 Hz, 3H); ^{13}C NMR (101 MHz, $CDCl_3$) δ = 162.6, 162.0, 134.9, 131.2 (q, *J* = 32.7 Hz), 127.7, 125.4 (q, *J* = 3.7 Hz), 123.8 (d, *J* = 273.3 Hz), 63.6, 62.6, 57.3, 47.2, 41.7, 13.7, 13.6; ^{19}F NMR (376 MHz, $CDCl_3$) δ = -62.8. HRMS (ESI-TOF) calcd for $C_{16}H_{18}F_3KNO_6S^+$ ([M+K$^+$]) = 448.0439, Found 448.0436.

Diethyl 3-(4-nitrophenyl)-1-methylsulfonylaziridine-2, 2′-dicarboxylate (1q)

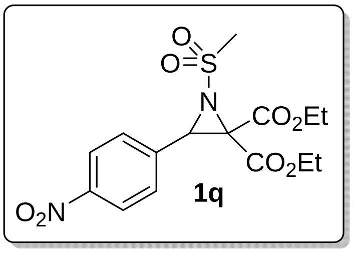

Prepared by *Method A*. White solid (Crystallized by Et_2O/petroleum ether), 74% yield. ^{1}H NMR (400 MHz, $CDCl_3$) δ = 8.22 (d, *J* = 8.8 Hz, 2H), 7.61 (d, *J* = 8.8 Hz, 2H), 4.81 (s, 1H), 4.44 - 4.32 (m, 2H), 4.11 - 3.96 (m, 2H), 3.37 (s, 3H), 1.37 (t, *J* = 7.2 Hz, 3H), 1.00 (t, *J* = 7.2 Hz, 3H); ^{13}C NMR (101 MHz, $CDCl_3$) δ = 162.4, 161.8, 148.3, 137.8, 128.4, 123.7, 63.8, 62.9, 57.4, 46.6, 41.7, 13.8. HRMS (ESI-TOF) calcd for $C_{15}H_{19}N_2O_8S^+$ ([M+H$^+$]) = 387.0857, Found 387.0866.

Diethyl 3-(4-phenylphenyl)-1-methylsulfonylaziridine-2, 2′-dicarboxylate (1r)

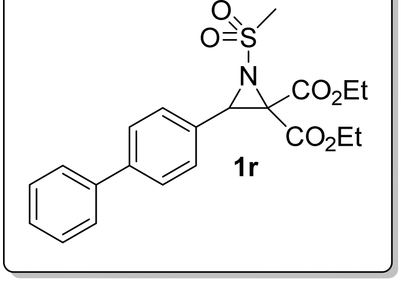

Prepared by *Method A*. White solid (Crystallized by Et_2O/petroleum ether), 60% yield. ^{1}H NMR (400 MHz, $CDCl_3$) δ = 7.60 - 7.54 (m, 4H), 7.50 - 7.41 (m, 4H), 7.39 - 7.33 (m, 1H), 4.81 (s, 1H), 4.42 - 4.32 (m, 2H), 4.10 - 3.98 (m, 2H), 3.35 (s, 3H) 1.37 (t, *J* = 7.2 Hz, 3H), 0.97 (t, *J* = 7.2 Hz, 3H); ^{13}C NMR (101 MHz, $CDCl_3$) δ = 163.0, 162.5, 142.0, 140.3, 129.7, 128.9, 127.7, 127.6, 127.2, 127.1, 63.5, 62.5, 57.4, 48.2, 42.0, 13.8, 13.7. HRMS (ESI-TOF) calcd

for $C_{21}H_{23}KNO_6S^+$ ([M+K$^+$]) = 456.0878, Found 456.0880.

Diethyl 3-(2-naphthyl)-1-methylsulfonylaziridine-2, 2′-dicarboxylate (1s)

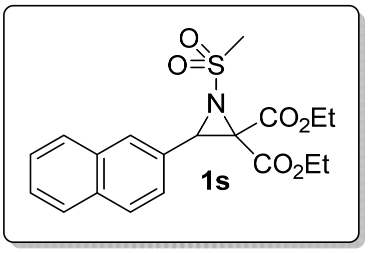

Prepared by *Method A*. White solid (Crystallized by Et_2O/petroleum ether), 55% yield. ^{1}H NMR (400 MHz, $CDCl_3$) δ = 7.89 (s, 1H), 7.87 -7.79 (m, 3H), 7.54 - 7.44 (m, 3H), 4.93 (s, 1H), 4.39 (dd, *J* = 6.8 Hz, 14.0 Hz, 2H), 4.05 - 3.88 (m, 2H), 3.38 (s, 3H), 1.38 (t, *J* = 7.2 Hz, 3H), 0.87 (t, *J* = 7.2 Hz, 3H); ^{13}C NMR (101 MHz, $CDCl_3$) δ = 163.0, 162.5, 133.5, 132.8, 128.4, 128.2, 128.1, 127.8, 126.8, 126.7, 126.6, 124.2, 63.6, 62.5, 57.5, 48.5, 41.9, 13.8, 13.7. HRMS (ESI-TOF) calcd for $C_{19}H_{21}NNaO_6S^+$ ([M+Na$^+$]) = 414.0982, Found 414.0987.

Diethyl 3-(3-methylphenyl)-1-methylsulfonylaziridine-2, 2′-dicarboxylate (1t)

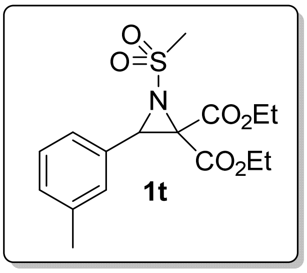

Prepared by *Method A*. Light yellow oil, 45% yield. ^{1}H NMR (400 MHz, $CDCl_3$) δ = 7.25 - 7.10 (m, 4H), 4.72 (s, 1H), 4.40 - 4.30 (m, 2H), 4.06 - 3.96 (m, 2H), 3.31 (s, 3H), 2.32 (s, 3H), 1.34 (t, *J* = 7.2 Hz, 3H), 0.94 (t, *J* = 7.2 Hz, 3H); ^{13}C NMR (101 MHz, $CDCl_3$) δ = 162.7, 162.1, 137.9, 130.4, 129.5, 128.1, 127.3, 123.8, 63.1, 62.0, 56.9, 47.9, 41.5, 20.9, 13.3. HRMS (ESI-TOF) calcd for $C_{16}H_{21}NNaO_6S^+$ ([M+Na$^+$]) = 378.0982, Found 378.0991.

Diethyl 3-cyclohexyl-1-methylsulfonylaziridine-2, 2′-dicarboxylate (1u)

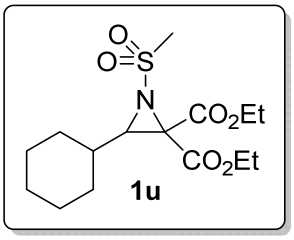

Prepared by *Method A*. White solid (Crystallized by Et_2O/petroleum ether), 20% yield. ^{1}H NMR (400 MHz, $CDCl_3$) δ = 4.39 - 4.24 (m, 4H), 3.36 (d, *J* = 9.6 Hz, 1H), 3.22 (s, 3H), 2.00 - 1.55 (m, 6H), 1.37 - 1.11 (m, 12H); ^{13}C NMR (101 MHz, $CDCl_3$) δ = 163.8, 163.4, 63.1, 62.7, 55.5, 51.1, 41.0, 36.8, 30.8, 29.0, 25.8, 25.1, 14.1, 13.7. HRMS (ESI-TOF) calcd for

$C_{15}H_{25}NNaO_6S^+$ ([M+Na$^+$]) = 370.1295, Found 370.1296.

(D) General procedure for chiral *N,N'*-dioxides preparation

The *N, N'*-dioxide ligands were prepared by the similar procedure in the literatures.[3]

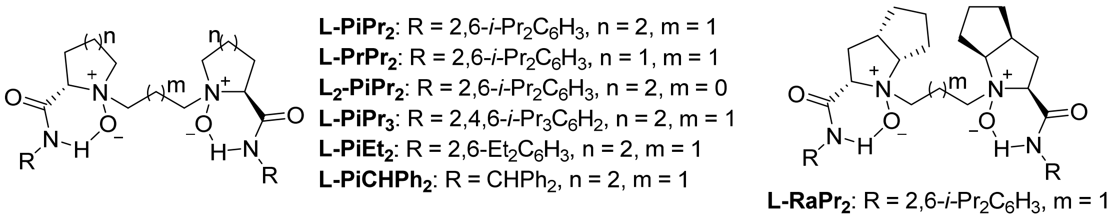

(E) General procedure for the preparation of the racemic products

Some known racemic products (**3aa**, **3ba**, **3ca**, **3da**, **3ea**, **3fa**) were synthesized according to the literature.[4] Other racemic products were prepared as following:

To an oven-dried reaction tube were added $Nd(OTf)_3$ (10 mol%), $LiNTf_2$ (15 mol%), 4 Å molecular sieves (100 mg) and $CHCl_3$ (1.0 mL). Then to the suspensions were added aldehydes (0.3 mmol) and aziridines (0.1 mmol). Then the solutions were stirred at room temperature for 12-40 h. After the completion of the reaction, the suspensions were directly purified by flash chromatography on basic aluminum oxide (pH = 9-10) (Eluent: Ethyl acetate:Petroleum ether = 1:10 - 3:7) to provide the desired products.

(F) General procedure for the catalytic asymmetric transformation

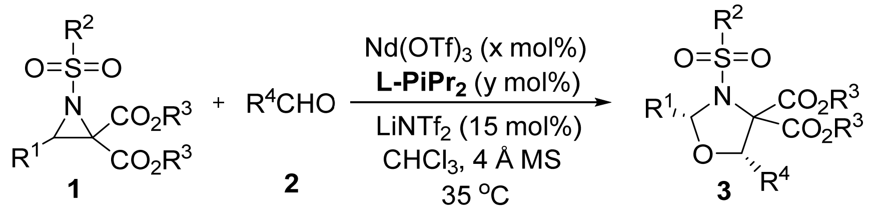

To an oven-dried reaction tube were added $Nd(OTf)_3$ (5-10 mol%), **L-PiPr$_2$** (2.5-5 mol%), $LiNTf_2$ (15 mol%), 4 Å molecular sieves (100 mg) and $CHCl_3$ (0.5 mL). The suspensions were stirred at 35 °C for 0.5 h under nitrogen atmosphere. Subsequently, aldehydes (0.15-0.3 mmol) and aziridines (0.1 mmol) in 0.25 mL of $CHCl_3$ were added. The solutions were stirred at 35 °C for the indicated time. After the completion

of the reactions, the suspensions were directly purified by flash chromatography on basic aluminum oxide (pH = 9-10) (Eluent: Ethyl acetate:Petroleum ether = 1:10 - 3:7) to afford the corresponding products (37-98% yield, >19:1 dr, 55-95% ee).

(G) Experimental procedure for the scale-up reaction

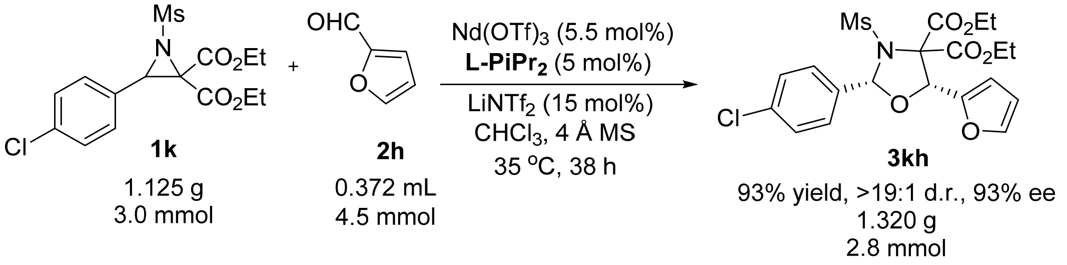

To an oven-dried 50 mL round-bottomed flask were added $Nd(OTf)_3$ (5.5 mol%), **L-PiPr$_2$** (5 mol%), $LiNTf_2$ (15 mol%), 4 Å molecular sieves (3.0 g) and $CHCl_3$ (15 mL). The suspension was stirred at 35 °C for 0.5 h under nitrogen atmosphere. Subsequently, aldehyde **2h** (0.375 mL, 4.5 mmol) and aziridine **1k** (1.125 g, 3.0 mmol) in 7.5 mL of $CHCl_3$ were added. The solution was stirred at 35 °C for 38 h. After the completion of the reaction, the suspension was directly purified by flash chromatography on basic aluminum oxide (pH = 9-10) (Eluent: Ethyl acetate:Petroleum ether = 1:14 - 1:3) to afford the desired product (1.320 g, 93% yield, >19:1 dr, 93% ee).

(H) Control experiments

(1)

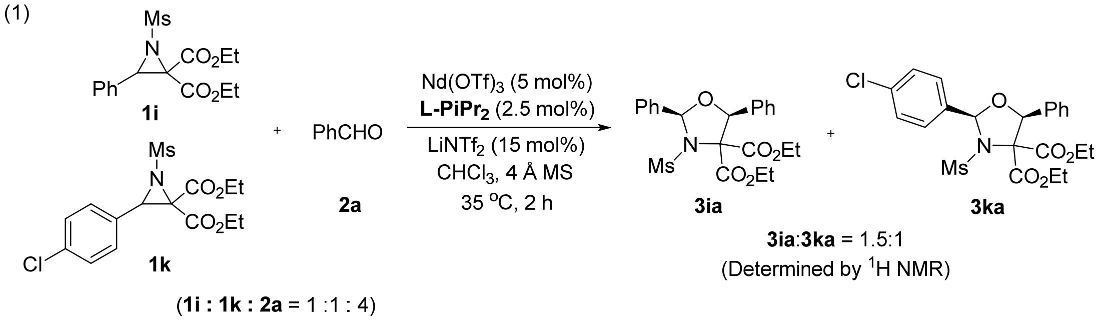

Procedure for control experiment a: To an oven-dried reaction tube were added $Nd(OTf)_3$ (5 mol%), **L-PiPr$_2$** (2.5 mol%), $LiNTf_2$ (15 mol%), 4 Å molecular sieves (100 mg) and $CHCl_3$ (0.5 mL). The suspension was stirred at 35 °C for 0.5 h under nitrogen atmosphere. Subsequently, benzaldehyde **2a** (0.2 mmol), aziridine **1i** (0.05

mmol) and **1k** (0.05 mmol) in 0.25 mL of $CHCl_3$ were added. The solution was stirred at 35 °C for 2 h. After the completion of the reaction, the suspension was directly purified by flash chromatography on basic aluminum oxide (pH = 9-10) (Eluent: Ethyl acetate:Petroleum ether = 1:9 - 3:7) to afford the mixture of **3ia** and **3ka** (**3ia**:**3ka** = 1.5:1, determined by **^{1}H NMR**).

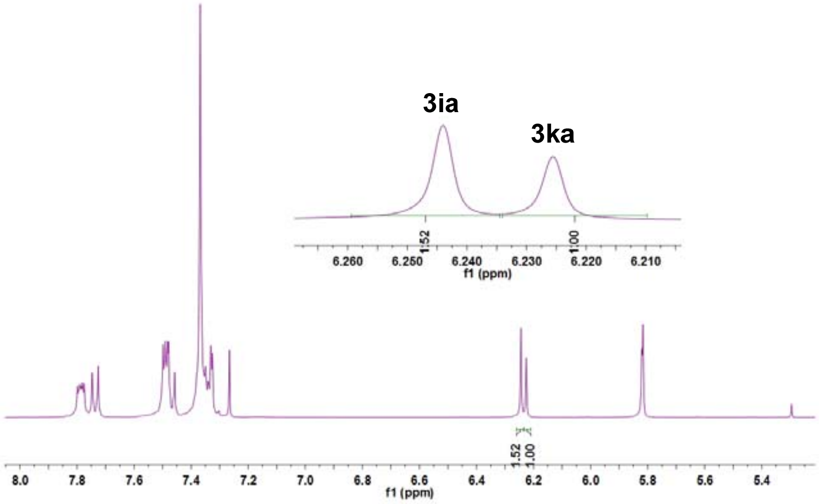

Figure 1 The ratio of **3ia** to **3ka** determined by **^{1}H NMR**

(2)

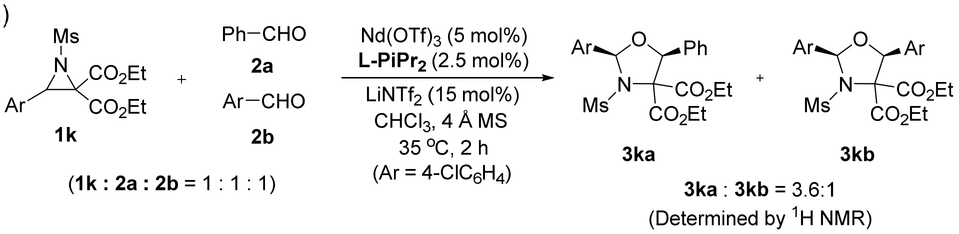

Procedure for control experiment b: To an oven-dried reaction tube were added $Nd(OTf)_3$ (5 mol%), **L-PiPr$_2$** (2.5 mol%), $LiNTf_2$ (15 mol%), 4 Å molecular sieves (100 mg) and $CHCl_3$ (0.5 mL). The suspension was stirred at 35 °C for 0.5 h under nitrogen atmosphere. Subsequently, benzaldehyde **2a** (0.1 mmol), 4-chloro benzaldehyde **2b** (0.1 mmol), and aziridine **1k** (0.1 mmol) in 0.25 mL of $CHCl_3$ were added. The solution was stirred at 35 °C for 2 h. After the completion of the reaction, the suspension was directly purified by flash chromatography on basic aluminum oxide (pH = 9-10) (Eluent: Ethyl acetate:Petroleum ether = 1:9 - 3:7) to afford the mixture of **3ka** and **3kb** (**3ka**:**3kb** = 3.6:1, determined by **^{1}H NMR**).

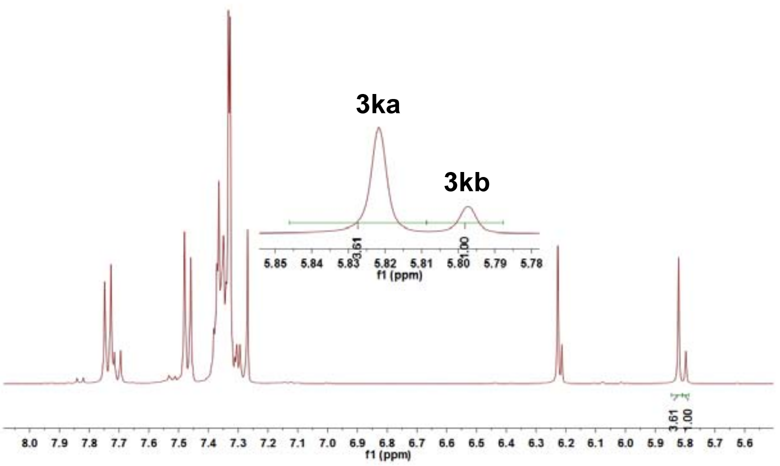

Figure 2 The ratio of **3ka** to **3kb** determined by **^{1}H NMR**

(3) HPLC traces of catalytic asymmetric [3+2]-cycloaddition of aziridine **1k** with aldehyde **2h**.

entry	substrate ratio (x/y)	ee of **1k** (%)	ee of **3kh** (%)
1	1:2	-	93
2	1:1.5	-	93
3	1:1	-	93
4	1.5:1	0	94
5	2:1	0	94
6	3:1	0	94

(4) Kinetic study on catalytic asymmetric [3+2]-cycloaddition of DA aziridine **1i** with aldehyde **2a**.

t/min	15 mol% $LiNTf_2$		no $LiNTf_2$	
	Conversion %	Yield %	Conversion %	Yield %
15	58	37	21	2
30	66	41	28	2
45	72	50	31	9
60	87	67	43	13

(5) ^{1}H NMR experiments.

(a) $LiNTf_2$ and $LiClO_4$ were selected as metal salt respectively. (mixing after 30 min)

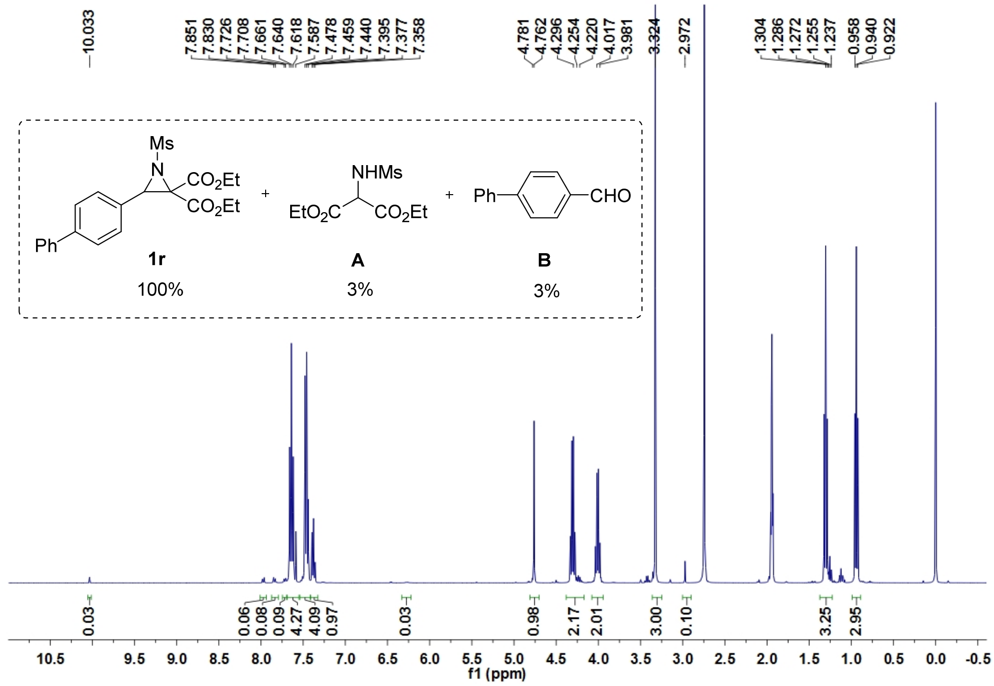

Figure 3 The solution of $LiClO_4$ (0.1 mmol) and aziridine **1r** (0.1 mmol) in CD_3CN (1 mL).

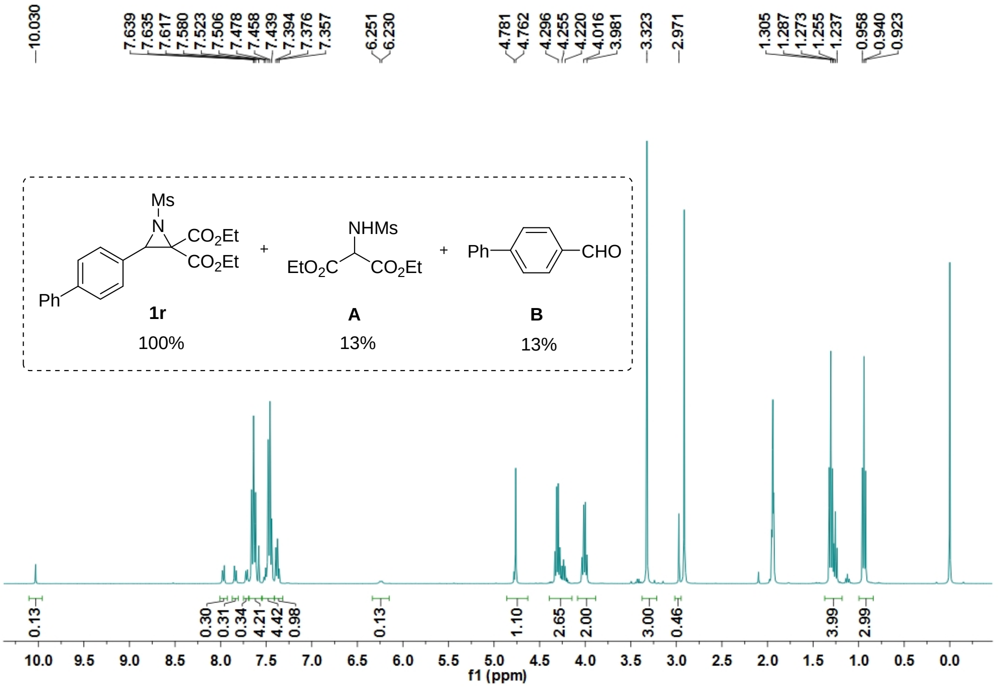

Figure 4 The solution of $LiNTf_2$ (0.1 mmol) and aziridine **1r** (0.1 mmol) in CD_3CN (1 mL).

Note: At first, $LiClO_4$ was selected as metal salt to detect the azomethine ylide intermediate according to previous report.[5] Instead, the side product A and B from the trapping of water were received, might for the unstable intermediate of DA *N*-sulfonylaziridines. Then the same operation

was carried out for $LiNTf_2$, proving its feature of promoting the ring-opening process more easily.

(6) HRMS experiments.

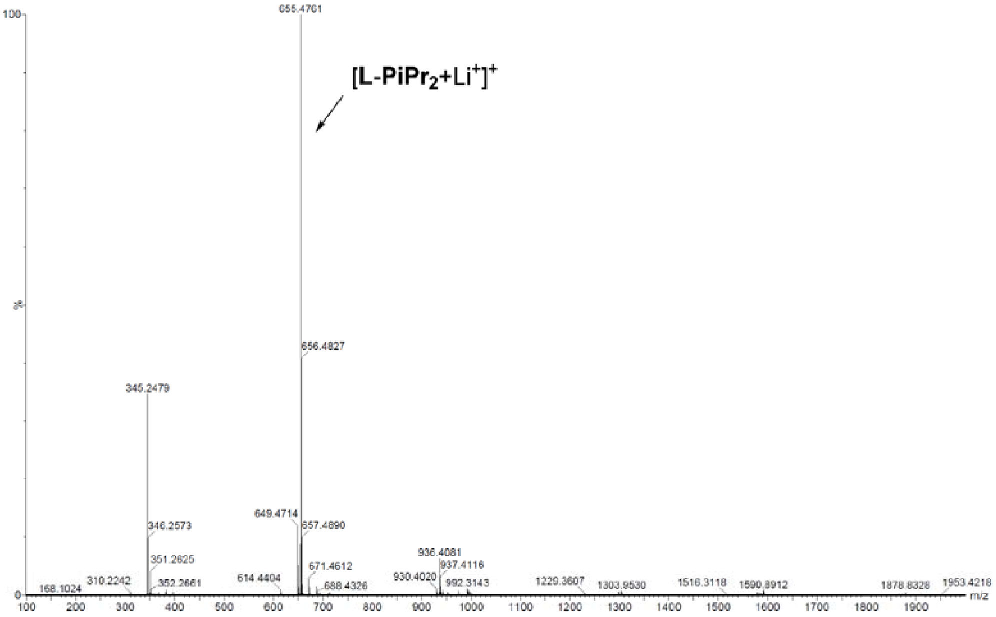

Figure 5 ESI-MS analysis of the mixture of $LiNTf_2$ and **L-PiPr$_2$** (1:1).

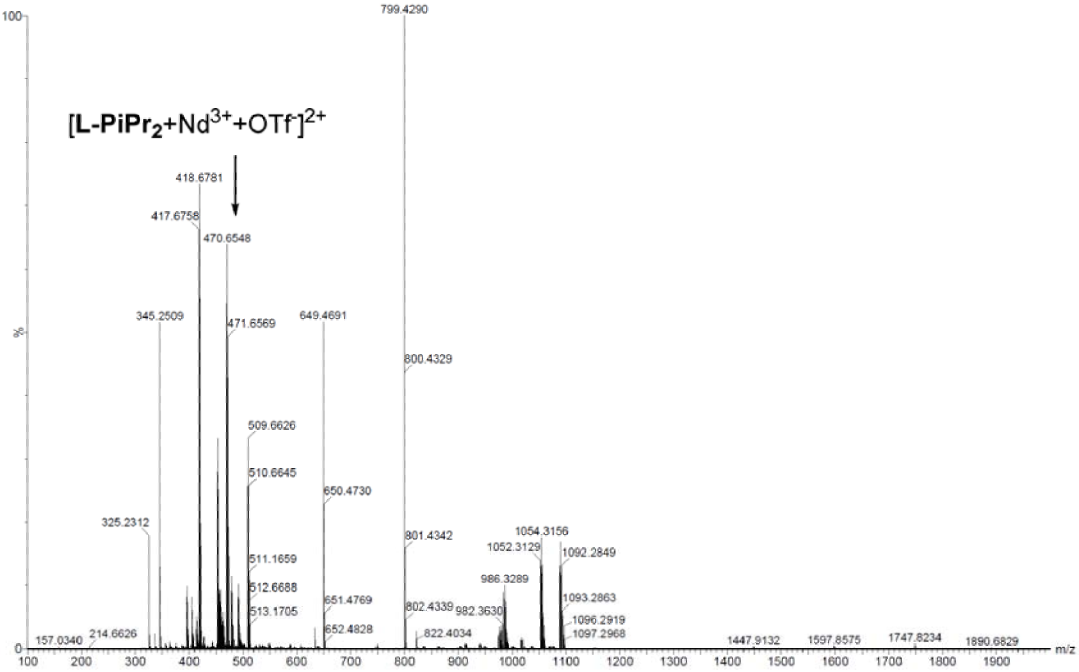

Figure 6 ESI-MS analysis of the mixture of $Nd(OTf)_3$ and **L-PiPr$_2$** (1:1).

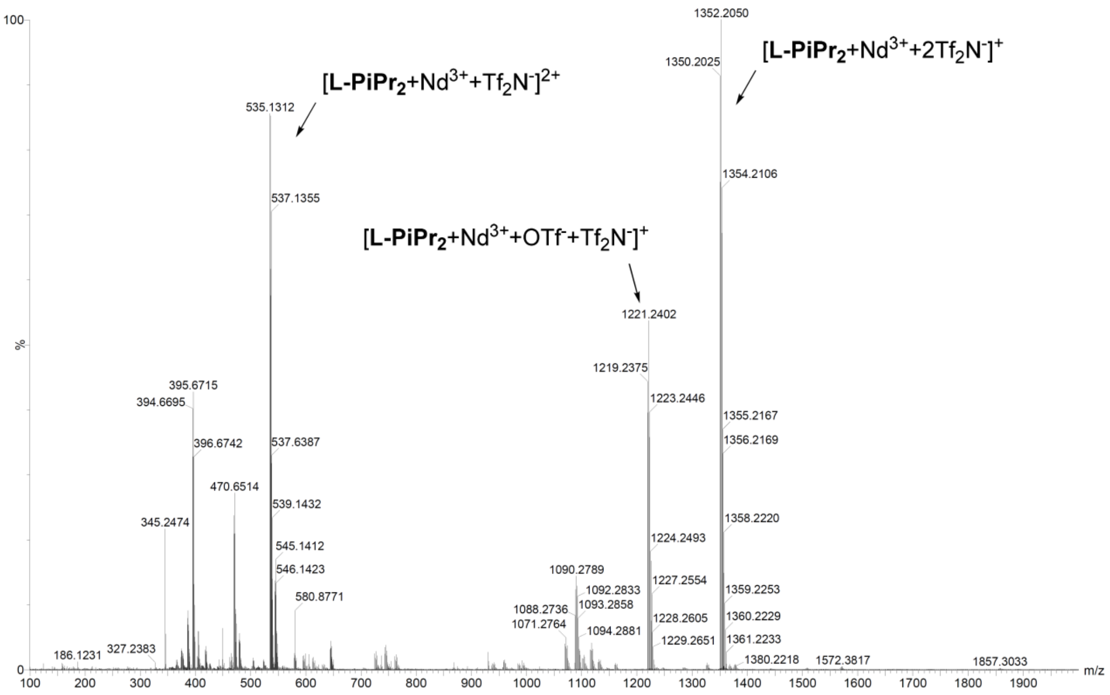

Figure 7 ESI-MS analysis of the mixture of $Nd(OTf)_3$, **L-PiPr$_2$** and $LiNTf_2$ (1.1:1:3).

(I) A plausible catalytic cycle

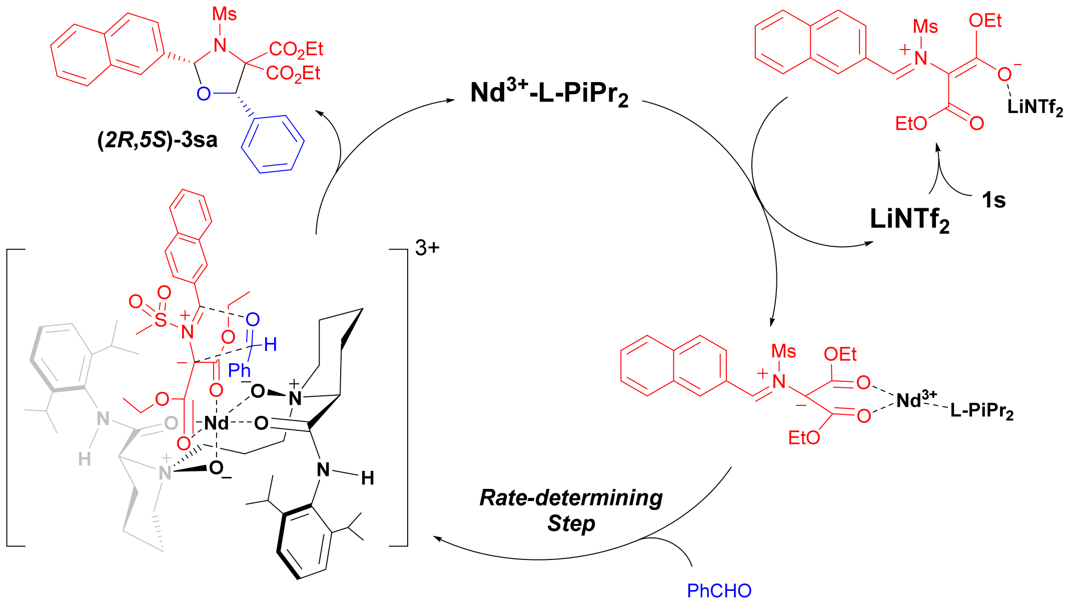

(J) Optimization of conditions[a]

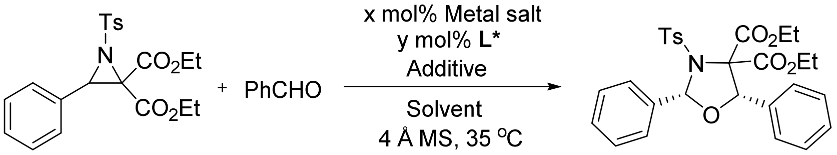

1a **2a** **3aa**

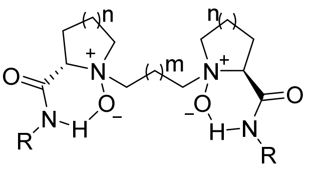

L-PiPr$_2$: R = 2,6-*i*-Pr$_2$C$_6$H$_3$, n = 2, m = 1
L-PrPr$_2$: R = 2,6-*i*-Pr$_2$C$_6$H$_3$, n = 1, m = 1
L$_2$-PiPr$_2$: R = 2,6-*i*-Pr$_2$C$_6$H$_3$, n = 2, m = 0
L-PiPr$_3$: R = 2,4,6-*i*-Pr$_3$C$_6$H$_2$, n = 2, m = 1
L-PiEt$_2$: R = 2,6-Et$_2$C$_6$H$_3$, n = 2, m = 1
L-PiCHPh$_2$: R = CHPh$_2$, n = 2, m = 1

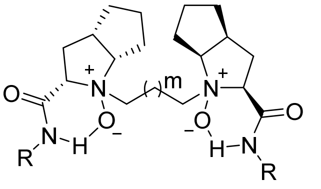

L-RiPr$_2$: R = 2,6-*i*-Pr$_2$C$_6$H$_3$, m = 1

Entry	Ligand	Metal salt	Solvent	Additive	x/y	t (h)	Yield (%)[b]	d.r.[c]	ee (%)[d]
1	**L-PiPr$_2$**	Sc(OTf)$_3$	toluene	-	10/10	12	45	>19:1	0
2	**L-PiPr$_2$**	Ni(ClO$_4$)$_2$·6H$_2$O	toluene	-	10/10	12	20	>19:1	-22
3	**L-PiPr$_2$**	Zn(OTf)$_2$	toluene	-	10/10	12	trace	-	-
4	**L-PiPr$_2$**	La(OTf)$_3$	toluene	-	10/10	12	14	>19:1	36
5	**L-PiPr$_2$**	In(OTf)$_3$	toluene	LiNTf$_2$	10/10	12	<10	> 19:1	-3
6	**L-PiPr$_2$**	La(OTf)$_3$	toluene	LiNTf$_2$	10/10	12	24	> 19:1	58
7	**L-PiPr$_2$**	Hf(OTf)$_3$	toluene	LiNTf$_2$	10/10	12	<10	> 19:1	9
8	**L-PiPr$_2$**	Sm(OTf)$_3$	toluene	LiNTf$_2$	10/10	12	18	> 19:1	50
9	**L-PiPr$_2$**	Eu(OTf)$_3$	toluene	LiNTf$_2$	10/10	12	15	> 19:1	40
10	**L-PiPr$_2$**	Gd(OTf)$_3$	toluene	LiNTf$_2$	10/10	12	22	> 19:1	56
11	**L-PiPr$_2$**	Tb(OTf)$_3$	toluene	LiNTf$_2$	10/10	12	14	> 19:1	55
12	**L-PiPr$_2$**	Ho(OTf)$_3$	toluene	LiNTf$_2$	10/10	12	14	> 19:1	50
13	**L-PiPr$_2$**	Er(OTf)$_3$	toluene	LiNTf$_2$	10/10	12	15	> 19:1	20
14	**L-PiPr$_2$**	Nd(OTf)$_3$	toluene	LiNTf$_2$	10/10	12	30	> 19:1	71
15	**L-PrPr$_2$**	Nd(OTf)$_3$	toluene	LiNTf$_2$	10/10	12	31	> 19:1	40
16	**L-RiPr$_2$**	Nd(OTf)$_3$	toluene	LiNTf$_2$	10/10	12	27	> 19:1	13
17	**C2-L-PiPr$_2$**	Nd(OTf)$_3$	toluene	LiNTf$_2$	10/10	12	45	> 19:1	4
18	**L-PiPr$_3$**	Nd(OTf)$_3$	toluene	LiNTf$_2$	10/10	12	33	> 19:1	56
19	**L-PiEt$_2$**	Nd(OTf)$_3$	toluene	LiNTf$_2$	10/10	12	24	> 19:1	59
20	**L-PiCHPh$_2$**	Nd(OTf)$_3$	toluene	LiNTf$_2$	10/10	12	26	> 19:1	-9

21	**L-PiPr$_2$**	Nd(OTf)$_3$	DCM	LiNTf$_2$	10/10	12	34	> 19:1	75
22	**L-PiPr$_2$**	Nd(OTf)$_3$	DCE	LiNTf$_2$	10/10	12	32	> 19:1	76
23	**L-PiPr$_2$**	Nd(OTf)$_3$	PhCl	LiNTf$_2$	10/10	12	32	> 19:1	74
24	**L-PiPr$_2$**	Nd(OTf)$_3$	TCE	LiNTf$_2$	10/10	12	31	> 19:1	80
25	**L-PiPr$_2$**	Nd(OTf)$_3$	CHCl$_3$	LiNTf$_2$	10/10	12	35	> 19:1	85
26[e]	**L-PiPr$_2$**	Nd(OTf)$_3$	CHCl$_3$	LiNTf$_2$	10/10	12	47	> 19:1	86
27[e]	**L-PiPr$_2$**	Nd(OTf)$_3$	CHCl$_3$	LiNTf$_2$	10/5	12	65	> 19:1	87
28[e]	**L-PiPr$_2$**	Nd(OTf)$_3$	CHCl$_3$	LiCl	10/5	12	55	>19:1	67
29[e]	**L-PiPr$_2$**	Nd(OTf)$_3$	CHCl$_3$	NaBAr$_{F4}$	10/5	12	66	>19:1	14
30[e]	**L-PiPr$_2$**	Nd(OTf)$_3$	CHCl$_3$	NaNTf$_2$	10/5	12	41	>19:1	73
31[e]	**L-PiPr$_2$**	Nd(OTf)$_3$	CHCl$_3$	LiBF$_4$	10/5	12	51	>19:1	63
32[e]	**L-PiPr$_2$**	Nd(OTf)$_3$	CHCl$_3$	LiClO$_4$	10/5	12	53	>19:1	63
33[e]	**L-PiPr$_2$**	Nd(OTf)$_3$	CHCl$_3$	LiBr	10/5	12	32	>19:1	64
34[e,f]	**L-PiPr$_2$**	Nd(OTf)$_3$	CHCl$_3$	LiNTf$_2$	10/5	12	68	>19:1	91
35[e,f]	**L-PiPr$_2$**	Nd(OTf)$_3$	CHCl$_3$	LiNTf$_2$	5/2.5	12	68	>19:1	91

[a] Unless otherwise noted, the reactions were performed with x mol% metal, y mol% ligand, 10 mol% additive, 4 Å MS (20 mg), **1a** (0.1 mmol) and **2a** (0.15 mmol) in solvent (1 mL) under N_2 at 35 ^{o}C for the indicated time. [b] Isolated yield by silica gel chromatography. [c] Determined by ^{1}H NMR spectroscopy and chiral HPLC analysis (Chiralcel IE). [d] Determined by chiral HPLC analysis (Chiralcel IE). [e] x mol% metal, y mol% ligand, 15 mol% additive, 4 Å MS (100 mg), **1a** (0.1 mmol) and **2a** (0.2 mmol) in solvent (0.75 mL). [f] Isolation by basic Al_2O_3 chromatography.

(K) The analytical and spectral characterization data of products

***cis*-Diethyl 2, 5-diphenyl-3-tosyloxazolidine-4, 4′-dicarboxylate (3aa)**

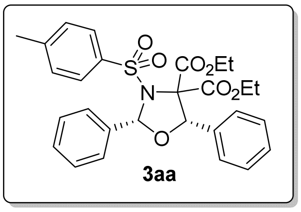

Colorless oil, 68% yield, 91% ee. $[\alpha]_D^{14}$ = +54.9 (c = 0.39 in CH_2Cl_2). (Chiralpak IE, hexane/*i*PrOH = 70/30, flow rate = 1.0 mL/min, λ = 210 nm: t_R (major) = 29.51 min, t_R (minor) = 16.33 min.) ^1H NMR (400 MHz, $CDCl_3$) δ = 7.49 (d, J = 7.2 Hz, 2H), 7.36 - 7.26 (m, 6H), 7.15 (t, J = 8.4 Hz, 4H), 6.90 (d, J = 8.0 Hz, 2H), 6.24 (s, 1H), 5.83 (s, 1H), 4.58 - 4.40 (m, 2H), 3.98 - 3.87 (m, 1H), 3.55 - 3.44 (m, 1H), 2.29 (s, 3H), 1.46 (t, J = 7.2 Hz, 3H), 0.80 (t, J = 7.2 Hz, 3H); ^{13}C NMR (101 MHz, $CDCl_3$) δ = 167.4, 166.3, 142.8, 137.6, 134.6, 134.0, 129.9, 129.8, 129.0, 128.3, 128.1, 127.9, 126.6, 92.9, 87.4, 76.9, 63.1, 62.0, 21.5, 14.0, 13.3. HRMS (ESI-TOF) calcd for $C_{28}H_{29}NO_7SNa$ ($[M+Na^+]$) = 546.1557, Found 546.1554.

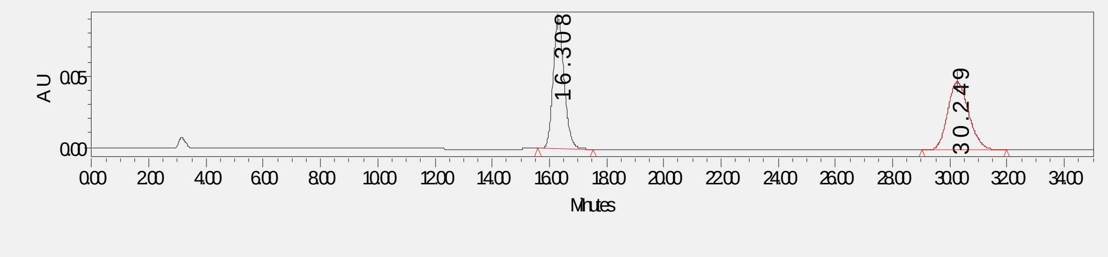

	Retention Time	Area	% Area
1	16.308	2481835	49.95
2	30.249	2486958	50.05

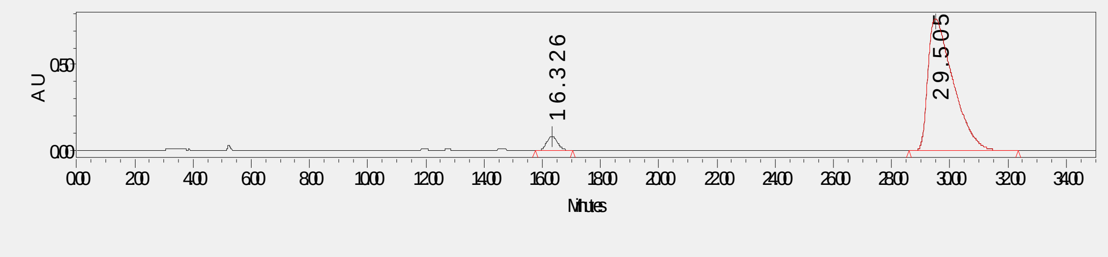

	Retention Time	Area	% Area
1	16.326	2295633	4.67
2	29.505	46816648	95.33

(2*R*,5*S*)-**Dimethyl 2, 5-diphenyl-3-tosyloxazolidine-4, 4′-dicarboxylate (3ba)**

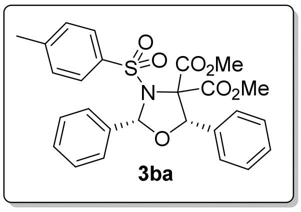

Colorless oil, 62% yield, 90% ee. $[\alpha]_D^{15}$ = +55.3 (c = 0.55 in CH_2Cl_2). (Chiralpak IE, hexane/*i*PrOH = 70/30, flow rate = 1.0 mL/min, λ = 210 nm: t_R (major) = 23.25 min, t_R (minor) = 17.25 min.) ^{1}H NMR (400 MHz, $CDCl_3$) δ = 7.52 - 7.45 (m, 2H), 7.36 - 7.27 (m, 6H), 7.20 - 7.14 (m, 3H), 7.12 (d, J = 8.4 Hz, 2H), 6.91 (d, J = 8.0 Hz, 2H), 6.26 (s, 1H), 5.83 (s, 1H), 4.02 (s, 3H), 3.24 (s, 3H), 2.30 (s, 3H); ^{13}C NMR (101 MHz, DMSO-d_6) δ = 166.8, 165.80, 143.1, 137.2, 134.0, 133.9, 130.0, 129.5, 129.1, 128.6, 128.2, 127.9, 127.3, 126.4, 92.2, 86.3, 76.1, 53.6, 52.2, 20.9. HRMS (ESI-TOF) calcd for $C_{26}H_{25}NNaO_7S^+$ ([M+Na$^+$]) = 518.1244, Found 518.1257.

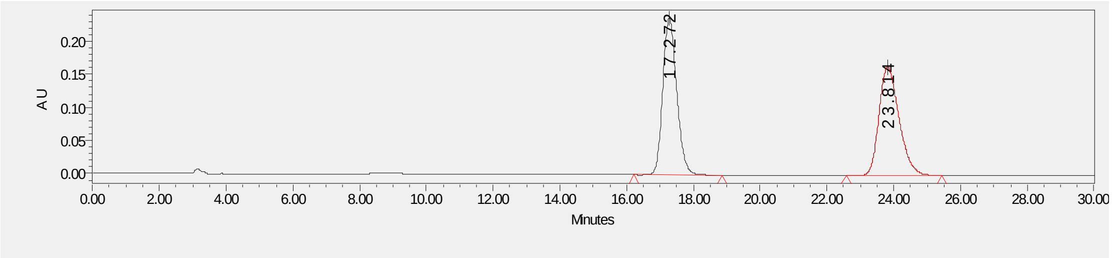

	Retention Time	Area	% Area
1	17.272	6853970	50.05
2	23.814	6839062	49.95

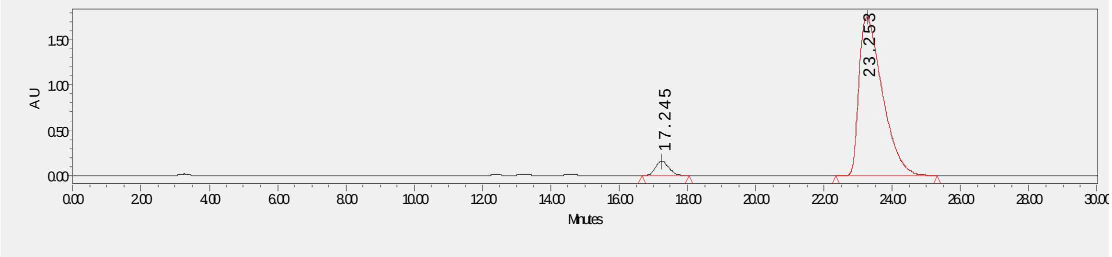

	Retention Time	Area	% Area
1	17.245	4475915	5.09
2	23.253	83399621	94.91

(*2R*,*5S*)-**Diisopropyl 2, 5-diphenyl-3-tosyloxazolidine-4, 4′-dicarboxylate (3ca)**

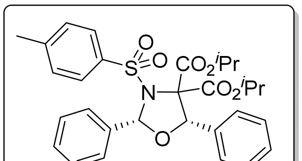

3ca

Colorless oil, 40% yield, 72% ee. $[\alpha]_{\lambda}^{27}$ = +181.5 (*c* = 0.54 in CH_2Cl_2, λ = 365 nm). (Chiralpak IE, hexane/*i*PrOH = 70/30, flow rate = 1.0 mL/min, λ = 210 nm: t_R (major) = 23.25 min, t_R (minor) = 17.25 min.) ^{1}H NMR (400 MHz, $CDCl_3$) δ = 7.47 (d, *J* = 7.6 Hz, 2H), 7.37 - 7.27 (m, 6H), 7.19 - 7.11 (m, 4H), 6.90 (d, *J* = 8.4 Hz, 2H), 6.19 (s, 1H), 5.82 (s, 1H), 5.41 - 5.31 (m, 1H), 4.68 - 4.58 (m, 1H), 2.29 (s, 3H), 1.49 - 1.42 (m, 6H), 1.08 (d, *J* = 6.0 Hz, 3H), 0.60 (d, *J* = 6.4 Hz, 3H); ^{13}C NMR (101 MHz, $CDCl_3$) δ = 167.0, 165.9, 142.7, 137.8, 134.8, 134.1, 129.9, 129.8, 128.8, 128.3, 128.2, 127.9, 126.7, 92.7, 87.3, 76.8, 71.1, 70.2, 21.7, 21.4, 21.3, 20.7. HRMS (ESI-TOF) calcd for $C_{30}H_{34}NO_7S^+$ ($[M+H^+]$) = 552.2050, Found 552.2051.

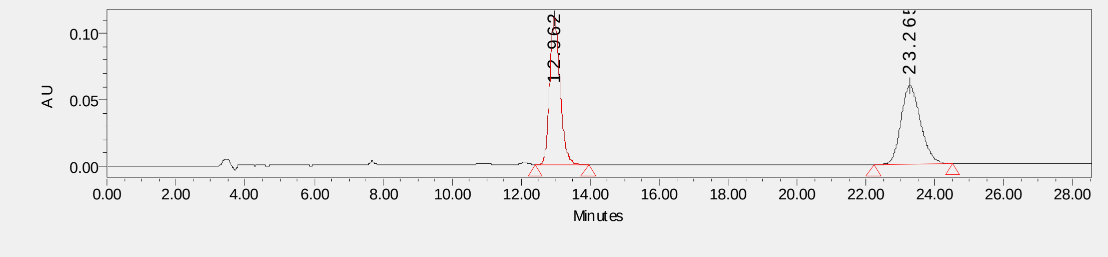

	Retention Time	Area	% Area
1	12.962	2333269	50.08
2	23.265	2325674	49.92

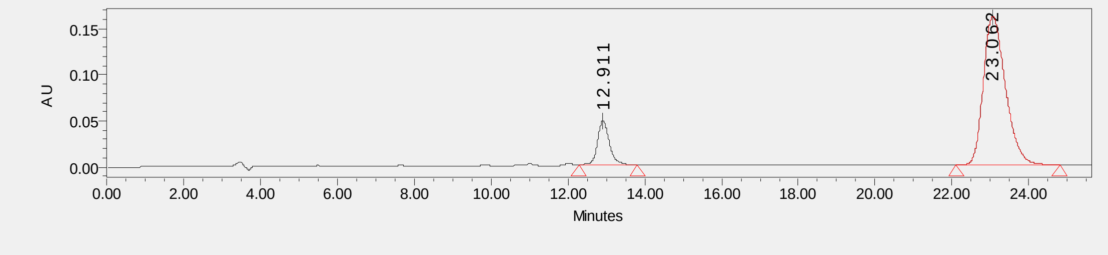

	Retention Time	Area	% Area
1	12.911	1047197	13.96
2	23.062	6455169	86.04

(*2R,5S*)-**Diethyl 2, 5-diphenyl-3-(4-chlorobenzenesulfonyl)oxazolidine-4, 4′-dicarboxylate (3da)**

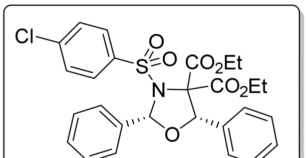

3da

Colorless oil, 74% yield, 89% ee. $[\alpha]_D^{14}$ = +59.6 (*c* = 0.70 in CH_2Cl_2). (Chiralpak IE, hexane/*i*PrOH = 80/20, flow rate = 1.0 mL/min, λ = 210 nm: t_R (major) = 18.54 min, t_R (minor) = 12.71 min.) ^{1}H NMR (400 MHz, $CDCl_3$) δ = 7.46 (d, *J* = 7.2 Hz, 2H), 7.37 - 7.29 (m, 6H), 7.23 - 7.14 (m, 4H), 7.09 - 7.03 (m, 2H), 6.22 (s, 1H), 5.82 (s, 1H), 4.58 - 4.41 (m, 2H), 3.99 - 3.89 (m, 1H), 3.55 - 3.45 (m, 1H), 1.46 (t, *J* = 7.2 Hz, 3H), 0.80 (t, J = 7.2 Hz, 3H); ^{13}C NMR (101 MHz, $CDCl_3$) δ = 167.2, 166.3, 139.0, 138.6, 134.4, 133.6, 130.2, 129.9, 129.6, 129.1, 128.3, 128.1, 127.9, 126.5, 92.8, 87.5, 77.2, 63.2, 62.2, 14.0, 13.3. HRMS (ESI-TOF) calcd for $C_{27}H_{26}NO_7S^{34.9689}ClNa$ ([M+Na$^+$]) = 566.1011, Found 566.1013. HRMS (ESI-TOF) calcd for $C_{27}H_{26}NO_7S^{36.9659}ClNa$ ([M+Na$^+$]) = 568.0982, Found 568.1010

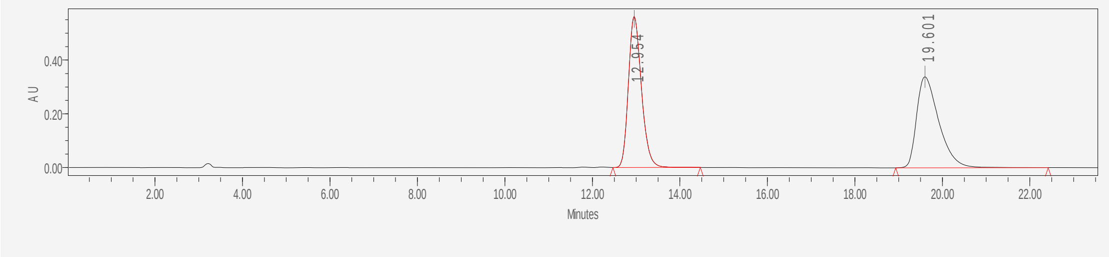

	Retention Time	Area	% Area
1	12.954	11628471	49.41
2	19.601	11904976	50.59

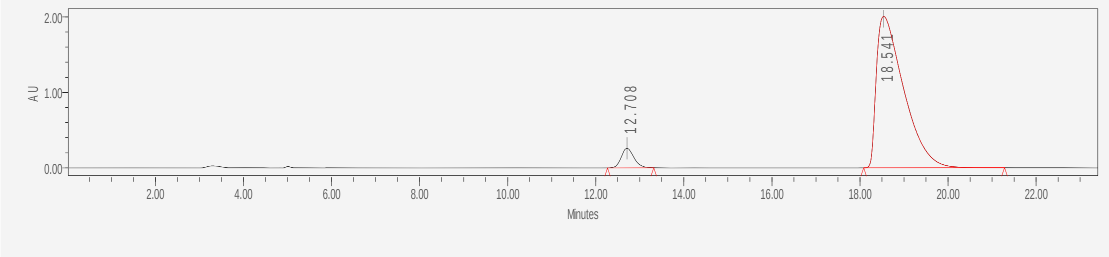

	Retention Time	Area	% Area
1	12.708	5133168	5.61
2	18.541	86387984	94.39

***cis*-Diethyl 2, 5-diphenyl-3-benzenesulfonyloxazolidine-4, 4′-dicarboxylate (3ea)**

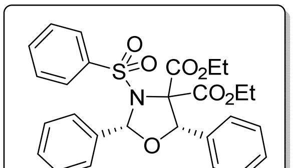

3ea

Colorless oil, 70% yield, 90% ee. $[\alpha]_D^{14}$ = +49.5 (c = 0.59 in CH_2Cl_2). (Chiralpak IE, hexane/*i*PrOH = 80/20, flow rate = 1.0 mL/min, λ = 210 nm: t_R (major) = 36.03 min, t_R (minor) = 20.06 min.) ^{1}H NMR (400 MHz, $CDCl_3$) δ = 7.48 (d, J = 7.6 Hz, 2H), 7.37 - 7.25 (m, 9H), 7.17 - 7.08 (m, 4H), 6.25 (s, 1H), 5.84 (s, 1H), 4.59 - 4.41 (m, 2H), 3.98 - 3.88 (m, 1H), 3.56 - 3.45 (m, 1H), 1.47 (t, J = 7.2 Hz, 3H), 0.81 (t, J = 7.2 Hz, 3H); ^{13}C NMR (101 MHz, $CDCl_3$) δ = 167.3, 166.2, 140.5, 134.5, 133.8, 132.1, 130.1, 129.8, 129.0, 128.2, 128.1, 128.0, 127.7, 126.6, 92.9, 87.4, 77.0, 63.1, 62.0, 14.0, 13.3. HRMS (ESI-TOF) calcd for $C_{27}H_{27}NO_7SNa^+$ ([M+Na$^+$]) = 532.1401, Found 532.1393.

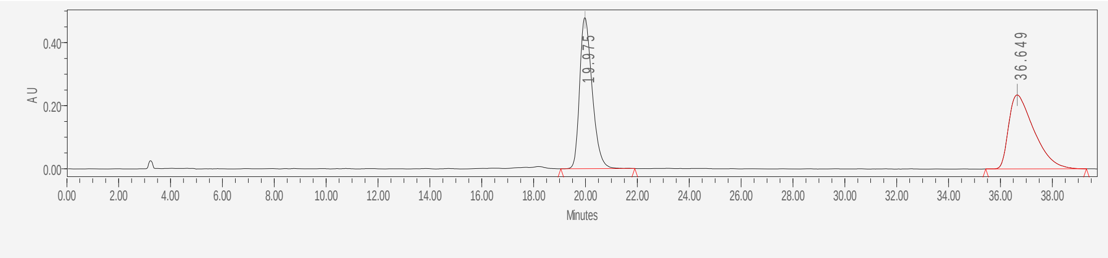

	Retention Time	Area	% Area
1	19.975	16085262	50.38
2	36.649	15840644	49.62

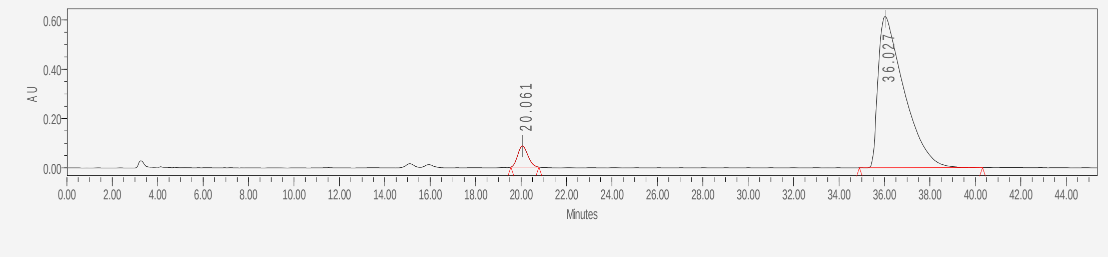

	Retention Time	Area	% Area
1	20.061	2679807	5.24
2	36.027	48483432	94.76

***cis*-Diethyl 2, 5-diphenyl-3-(4-methoxylbenzenesulfonyl)oxazolidine-4, 4′-dicarboxylate (3fa)**

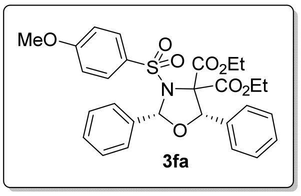

Colorless oil, 60% yield, 90% ee. $[\alpha]_{\lambda}^{26}$ = +216.1 (c = 0.58 in CH_2Cl_2, λ = 365 nm). (Chiralpak IE, hexane/*i*PrOH = 70/30, flow rate = 1.0 mL/min, λ = 210 nm: t_R (major) = 34.17 min, t_R (minor) = 20.58 min.) ^{1}H NMR (400 MHz, DMSO-d_6) δ = 7.48 (d, J = 7.2 Hz, 2H), 7.37 - 7.27 (m, 6H), 7.25 - 7.14 (m, 4H), 6.57 (d, J = 9.2 Hz, 2H), 6.22 (s, 1H), 5.82 (s, 1H), 4.59 - 4.40 (m, 2H), 3.98 - 3.88 (m, 1H), 3.77 (s, 3H), 3.55 - 3.45 (m, 1H), 1.46 (t, J = 7.2 Hz, 3H), 0.81 (t, J = 7.2 Hz, 3H); ^{13}C NMR (101 MHz, DMSO) δ = 166.3, 165.4, 162.2, 134.3, 133.9, 131.8, 130.0, 129.6, 129.4, 128.9, 128.1, 127.8, 126.4, 113.3, 92.0, 86.3, 76.0, 62.4, 61.5, 55.6, 13.7, 13.0. HRMS (ESI-TOF) calcd for $C_{28}H_{29}NNaO_8S^+$ ([M+Na$^+$]) = 562.1506, Found 562.1508.

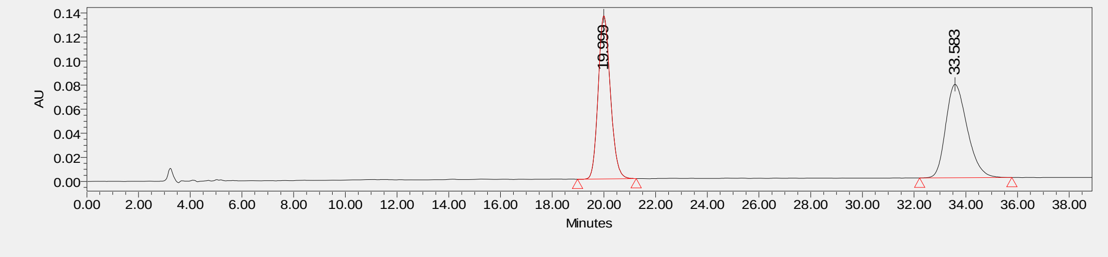

	Retention Time	Area	% Area
1	19.999	4446598	49.73
2	33.583	4494113	50.27

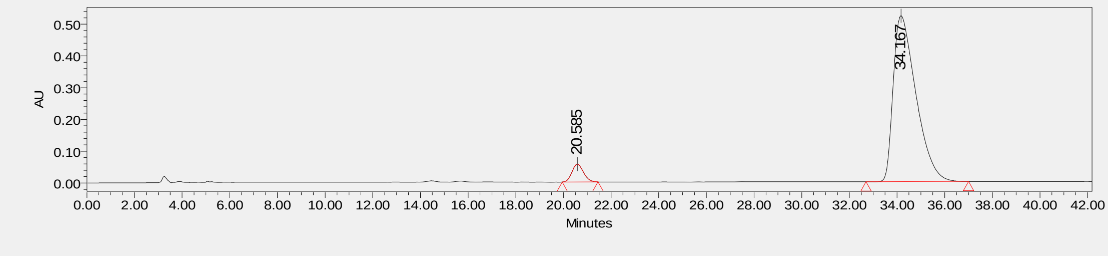

	Retention Time	Area	% Area
1	20.585	1897845	5.19
2	34.167	34638368	94.81

***cis*-Diethyl 2, 5-diphenyl-3-(2-methylbenzenesulfonyl)oxazolidine-4, 4′-dicarboxylate (3ga)**

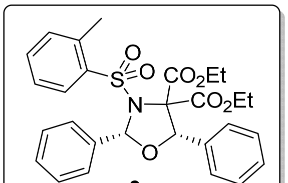

3ga

Colorless oil, 54% yield, 76% ee. (4% recovered aziridine) (Chiralpak IE, hexane/*i*PrOH = 70/30, flow rate = 1.0 mL/min, λ = 210 nm: t_R (major) = 25.51 min, t_R (minor) = 13.24 min.) ^{1}H NMR (400 MHz, $CDCl_3$) δ = 7.60 (d, *J* = 7.2 Hz, 2H), 7.41 (d, *J* = 8.0 Hz, 1H), 7.38 - 7.29 (m, 5H), 7.29 - 7.23 (m, 2H), 7.19 (dd, *J* = 7.6, 15.2 Hz, 3H), 6.98 (d, *J* = 7.6 Hz, 1H), 6.91 (t, *J* = 8.0 Hz, 1H), 6.34 (s, 1H), 5.97 (s, 1H), 4.52 - 4.29 (m, 2H), 4.00 - 3.85 (m, 1H), 3.55 - 3.39 (m, 1H), 2.33 (s, 3H), 1.36 (t, *J* = 7.2 Hz, 3H), 0.79 (t, *J* = 7.2 Hz, 3H); ^{13}C NMR (101 MHz, $CDCl_3$) δ = 166.5, 166.4, 138.8, 138.0, 134.6, 134.4, 132.3, 131.9, 130.2, 129.9, 129.7, 128.9, 128.2, 127.9, 126.5, 125.4, 93.7, 87.6, 76.7, 63.0, 61.9, 20.9, 13.8, 13.3. HRMS (ESI-TOF) calcd for $C_{28}H_{29}NNaO_7S^+$ ([M+Na$^+$]) = 546.1557, Found 546.1555.

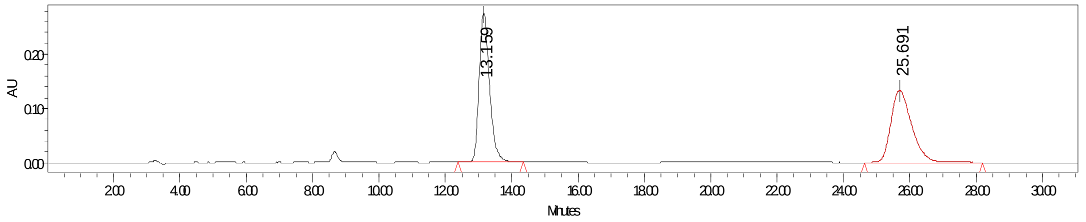

	Retention Time	Area	% Area
1	13.159	5987903	50.96
2	25.691	5763400	49.04

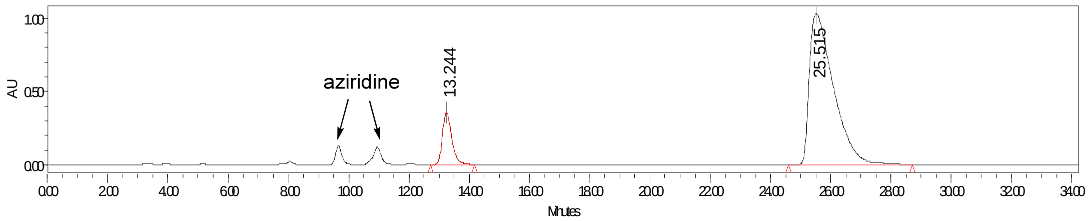

	Retention Time	Area	% Area
1	13.244	7767003	11.96
2	25.515	57154166	88.04

(*2R,5S*)-**Diethyl 2, 5-diphenyl-3-methylsulfonyloxazolidine-4, 4′-dicarboxylate (3ia)**

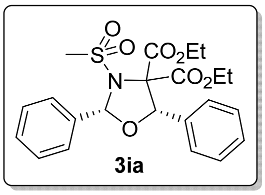

Colorless oil, 77% yield, 95% ee. $[\alpha]_{\lambda}^{31}$ = +58.9 (*c* = 0.43 in CH_2Cl_2, λ = 365 nm). (Chiralpak IE, hexane/*i*PrOH = 70/30, flow rate = 1.0 mL/min, λ = 210 nm: t_R (major) = 17.73 min, t_R (minor) = 13.21 min.) ^{1}H NMR (400 MHz, $CDCl_3$) δ = 7.82 - 7.75 (m, 2H), 7.52 - 7.46 (m, 3H), 7.37 (s, 5H), 6.25 (s, 1H), 5.82 (s, 1H), 4.50 - 4.32 (m, 2H), 4.02 - 3.90 (m, 1H), 3.63 - 3.52 (m, 1H), 2.48 (s, 3H), 1.39 (t, *J* = 7.2 Hz, 3H), 0.79 (t, *J* = 7.2 Hz, 3H); ^{13}C NMR (101 MHz, $CDCl_3$) δ = 167.1, 166.9, 134.6, 134.5, 130.6, 129.7, 129.1, 128.6, 128.3, 126.4, 92.3, 87.6, 77.0, 63.2, 62.1, 43.0, 14.0, 13.2. HRMS (ESI-TOF) calcd for $C_{22}H_{25}NNaO_7S^+$ ($[M+Na^+]$) = 470.1244, Found 470.1255.

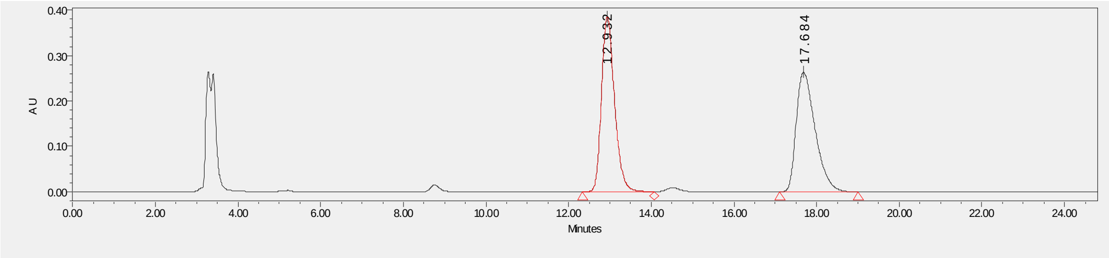

	Retention Time	Area	% Area
1	12.932	8488676	50.20
2	17.684	8422281	49.80

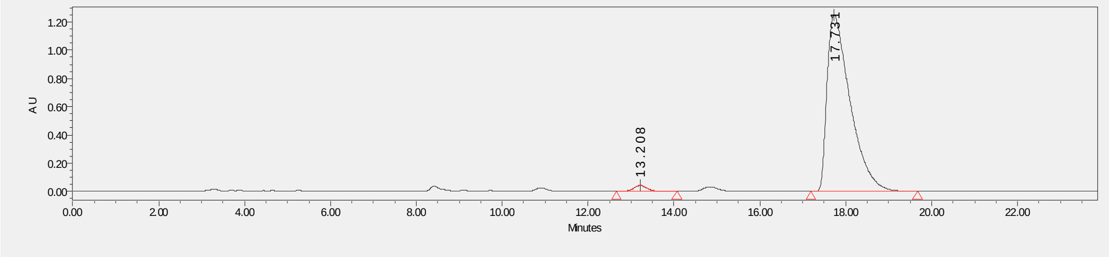

	Retention Time	Area	% Area
1	13.208	858882	1.80
2	17.731	46729578	98.20

(*2R,5S*)-**Diethyl 2, 5-diphenyl-3-(2-trimethylsilylethanesulfonyl)oxazolidine-4, 4′-dicarboxylate (3ja)**

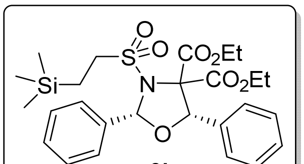

White solid, m.p. 108-109 °C, 66% yield, 93% ee. $[\alpha]_{\lambda}^{27}$ = +65.3 (c = 1.32 in CH_2Cl_2, λ = 365 nm). (Chiralpak IE, hexane/*i*PrOH = 70/30, flow rate = 1.0 mL/min, λ = 210 nm: t_R (major) = 32.19 min, t_R (minor) = 11.22 min.) ^{1}H NMR (400 MHz, $CDCl_3$) δ = 7.76 - 7.67 (m, 2H), 7.44 - 7.36 (m, 3H), 7.34 - 7.25 (m, 5H), 6.18 (s, 1H), 5.76 (s, 1H), 4.48 - 4.22 (m, 2H), 3.93 - 3.80 (m, 1H), 3.53 - 3.40 (m, 1H), 2.95 - 2.82 (m, 1H), 1.88 - 1.75 (m, 1H), 1.33 (t, J = 7.2 Hz, 3H), 0.80- 0.69 (m, 4H), 0.65 - 0.55 (m, 1H), -0.27 (s, 9H); ^{13}C NMR (101 MHz, $CDCl_3$) δ = 167.2, 166.8, 135.2, 134.6, 130.5, 129.6, 128.9, 128.4, 128.2, 126.4, 92.2, 87.6, 76.8, 63.0, 61.9, 51.8, 13.9, 13.2, 8.8, -2.3. HRMS (ESI-TOF) calcd for $C_{26}H_{35}NNaO_7SSi^+$ ([M+Na^+]) = 556.1796, Found 556.1806.

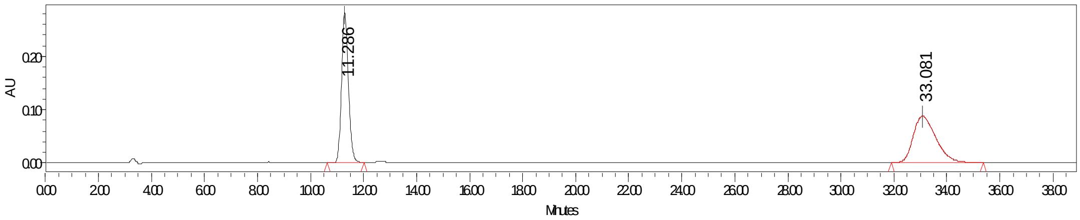

	Retention Time	Area	% Area
1	11.286	5149747	49.99
2	33.081	5150946	50.01

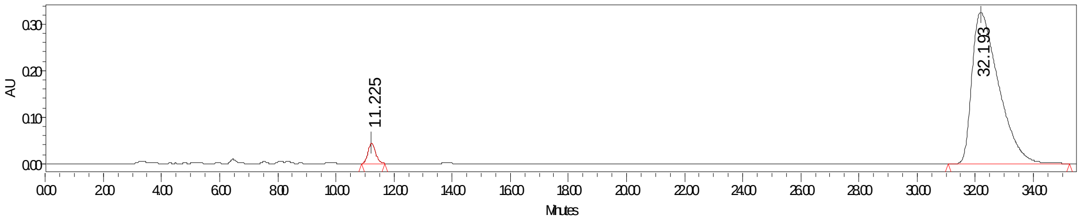

	Retention Time	Area	% Area
1	11.225	778015	3.63
2	32.193	20653020	96.37

(2*R*,5*S*)-**Diethyl 2-(4-chlorophenyl)-5-phenyl-3-methylsulfonyloxazolidine-4, 4′-dicarboxylate (3ka)**

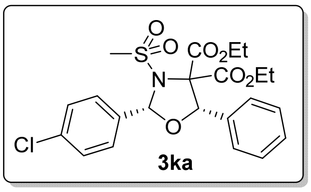

Colorless oil, 80% yield, 94% ee. $[\alpha]_{\lambda}^{31}$ = +19.8 (*c* = 0.51 in CH_2Cl_2, λ = 365 nm). (Chiralpak IE, hexane/*i*PrOH = 70/30, flow rate = 1.0 mL/min, λ = 210 nm: t_R (major) = 17.67 min, t_R (minor) = 12.23 min.) ^{1}H NMR (400 MHz, $CDCl_3$) δ = 7.74 (d, *J* = 8.4 Hz, 2H), 7.47 (d, *J* = 8.0 Hz, 2H), 7.41 - 7.30 (m, 5H), 6.23 (s, 1H), 5.82 (s, 1H), 4.49 - 4.30 (m, 2H), 4.01 - 3.90 (m, 1H), 3.61 - 3.49 (m, 1H), 2.56 (s, 3H), 1.38 (t, *J* = 7.2 Hz, 3H), 0.78 (t, *J* = 7.2 Hz, 3H); ^{13}C NMR (101 MHz, $CDCl_3$) δ = 166.9, 166.9, 136.6, 134.3, 133.4, 131.0, 129.2, 128.9, 128.4, 126.3, 91.6, 87.7, 76.8, 63.2, 62.2, 43.2, 13.9, 13.2. HRMS (ESI-TOF) calcd for $C_{22}H_{24}NO_7S^{34.9689}ClNa$ ([M+Na$^+$]) = 504.0855, Found 504.0862. HRMS (ESI-TOF) calcd for $C_{22}H_{24}NO_7S^{36.9659}ClNa$ ([M+Na$^+$]) = 506.0825, Found 506.0844.

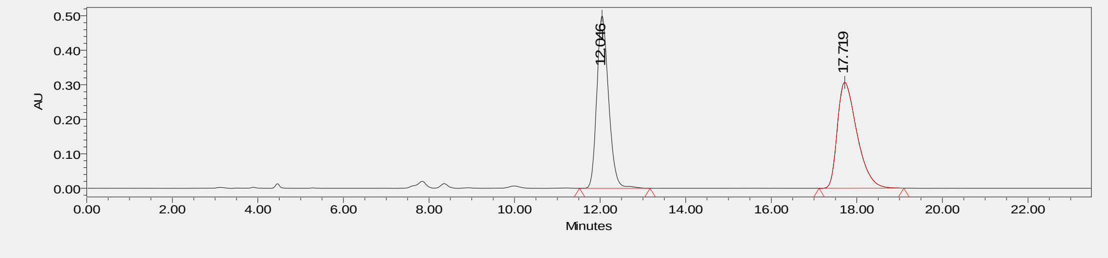

	Retention Time	Area	% Area
1	12.046	9489188	49.50
2	17.719	9681523	50.50

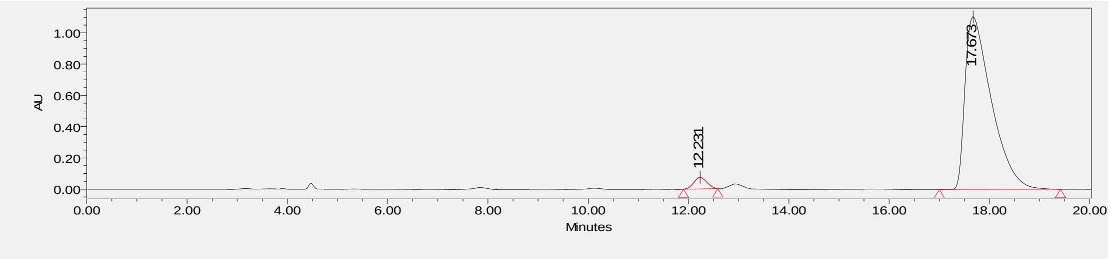

	Retention Time	Area	% Area
1	12.231	1346321	3.24
2	17.673	40206392	96.76

(*2R,5S*)-**Diethyl 2-(3-chlorophenyl)-5-phenyl-3-methylsulfonyloxazolidine-4, 4′-dicarboxylate (3la)**

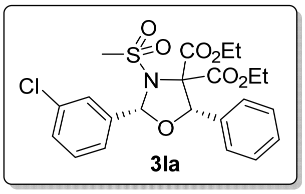

Colorless oil, 78% yield, 92% ee. $[\alpha]_{\lambda}^{25}$ = +34.6 (*c* = 0.48 in CH_2Cl_2, λ = 365 nm). (Chiralpak IE, hexane/*i*PrOH = 70/30, flow rate = 1.0 mL/min, λ = 210 nm: t_R (major) = 21.0 min, t_R (minor) = 11.5 min.) ^{1}H NMR (400 MHz, $CDCl_3$) δ = 7.83 - 7.77 (m, 1H), 7.71 - 7.65 (m, 1H), 7.50 - 7.30 (m, 7H), 6.21 (s, 1H), 5.82 (s, 1H), 4.48 - 4.31 (m, 2H), 4.03 - 3.92 (m, 1H), 3.65 - 3.53 (m, 1H), 2.59 (s, 3H), 1.38 (t, *J* = 7.2 Hz, 3H), 0.80 (t, *J* = 7.2 Hz, 3H); ^{13}C NMR (101 MHz, $CDCl_3$) δ = 166.9, 166.8, 136.9, 134.5, 134.2, 130.8, 129.9, 129.6, 129.2, 128.4, 127.9, 126.4, 91.6, 87.8, 76.8, 63.3, 62.2, 43.2, 13.9, 13.2. HRMS (ESI-TOF) calcd for $C_{22}H_{24}NO_7S^{34.9689}ClNa$ ([M+Na$^+$]) = 504.0855, Found 504.0864. HRMS (ESI-TOF) calcd for $C_{22}H_{24}NO_7S^{36.9659}ClNa$ ([M+Na$^+$]) = 506.0825, Found 506.0846.

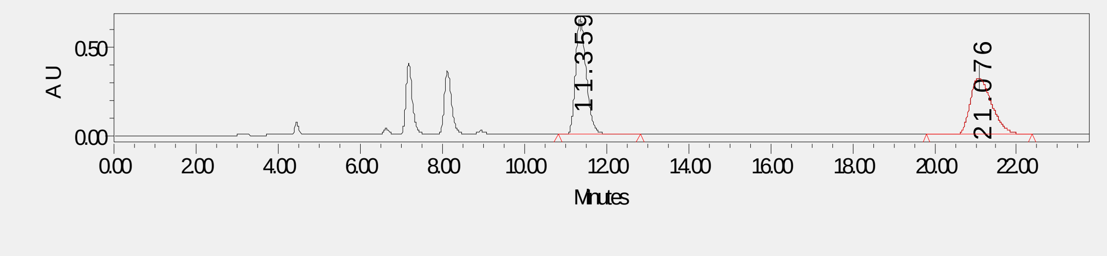

	Retention Time	Area	% Area
1	11.359	12113629	50.24
2	21.076	11995954	49.76

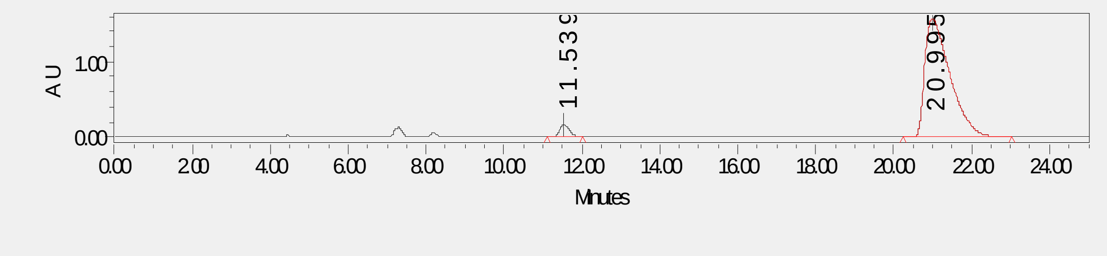

	Retention Time	Area	% Area
1	11.539	2842464	3.87
2	20.995	70607097	96.13

(*2R*,*5S*)-**Diethyl 2-(2-chlorophenyl)-5-phenyl-3-methylsulfonyloxazolidine-4, 4′-dicarboxylate (3ma)**

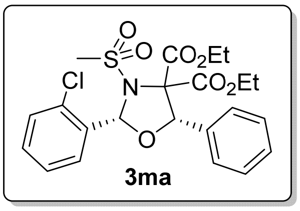

Colorless oil, 71% yield, 88% ee. $[\alpha]_{\lambda}^{26}$ = +43.4 (*c* = 0.58 in CH_2Cl_2, λ = 365 nm). (Chiralpak ID, hexane/*i*PrOH = 70/30, flow rate = 1.0 mL/min, λ = 210 nm: t_R (major) = 27.6 min, t_R (minor) = 12.0 min.) ^{1}H NMR (400 MHz, DMSO-d$_6$) δ = 8.11 (d, *J* = 7.2 Hz, 1H), 7.63 - 7.51 (m, 3H), 7.46 - 7.37 (m, 3H), 7.37 - 7.24 (m, 2H), 6.68 (s, 1H), 5.89 (s, 1H), 4.46 - 4.17 (m, 2H), 3.94 - 3.82 (m, 1H), 3.60 - 3.50 (m, 1H), 2.80 (s, 3H), 1.30 (t, *J* = 7.2 Hz, 3H), 0.76 (t, *J* = 7.2 Hz, 3H); ^{13}C NMR (101 MHz, DMSO-d$_6$) δ = 166.1, 166.0, 134.2, 133.9, 132.4, 131.9, 130.9, 129.7, 129.0, 128.2, 127.6, 126.6, 87.7, 86.6, 75.9, 62.6, 61.7, 42.8, 13.6, 13.0. HRMS (ESI-TOF) calcd for $C_{22}H_{24}NO_7S^{34.9689}ClNa$ ([M+Na$^+$]) = 504.0855, Found 504.0851. HRMS (ESI-TOF) calcd for $C_{22}H_{24}NO_7S^{36.9659}ClNa$ ([M+Na$^+$]) = 506.0825, Found 506.0842.

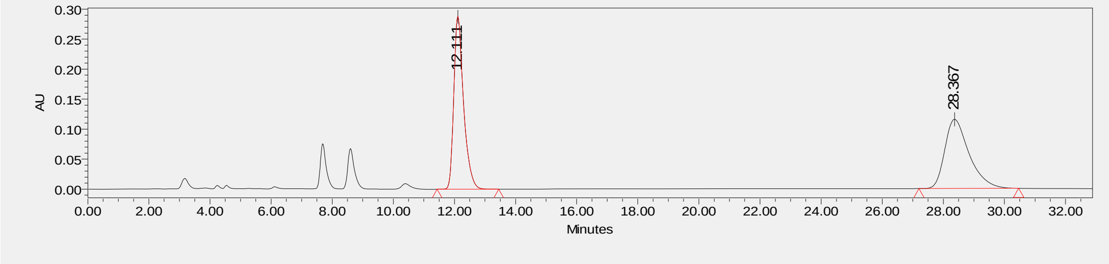

	Retention Time	Area	% Area
1	12.111	6375058	49.86
2	28.367	6410644	50.14

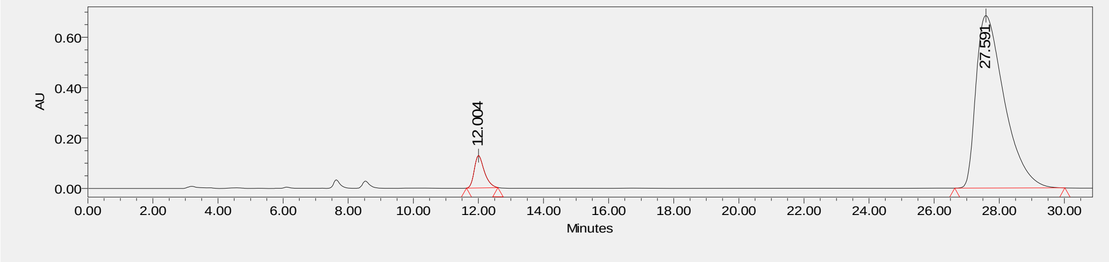

	Retention Time	Area	% Area
1	12.004	2746877	6.21
2	27.591	41471338	93.79

(*2R*,*5S*)-Diethyl 2-(4-bromophenyl)-5-phenyl-3-methylsulfonyloxazolidine-4, 4′-dicarboxylate (3na)

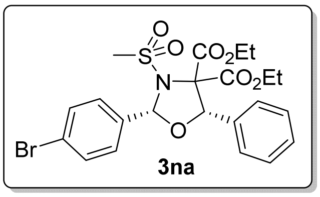

Colorless oil, 70% yield, 93% ee. $[\alpha]_{\lambda}^{31}$ = +18.8 (c = 0.55 in CH_2Cl_2, λ = 365 nm). (Chiralpak IE, hexane/*i*PrOH = 70/30, flow rate = 1.0 mL/min, λ = 210 nm: t_R (major) = 19.97 min, t_R (minor) = 12.91 min.) ^{1}H NMR (400 MHz, $CDCl_3$) δ = 7.65 (dd, J = 8.8 Hz, 20.0 Hz, 4H), 7.43 - 7.30 (m, 5H), 6.21 (s, 1H), 5.82 (s, 1H), 4.48 - 4.30 (m, 2H), 4.01 - 3.90 (m, 1H), 3.61 - 3.49 (m, 1H), 2.57 (s, 3H), 1.38 (t, J = 7.2 Hz, 3H), 0.78 (t, J = 7.2 Hz, 3H); ^{13}C NMR (101 MHz, $CDCl_3$) δ = 166.9, 166.8, 134.3, 133.9, 131.8, 131.3, 129.2, 128.4, 126.4, 124.9, 91.7, 87.7, 76.8, 63.2, 62.2, 43.3, 13.9, 13.2. HRMS (ESI-TOF) calcd for $C_{22}H_{24}{}^{78.9183}BrNNaO_7S^+$ ([M+Na$^+$]) = 548.0350, Found 548.0352. HRMS (ESI-TOF) calcd for $C_{22}H_{24}{}^{80.9163}BrNNaO_7S^+$ ([M+Na$^+$]) = 550.0329, Found 550.0331.

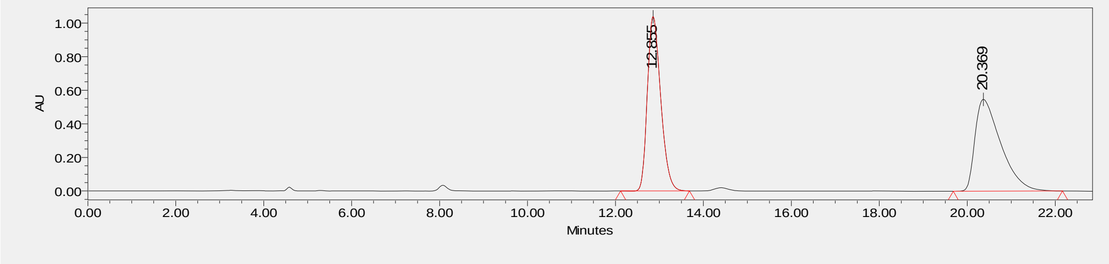

	Retention Time	Area	% Area
1	12.855	21959895	49.65
2	20.369	22270136	50.35

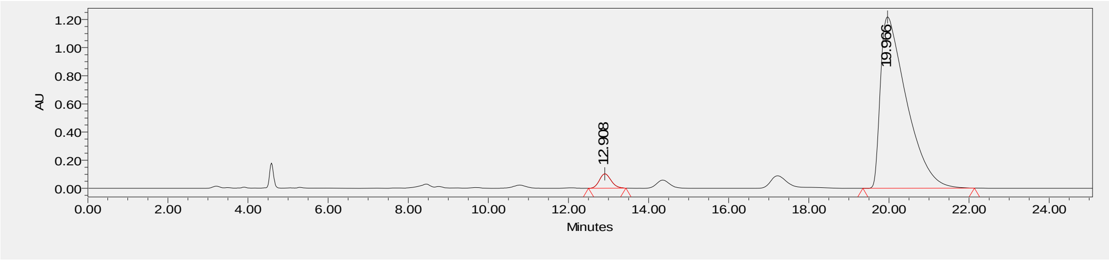

	Retention Time	Area	% Area
1	12.908	2090620	3.64
2	19.966	55326464	96.36

(*2R,5S*)-Diethyl 2-(4-fluorophenyl)-5-phenyl-3-methylsulfonyloxazolidine-4, 4′-dicarboxylate (3oa)

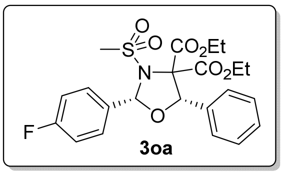

3oa

Colorless oil, 66% yield, 94% ee. $[\alpha]_{\lambda}^{22}$ = +39.2 (*c* = 0.56 in CH_2Cl_2, λ = 365 nm). (Chiralpak IE, hexane/*i*PrOH = 80/20, flow rate = 1.0 mL/min, λ = 210 nm: t_R (major) = 19.17 min, t_R (minor) = 15.52 min.) ^{1}H NMR (400 MHz, $CDCl_3$) δ = 7.79 (dd, *J* = 8.4, 5.2 Hz, 2H), 7.42 - 7.31 (m, 5H), 7.18 (t, *J* = 8.4 Hz, 2H), 6.24 (s, 1H), 5.82 (s, 1H), 4.50 - 4.30 (m, 2H), 4.02 - 3.89 (m, 1H), 3.60 - 3.50 (m, 1H), 2.53 (s, 3H), 1.39 (t, *J* = 7.2 Hz, 3H), 0.78 (t, *J* = 7.2 Hz, 3H); ^{13}C NMR (101 MHz, $CDCl_3$) δ = 167.0, 166.9, 164.0 (d, *J* = 251.1 Hz), 134.3, 131.6 (d, *J* = 8.7 Hz), 130.7 (d, *J* = 3.2 Hz), 129.1, 128.4, 126.3, 115.7 (*J* = 21.9 Hz), 91.6, 87.6, 76.9, 63.2, 62.2, 43.1, 13.9, 13.2; ^{19}F NMR (376 MHz, $CDCl_3$) δ = -109.8. HRMS (ESI-TOF) calcd for $C_{22}H_{24}FNNaO_7S^+$ ([M+Na$^+$]) = 488.1150, Found 488.1156.

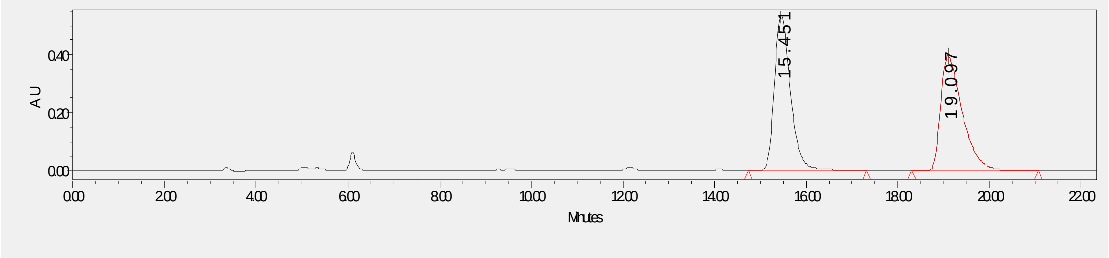

	Retention Time	Area	% Area
1	15.451	13202966	50.32
2	19.097	13033592	49.68

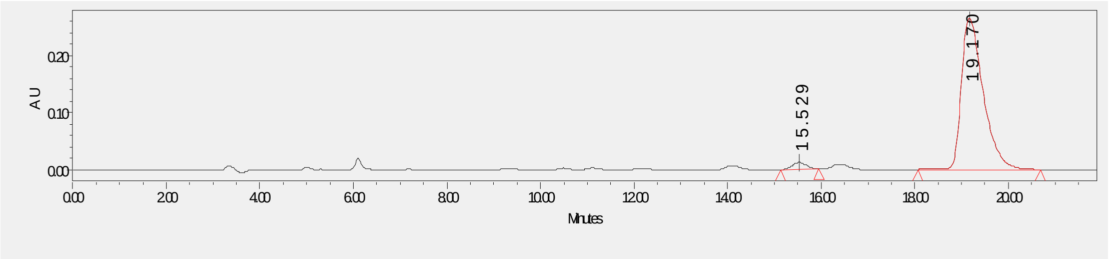

	Retention Time	Area	% Area
1	15.529	262149	2.97
2	19.170	8567033	97.03

(*2R,5S*)-**Diethyl 2-(2-trifluoromethylphenyl)-5-phenyl-3-methylsulfonyloxazoidine-4, 4′-dicarboxylate (3pa)**

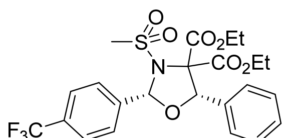

3pa

Colorless oil, 98% yield, 91% ee. $[\alpha]_{\lambda}^{25}$ = +32.2 (*c* = 0.85 in CH_2Cl_2, λ = 365 nm). (Chiralpak IE, hexane/*i*PrOH = 80/20, flow rate = 1.0 mL/min, λ = 210 nm: t_R (major) = 12.30 min, t_R (minor) = 11.48 min.) ^{1}H NMR (400 MHz, $CDCl_3$) δ = 7.94 (d, *J* = 8.0 Hz, 2H), 7.76 (d, *J* = 8.0 Hz, 2H), 7.40 - 7.32 (m, 5H), 6.31 (s, 1H), 5.87 (s, 1H), 4.51 - 4.27 (m, 2H), 4.02 - 3.91 (m, 1H), 3.61 - 3.50 (m, 1H), 2.57 (s, 3H), 1.38 (t, *J* = 7.2 Hz, 3H), 0.78 (t, *J* = 7.2 Hz, 3H); ^{13}C NMR (101 MHz, $CDCl_3$) δ = 166.9, 166.7, 139.0, 134.2, 132.5 (q, *J* = 32.7 Hz), 130.1, 129.2, 128.4, 126.3, 125.5 (q, *J* = 3.7 Hz), 123.8 (d, *J* = 273.5 Hz), 91.5, 87.9, 76.8, 63.3, 62.3, 43.3, 13.9, 13.2; ^{19}F NMR (376 MHz, $CDCl_3$) δ = -62.8. HRMS (ESI-TOF) calcd for $C_{23}H_{24}F_3NNaO_7S^+$ ([M+Na$^+$]) = 538.1118, Found 538.1121.

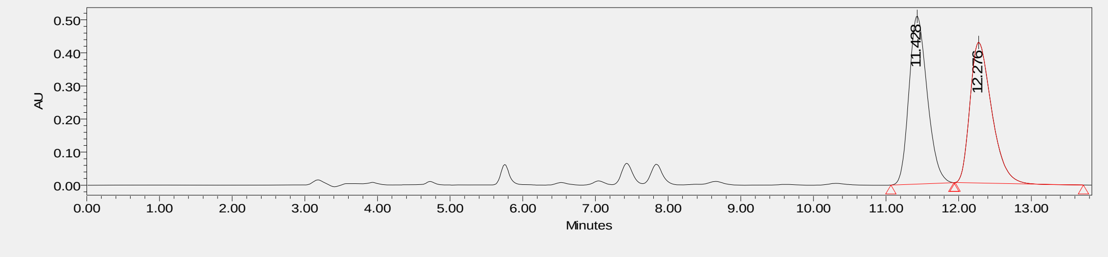

	Retention Time	Area	% Area
1	11.428	8849821	50.21
2	12.276	8774340	49.79

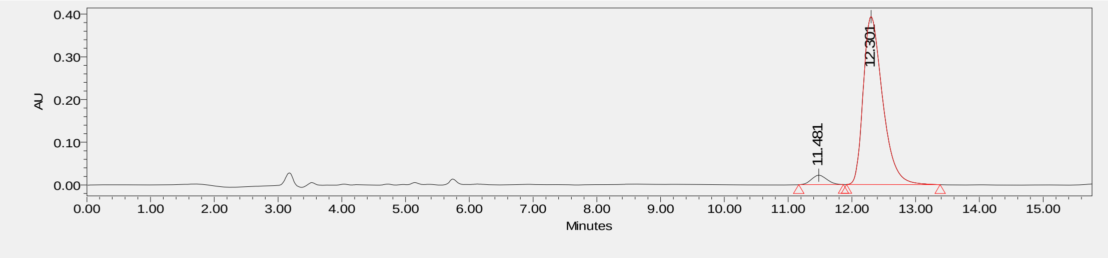

	Retention Time	Area	% Area
1	11.481	371533	4.28
2	12.301	8308964	95.72

***cis*-Diethyl 2-(4-nitrophenyl)-5-phenyl-3-methylsulfonyloxazolidine-4, 4′-dicarboxylate (3qa)**

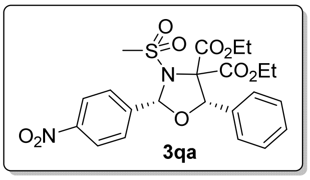

Colorless oil, 84% yield, 87% ee. $[\alpha]_{\lambda}^{23}$ = -13.6 (c = 0.74 in CH_2Cl_2, λ = 405 nm). (Chiralpak IE, hexane/*i*PrOH = 70/30, flow rate = 1.0 mL/min, λ = 210 nm: t_R (major) = 32.68 min, t_R (minor) = 17.55 min.) ^{1}H NMR (400 MHz, $CDCl_3$) δ = 8.35 (d, J = 8.8 Hz, 2H), 8.01 (d, J = 8.8 Hz, 2H), 7.45 - 7.30 (m, 5H), 6.35 (s, 1H), 5.91 (s, 1H), 4.50 - 4.30 (m, 2H), 4.03 - 3.93 (m, 1H), 3.60 - 3.49 (m, 1H), 2.64 (s, 3H), 1.38 (t, J = 7.2 Hz, 3H), 0.78 (t, J = 7.2 Hz, 3H); ^{13}C NMR (101 MHz, $CDCl_3$) δ = 167.0, 166.3, 149.1, 142.1, 133.9, 130.7, 129.3, 128.5, 126.3, 123.7, 91.1, 88.0, 76.6, 63.4, 62.4, 43.4, 13.9, 13.2. HRMS (ESI-TOF) calcd for $C_{22}H_{24}N_2NaO_9S^+$ ([M+Na$^+$]) = 515.1095, Found 515.1099.

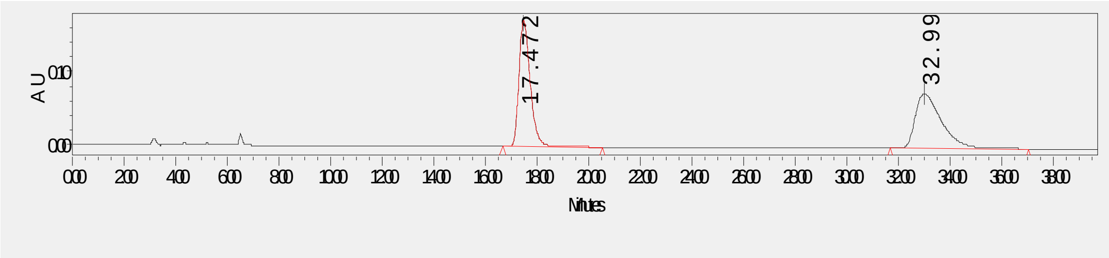

	Retention Time	Area	% Area
1	17.472	5281685	50.23
2	32.999	5232601	49.77

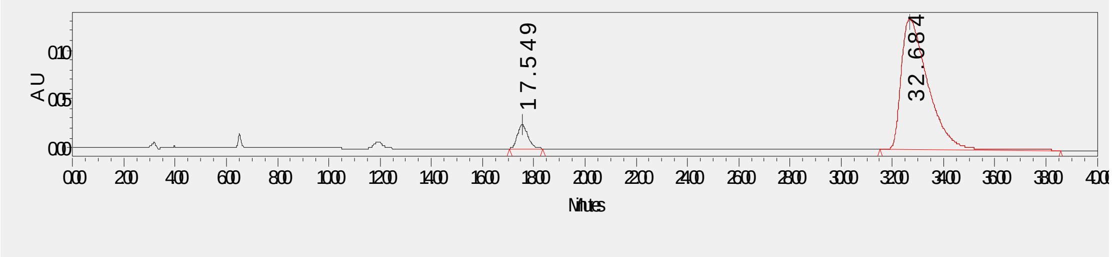

	Retention Time	Area	% Area
1	17.549	681501	6.59
2	32.684	9661414	93.41

***cis*-Diethyl 2-(4-phenylphenyl)-5-phenyl-3-methylsulfonyloxazolidine-4, 4′-dicarboxylate (3ra)**

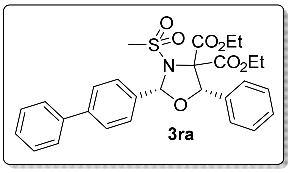

Light yellow oil, 70% yield, 93% ee. $[\alpha]_{\lambda}^{28}$ = -31.2 (c = 2.30 in CH_2Cl_2, 365 nm). (Chiralpak IA, hexane/*i*PrOH = 70/30, flow rate = 1.0 mL/min, λ = 210 nm: t_R (major) = 6.16 min, t_R (minor) = 8.02 min.) ^{1}H NMR(400 MHz, $CDCl_3$) δ = 7.86 (d, J = 8.4 Hz, 2H), 7.72 (d, J = 8.4 Hz, 2H), 7.63 (d, J = 7.2 Hz, 2H), 7.47 (t, J = 7.6 Hz, 2H), 7.41 - 7.34 (m, 6H), 6.30 (s, 1H), 5.84 (s, 1H), 4.50 - 4.33 (m, 2H), 4.02 - 3.92 (m, 1H), 3.64 - 3.53 (m, 1H), 2.57 (s, 3H), 1.40 (t, J = 7.2 Hz, 3H), 0.80 (t, J = 7.2 Hz, 3H); ^{13}C NMR (101 MHz, $CDCl_3$) δ = 167.1, 167.0, 143.4, 140.2, 134.5, 133.6, 130.1, 129.1, 129.0, 128.4, 127.9, 127.3, 127.2, 126.4, 92.1, 87.7, 77.0, 63.2, 62.2, 43.2, 14.0, 13.3. HRMS (ESI-TOF) calcd for $C_{28}H_{29}NNaO_7S^+$ ($[M+Na^+]$) = 546.1557, Found 546.1564.

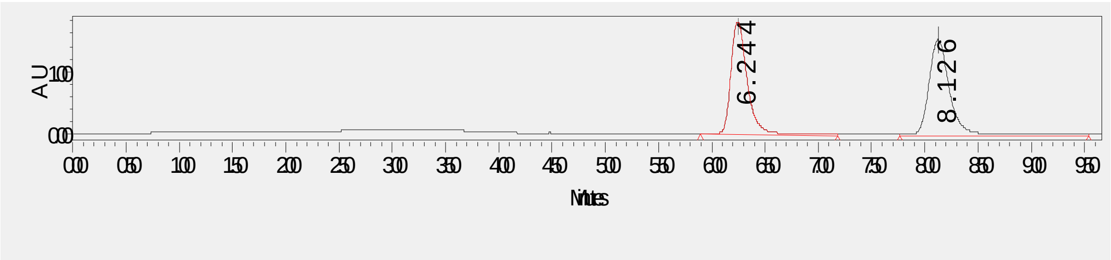

	Retention Time	Area	% Area
1	6.244	19265728	49.32
2	8.126	19798207	50.68

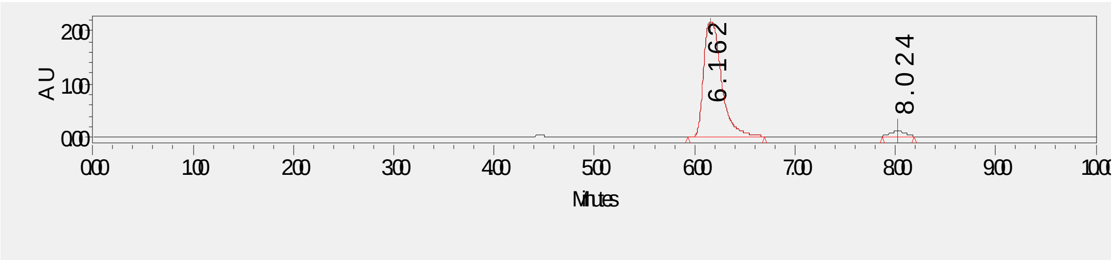

	Retention Time	Area	% Area
1	6.162	26928620	96.54
2	8.024	964882	3.46

(*2R,5S*)-**Diethyl 2-(2-naphthyl)-5-phenyl-3-methylsulfonyloxazolidine-4, 4′-dicarboxylate (3sa)**

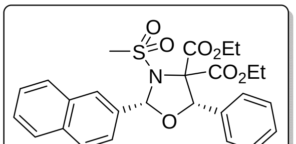

3sa

White solid, m.p. 146-148 °C, 94% yield, 93% ee. $[\alpha]_{\lambda}^{31}$ = -4.3 (*c* = 0.56 in CH_2Cl_2, λ = 365 nm). (Chiralpak IE, hexane/*i*PrOH = 70/30, flow rate = 1.0 mL/min, λ = 210 nm: *t*R (major) = 49.42 min, *t*R (minor) = 19.98 min.) ^{1}H NMR (400 MHz, $CDCl_3$) δ = 8.19 (s, 1H), 8.01 - 7.87 (m, 4H), 7.60 - 7.52 (m, 2H), 7.43 - 7.35 (m, 5H), 6.42 (s, 1H), 5.87 (s, 1H), 4.54 - 4.33 (m, 2H), 4.05 - 3.94 (m, 1H), 3.67 - 3.56 (m, 1H), 2.47 (s, 3H), 1.41 (t, *J* = 7.2 Hz, 3H), 0.82 (t, *J* = 7.2 Hz, 3H); ^{13}C NMR (101 MHz, DMSO-d_6) δ = 166.3, 166.0, 134.3, 133.8, 132.1, 132.0, 130.0, 129.0, 128.4, 128.3, 128.3, 127.7, 127.4, 126.7, 126.5, 125.4, 91.7, 86.7, 76.2, 62.5, 61.7, 42.7, 13.7, 13.1. HRMS (ESI-TOF) calcd for $C_{26}H_{27}NO_7SNa$ ($[M+Na^+]$) = 520.1400, Found 520.1405.

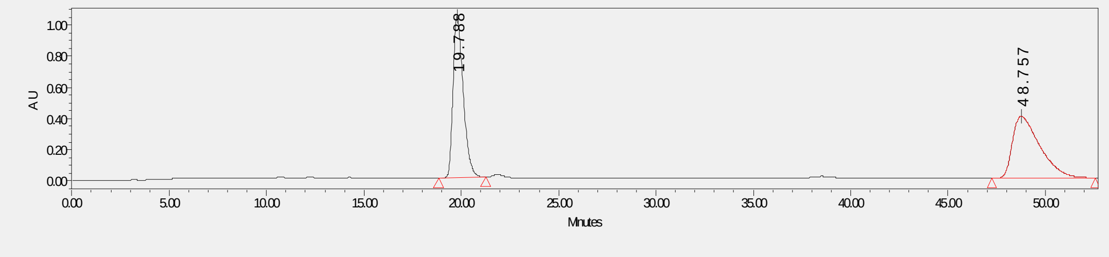

	Retention Time	Area	% Area
1	19.788	37694278	49.40
2	48.757	38608447	50.60

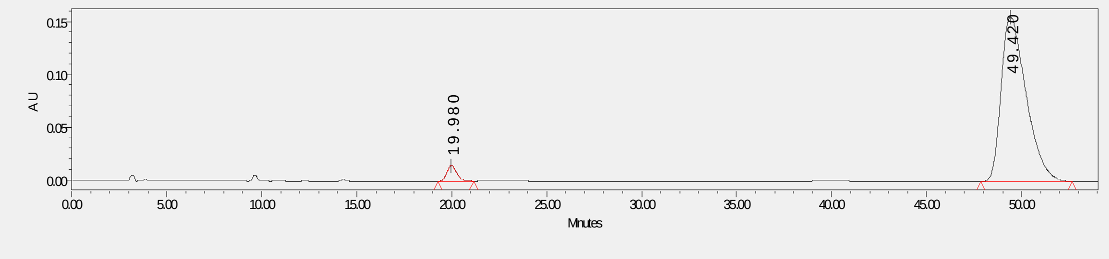

	Retention Time	Area	% Area
1	19.980	522238	3.54
2	49.420	14232749	96.46

***cis*-Diethyl-2-phenyl-5-(4-chlorophenyl)-3-methylsulfonyloxazolidine-4, 4′-dicarboxylate (3ib)**

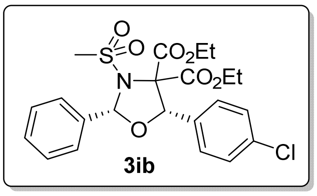

Colorless oil, 70% yield, 90% ee. $[\alpha]_{\lambda}^{31}$ = +91.7 (c = 0.37 in CH_2Cl_2, λ = 365 nm). (Chiralpak IE, hexane/iPrOH = 70/30, flow rate = 1.0 mL/min, λ = 210 nm: t_R (major) = 16.40 min, t_R (minor) = 10.27min.) ^{1}H NMR (400 MHz, $CDCl_3$) δ = 7.80 - 7.72 (m, 2H), 7.54 - 7.46 (m, 3H), 7.40 - 7.27 (m, 4H), 6.23 (s, 1H), 5.79 (s, 1H), 4.49 - 4.30 (m, 2H), 4.05 – 3.94 (m, 1H), 3.73 - 3.62 (m, 1H), 2.49 (s, 3H), 1.38 (t, J = 7.2 Hz, 3H), 0.87 (t, J = 7.2 Hz, 3H); ^{13}C NMR (101 MHz, $CDCl_3$) δ = 166.9, 166.8, 134.9, 134.4, 133.1, 130.7, 129.6, 128.6, 128.5, 127.8, 92.4, 86.9, 76.8, 63.3, 62.3, 43.0, 13.9, 13.3. HRMS (ESI-TOF) calcd for $C_{22}H_{24}NO_7S^{34.9689}ClNa$ ($[M+Na^+]$) = 504.0855, Found 504.0857. HRMS (ESI-TOF) calcd for $C_{22}H_{24}NO_7S^{36.9659}ClNa$ ($[M+Na^+]$) = 506.0825, Found 506.0826.

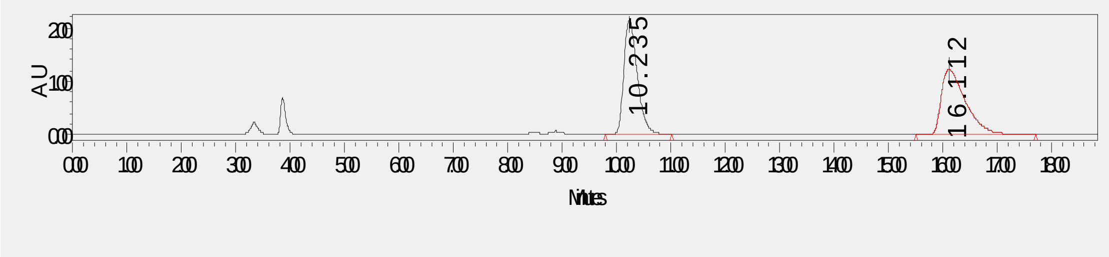

	Retention Time	Area	% Area
1	10.235	36950127	49.44
2	16.112	37783222	50.56

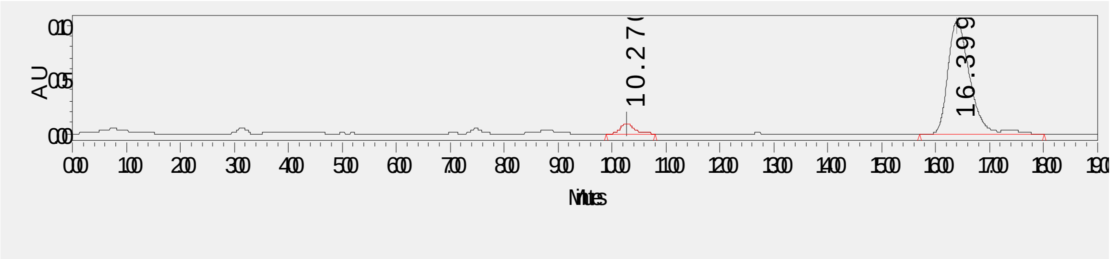

	Retention Time	Area	% Area
1	10.270	157976	5.06
2	16.399	2963710	94.94

***cis*-Diethyl 2-(3-chlorophenyl)-5-(3-chlorophenyl)-3-methylsulfonyloxazolidine-4, 4′-dicarboxylate (3lc)**

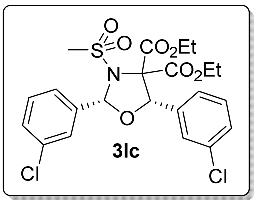

Colorless oil, 38% yield, 89% ee. $[\alpha]_{\lambda}^{26}$ = +13.4 (c = 0.30 in CH_2Cl_2, λ = 365 nm). (Chiralpak IE, hexane/*i*PrOH = 70/30, flow rate = 1.0 mL/min, λ = 210 nm: t_R (major) = 22.62 min, t_R (minor) = 10.71 min.) ^{1}H NMR (400 MHz, $CDCl_3$) δ = 7.77 - 7.74 (m, 1H), 7.68 - 7.63 (m, 1H), 7.51 - 7.41 (m, 2H), 7.38 - 7.29 (m, 3H), 7.25 - 7.21 (m, 1H), 6.20 (s, 1H), 5.79 (s, 1H), 4.49 - 4.31 (m, 2H), 4.06 - 3.95 (m, 1H), 3.77 - 3.66 (m, 1H), 2.61 (s, 3H), 1.38 (t, J = 7.2 Hz, 3H), 0.88 (t, J = 7.2 Hz, 3H); ^{13}C NMR (101 MHz, $CDCl_3$) δ = 166.6, 166.6, 136.7, 136.2, 134.6, 134.4, 130.9, 129.9, 129.7, 129.5, 129.2, 127.8, 126.5, 124.6, 91.7, 86.8, 76.6, 63.5, 62.4, 43.2, 13.9, 13.3. HRMS (ESI-TOF) calcd for $C_{22}H_{23}{}^{34.9689}Cl_2NNaO_7S^+$ ([M+Na$^+$]) = 538.0465, Found 538.0477. HRMS (ESI-TOF) calcd for $C_{22}H_{23}{}^{34.9689}Cl^{36.9659}ClNNaO_7S^+$ ([M+Na$^+$]) = 540.0435, Found 540.0454.

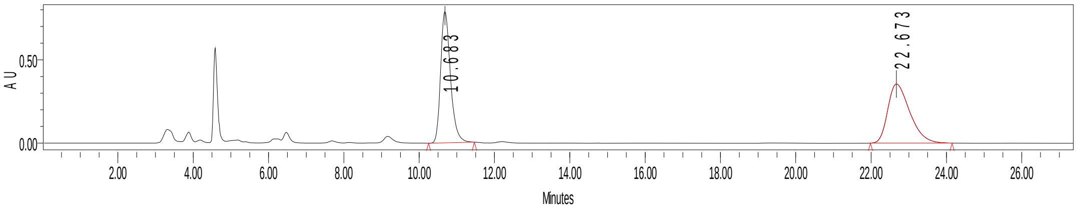

	Retention Time	Area	% Area
1	10.683	14251319	49.69
2	22.673	14426564	50.31

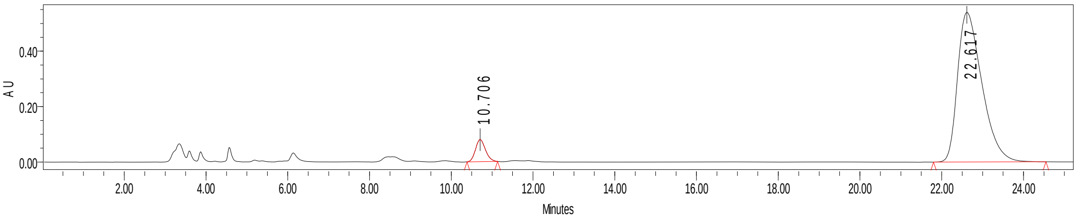

	Retention Time	Area	% Area
1	10.706	1347917	5.70
2	22.617	22296304	94.30

(*2R,5S*)-**Diethyl 2-(4-chlorophenyl)-5-(4-methylphenyl)-3-methylsulfonyloxazolidine-4, 4′-dicarboxylate (3kd)**

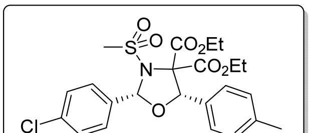
3kd

Colorless oil, 51% yield, 84% ee. $[\alpha]_{\lambda}^{32}$ = +45.1 (*c* = 0.29 in CH_2Cl_2, λ = 365 nm). (Chiralpak IE, hexane/*i*PrOH = 70/30, flow rate = 1.0 mL/min, λ = 210 nm: t_R (major) = 19.99 min, t_R (minor) = 11.94 min.) ^{1}H NMR (400 MHz, $CDCl_3$) δ = 7.73 (d, *J* = 8.4 Hz, 2H), 7.46 (d, *J* = 8.4 Hz, 2H), 7.22 (d, *J* = 8.4 Hz, 2H), 7.17 (d, *J* = 8.0 Hz, 2H), 6.21 (s, 1H), 5.78 (s, 1H), 4.49 - 4.28 (m, 2H), 4.02 - 3.90 (m, 1H), 3.65 - 3.52 (m, 1H), 2.55 (s, 3H), 2.36 (s, 3H), 1.38 (t, *J* = 7.2 Hz, 3H), 0.80 (t, *J* = 7.2 Hz, 3H); ^{13}C NMR (101 MHz, $CDCl_3$) δ = 167.0, 166.9, 139.1, 136.5, 133.4, 131.3, 131.0, 129.0, 128.8, 126.3, 91.5, 87.8, 76.8, 63.2, 62.2, 43.2, 21.2, 13.9, 13.2. HRMS (ESI-TOF) calcd for $C_{23}H_{26}{}^{34.9689}ClNNaO_7S^+$ ([M+Na$^+$]) = 518.1011, Found 518.1019. HRMS (ESI-TOF) calcd for $C_{23}H_{26}{}^{36.9659}ClNNaO_7S^+$ ([M+Na$^+$]) = 520.0982, Found 520.0997.

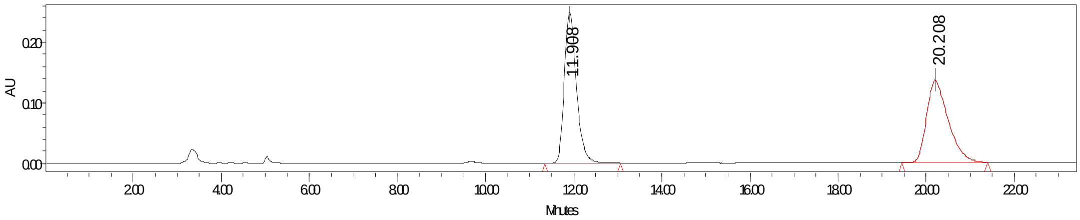

	Retention Time	Area	% Area
1	11.908	4758408	50.68
2	20.208	4629832	49.32

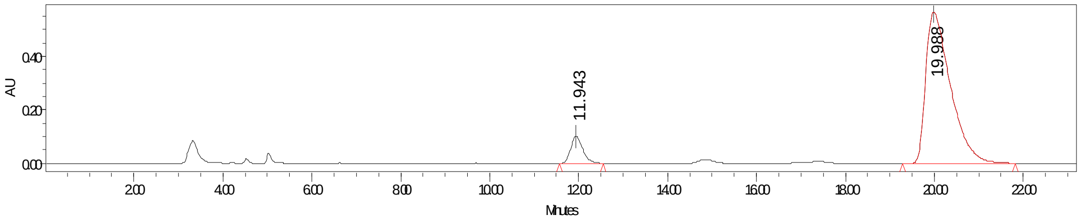

	Retention Time	Area	% Area
1	11.943	1904690	8.02
2	19.988	21836030	91.98

(*2R,5S*)-**Diethyl 2-(4-chlorophenyl)-5-(3-methylphenyl)-3-methylsulfonyloxazolidine-4, 4′-dicarboxylate (3ke)**

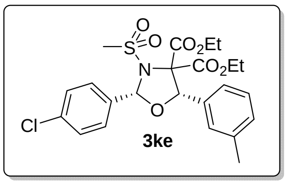

Colorless oil, 73% yield, 94% ee. $[\alpha]_{\lambda}^{32}$ = +14.9 (*c* = 0.57 in CH_2Cl_2, λ = 365 nm). (Chiralpak IE, hexane/*i*PrOH = 70/30, flow rate = 1.0 mL/min, λ = 210 nm: t_R (major) = 16.55 min, t_R (minor) = 11.68 min.) ^{1}H NMR (400 MHz, $CDCl_3$) δ = 7.74 (d, *J* = 8.4 Hz, 2H), 7.47 (d, *J* = 8.8 Hz, 2H), 7.25 (t, *J* = 8.0 Hz, 1H), 7.20 - 7.10 (m, 3H), 6.21 (s, 1H), 5.78 (s, 1H), 4.53 - 4.26 (m, 2H), 4.04 - 3.90 (m, 1H), 3.64 - 3.50 (m, 1H), 2.56 (s, 3H), 2.35 (s, 3H), 1.38 (t, *J* = 7.2 Hz, 3H), 0.79 (t, *J* = 7.2 Hz, 3H); ^{13}C NMR (101 MHz, $CDCl_3$) δ = 167.0, 166.9, 138.1, 136.5, 134.2, 133.4, 131.0, 129.9, 128.8, 128.3, 126.9, 123.5, 91.6, 87.8, 76.8, 63.2, 62.1, 43.2, 21.4, 13.9, 13.2. HRMS (ESI-TOF) calcd for $C_{23}H_{26}{}^{34.9689}ClNNaO_7S^+$ ([M+Na$^+$]) = 518.1011, Found 518.1013. HRMS (ESI-TOF) calcd for $C_{23}H_{26}{}^{36.9659}ClNNaO_7S^+$ ([M+Na$^+$]) = 520.0982, Found 520.0991.

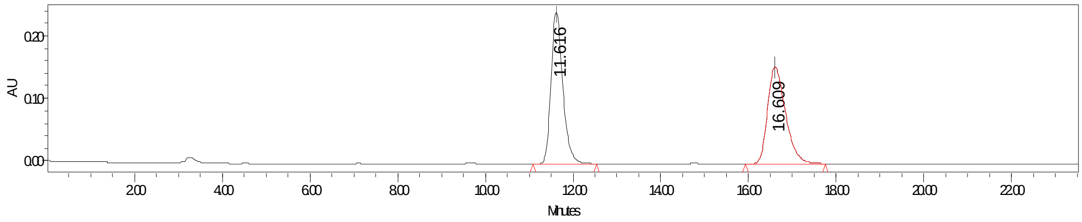

	Retention Time	Area	% Area
1	11.616	4478707	50.47
2	16.609	4395193	49.53

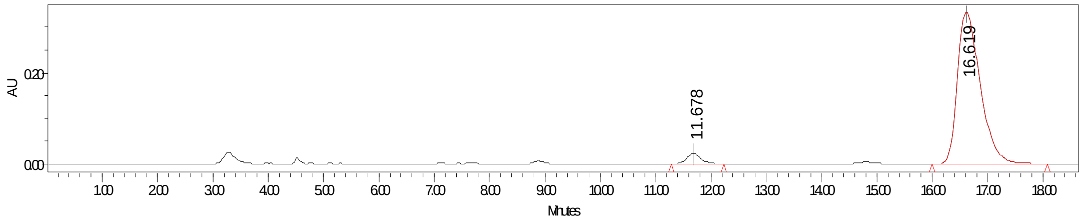

	Retention Time	Area	% Area
1	11.676	612739	3.22
2	16.546	18439245	96.78

(*2R*,*5S*)-**Diethyl 2-(3-methylphenyl)-5-(3-methylphenyl)-3-methylsulfonyloxazolidine-4, 4′-dicarboxylate (3te)**

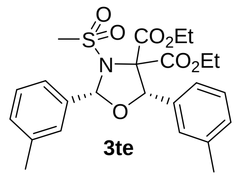

3te

Colorless oil, 51% yield, 92% ee. $[\alpha]_{\lambda}^{30}$ = +44.2 (*c* = 1.00 in CH_2Cl_2, λ = 365 nm). (Chiralpak IE, hexane/*i*PrOH = 70/30, flow rate = 1.0 mL/min, λ = 210 nm: t_R (major) = 26.00 min, t_R (minor) = 12.96 min.) ^{1}H NMR (400 MHz, $CDCl_3$) δ = 7.61 (d, *J* = 7.6 Hz, 1H), 7.55 (s, 1H), 7.38 (t, *J* = 7.6 Hz, 1H), 7.29 (d, *J* = 7.6 Hz, 1H), 7.24 (d, *J* = 8.0 Hz, 1H), 7.19 - 7.13 (m, 3H), 6.19 (s, 1H), 5.75 (s, 1H), 4.53 - 4.28 (m, 2H), 4.02 - 3.88 (m, 1H), 3.67 - 3.55 (m, 1H), 2.51 (s, 3H), 2.43 (s, 3H), 2.35 (s, 3H), 1.40 (t, *J* = 7.2 Hz, 3H), 0.82 (t, *J* = 7.2 Hz, 3H); ^{13}C NMR (101 MHz, $CDCl_3$) δ = 167.2, 166.8, 138.2, 137.9, 134.4, 131.3, 130.3, 129.7, 128.4, 128.2, 127.0, 126.6, 123.5, 92.3, 87.7, 76.9, 63.0, 62.0, 43.0, 21.4, 13.9, 13.2. HRMS (ESI-TOF) calcd for $C_{24}H_{29}NNaO_7S^+$ ([M+Na$^+$]) = 498.1557, Found 498.1566.

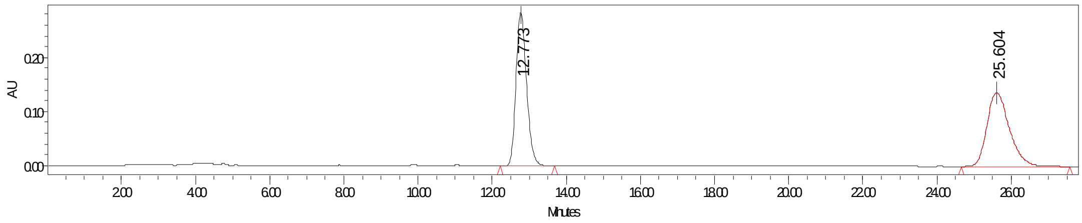

	Retention Time	Area	% Area
1	12.773	5663981	49.80
2	25.604	5710018	50.20

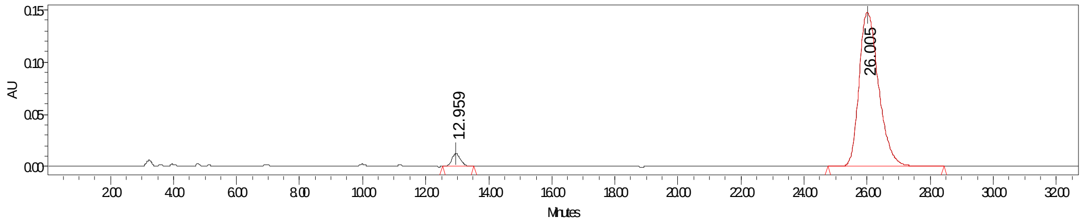

	Retention Time	Area	% Area
1	12.959	250292	3.78
2	26.005	6369513	96.22

(*2R,5S*)-**Diethyl 2-(4-chlorophenyl)-5-(3-methoxylphenyl)-3-methylsulfonyloxazolidine-4, 4′-dicarboxylate (3kf)**

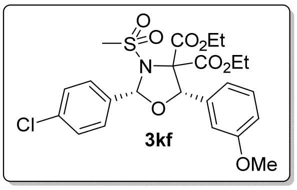

3kf

Colorless oil, 70% yield, 93% ee. $[\alpha]_{\lambda}^{30}$ = -2.4 (*c* = 1.55 in CH_2Cl_2, λ = 365 nm). (Chiralpak IE, hexane/*i*PrOH = 70/30, flow rate = 1.0 mL/min, λ = 210 nm: t_R (major) = 25.51 min, t_R (minor) = 13.75 min.) ^1H NMR (400 MHz, $CDCl_3$) δ = 7.73 (d, *J* = 8.4 Hz, 2H), 7.46 (d, *J* = 8.4 Hz, 2H), 7.33 - 7.27 (m, 1H), 6.97 - 6.85 (m, 3H), 6.21 (s, 1H), 5.79 (s, 1H), 4.52 - 4.25 (m, 2H), 4.03 - 3.92 (m, 1H), 3.80 (s, 3H), 3.68 - 3.57 (m, 1H), 2.56 (s, 3H), 1.38 (t, *J* = 7.2 Hz, 3H), 0.83 (t, *J* = 7.2 Hz, 3H); ^{13}C NMR (101 MHz, $CDCl_3$) δ = 166.9, 166.8, 159.6, 136.6, 135.7, 133.4, 131.0, 129.5, 128.8, 118.8, 114.3, 112.2, 91.6, 87.5, 76.8, 63.2, 62.2, 55.3, 43.2, 13.9, 13.3. HRMS (ESI-TOF) calcd for $C_{23}H_{26}{}^{34.9689}ClNNaO_8S^+$ ([M+Na$^+$]) = 534.0960, Found 534.0962. HRMS (ESI-TOF) calcd for $C_{23}H_{26}{}^{36.9659}ClNNaO_8S^+$ ([M+Na$^+$]) = 536.0931, Found 536.0958.

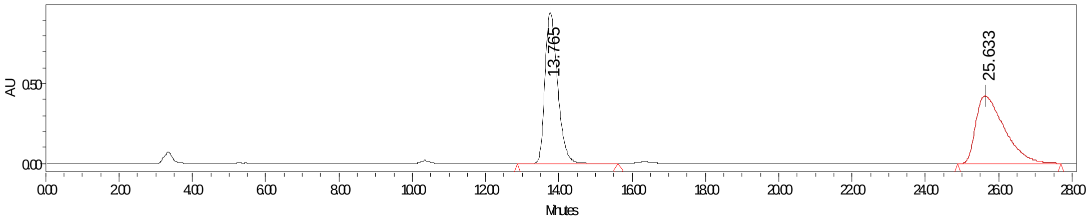

	Retention Time	Area	% Area
1	13.765	22737750	49.89
2	25.633	22834508	50.11

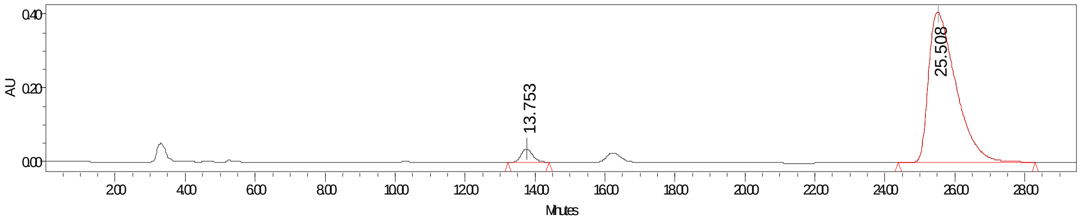

	Retention Time	Area	% Area
1	13.753	828211	3.64
2	25.508	21945739	96.36

(*2R,5S*)-**Diethyl 2-(4-chlorophenyl)-5-(3-thienyl)-3-methylsulfonyloxazolidine-4, 4′-dicarboxylate (3kg)**

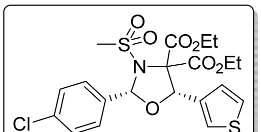
3kg

Colorless oil, 84% yield, 91% ee. $[\alpha]_{\lambda}^{26}$ = +5.8 (*c* = 0.64 in CH_2Cl_2, λ = 365 nm). (Chiralpak IE, hexane/*i*PrOH = 70/30, flow rate = 1.0 mL/min, λ = 210 nm: t_R (major) = 15.12 min, t_R (minor) = 12.30 min.) ^1H NMR (400 MHz, $CDCl_3$) δ = 7.70 (d, *J* = 8.4 Hz, 2H), 7.45 (d, *J* = 8.4 Hz, 2H), 7.33 (d, *J* = 3.2 Hz, 2H), 7.04 (t, *J* = 3.2 Hz, 1H), 6.19 (s, 1H), 5.87 (s, 1H), 4.49 - 4.27 (m, 2H), 4.10 - 3.97 (m, 1H), 3.76 - 3.64 (m, 1H), 2.56 (s, 3H), 1.37 (t, *J* = 7.2 Hz, 3H), 0.94 (t, *J* = 7.2 Hz, 3H); ^{13}C NMR (101 MHz, $CDCl_3$) δ = 167.0, 166.7, 136.5, 135.2, 133.4, 130.9, 128.8, 126.0, 125.8, 123.1, 91.6, 84.7, 76.3, 63.3, 62.4, 43.2, 13.9, 13.4. HRMS (ESI-TOF) calcd for $C_{20}H_{22}{}^{34.9689}ClNNaO_7S_2^+$ ([M+Na$^+$]) = 510.0419, Found 510.0426. HRMS (ESI-TOF) calcd for $C_{20}H_{22}{}^{36.9659}ClNNaO_7S_2^+$ ([M+Na$^+$]) = 512.0389, Found 512.0407.

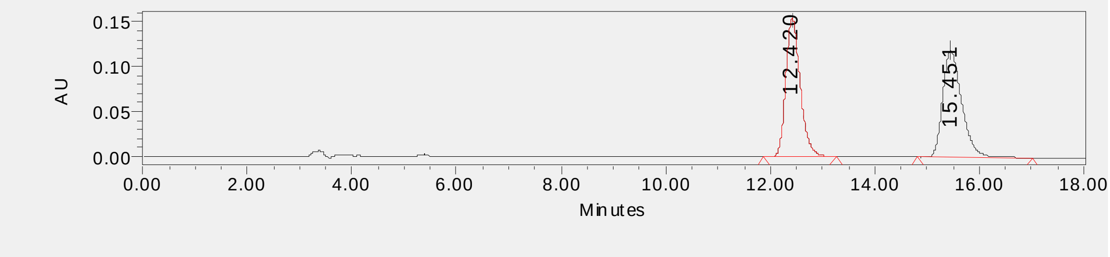

	Retention Time	Area	% Area
1	12.420	2991143	49.79
2	15.451	3016342	50.21

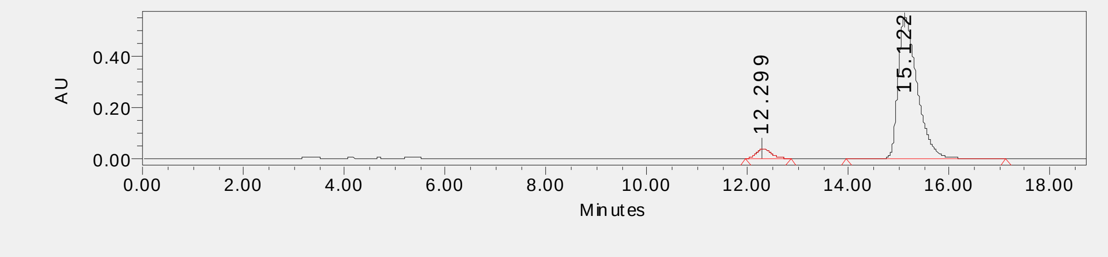

	Retention Time	Area	% Area
1	12.299	721904	4.74
2	15.122	14519480	95.26

(2*R*,5*R*)-**Diethyl 2-(4-chlorophenyl)-5-(2-furyl)-3-methylsulfonyloxazolidine-4, 4′-dicarboxylate (3kh)**

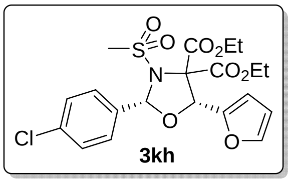

Colorless oil, 93% yield, 94% ee. $[\alpha]_{\lambda}^{32}$ = +20.1 (*c* = 0.80 in CH_2Cl_2, λ = 365 nm). (Chiralpak IA, hexane/*i*PrOH = 70/30, flow rate = 1.0 mL/min, λ = 210 nm: t_R (major) = 5.78 min, t_R (minor) = 10.02 min.) ^{1}H NMR (400 MHz, $CDCl_3$) δ = 7.68 (d, *J* = 8.4 Hz, 2H), 7.45 (t, *J* = 8.4 Hz, 3H), 6.45 (d, *J* = 3.2 Hz, 1H), 6.42 - 6.34 (m, 1H), 6.21 (s, 1H), 5.79 (s, 1H), 4.46 - 4.28 (m, 2H), 4.25 - 4.13 (m, 1H), 3.92 - 3.80 (m, 1H), 2.61 (s, 3H), 1.37 (t, *J* = 7.2 Hz, 3H), 1.08 (t, *J* = 7.2 Hz, 3H); ^{13}C NMR (101 MHz, $CDCl_3$) δ = 167.0, 166.8, 147.2, 143.5, 136.5, 133.5, 130.9, 128.8, 110.6, 110.3, 91.8, 81.8, 75.5, 63.4, 62.8, 43.3, 13.8, 13.6. HRMS (ESI-TOF) calcd for $C_{20}H_{22}{}^{34.9689}ClNNaO_8S^+$ ([M+Na$^+$]) = 494.0647, Found 494.0659. HRMS (ESI-TOF) calcd for $C_{20}H_{22}{}^{36.9659}ClNNaO_8S^+$ ([M+Na$^+$]) = 496.0618, Found 496.0642.

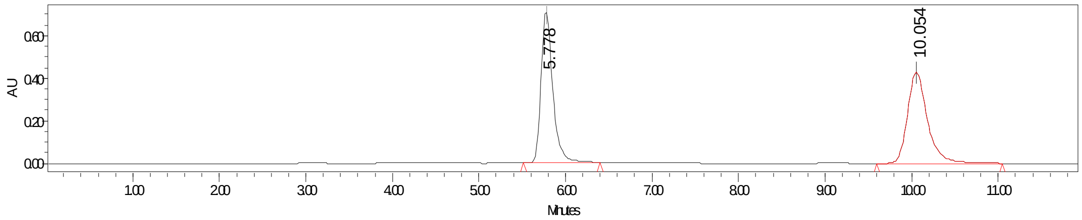

	Retention Time	Area	% Area
1	5.778	6616999	49.26
2	10.054	6816254	50.74

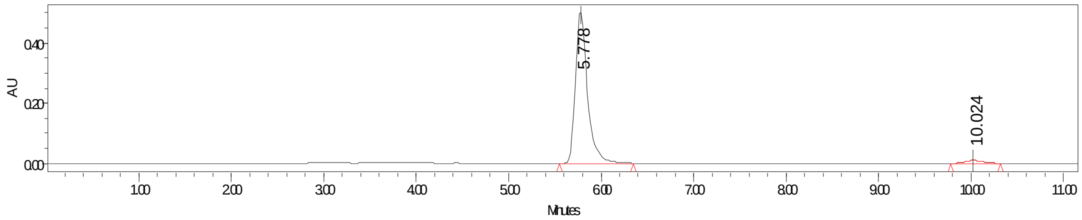

	Retention Time	Area	% Area
1	5.778	4536714	96.96
2	10.024	142112	3.04

(*2R,5S*)-**Diethyl 2-(4-chlorophenyl)-5-(*E*)-styryl-3-methylsulfonyloxazolidine-4, 4′-dicarboxylate (3ki)**

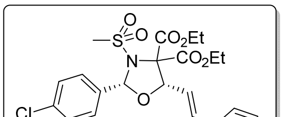

3ki

Colorless oil, 84% yield, 55% ee. $[\alpha]_{\lambda}^{26}$ = -2.5 (*c* = 0.80 in CH_2Cl_2, λ = 365 nm). (Chiralpak IA, hexane/*i*PrOH = 70/30, flow rate = 1.0 mL/min, λ = 210 nm: t_R (major) = 25.51 min, t_R (minor) = 13.75 min.) ^{1}H NMR (400 MHz, $CDCl_3$) δ = 7.61 (d, *J* = 8.0 Hz, 2H), 7.46 - 7.37 (m, 4H), 7.36 - 7.26 (m, 3H), 6.72 (d, *J* = 15.6 Hz, 1H), 6.28 (dd, *J* = 6.8, 15.6 Hz, 1H), 6.14 (s, 1H), 5.33 (d, *J* = 6.8 Hz, 1H), 4.46 - 4.19 (m, 4H), 2.57 (s, 3H), 1.35 (t, *J* = 7.2 Hz, 3H), 1.25 (t, *J* = 7.2 Hz, 3H); ^{13}C NMR (101 MHz, $CDCl_3$) δ = 167.1, 166.5, 136.5, 135.6, 134.0, 133.5, 130.8, 128.8, 128.7, 128.6, 126.8, 121.3, 91.7, 86.9, 75.9, 63.3, 62.7, 43.2, 14.1, 13.9. HRMS (ESI-TOF) calcd for $C_{24}H_{26}{}^{34.9689}ClNNaO_7S^+$ ([M+Na$^+$]) = 530.1011, Found 530.1014. HRMS (ESI-TOF) calcd for $C_{24}H_{26}{}^{36.9659}ClNNaO_7S^+$ ([M+Na$^+$]) = 532.0982, Found 532.0988.

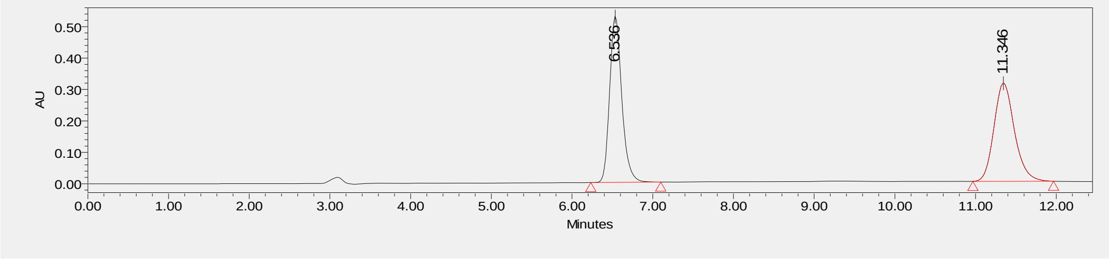

	Retention Time	Area	% Area
1	6.536	5523739	49.99
2	11.346	5526605	50.01

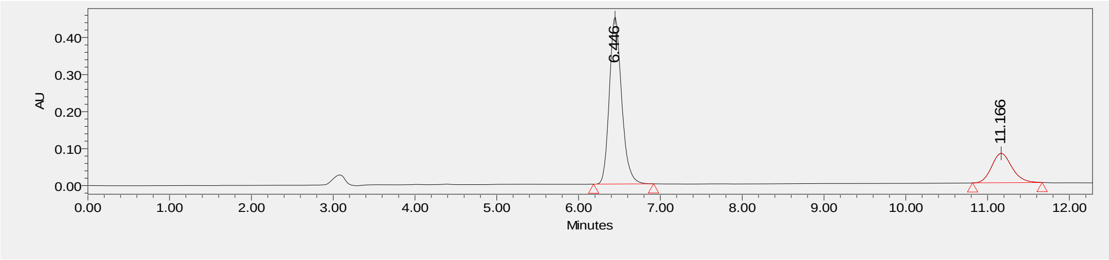

	Retention Time	Area	% Area
1	6.446	4712372	77.39
2	11.166	1376722	22.61

(L) References

1 (a) K. Y. Lee, C. G. Lee and J. N. Kim, *Tetrahedron Lett.*, 2003, **44**, 1231; (b) D.-J.

Dong, H.-H. Li and S.-K. Tian, *J. Am. Chem. Soc.*, 2010, **132**, 5018.

2 (a) X. Wu, L. Li and J. Zhang, *Adv. Synth. Catal.*, 2012, **354**, 3485; (b) R. H. Fan and Y. Ye, *Adv. Synth. Catal.*, 2008, **350**, 1526.

3 (a) Y. H. Wen, X. Huang, J. L. Huang, Y. Xiong, B. Qin and X. M. Feng, *Synlett.*, 2005, **16**, 2445; (b) X. H. Liu, L. L. Lin and X. M. Feng, *Acc. Chem. Res.*, 2011, **44**, 574; (c) X. H. Liu, L. L. Lin and X. M. Feng, *Org. Chem. Front.*, 2014, **1**, 298.

4 X. Wu, L. Li and J. Zhang, *Chem. Commun.*, 2011, **47**, 7824.

5 M. Vaultier and R. Carrie, *Tetrahedron Lett.*, 1978, **19**, 1195.

(M) The X-ray data for 3sa

The following single crystal **3sa** was recrystallized from Et_2O. CCDC-1057118 (**3sa**) contain the supplementary crystallographic data for this paper. These data can be obtained free of charge from the Cambridge Crystallographic Data Centre via www.ccdc.cam.ac.uk./ data_request/cif.

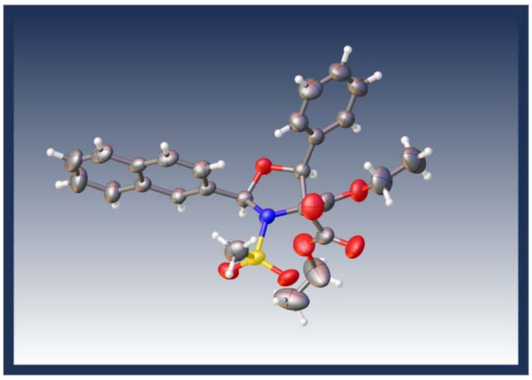

Table 1 Crystal data and structure refinement for fxm-lyt-20150303.

Identification code	fxm-lyt-20150303
Empirical formula	$C_{26}H_{27}NO_7S$
Formula weight	497.54
Temperature/K	293
Crystal system	monoclinic
Space group	$P2_1$
a/Å	11.1671(2)
b/Å	7.62440(10)
c/Å	14.6056(3)
α/°	90
β/°	94.103(2)
γ/°	90

Volume/Å^3	1240.37(4)
Z	2
ρ_{calc}g/cm^3	1.332
μ/mm^{-1}	1.552
F(000)	524.0
Crystal size/mm^3	0.4 × 0.3 × 0.2
Radiation	CuKα (λ = 1.54184)
2Θ range for data collection/°	9.644 to 134.112
Index ranges	$-13 \leq h \leq 12$, $-9 \leq k \leq 6$, $-17 \leq l \leq 17$
Reflections collected	12899
Independent reflections	3607 [R_{int} = 0.0319, R_{sigma} = 0.0223]
Data/restraints/parameters	3607/1/319
Goodness-of-fit on F^2	1.070
Final R indexes [I>=2σ (I)]	R_1 = 0.0526, wR_2 = 0.1353
Final R indexes [all data]	R_1 = 0.0534, wR_2 = 0.1366
Largest diff. peak/hole / e Å^{-3}	0.25/-0.39
Flack parameter	0.011(12)

(N) Copies of NMR spectra

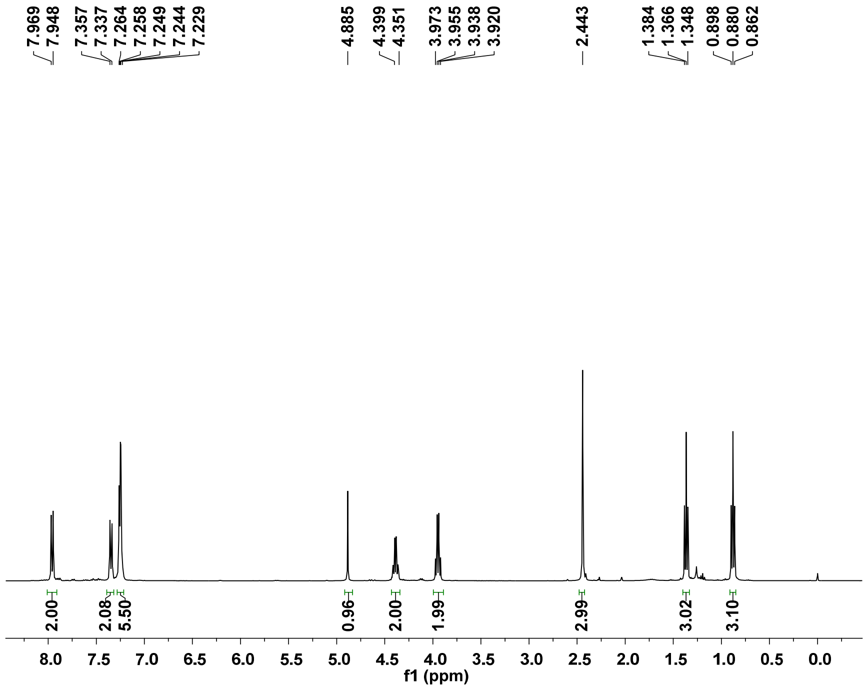

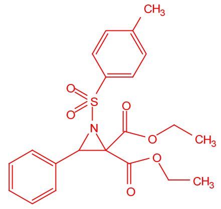

Current Data Parameters

F2 - Acquisition Parameters
DATE: 2014-10-08T00:36:32
PULPROG: zg30
TD: 32768
Solvent: CDCl3
NS: 32
DS: undefined
SWH: 8223.7 Hz
AQ: undefined
TE: 298.4 C

======== CHANNEL f1 ========
NUC1: 1H
P1: 9.93 usec
SFO1: undefined MHz

F2 - Processing Parameters
SI: 65536
DC: 0.05
LB: 0.30 Hz
First Point: 0.50
FT: Hyper Quadrature
Phase: Manual
Ph0: 89.44
Ph1: 19.58

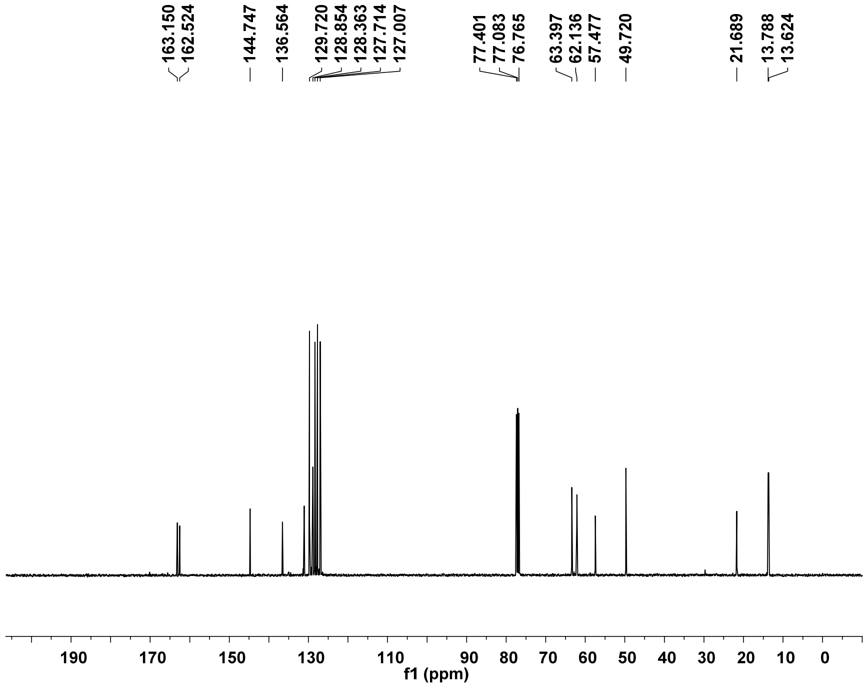

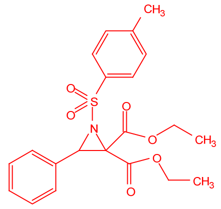

Current Data Parameters

F2 - Acquisition Parameters
DATE: 2014-10-08T01:06:44
PULPROG: zgpg30
TD: 32768
Solvent: CDCl3
NS: 512
DS: undefined
SWH: 24038.5 Hz
AQ: undefined
TE: 298.8 C

======== CHANNEL f1 ========
NUC1: 13C
P1: 9.63 usec
SFO1: undefined MHz

F2 - Processing Parameters
SI: 65536
DC: 0.05
LB: 1.00 Hz
First Point: 0.50
FT: Hyper Quadrature
Phase: Manual
Ph0: -75.54
Ph1: 67.06

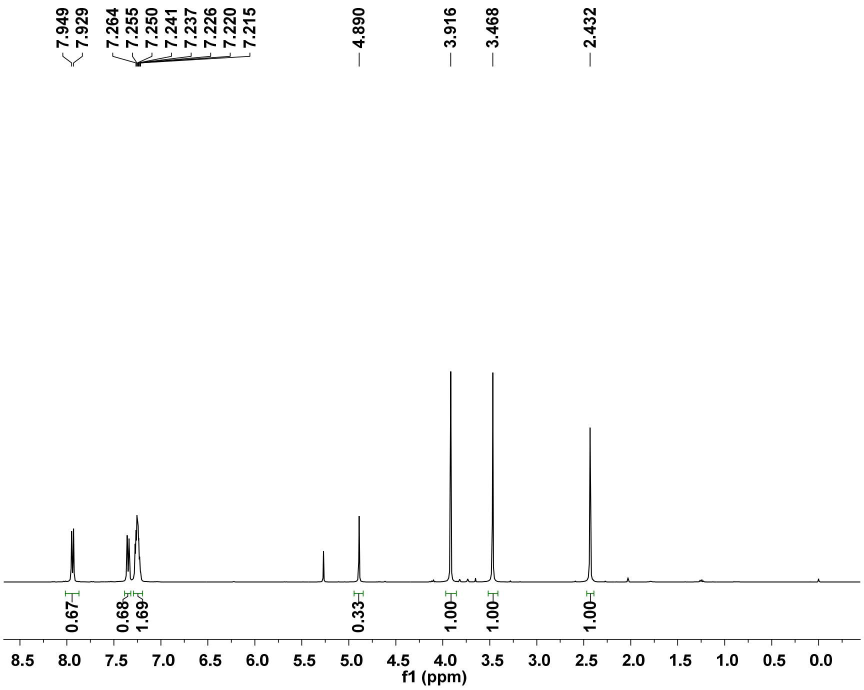

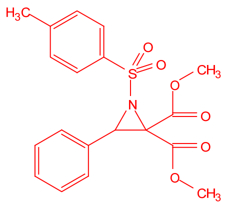

Current Data Parameters

F2 - Acquisition Parameters
DATE: 2014-12-19T21:04:27
PULPROG: zg30
TD: 32768
Solvent: CDCl3
NS: 32
DS: undefined
SWH: 8223.7 Hz
AQ: undefined
TE: 293.6 C

======== CHANNEL f 1 ========
NUC1: 1H
P1: 9.93 usec
SFO1: undefined MHz

F2 - Processing Parameters
SI: 65536
DC: 0.05
LB: 0.30 Hz
First Point: 0.50
FT: Hyper Quadrature
Phase: Manual
Ph0: -258.90
Ph1: 21.62

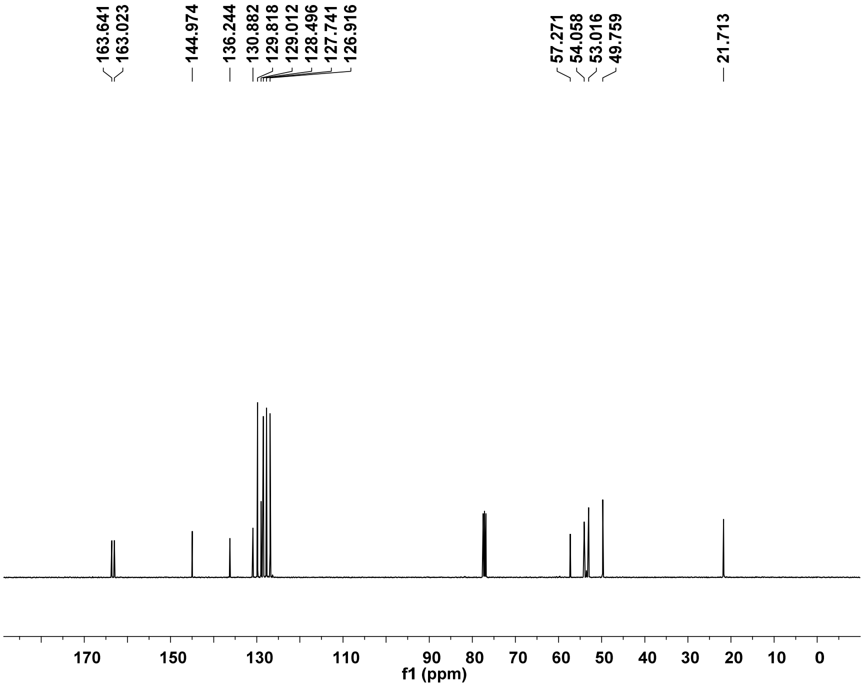

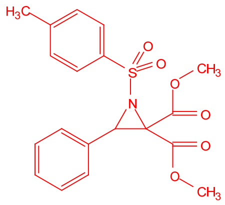

Current Data Parameters

F2 - Acquisition Parameters
DATE: 2014-12-19T21:34:39
PULPROG: zgpg30
TD: 32768
Solvent: CDCl3
NS: 512
DS: undefined
SWH: 24038.5 Hz
AQ: undefined
TE: 294.2 C

======== CHANNEL f 1 ========
NUC1: 13C
P1: 9.63 usec
SFO1: undefined MHz

F2 - Processing Parameters
SI: 65536
DC: 0.05
LB: 1.00 Hz
First Point: 0.50
FT: Hyper Quadrature
Phase: Manual
Ph0: -60.25
Ph1: 61.82

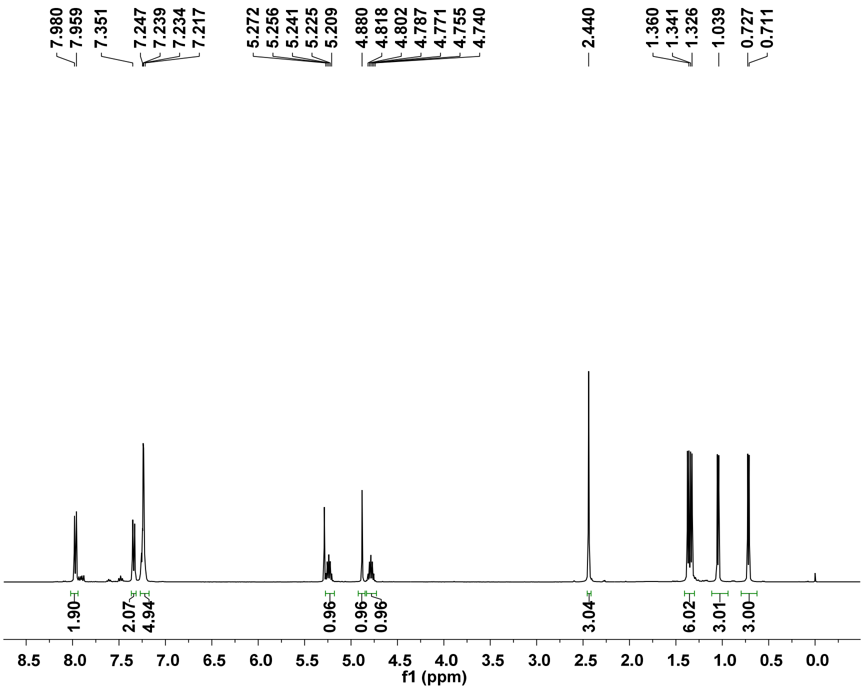

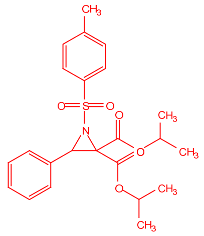

Current Data Parameters

F2 - Acquisition Parameters
DATE: 2014-11-05T23:13:36
PULPROG: zg30
TD: 32768
Solvent: CDCl3
NS: 32
DS: undefined
SWH: 8223.7 Hz
AQ: undefined
TE: 295.6 C

======== CHANNEL f1 ========
NUC1: 1H
P1: 9.93 usec
SFO1: undefined MHz

F2 - Processing Parameters
SI: 65536
DC: 0.05
LB: 0.30 Hz
First Point: 0.50
FT: Hyper Quadrature
Phase: Manual
Ph0: 91.00
Ph1: 20.85

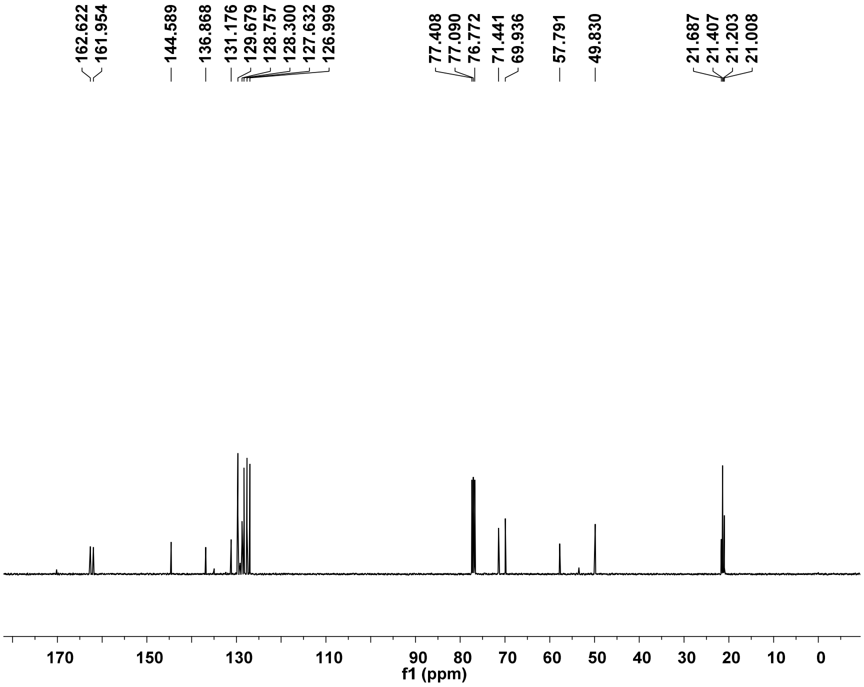

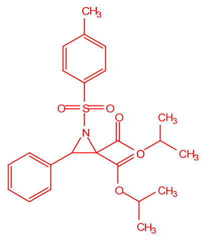

Current Data Parameters

F2 - Acquisition Parameters
DATE: 2014-11-05T23:29:11
PULPROG: zgpg30
TD: 32768
Solvent: CDCl3
NS: 256
DS: undefined
SWH: 24038.5 Hz
AQ: undefined
TE: 296.1 C

======== CHANNEL f1 ========
NUC1: 13C
P1: 9.63 usec
SFO1: undefined MHz

F2 - Processing Parameters
SI: 65536
DC: 0.05
LB: 1.00 Hz
First Point: 0.50
FT: Hyper Quadrature
Phase: Manual
Ph0: -70.01
Ph1: 58.69

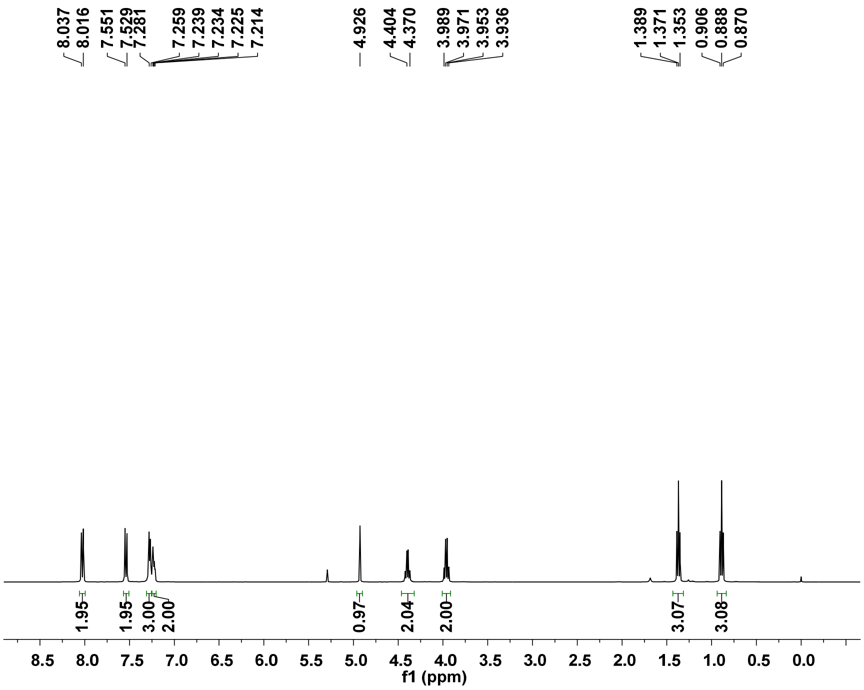

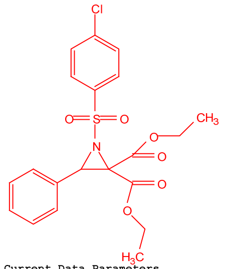

Current Data Parameters

F2 - Acquisition Parameters
DATE: 2014-11-25T22:41:21
PULPROG: zg30
TD: 32768
Solvent: CDCl3
NS: 32
DS: undefined
SWH: 8223.7 Hz
AQ: undefined
TE: 295.7 C

======== CHANNEL f1 ========
NUC1: 1H
P1: 9.93 usec
SFO1: undefined MHz

F2 - Processing Parameters
SI: 65536
DC: 0.05
LB: 0.30 Hz
First Point: 0.50
FT: Hyper Quadrature
Phase: Manual
Ph0: 90.23
Ph1: 19.22

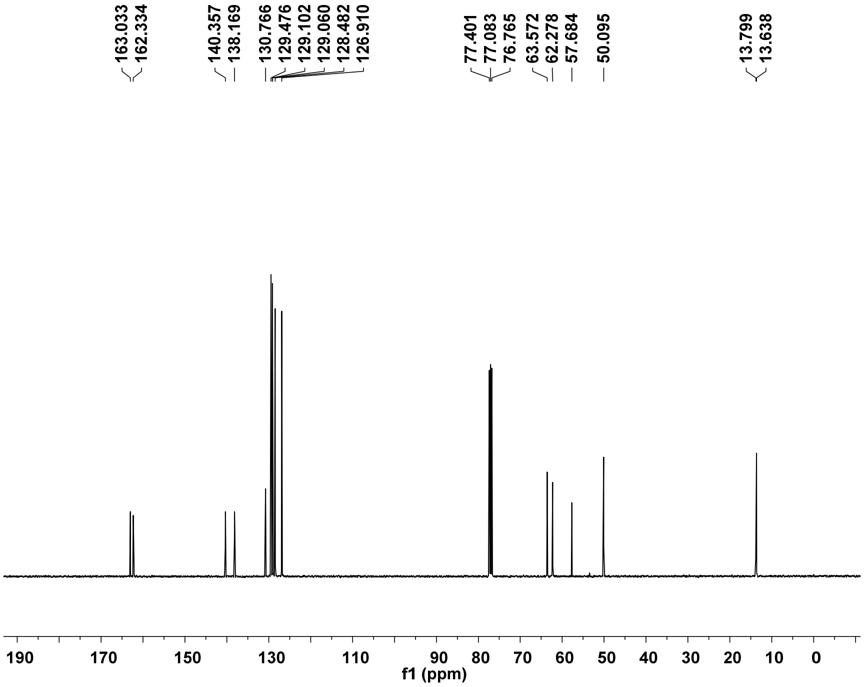

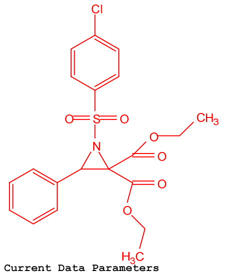

Current Data Parameters

F2 - Acquisition Parameters
DATE: 2014-11-25T23:40:47
PULPROG: zgpg30
TD: 32768
Solvent: CDCl3
NS: 1024
DS: undefined
SWH: 24038.5 Hz
AQ: undefined
TE: 296.1 C

======== CHANNEL f1 ========
NUC1: 13C
P1: 9.63 usec
SFO1: undefined MHz

F2 - Processing Parameters
SI: 65536
DC: 0.05
LB: 1.00 Hz
First Point: 0.50
FT: Hyper Quadrature
Phase: Manual
Ph0: -66.08
Ph1: 64.59

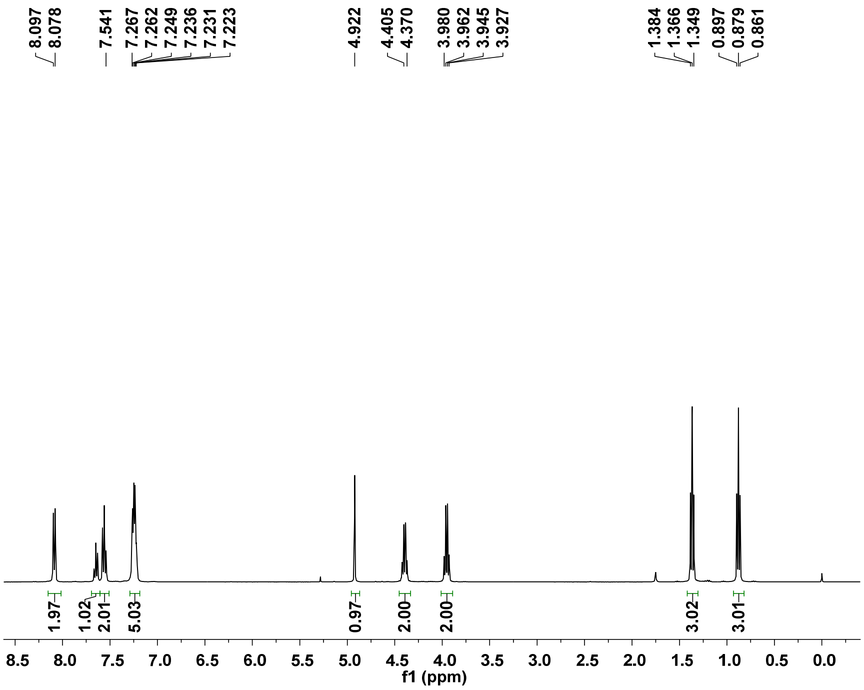

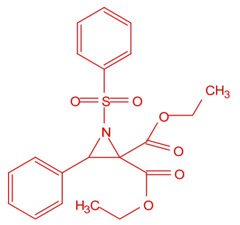

Current Data Parameters

F2 - Acquisition Parameters
DATE: 2015-01-13T22:52:55
PULPROG: zg30
TD: 32768
Solvent: CDCl3
NS: 32
DS: undefined
SWH: 8223.7 Hz
AQ: undefined
TE: 293.7 C

======== CHANNEL f1 ========
NUC1: 1H
P1: 9.93 usec
SFO1: undefined MHz

F2 - Processing Parameters
SI: 65536
DC: 0.05
LB: 0.30 Hz
First Point: 0.50
FT: Hyper Quadrature
Phase: Manual
Ph0: -261.59
Ph1: 19.60

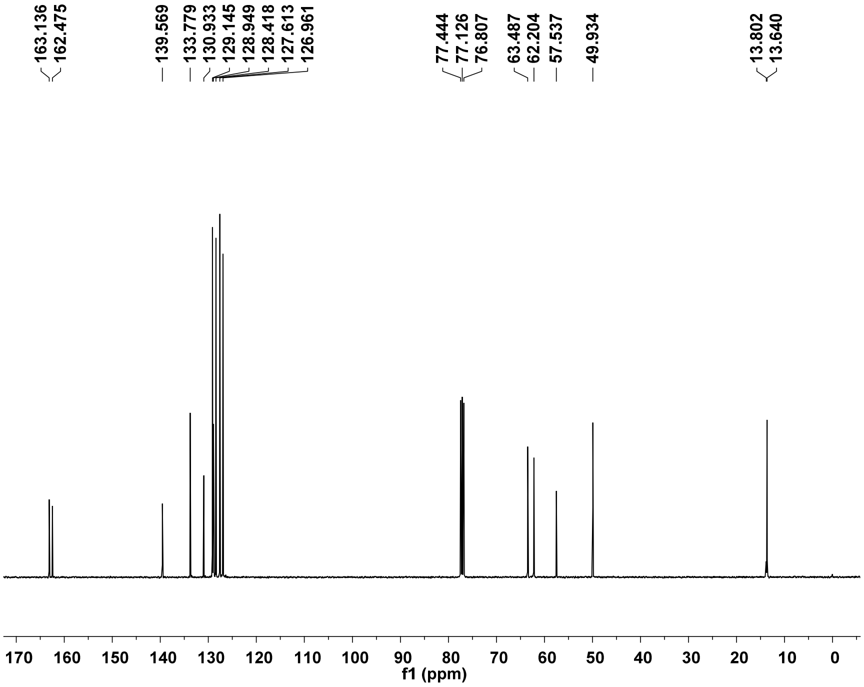

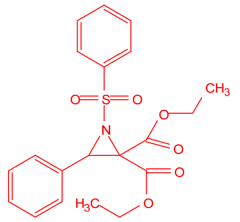

Current Data Parameters

F2 - Acquisition Parameters
DATE: 2015-01-13T23:52:21
PULPROG: zgpg30
TD: 32768
Solvent: CDCl3
NS: 1024
DS: undefined
SWH: 24038.5 Hz
AQ: undefined
TE: 294.2 C

======== CHANNEL f1 ========
NUC1: 13C
P1: 9.63 usec
SFO1: undefined MHz

F2 - Processing Parameters
SI: 65536
DC: 0.05
LB: 1.00 Hz
First Point: 0.50
FT: Hyper Quadrature
Phase: Manual
Ph0: -59.20
Ph1: 62.79

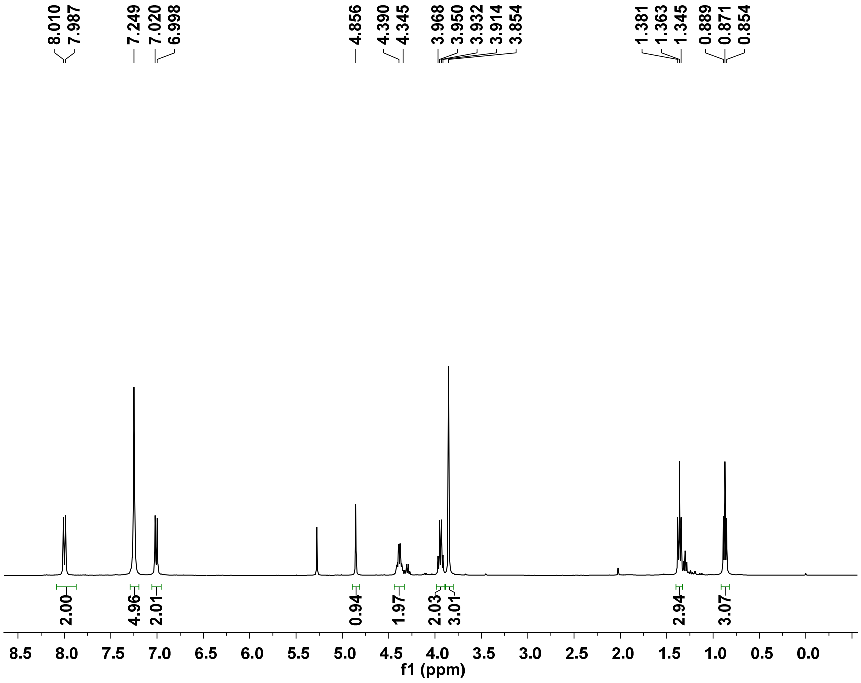

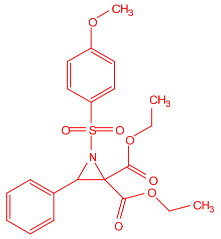

Current Data Parameters

F2 - Acquisition Parameters
DATE: 2014-11-26T22:59:36
PULPROG: zg30
TD: 32768
Solvent: CDCl3
NS: 32
DS: undefined
SWH: 8223.7 Hz
AQ: undefined
TE: 295.7 C

======== CHANNEL f1 ========
NUC1: 1H
P1: 9.93 usec
SFO1: undefined MHz

F2 - Processing Parameters
SI: 65536
DC: 0.05
LB: 0.30 Hz
First Point: 0.50
FT: Hyper Quadrature
Phase: Manual
Ph0: 89.21
Ph1: 18.90

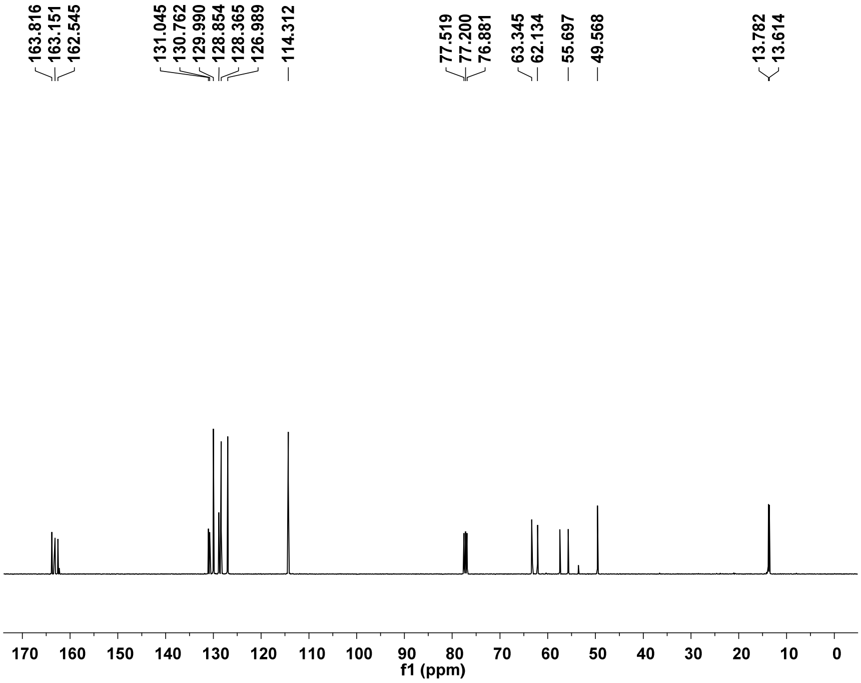

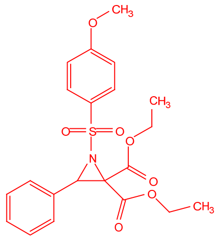

Current Data Parameters

F2 - Acquisition Parameters
DATE: 2014-11-26T23:59:02
PULPROG: zgpg30
TD: 32768
Solvent: CDCl3
NS: 1024
DS: undefined
SWH: 24038.5 Hz
AQ: undefined
TE: 296 C

======== CHANNEL f1 ========
NUC1: 13C
P1: 9.63 usec
SFO1: undefined MHz

F2 - Processing Parameters
SI: 65536
DC: 0.05
LB: 1.00 Hz
First Point: 0.50
FT: Hyper Quadrature
Phase: Manual
Ph0: -61.20
Ph1: 62.03

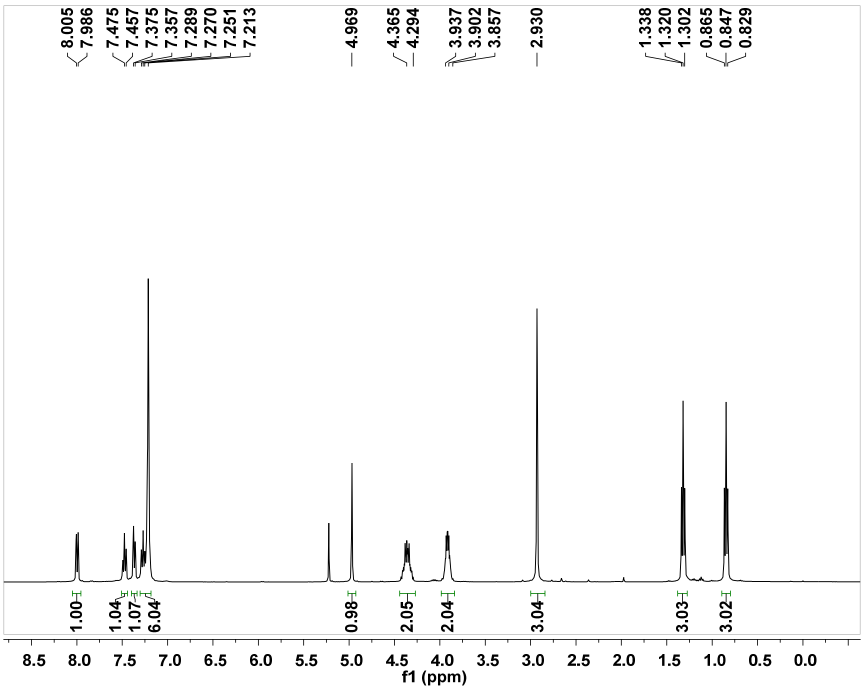

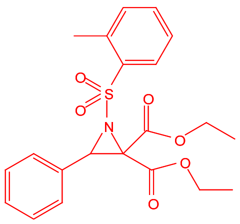

Current Data Parameters

F2 - Acquisition Parameters
DATE: 2015-05-20T03:04:32
PULPROG: zg30
TD: 32768
Solvent: CDCl3
NS: 16
DS: undefined
SWH: 8223.7 Hz
AQ: undefined
TE: 295.9 C

======== CHANNEL f1 ========
NUC1: 1H
P1: 9.93 usec
SFO1: undefined MHz

F2 - Processing Parameters
SI: 65536
DC: 0.05
LB: 0.30 Hz
First Point: 0.50
FT: Hyper Quadrature
Phase: Manual
Ph0: 99.20
Ph1: 11.99

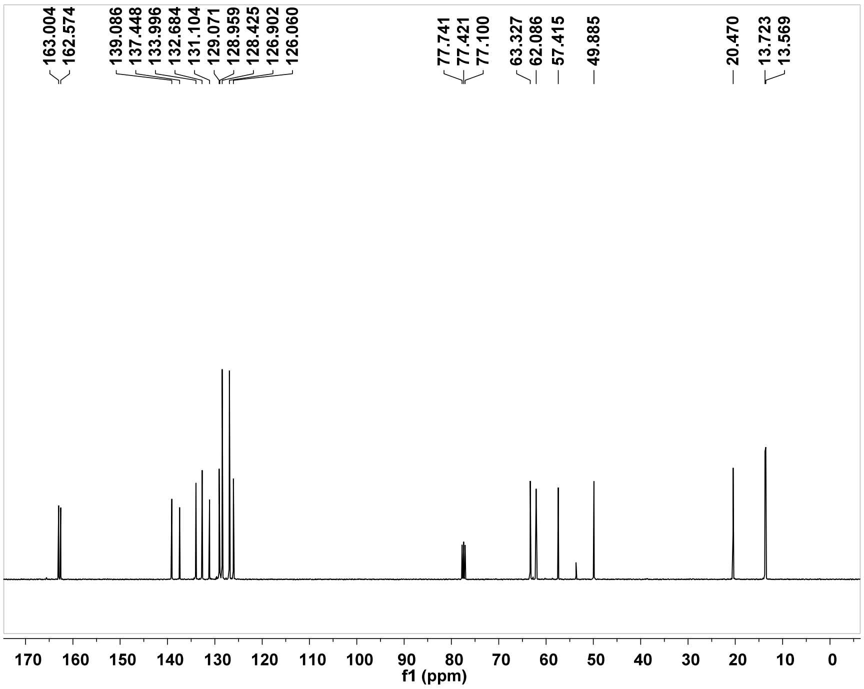

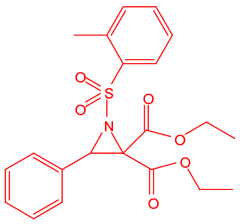

Current Data Parameters

F2 - Acquisition Parameters
DATE: 2015-05-20T03:20:09
PULPROG: zgpg30
TD: 32768
Solvent: CDCl3
NS: 256
DS: undefined
SWH: 24038.5 Hz
AQ: undefined
TE: 296.2 C

======== CHANNEL f1 ========
NUC1: 13C
P1: 9.63 usec
SFO1: undefined MHz

F2 - Processing Parameters
SI: 65536
DC: 0.05
LB: 1.00 Hz
First Point: 0.50
FT: Hyper Quadrature
Phase: Manual
Ph0: -60.96
Ph1: 60.81

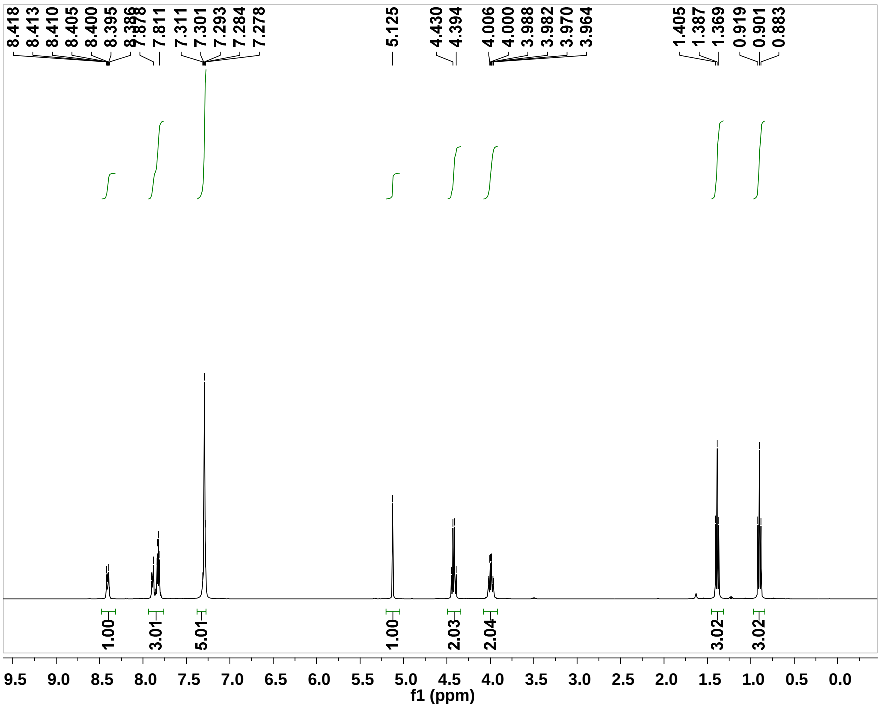

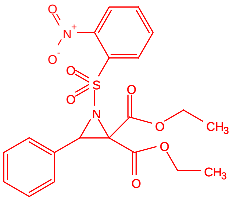

Current Data Parameters

F2 - Acquisition Parameters
DATE: 2016-01-28T09:52:53
PULPROG: zg30
TD: 32768
Solvent: CDCl3
NS: 32
DS: undefined
SWH: 8223.7 Hz
AQ: undefined
TE: 0 C

======== CHANNEL f1 ========
NUC1: 1H
P1: 9.93 usec
SFO1: undefined MHz

F2 - Processing Parameters
SI: 65536
DC: 0.05
LB: 0.30 Hz
First Point: 0.50
FT: Hyper Quadrature
Phase: Manual
Ph0: 93.23
Ph1: 19.75

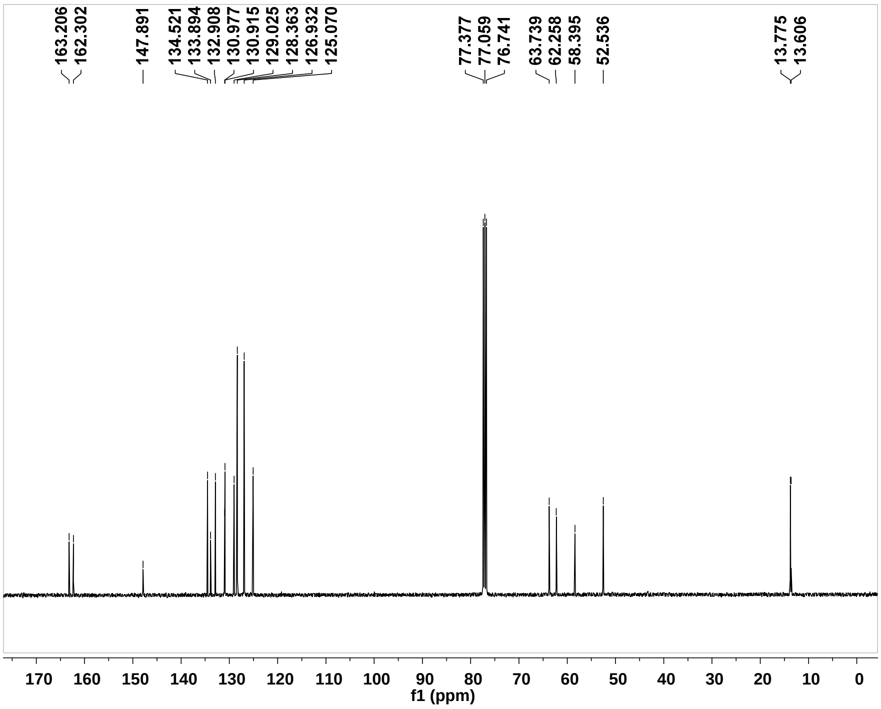

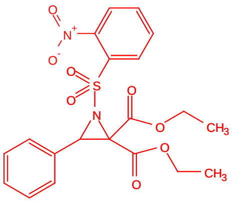

Current Data Parameters

F2 - Acquisition Parameters
DATE: 2016-01-28T10:52:19
PULPROG: zgpg30
TD: 32768
Solvent: CDCl3
NS: 1024
DS: undefined
SWH: 24038.5 Hz
AQ: undefined
TE: 0 C

======== CHANNEL f1 ========
NUC1: 13C
P1: 9.63 usec
SFO1: undefined MHz

F2 - Processing Parameters
SI: 65536
DC: 0.05
LB: 1.00 Hz
First Point: 0.50
FT: Hyper Quadrature
Phase: Manual
Ph0: -71.63
Ph1: 67.11

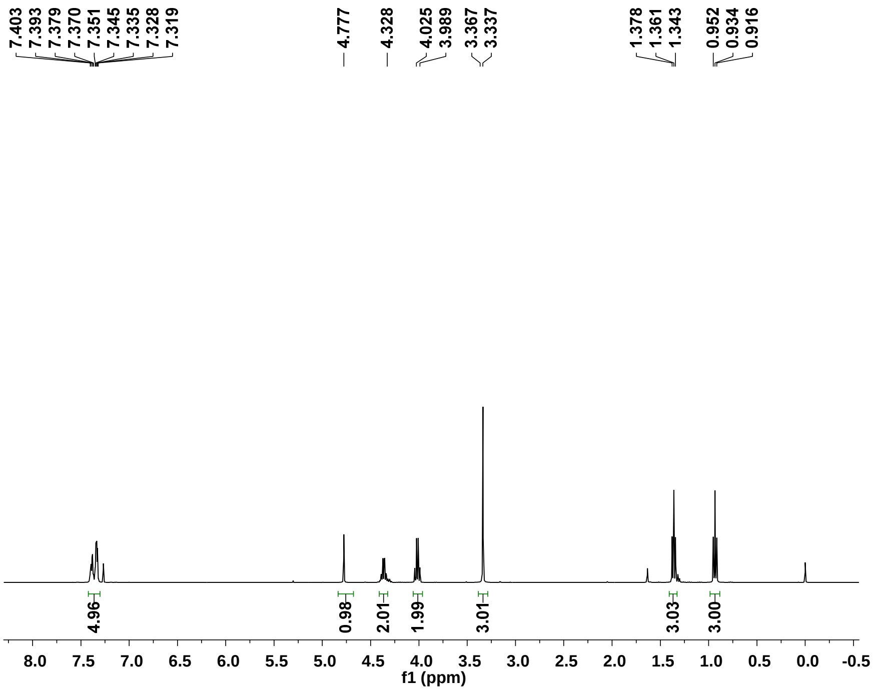

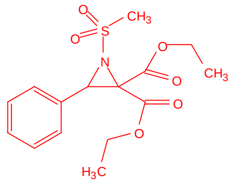

Current Data Parameters

F2 - Acquisition Parameters
DATE: 2015-01-01T15:08:53
PULPROG: zg30
TD: 32768
Solvent: CDCl3
NS: 32
DS: undefined
SWH: 8223.7 Hz
AQ: undefined
TE: 291.8 C

======== CHANNEL f1 ========
NUC1: 1H
P1: 9.93 usec
SFO1: undefined MHz

F2 - Processing Parameters
SI: 65536
DC: 0.05
LB: 0.30 Hz
First Point: 0.50
FT: Hyper Quadrature
Phase: Manual
Ph0: 96.89
Ph1: 20.97

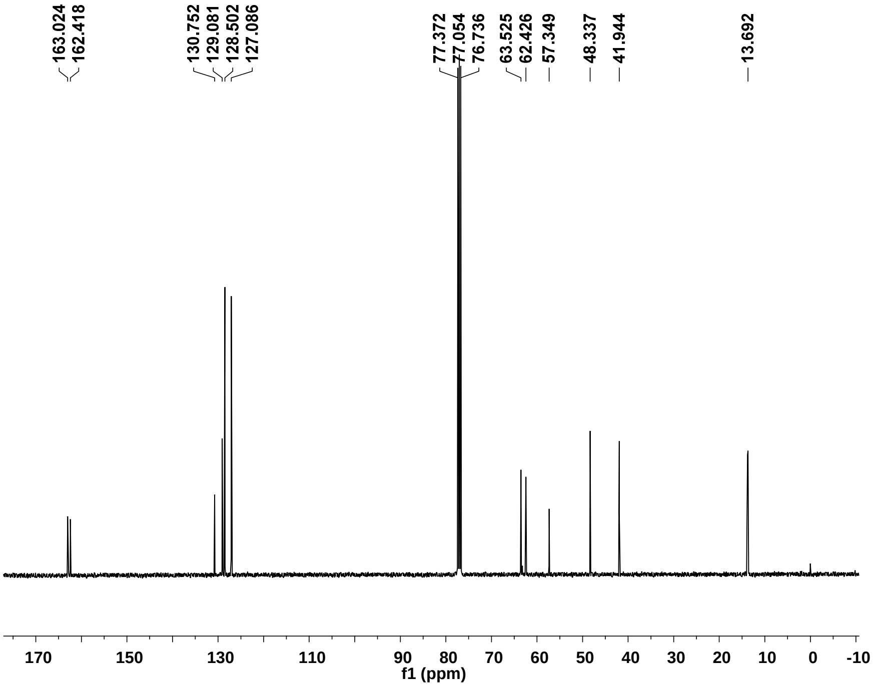

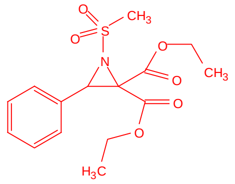

Current Data Parameters

F2 - Acquisition Parameters
DATE: 2015-01-01T16:08:18
PULPROG: zgpg30
TD: 32768
Solvent: CDCl3
NS: 1024
DS: undefined
SWH: 24038.5 Hz
AQ: undefined
TE: 292.1 C

======== CHANNEL f1 ========
NUC1: 13C
P1: 9.63 usec
SFO1: undefined MHz

F2 - Processing Parameters
SI: 65536
DC: 0.05
LB: 1.00 Hz
First Point: 0.50
FT: Hyper Quadrature
Phase: Manual
Ph0: -62.47
Ph1: 56.90

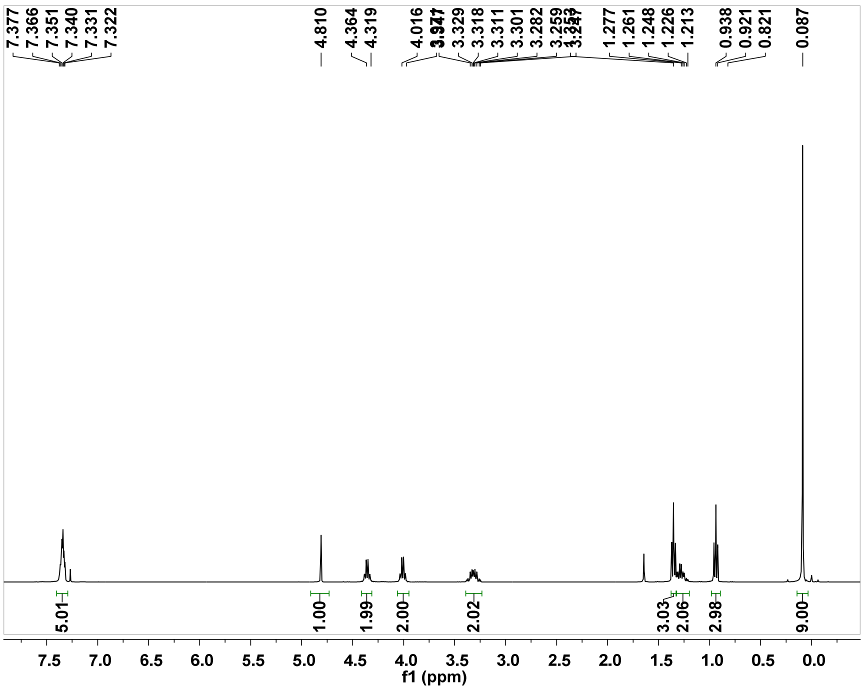

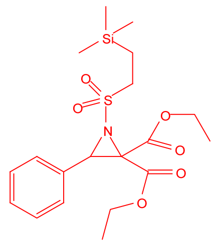

Current Data Parameters

F2 - Acquisition Parameters
DATE: 2015-05-25T10:30:48
PULPROG: zg30
TD: 32768
Solvent: CDCl3
NS: 32
DS: undefined
SWH: 8223.7 Hz
AQ: undefined
TE: 295 C

======== CHANNEL f1 ========
NUC1: 1H
P1: 9.93 usec
SFO1: undefined MHz

F2 - Processing Parameters
SI: 65536
DC: 0.05
LB: 0.30 Hz
First Point: 0.50
FT: Hyper Quadrature
Phase: Manual
Ph0: -260.73
Ph1: 20.45

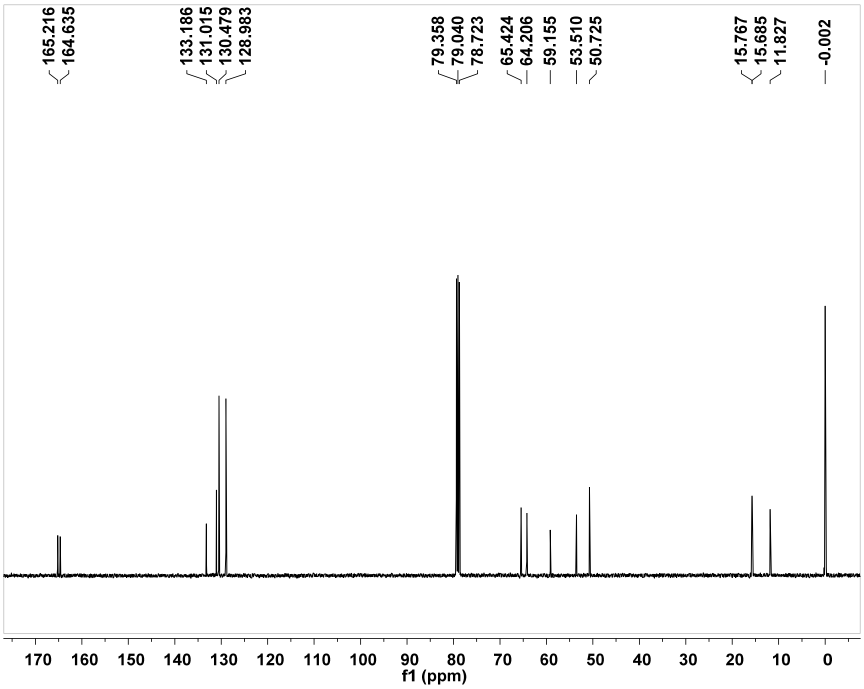

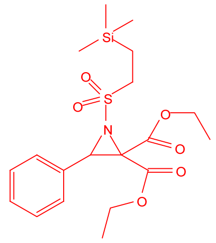

Current Data Parameters

F2 - Acquisition Parameters
DATE: 2015-05-25T11:01:00
PULPROG: zgpg30
TD: 32768
Solvent: CDCl3
NS: 512
DS: undefined
SWH: 24038.5 Hz
AQ: undefined
TE: 295.4 C

======== CHANNEL f1 ========
NUC1: 13C
P1: 9.63 usec
SFO1: undefined MHz

F2 - Processing Parameters
SI: 65536
DC: 0.05
LB: 1.00 Hz
First Point: 0.50
FT: Hyper Quadrature
Phase: Manual
Ph0: -51.97
Ph1: 52.28

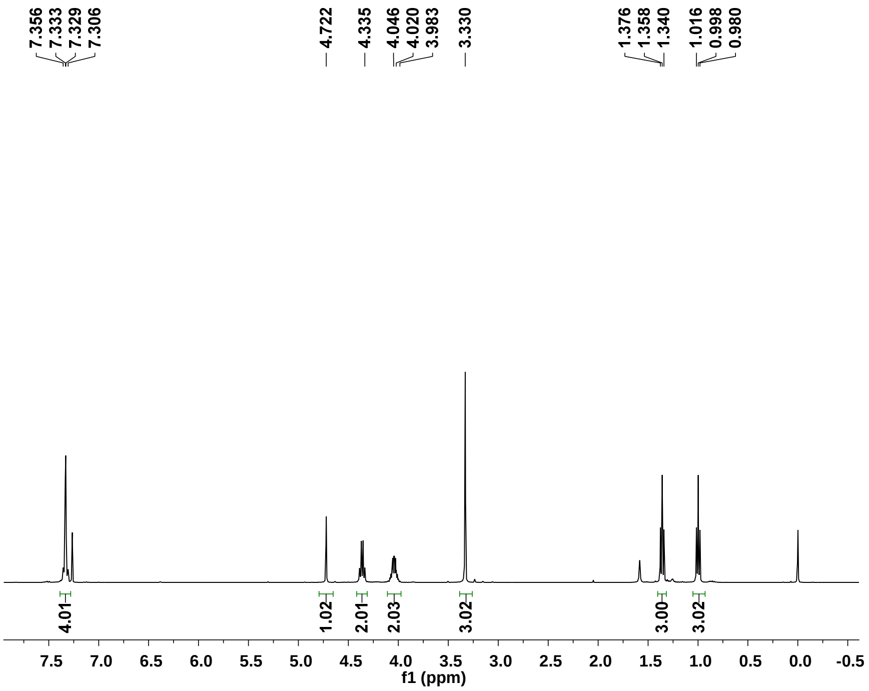

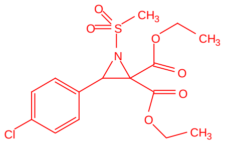

Current Data Parameters

F2 - Acquisition Parameters
DATE: 2015-01-20T09:32:12
PULPROG: zg30
TD: 32768
Solvent: CDCl3
NS: 32
DS: undefined
SWH: 8223.7 Hz
AQ: undefined
TE: 294.3 C

======== CHANNEL f1 ========
NUC1: 1H
P1: 9.93 usec
SFO1: undefined MHz

F2 - Processing Parameters
SI: 65536
DC: 0.05
LB: 0.30 Hz
First Point: 0.50
FT: Hyper Quadrature
Phase: Manual
Ph0: 92.75
Ph1: 18.93

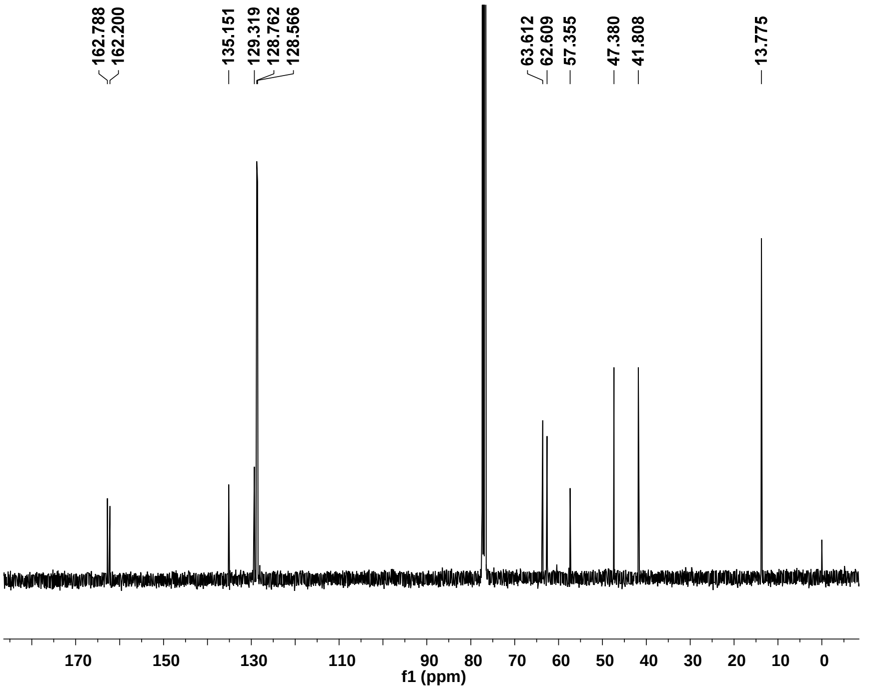

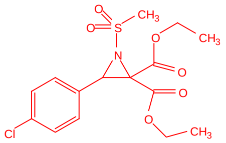

Current Data Parameters

F2 - Acquisition Parameters
DATE: 2015-01-20T10:31:38
PULPROG: zgpg30
TD: 32768
Solvent: CDCl3
NS: 1024
DS: undefined
SWH: 24038.5 Hz
AQ: undefined
TE: 294.6 C

======== CHANNEL f1 ========
NUC1: 13C
P1: 9.63 usec
SFO1: undefined MHz

F2 - Processing Parameters
SI: 65536
DC: 0.05
LB: 1.00 Hz
First Point: 0.50
FT: Hyper Quadrature
Phase: Manual
Ph0: -53.06
Ph1: 42.62

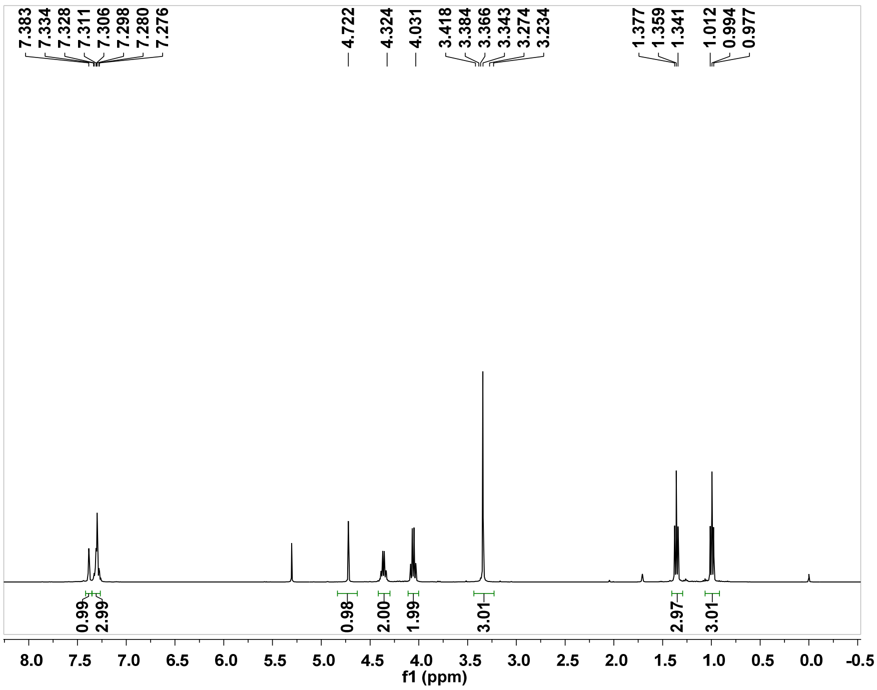

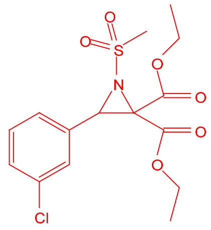

Current Data Parameters

F2 - Acquisition Parameters
DATE: 2015-03-24T22:53:44
PULPROG: zg30
TD: 32768
Solvent: CDCl3
NS: 32
DS: undefined
SWH: 8223.7 Hz
AQ: undefined
TE: 294.2 C

======== CHANNEL f1 ========
NUC1: 1H
P1: 9.93 usec
SFO1: undefined MHz

F2 - Processing Parameters
SI: 65536
DC: 0.05
LB: 0.30 Hz
First Point: 0.50
FT: Hyper Quadrature
Phase: Manual
Ph0: -259.84
Ph1: 16.78

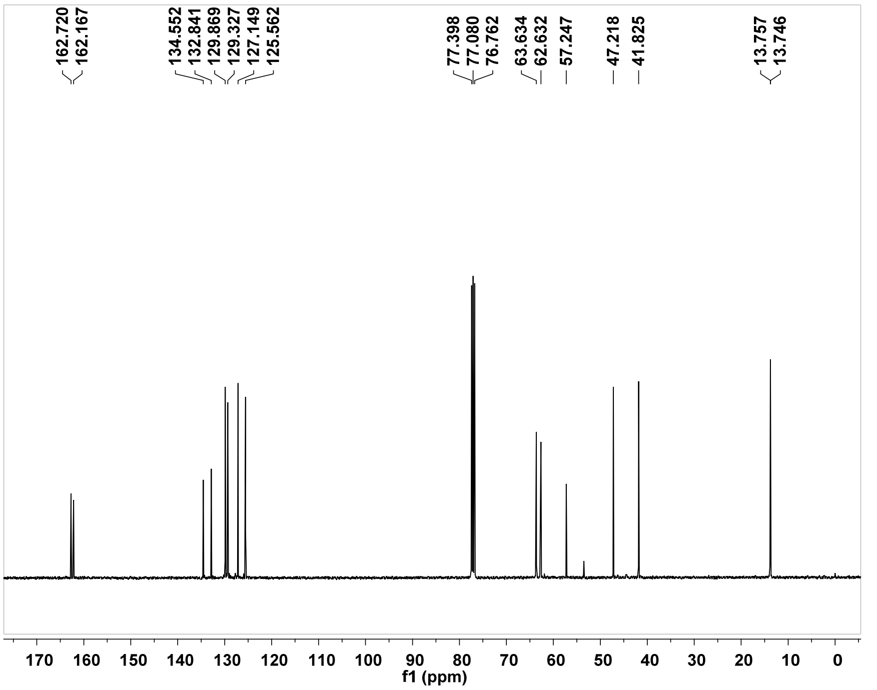

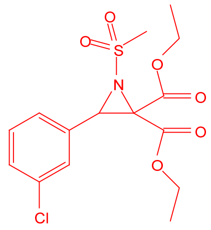

Current Data Parameters

F2 - Acquisition Parameters
DATE: 2015-03-24T23:53:10
PULPROG: zgpg30
TD: 32768
Solvent: CDCl3
NS: 1024
DS: undefined
SWH: 24038.5 Hz
AQ: undefined
TE: 294.6 C

======== CHANNEL f1 ========
NUC1: 13C
P1: 9.63 usec
SFO1: undefined MHz

F2 - Processing Parameters
SI: 65536
DC: 0.05
LB: 1.00 Hz
First Point: 0.50
FT: Hyper Quadrature
Phase: Manual
Ph0: -61.73
Ph1: 65.20

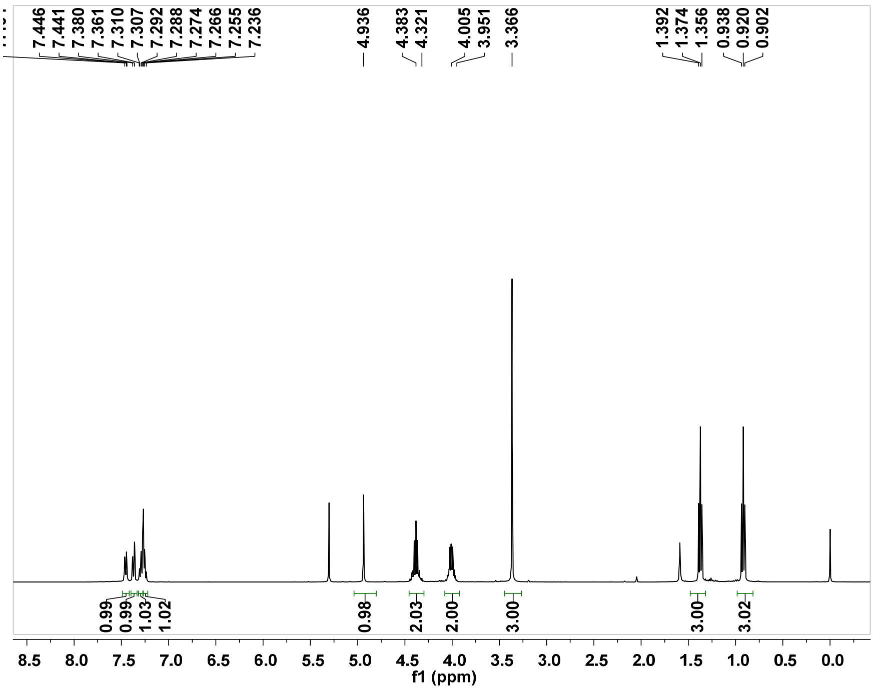

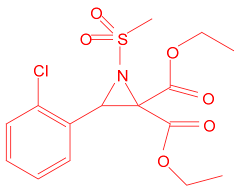

Current Data Parameters

F2 - Acquisition Parameters
DATE: 2015-04-20T23:35:05
PULPROG: zg30
TD: 32768
Solvent: CDCl3
NS: 32
DS: undefined
SWH: 8223.7 Hz
AQ: undefined
TE: 295 C

======== CHANNEL f1 ========
NUC1: 1H
P1: 9.93 usec
SFO1: undefined MHz

F2 - Processing Parameters
SI: 65536
DC: 0.05
LB: 0.30 Hz
First Point: 0.50
FT: Hyper Quadrature
Phase: Manual
Ph0: 94.27
Ph1: 14.62

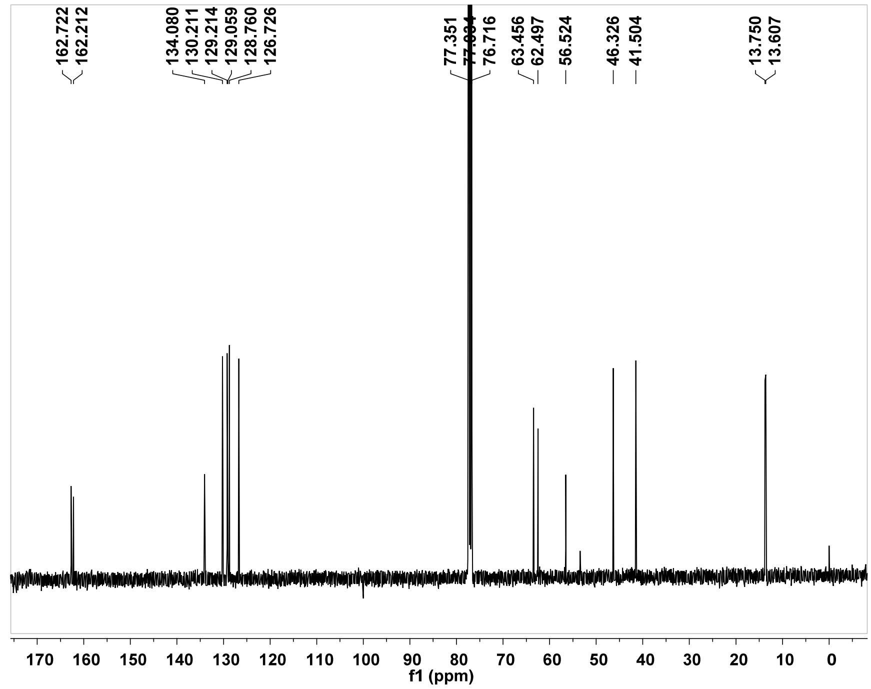

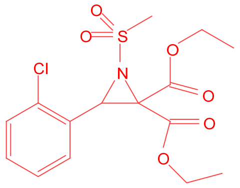

Current Data Parameters

F2 - Acquisition Parameters
DATE: 2015-04-21T00:34:33
PULPROG: zgpg30
TD: 32768
Solvent: CDCl3
NS: 1024
DS: undefined
SWH: 24038.5 Hz
AQ: undefined
TE: 295.4 C

======== CHANNEL f1 ========
NUC1: 13C
P1: 9.63 usec
SFO1: undefined MHz

F2 - Processing Parameters
SI: 65536
DC: 0.05
LB: 1.00 Hz
First Point: 0.50
FT: Hyper Quadrature
Phase: Manual
Ph0: -63.40
Ph1: 67.49

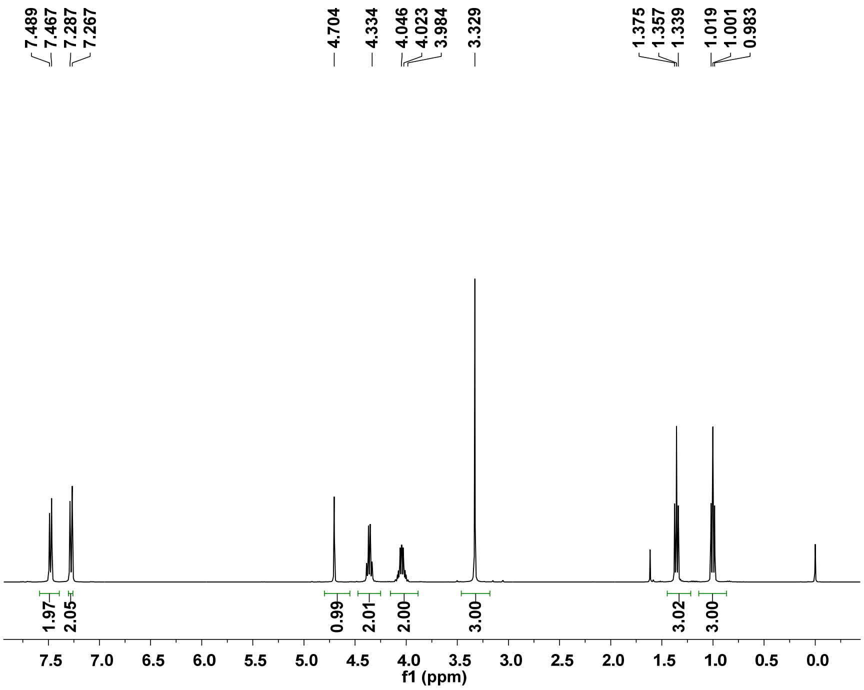

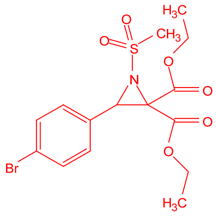

Current Data Parameters

F2 - Acquisition Parameters
DATE: 2015-01-26T21:04:54
PULPROG: zg30
TD: 32768
Solvent: CDCl3
NS: 32
DS: undefined
SWH: 8223.7 Hz
AQ: undefined
TE: 293.9 C

======== CHANNEL f1 ========
NUC1: 1H
P1: 9.93 usec
SFO1: undefined MHz

F2 - Processing Parameters
SI: 65536
DC: 0.05
LB: 0.30 Hz
First Point: 0.50
FT: Hyper Quadrature
Phase: Manual
Ph0: 94.08
Ph1: 20.48

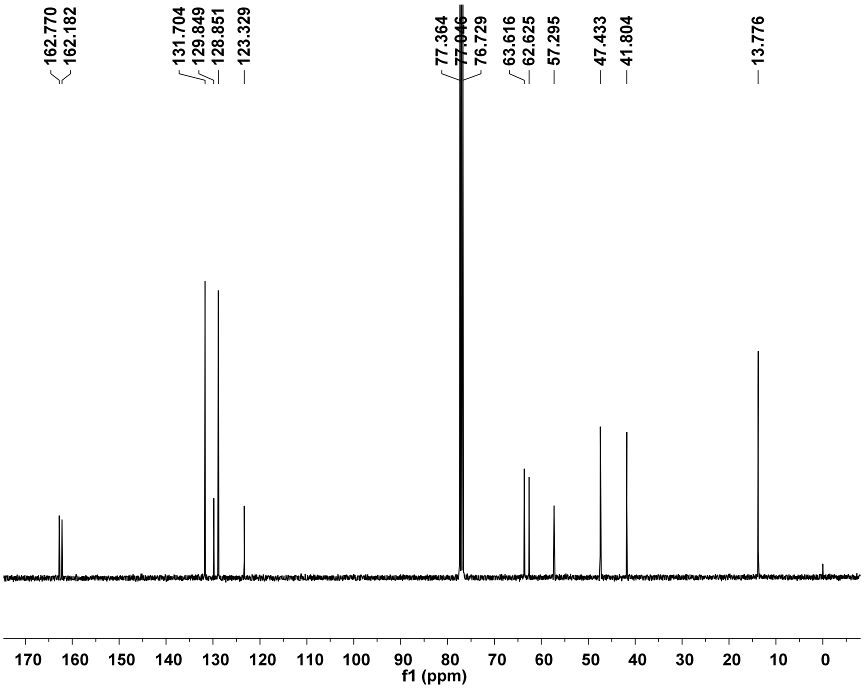

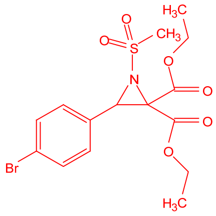

Current Data Parameters

F2 - Acquisition Parameters
DATE: 2015-01-26T22:04:20
PULPROG: zgpg30
TD: 32768
Solvent: CDCl3
NS: 1024
DS: undefined
SWH: 24038.5 Hz
AQ: undefined
TE: 294.3 C

======== CHANNEL f1 ========
NUC1: 13C
P1: 9.63 usec
SFO1: undefined MHz

F2 - Processing Parameters
SI: 65536
DC: 0.05
LB: 1.00 Hz
First Point: 0.50
FT: Hyper Quadrature
Phase: Manual
Ph0: -57.16
Ph1: 50.96

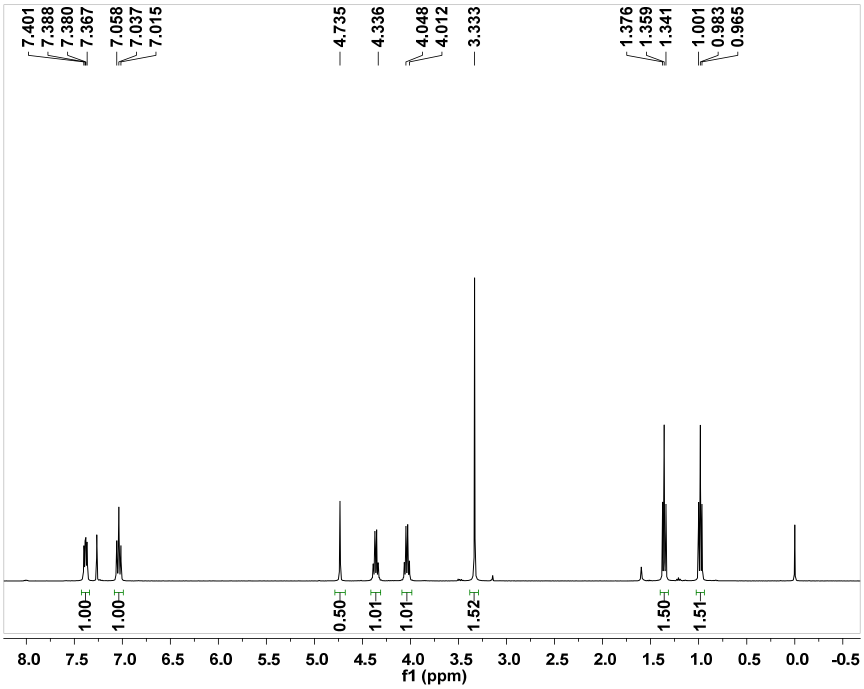

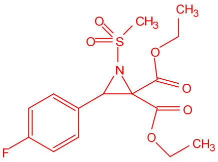

Current Data Parameters

F2 - Acquisition Parameters
DATE: 2015-02-07T16:16:32
PULPROG: zg30
TD: 32768
Solvent: CDCl3
NS: 16
DS: undefined
SWH: 8223.7 Hz
AQ: undefined
TE: 293.5 C

======== CHANNEL f1 ========
NUC1: 1H
P1: 9.93 usec
SFO1: undefined MHz

F2 - Processing Parameters
SI: 65536
DC: 0.05
LB: 0.30 Hz
First Point: 0.50
FT: Hyper Quadrature
Phase: Manual
Ph0: 94.07
Ph1: 19.67

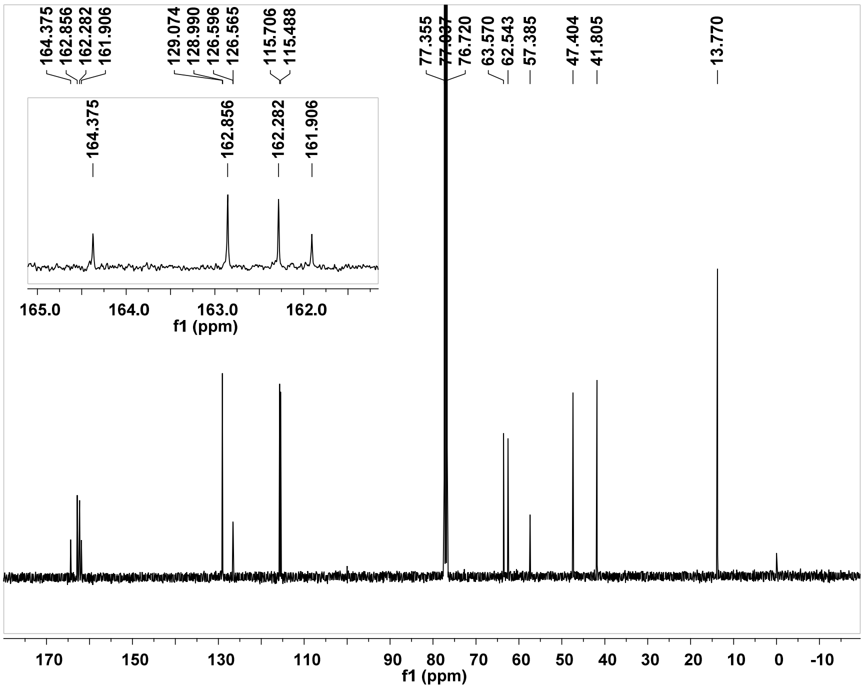

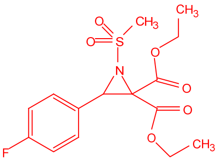

Current Data Parameters

F2 - Acquisition Parameters
DATE: 2015-02-07T19:57:00
PULPROG: zgpg30
TD: 32768
Solvent: CDCl3
NS: 1024
DS: undefined
SWH: 24038.5 Hz
AQ: undefined
TE: 294.1 C

======== CHANNEL f1 ========
NUC1: 13C
P1: 9.63 usec
SFO1: undefined MHz

F2 - Processing Parameters
SI: 65536
DC: 0.05
LB: 1.00 Hz
First Point: 0.50
FT: Hyper Quadrature
Phase: Manual
Ph0: -62.45
Ph1: 56.12

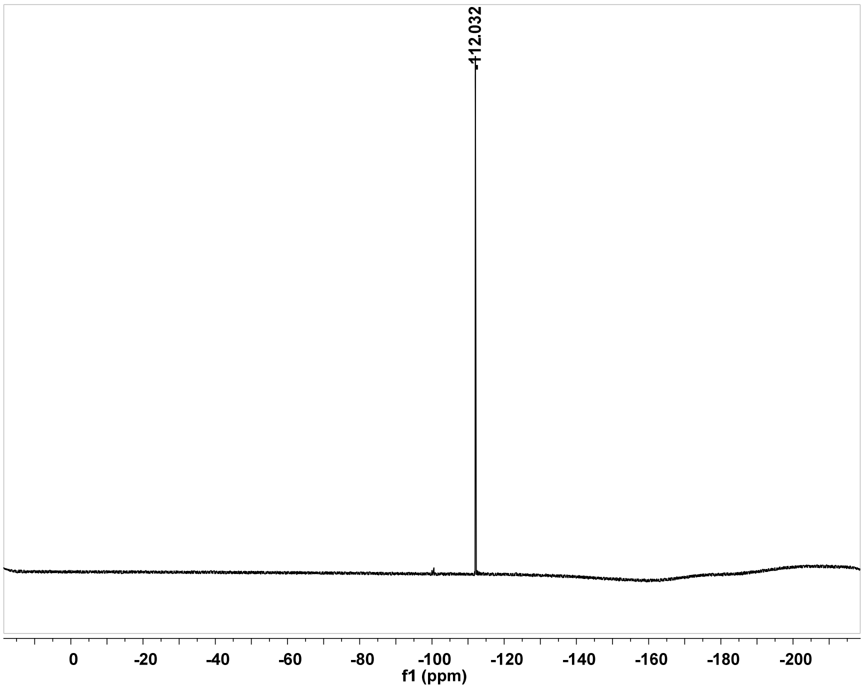

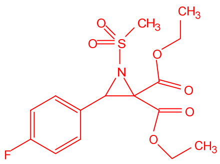

Current Data Parameters

F2 - Acquisition Parameters
DATE: 2015-02-07T19:58:31
PULPROG: zgflqn
TD: 65536
Solvent: CDCl3
NS: 16
DS: undefined
SWH: 89285.7 Hz
AQ: undefined
TE: 293.9 C

======== CHANNEL f1 ========
NUC1: 19F
P1: 12.75 usec
SFO1: undefined MHz

F2 - Processing Parameters
SI: 131072
DC: 0.05
LB: 0.30 Hz
First Point: 0.50
FT: Hyper Quadrature
Phase: Manual
Ph0: 4.39
Ph1: -65.19

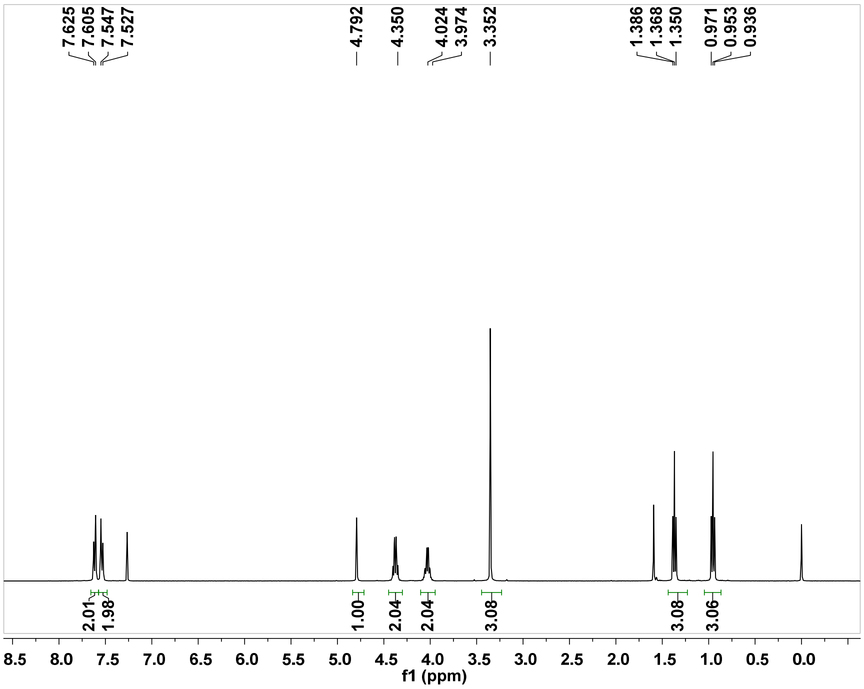

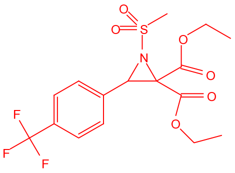

Current Data Parameters

F2 - Acquisition Parameters
DATE: 2015-03-10T22:19:49
PULPROG: zg30
TD: 32768
Solvent: CDCl3
NS: 16
DS: undefined
SWH: 8223.7 Hz
AQ: undefined
TE: 293.4 C

======== CHANNEL f1 ========
NUC1: 1H
P1: 9.93 usec
SFO1: undefined MHz

F2 - Processing Parameters
SI: 65536
DC: 0.05
LB: 0.30 Hz
First Point: 0.50
FT: Hyper Quadrature
Phase: Manual
Ph0: 96.50
Ph1: 19.76

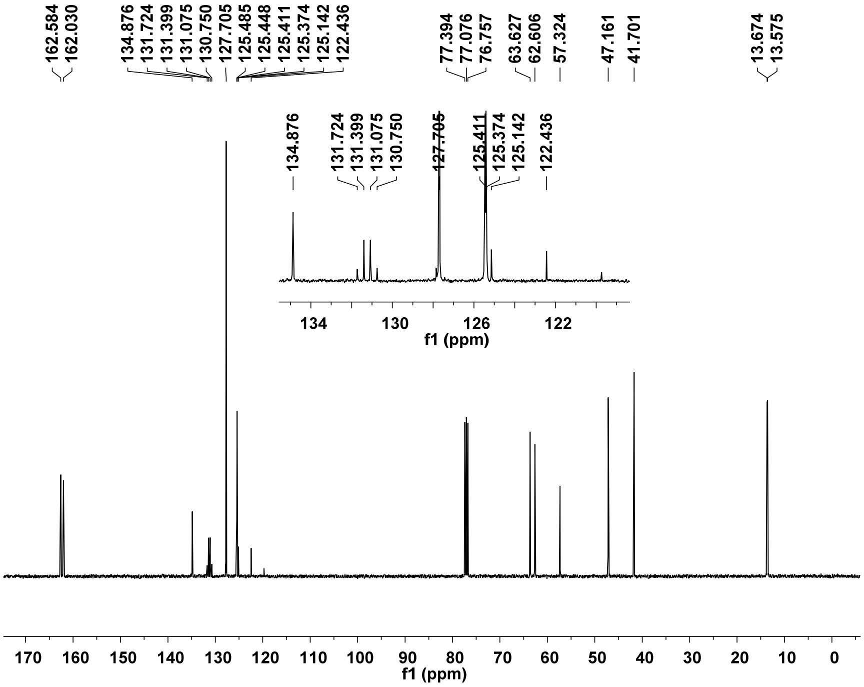

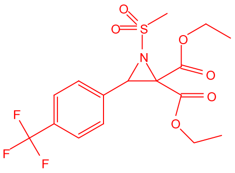

Current Data Parameters

F2 - Acquisition Parameters
DATE: 2015-08-22T16:05:11
PULPROG: zgpg30
TD: 32768
Solvent: CDCl3
NS: 256
DS: undefined
SWH: 24038.5 Hz
AQ: undefined
TE: 0 C

======== CHANNEL f1 ========
NUC1: 13C
P1: 9.63 usec
SFO1: undefined MHz

F2 - Processing Parameters
SI: 65536
DC: 0.05
LB: 1.00 Hz
First Point: 0.50
FT: Hyper Quadrature
Phase: Manual
Ph0: -74.22
Ph1: 64.15

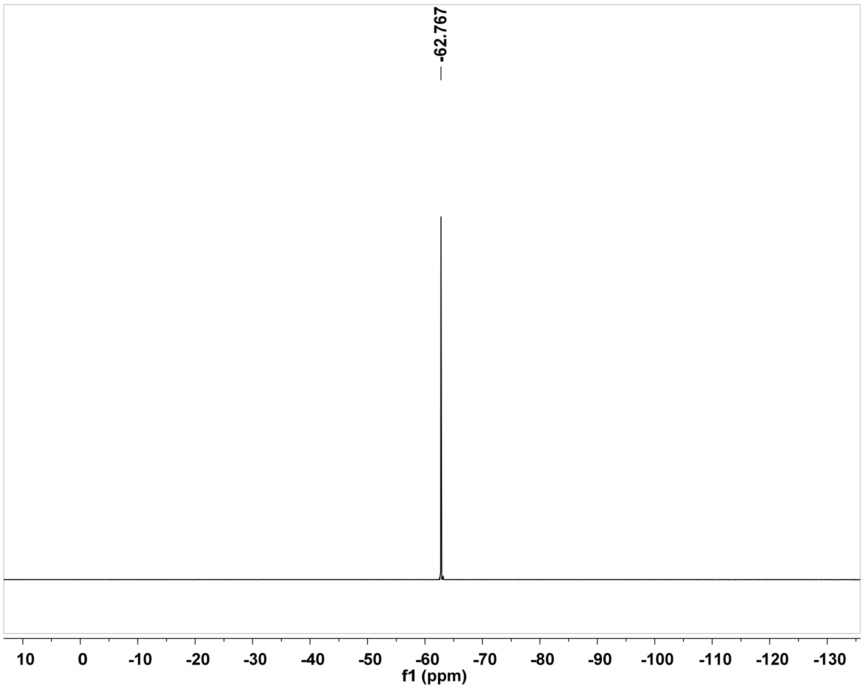

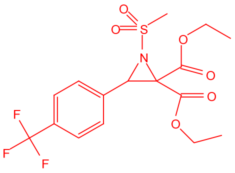

Current Data Parameters

F2 - Acquisition Parameters
DATE: 2015-03-16T17:16:11
PULPROG: zgflqn
TD: 65536
Solvent: CDCl3
NS: 16
DS: undefined
SWH: 89285.7 Hz
AQ: undefined
TE: 293.8 C

======== CHANNEL f1 ========
NUC1: 19F
P1: 12.75 usec
SFO1: undefined MHz

F2 - Processing Parameters
SI: 131072
DC: 0.05
LB: 0.30 Hz
First Point: 0.50
FT: Hyper Quadrature
Phase: Manual
Ph0: -121.85
Ph1: 0.00

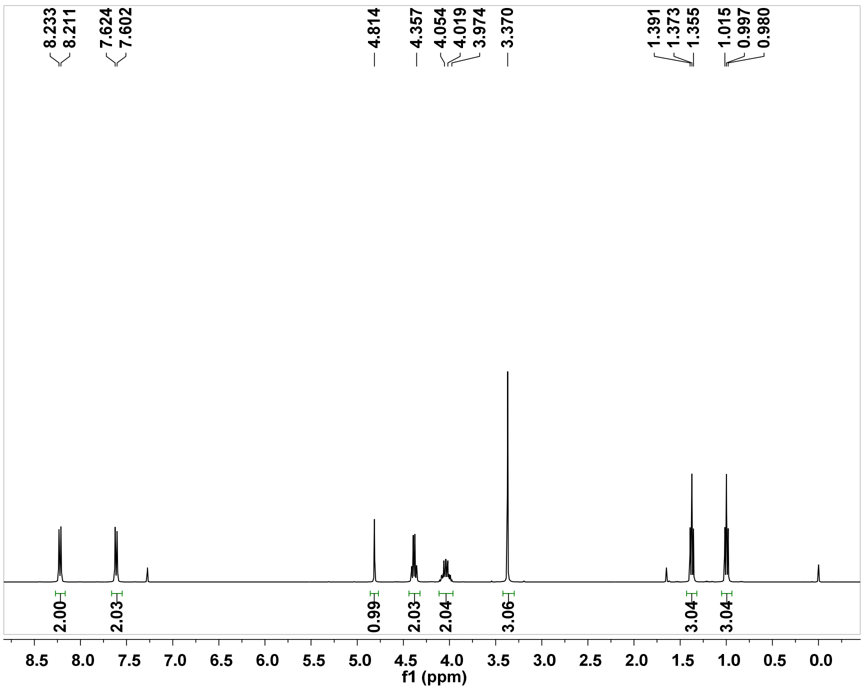

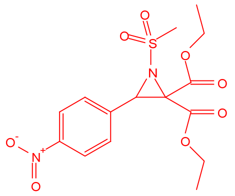

Current Data Parameters

F2 - Acquisition Parameters
DATE: 2015-03-06T00:06:36
PULPROG: zg30
TD: 32768
Solvent: CDCl3
NS: 32
DS: undefined
SWH: 8223.7 Hz
AQ: undefined
TE: 293.5 C

======== CHANNEL f1 ========
NUC1: 1H
P1: 9.93 usec
SFO1: undefined MHz

F2 - Processing Parameters
SI: 65536
DC: 0.05
LB: 0.30 Hz
First Point: 0.50
FT: Hyper Quadrature
Phase: Manual
Ph0: 97.03
Ph1: 20.90

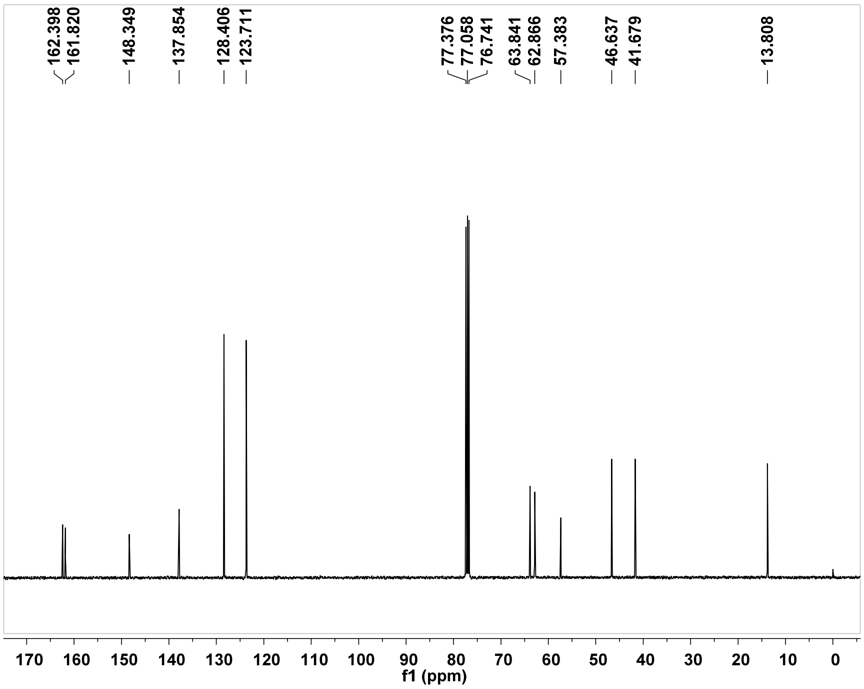

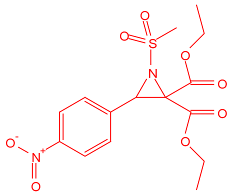

Current Data Parameters

F2 - Acquisition Parameters
DATE: 2015-03-06T01:06:02
PULPROG: zgpg30
TD: 32768
Solvent: CDCl3
NS: 1024
DS: undefined
SWH: 24038.5 Hz
AQ: undefined
TE: 293.9 C

======== CHANNEL f1 ========
NUC1: 13C
P1: 9.63 usec
SFO1: undefined MHz

F2 - Processing Parameters
SI: 65536
DC: 0.05
LB: 1.00 Hz
First Point: 0.50
FT: Hyper Quadrature
Phase: Manual
Ph0: -62.57
Ph1: 68.66

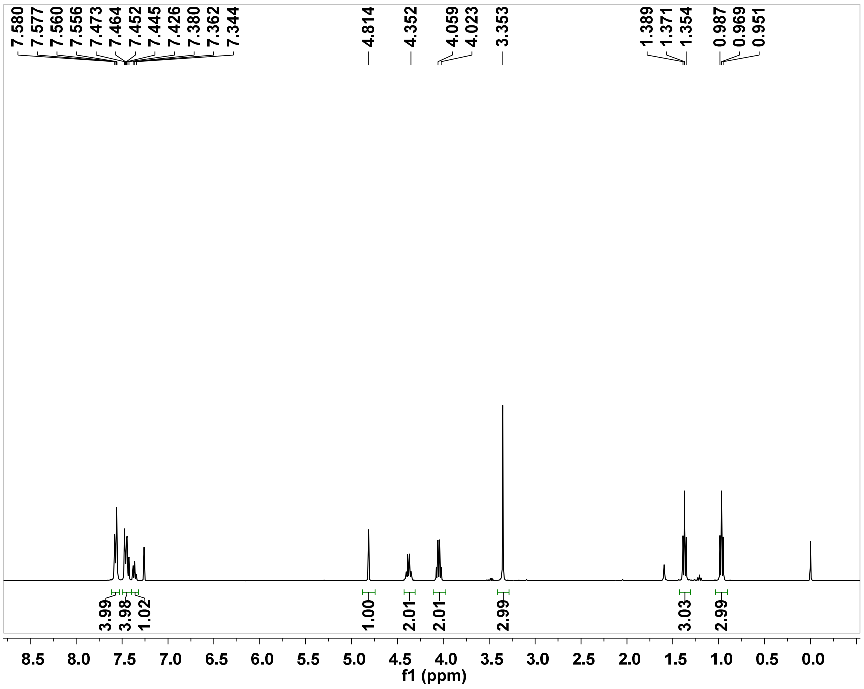

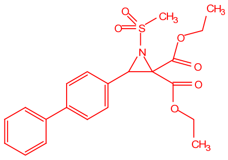

Current Data Parameters

F2 - Acquisition Parameters
DATE: 2015-02-10T19:56:17
PULPROG: zg30
TD: 32768
Solvent: CDCl3
NS: 32
DS: undefined
SWH: 8223.7 Hz
AQ: undefined
TE: 293.6 C

======== CHANNEL f1 ========
NUC1: 1H
P1: 9.93 usec
SFO1: undefined MHz

F2 - Processing Parameters
SI: 65536
DC: 0.05
LB: 0.30 Hz
First Point: 0.50
FT: Hyper Quadrature
Phase: Manual
Ph0: 96.62
Ph1: 19.44

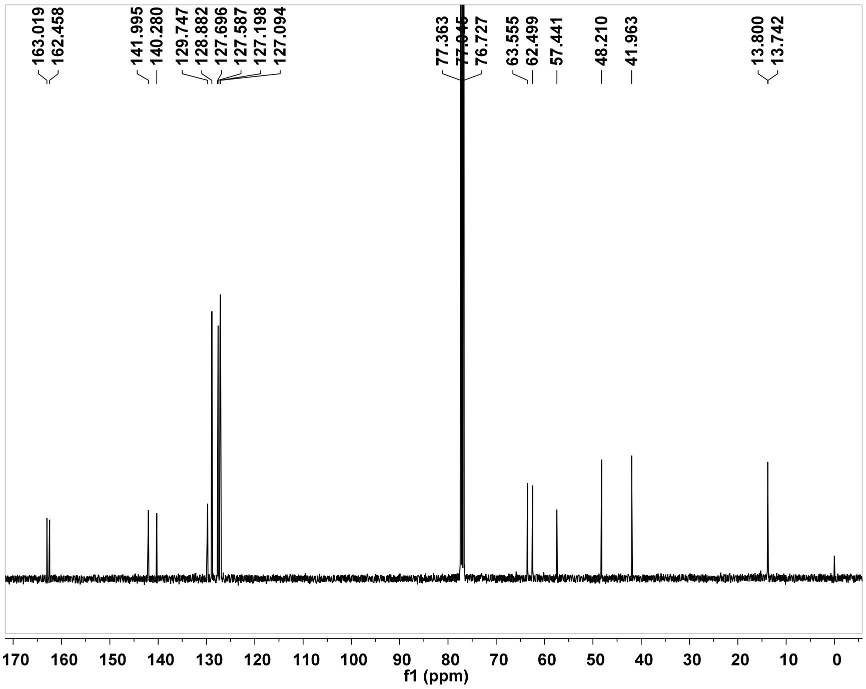

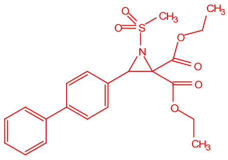

Current Data Parameters

F2 - Acquisition Parameters
DATE: 2015-02-10T20:55:43
PULPROG: zgpg30
TD: 32768
Solvent: CDCl3
NS: 1024
DS: undefined
SWH: 24038.5 Hz
AQ: undefined
TE: 293.8 C

======== CHANNEL f1 ========
NUC1: 13C
P1: 9.63 usec
SFO1: undefined MHz

F2 - Processing Parameters
SI: 65536
DC: 0.05
LB: 1.00 Hz
First Point: 0.50
FT: Hyper Quadrature
Phase: Manual
Ph0: -61.00
Ph1: 67.81

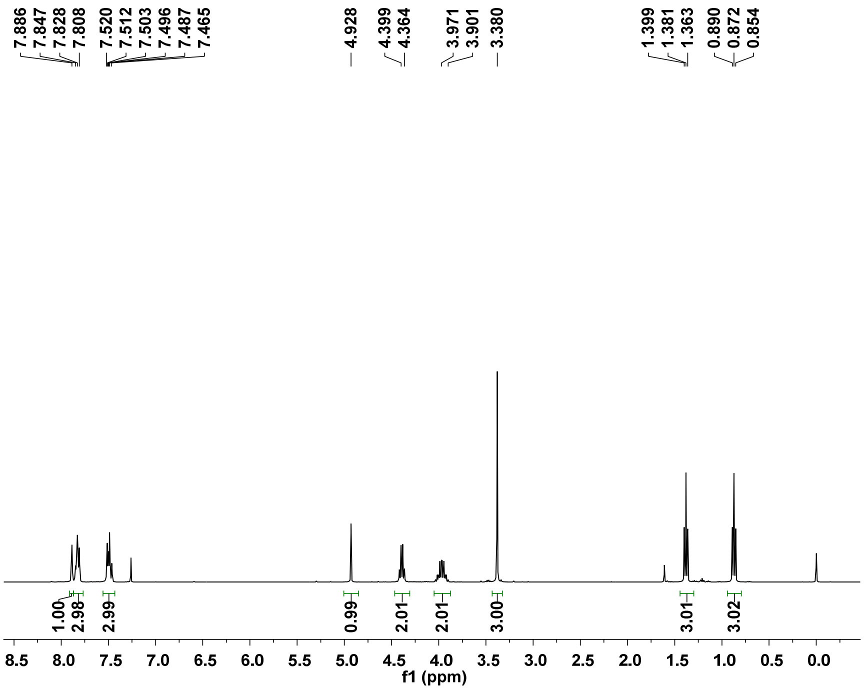

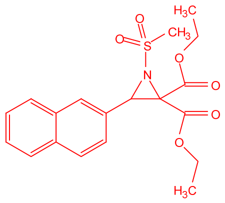

Current Data Parameters

F2 - Acquisition Parameters
DATE: 2015-01-26T20:00:06
PULPROG: zg30
TD: 32768
Solvent: CDCl3
NS: 32
DS: undefined
SWH: 8223.7 Hz
AQ: undefined
TE: 293.8 C

======== CHANNEL f1 ========
NUC1: 1H
P1: 9.93 usec
SFO1: undefined MHz

F2 - Processing Parameters
SI: 65536
DC: 0.05
LB: 0.30 Hz
First Point: 0.50
FT: Hyper Quadrature
Phase: Manual
Ph0: 94.31
Ph1: 19.22

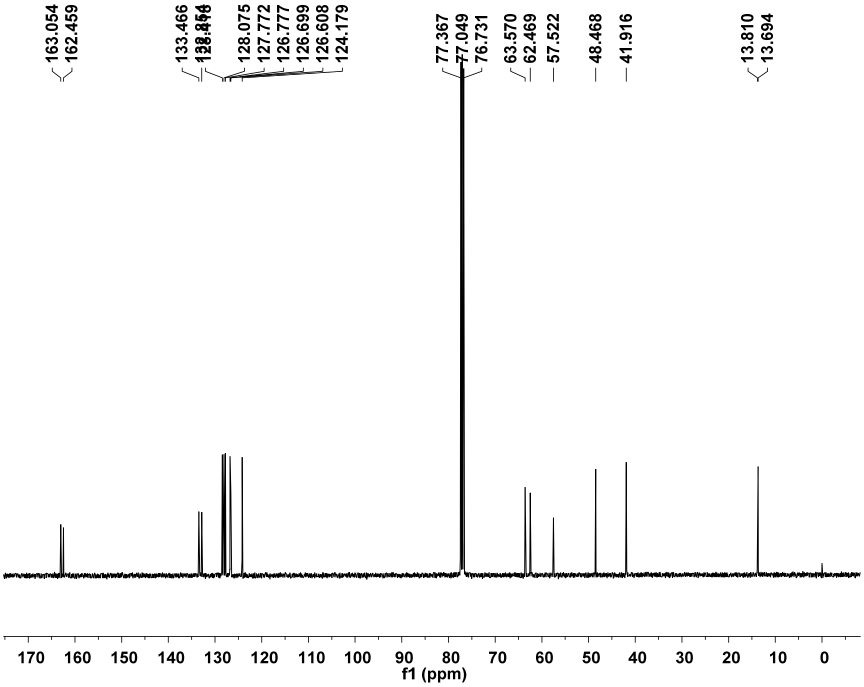

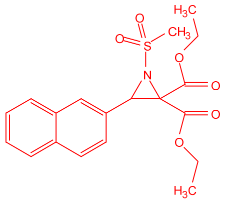

Current Data Parameters

F2 - Acquisition Parameters
DATE: 2015-01-26T20:59:32
PULPROG: zgpg30
TD: 32768
Solvent: CDCl3
NS: 1024
DS: undefined
SWH: 24038.5 Hz
AQ: undefined
TE: 294.1 C

======== CHANNEL f1 ========
NUC1: 13C
P1: 9.63 usec
SFO1: undefined MHz

F2 - Processing Parameters
SI: 65536
DC: 0.05
LB: 1.00 Hz
First Point: 0.50
FT: Hyper Quadrature
Phase: Manual
Ph0: -63.22
Ph1: 58.38

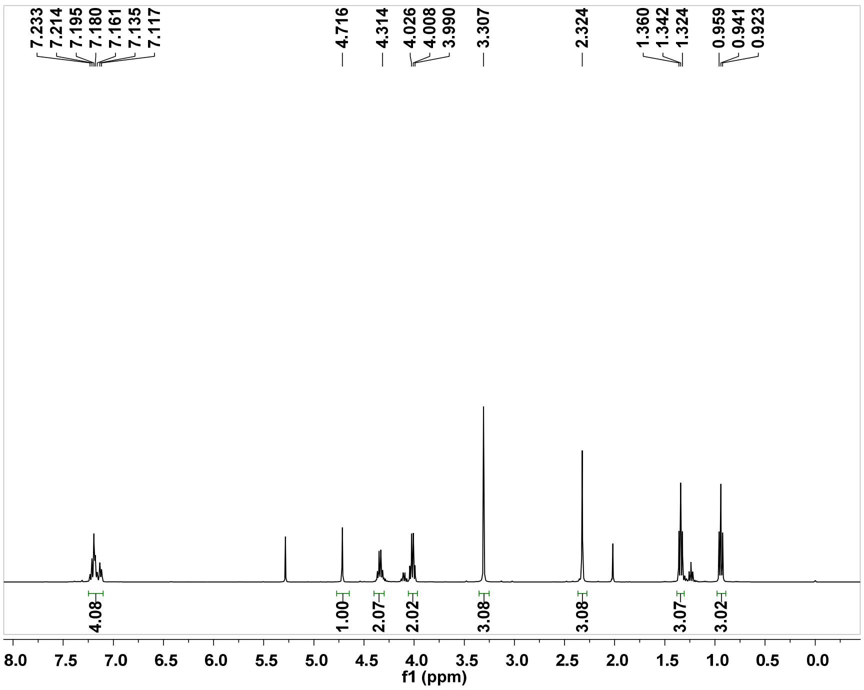

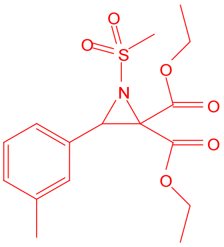

Current Data Parameters

F2 - Acquisition Parameters
DATE: 2015-06-04T23:42:03
PULPROG: zg30
TD: 32768
Solvent: CDCl3
NS: 16
DS: undefined
SWH: 8223.7 Hz
AQ: undefined
TE: 295 C

======== CHANNEL f1 ========
NUC1: 1H
P1: 9.93 usec
SFO1: undefined MHz

F2 - Processing Parameters
SI: 65536
DC: 0.05
LB: 0.30 Hz
First Point: 0.50
FT: Hyper Quadrature
Phase: Manual
Ph0: 96.36
Ph1: 19.56

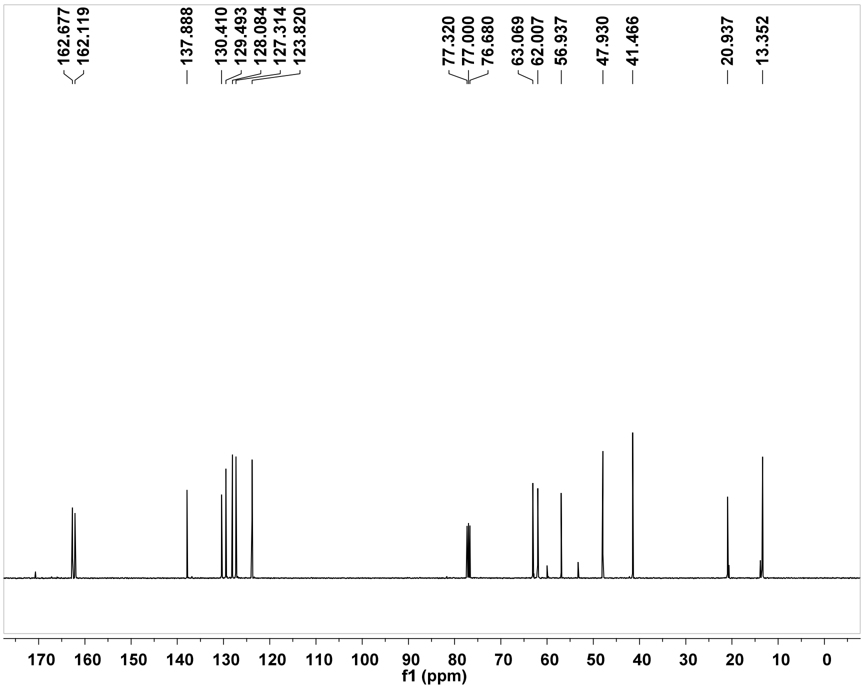

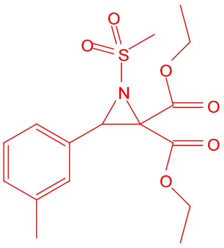

Current Data Parameters

F2 - Acquisition Parameters
DATE: 2015-06-04T23:57:38
PULPROG: zgpg30
TD: 32768
Solvent: CDCl3
NS: 256
DS: undefined
SWH: 24038.5 Hz
AQ: undefined
TE: 295.3 C

======== CHANNEL f1 ========
NUC1: 13C
P1: 9.63 usec
SFO1: undefined MHz

F2 - Processing Parameters
SI: 65536
DC: 0.05
LB: 1.00 Hz
First Point: 0.50
FT: Hyper Quadrature
Phase: Manual
Ph0: -61.76
Ph1: 64.82

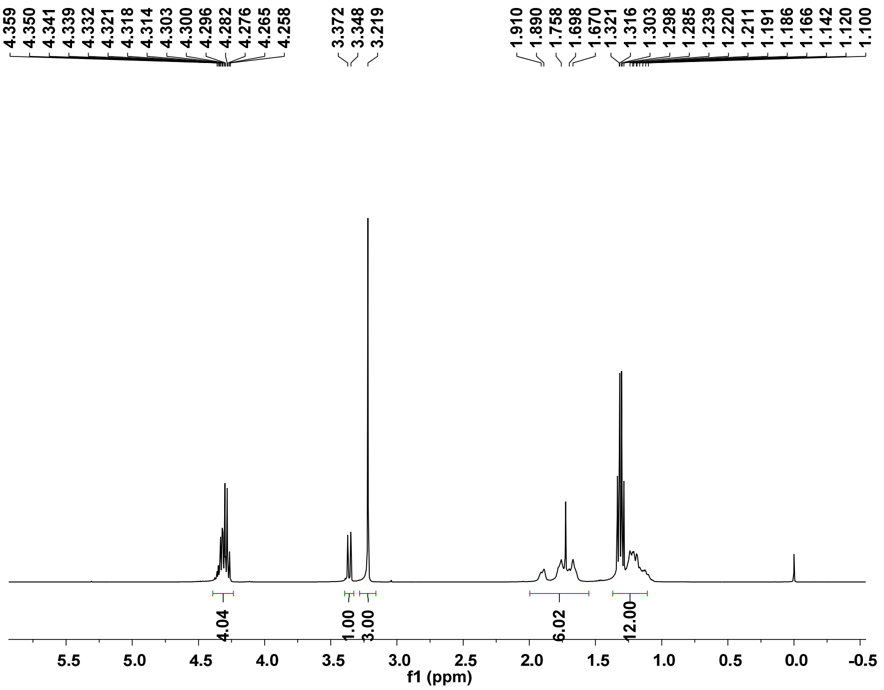

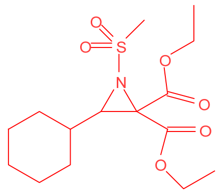

Current Data Parameters

F2 - Acquisition Parameters
DATE: 2015-07-15T04:11:12
PULPROG: zg30
TD: 32768
Solvent: CDCl3
NS: 32
DS: undefined
SWH: 8223.7 Hz
AQ: undefined
TE: 0 C

======== CHANNEL f1 ========
NUC1: 1H
P1: 9.93 usec
SFO1: undefined MHz

F2 - Processing Parameters
SI: 65536
DC: 0.05
LB: 0.30 Hz
First Point: 0.50
FT: Hyper Quadrature
Phase: Manual
Ph0: 96.60
Ph1: 17.60

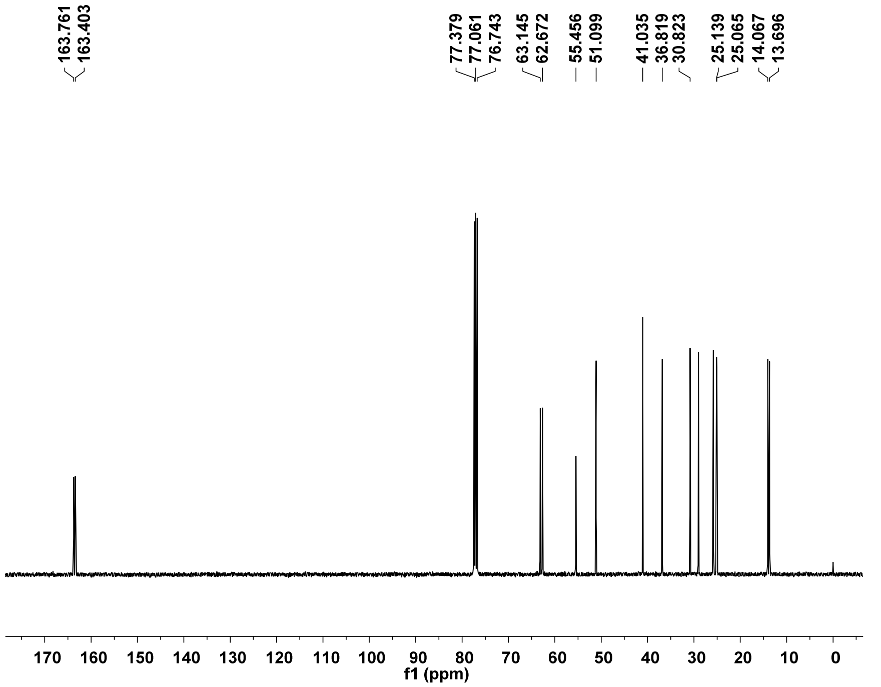

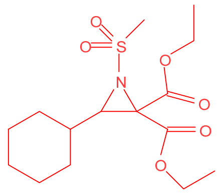

Current Data Parameters

F2 - Acquisition Parameters
DATE: 2015-07-15T05:10:38
PULPROG: zgpg30
TD: 32768
Solvent: CDCl3
NS: 1024
DS: undefined
SWH: 24038.5 Hz
AQ: undefined
TE: 0 C

======== CHANNEL f1 ========
NUC1: 13C
P1: 9.63 usec
SFO1: undefined MHz

F2 - Processing Parameters
SI: 65536
DC: 0.05
LB: 1.00 Hz
First Point: 0.50
FT: Hyper Quadrature
Phase: Manual
Ph0: -59.63
Ph1: 64.40

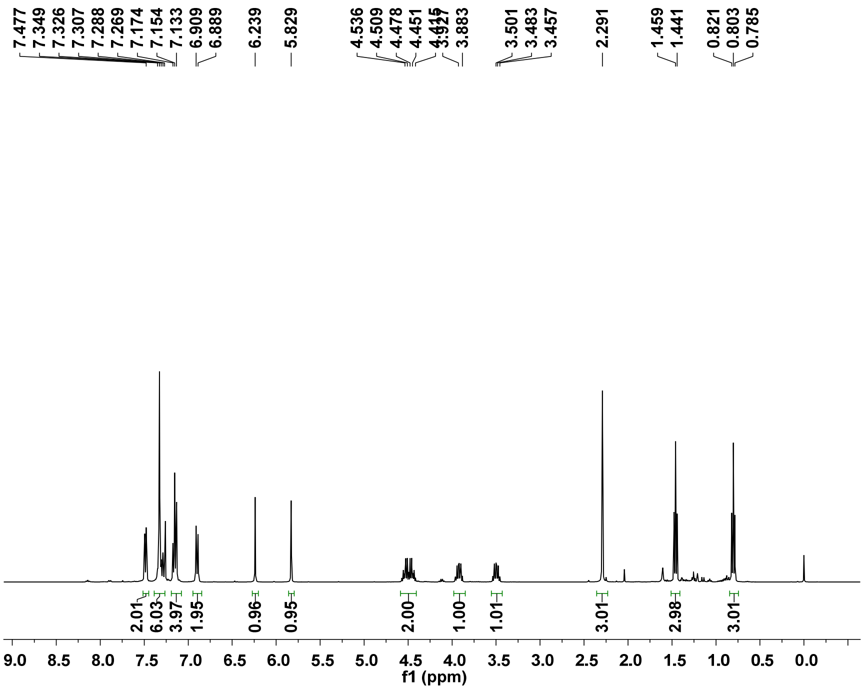

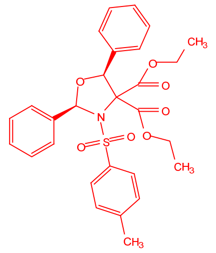

Current Data Parameters

F2 - Acquisition Parameters
DATE: 2014-12-05T16:29:32
PULPROG: zg30
TD: 32768
Solvent: CDCl3
NS: 32
DS: undefined
SWH: 8223.7 Hz
AQ: undefined
TE: 295.5 C

======== CHANNEL f1 ========
NUC1: 1H
P1: 9.93 usec
SFO1: undefined MHz

F2 - Processing Parameters
SI: 65536
DC: 0.05
LB: 0.30 Hz
First Point: 0.50
FT: Hyper Quadrature
Phase: Manual
Ph0: 88.91
Ph1: 18.39

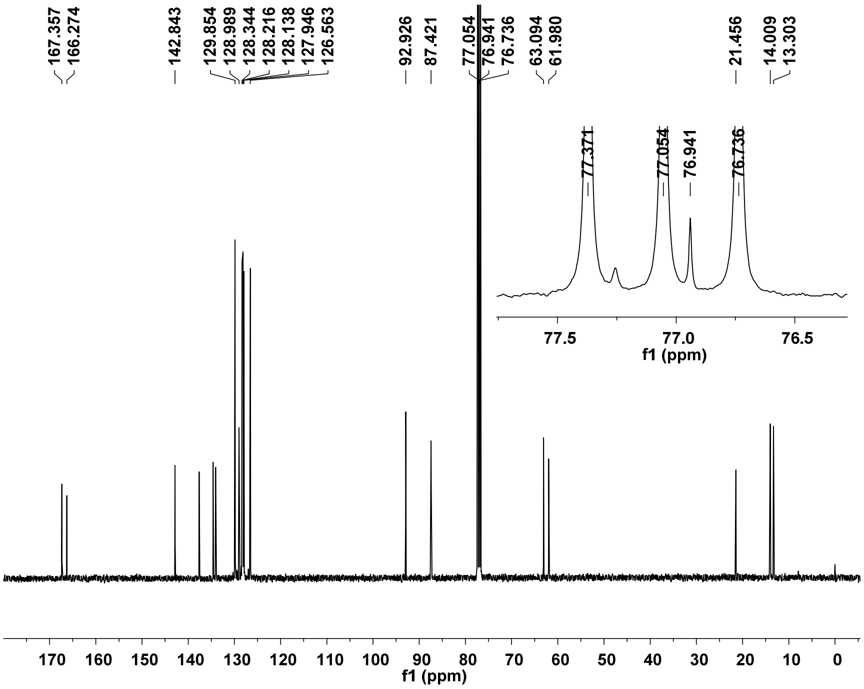

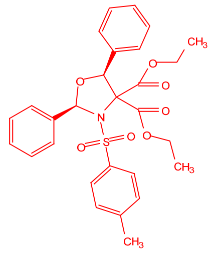

Current Data Parameters

F2 - Acquisition Parameters
DATE: 2014-12-05T17:46:37
PULPROG: zgpg30
TD: 32768
Solvent: CDCl3
NS: 1024
DS: undefined
SWH: 24038.5 Hz
AQ: undefined
TE: 296 C

======== CHANNEL f1 ========
NUC1: 13C
P1: 9.63 usec
SFO1: undefined MHz

F2 - Processing Parameters
SI: 65536
DC: 0.05
LB: 1.00 Hz
First Point: 0.50
FT: Hyper Quadrature
Phase: Manual
Ph0: -69.35
Ph1: 70.71

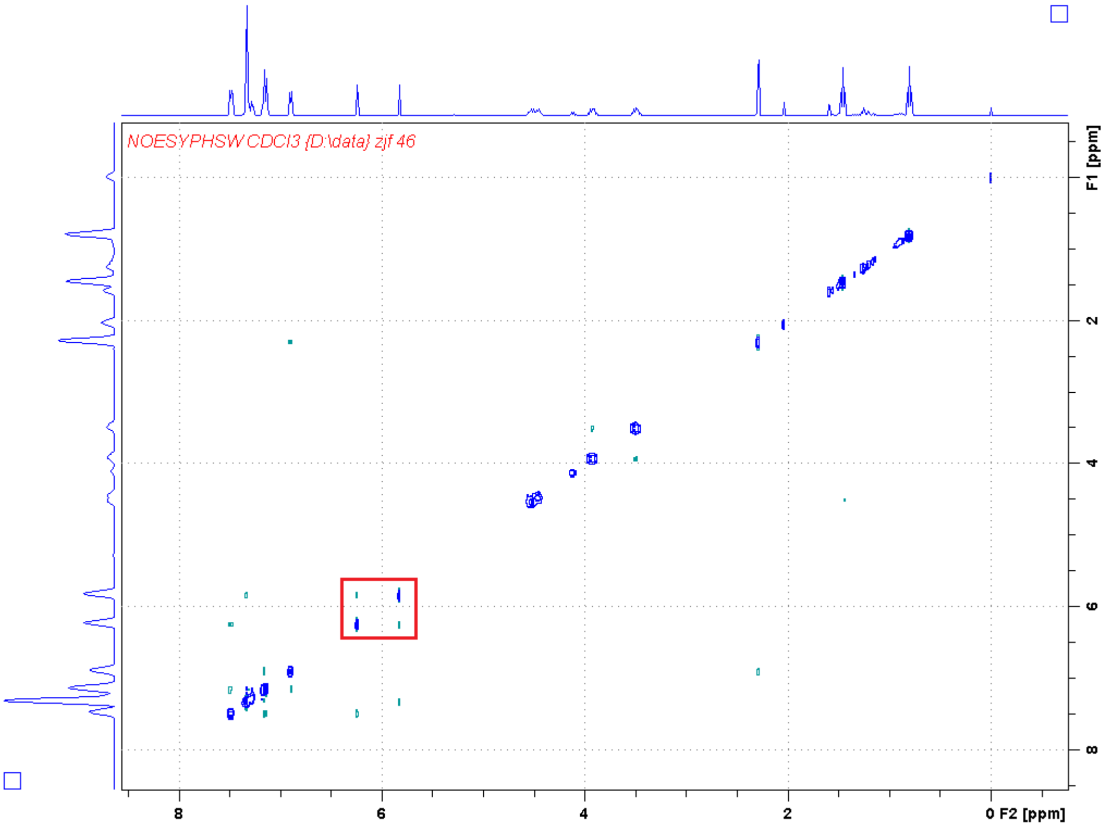
NOESYPHSW CDCl3 {D:\data} zjf 46
F1 [ppm]
F2 [ppm]
8
6
4
2
0
2
4
6
8

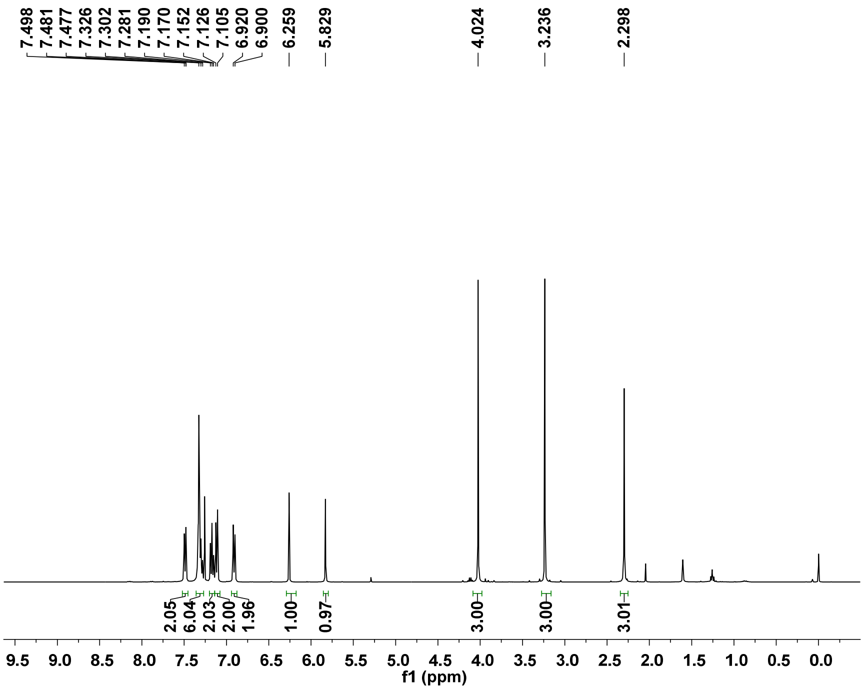

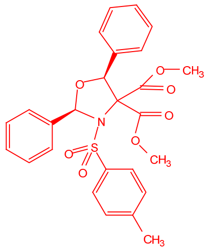

Current Data Parameters

F2 - Acquisition Parameters
DATE: 2014-12-23T14:56:22
PULPROG: zg30
TD: 32768
Solvent: CDCl3
NS: 32
DS: undefined
SWH: 8223.7 Hz
AQ: undefined
TE: 293.6 C

======== CHANNEL f1 ========
NUC1: 1H
P1: 9.93 usec
SFO1: undefined MHz

F2 - Processing Parameters
SI: 65536
DC: 0.05
LB: 0.30 Hz
First Point: 0.50
FT: Hyper Quadrature
Phase: Manual
Ph0: -264.99
Ph1: 17.83

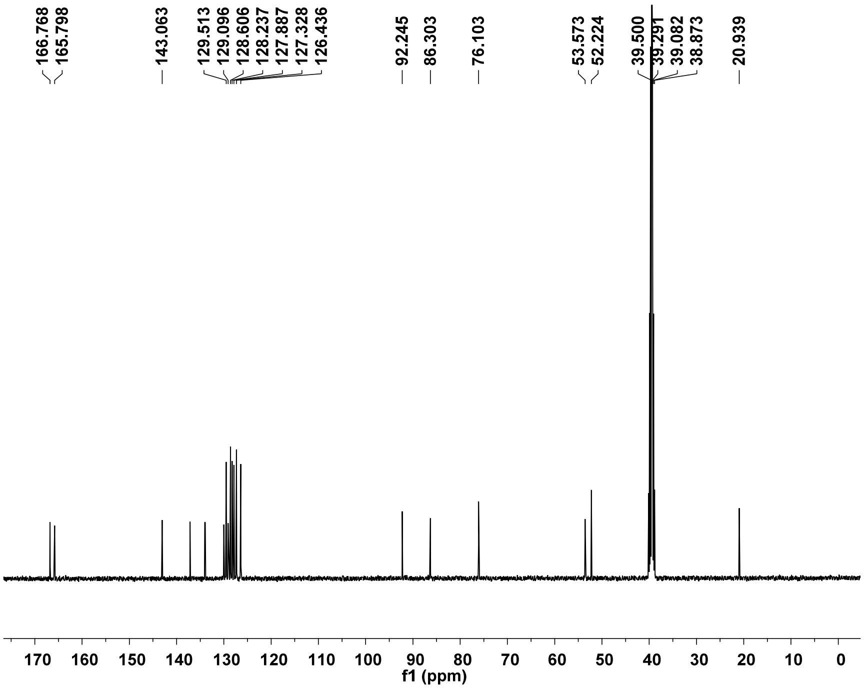

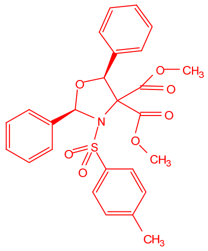

Current Data Parameters

F2 - Acquisition Parameters
DATE: 2015-06-14T01:32:59
PULPROG: zgpg30
TD: 32768
Solvent: DMSO
NS: 512
DS: undefined
SWH: 24038.5 Hz
AQ: undefined
TE: 295.8 C

======== CHANNEL f1 ========
NUC1: 13C
P1: 9.63 usec
SFO1: undefined MHz

F2 - Processing Parameters
SI: 65536
DC: 0.05
LB: 1.00 Hz
First Point: 0.50
FT: Hyper Quadrature
Phase: Manual
Ph0: -58.73
Ph1: 60.63

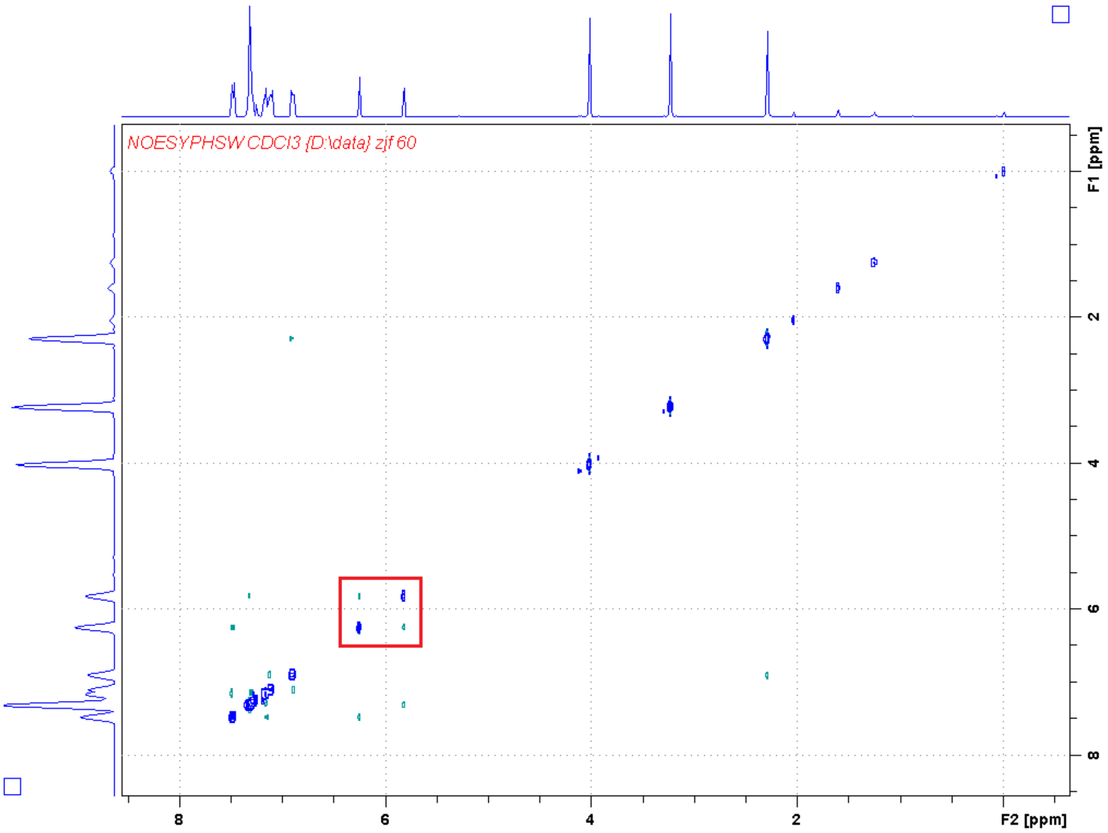
NOESYPHSW CDCl3 {D:\data} zjf 60
F1 [ppm]
F2 [ppm]
8
6
4
2

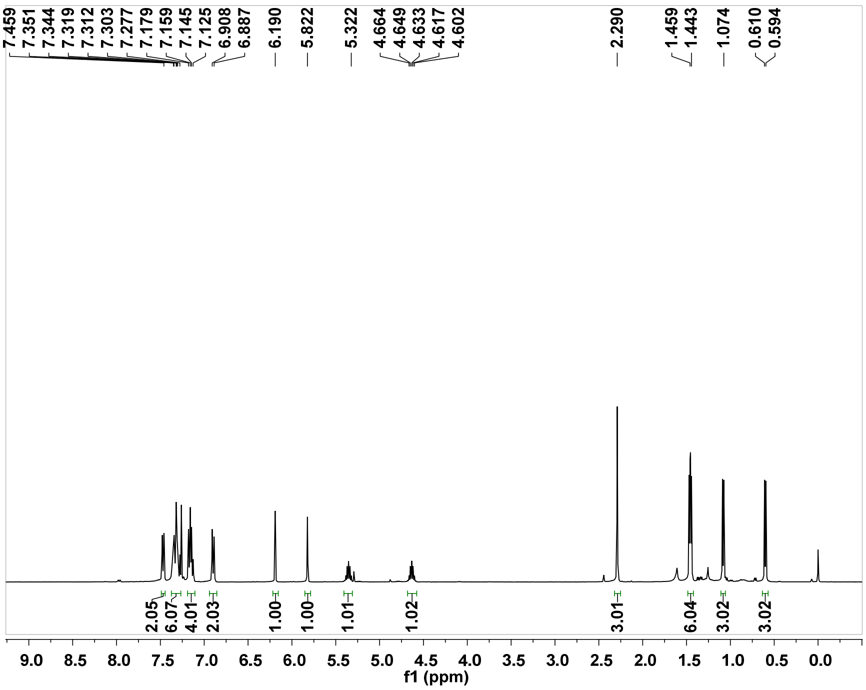

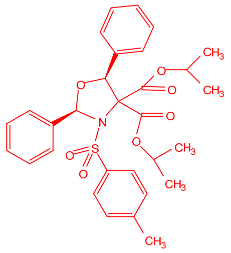

Current Data Parameters

F2 - Acquisition Parameters
DATE: 2015-05-16T23:39:26
PULPROG: zg30
TD: 32768
Solvent: CDCl3
NS: 16
DS: undefined
SWH: 8223.7 Hz
AQ: undefined
TE: 295.1 C

======== CHANNEL f1 ========
NUC1: 1H
P1: 9.93 usec
SFO1: undefined MHz

F2 - Processing Parameters
SI: 65536
DC: 0.05
LB: 0.30 Hz
First Point: 0.50
FT: Hyper Quadrature
Phase: Manual
Ph0: 94.98
Ph1: 17.35

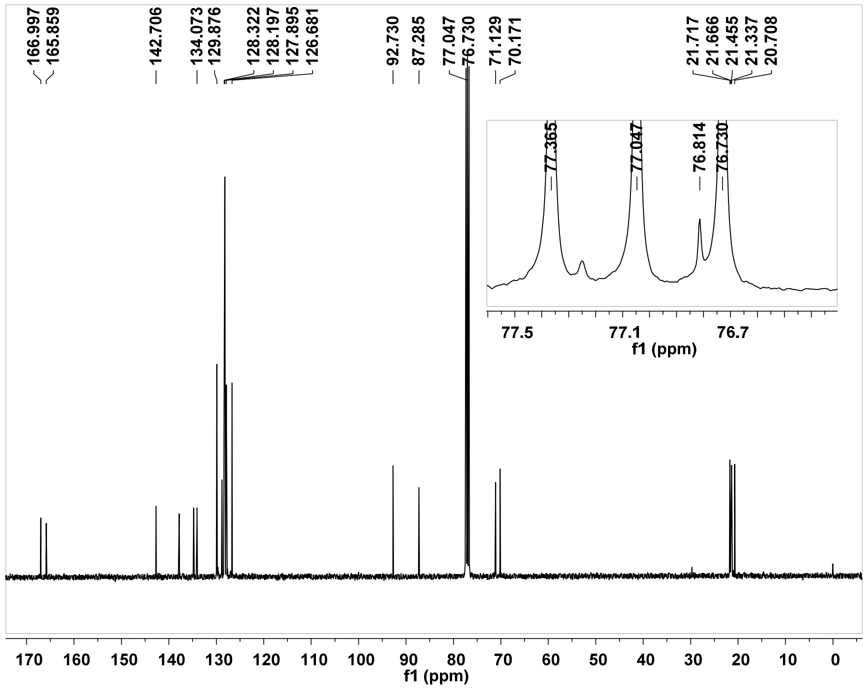

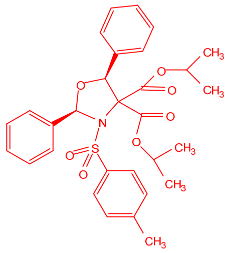

Current Data Parameters

F2 - Acquisition Parameters
DATE: 2015-05-17T01:35:10
PULPROG: zgpg30
TD: 32768
Solvent: CDCl3
NS: 1024
DS: undefined
SWH: 24038.5 Hz
AQ: undefined
TE: 295.7 C

======== CHANNEL f1 ========
NUC1: 13C
P1: 9.63 usec
SFO1: undefined MHz

F2 - Processing Parameters
SI: 65536
DC: 0.05
LB: 1.00 Hz
First Point: 0.50
FT: Hyper Quadrature
Phase: Manual
Ph0: -60.76
Ph1: 61.81

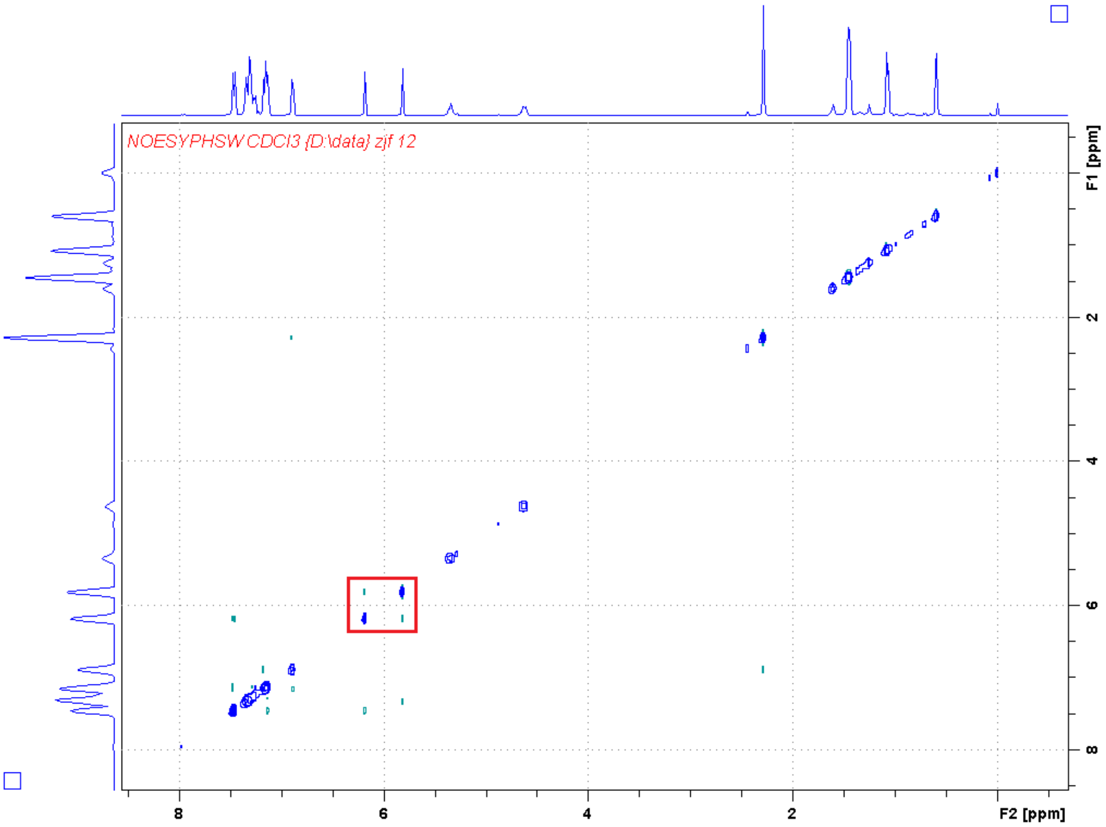

NOESYPHSW CDCl3 {D:\data} zjf 12
F1 [ppm]
F2 [ppm]
8
6
4
2

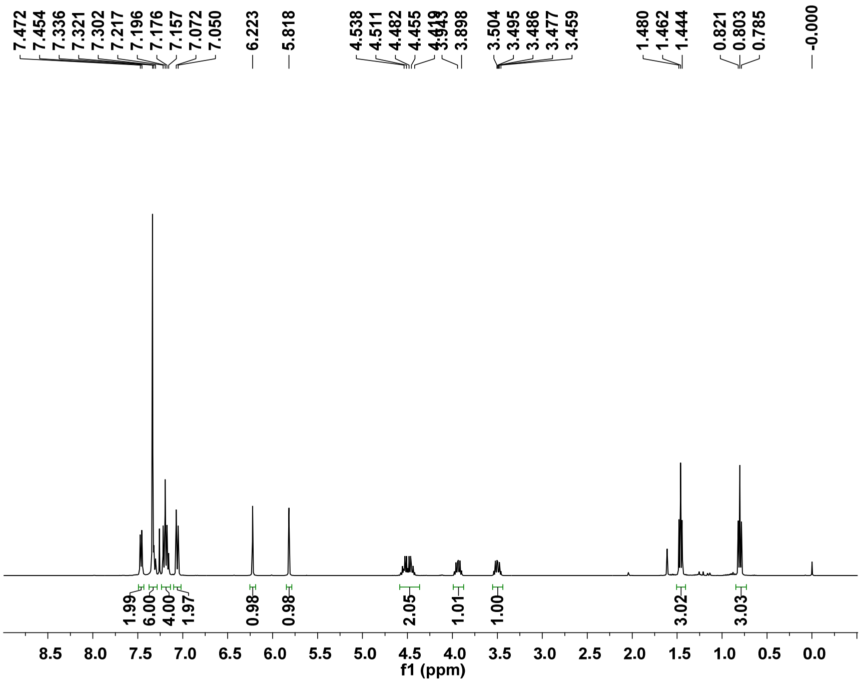

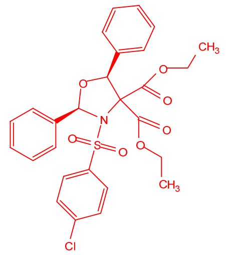

Current Data Parameters

F2 - Acquisition Parameters
DATE: 2014-12-03T12:38:23
PULPROG: zg30
TD: 32768
Solvent: CDCl3
NS: 32
DS: undefined
SWH: 8223.7 Hz
AQ: undefined
TE: 295.7 C

======== CHANNEL f1 ========
NUC1: 1H
P1: 9.93 usec
SFO1: undefined MHz

F2 - Processing Parameters
SI: 65536
DC: 0.05
LB: 0.30 Hz
First Point: 0.50
FT: Hyper Quadrature
Phase: Manual
Ph0: 89.91
Ph1: 18.93

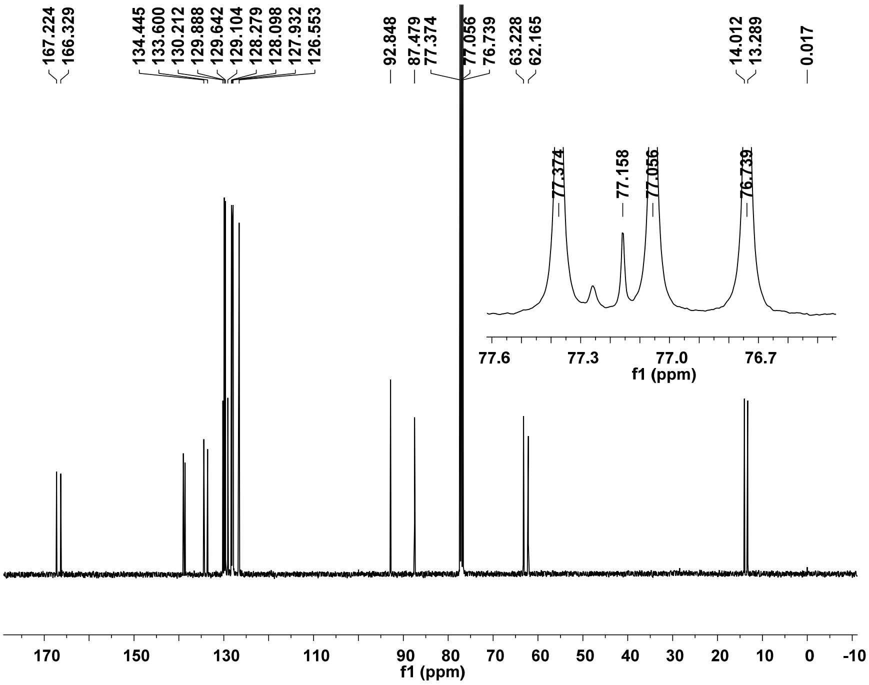

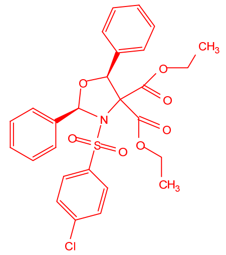

Current Data Parameters

F2 - Acquisition Parameters
DATE: 2014-12-03T19:08:57
PULPROG: zgpg30
TD: 32768
Solvent: CDCl3
NS: 1024
DS: undefined
SWH: 24038.5 Hz
AQ: undefined
TE: 296 C

======== CHANNEL f1 ========
NUC1: 13C
P1: 9.63 usec
SFO1: undefined MHz

F2 - Processing Parameters
SI: 65536
DC: 0.05
LB: 1.00 Hz
First Point: 0.50
FT: Hyper Quadrature
Phase: Manual
Ph0: -71.08
Ph1: 67.97

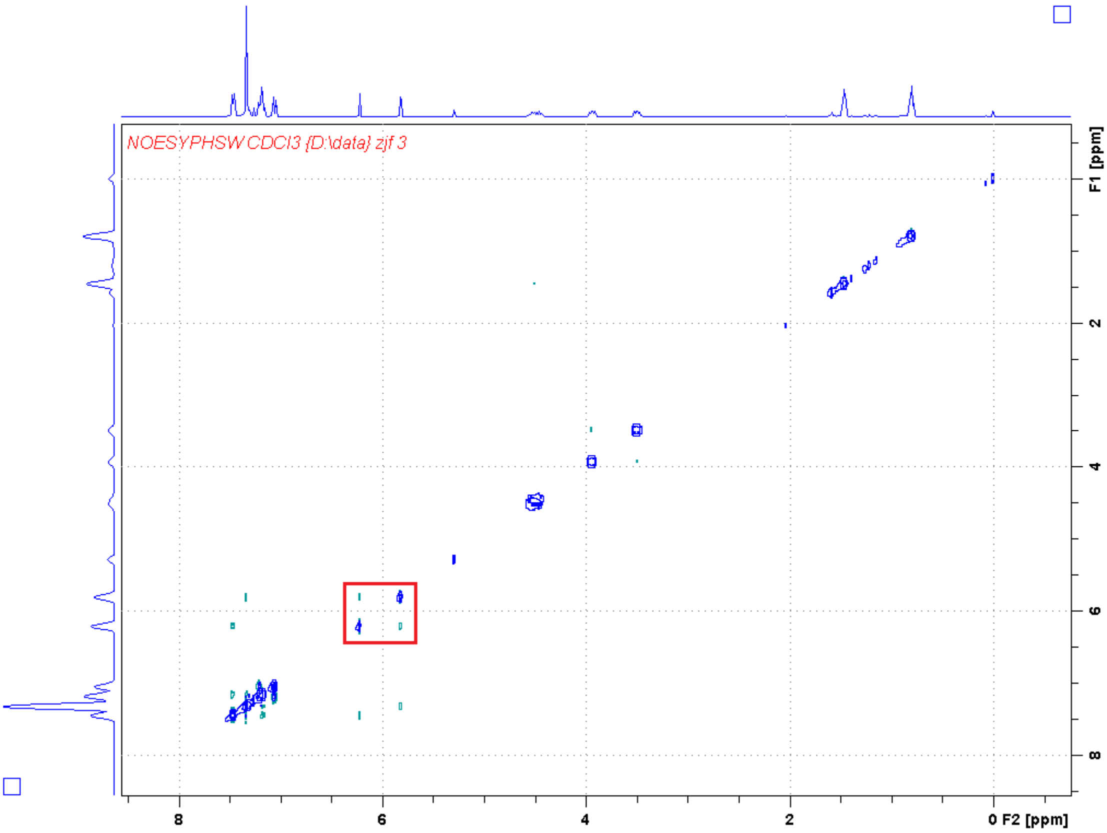

NOESYPHSW CDCl3 {D:\data} zjf 3
F1 [ppm]
F2 [ppm]
8
6
4
2
0

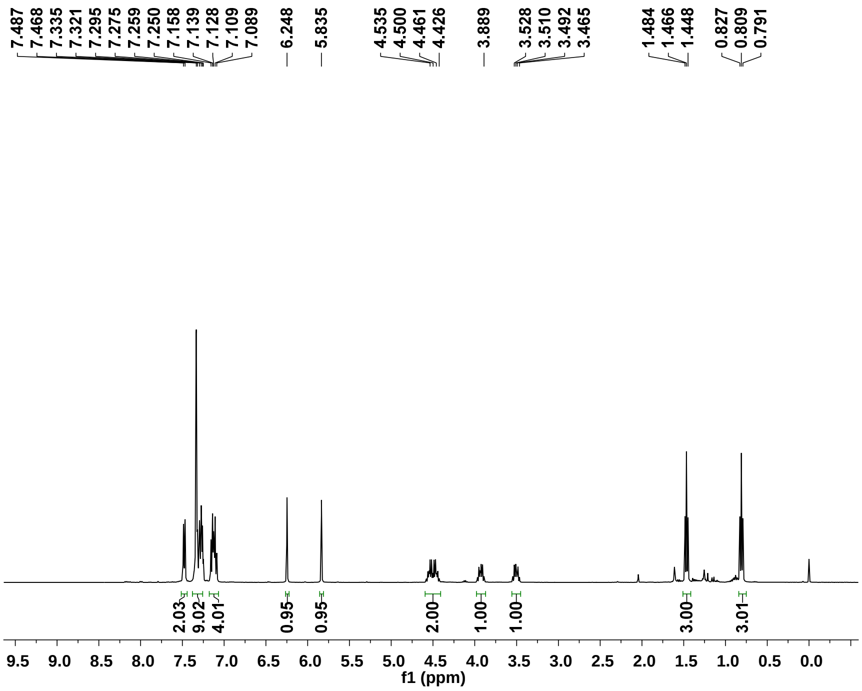

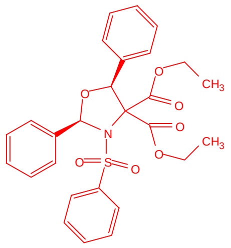

Current Data Parameters

F2 - Acquisition Parameters
DATE: 2014-12-11T16:06:31
PULPROG: zg30
TD: 32768
Solvent: CDCl3
NS: 32
DS: undefined
SWH: 8223.7 Hz
AQ: undefined
TE: 293.6 C

======== CHANNEL f1 ========
NUC1: 1H
P1: 9.93 usec
SFO1: undefined MHz

F2 - Processing Parameters
SI: 65536
DC: 0.05
LB: 0.30 Hz
First Point: 0.50
FT: Hyper Quadrature
Phase: Manual
Ph0: 92.94
Ph1: 18.25

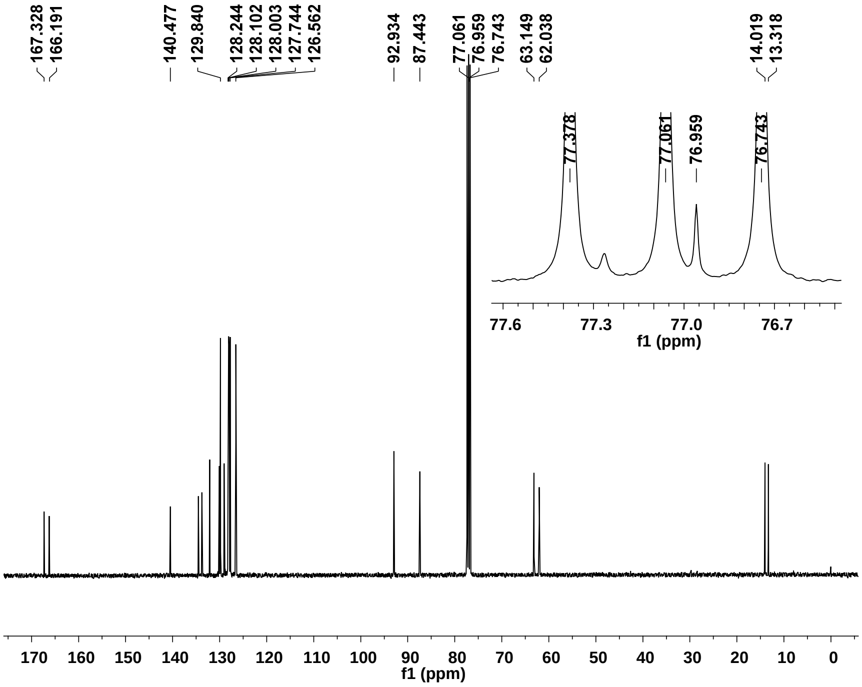

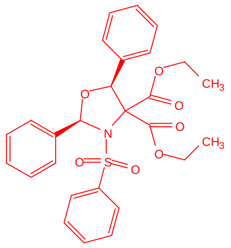

Current Data Parameters

F2 - Acquisition Parameters
DATE: 2014-12-11T21:24:54
PULPROG: zgpg30
TD: 32768
Solvent: CDCl3
NS: 1024
DS: undefined
SWH: 24038.5 Hz
AQ: undefined
TE: 294 C

======== CHANNEL f1 ========
NUC1: 13C
P1: 9.63 usec
SFO1: undefined MHz

F2 - Processing Parameters
SI: 65536
DC: 0.05
LB: 1.00 Hz
First Point: 0.50
FT: Hyper Quadrature
Phase: Manual
Ph0: -64.56
Ph1: 66.78

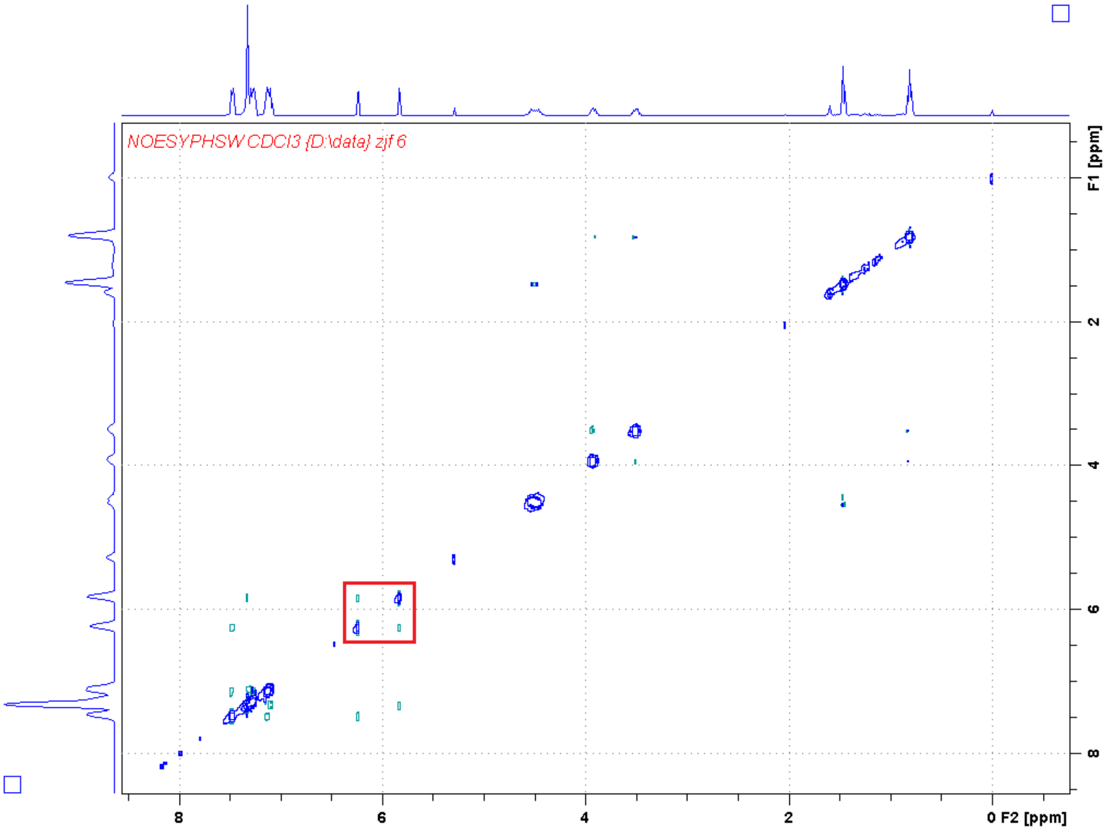
NOESYPHSW CDCl3 {D:\data} zjf 6
F1 [ppm]
2
4
6
8
0 F2 [ppm]

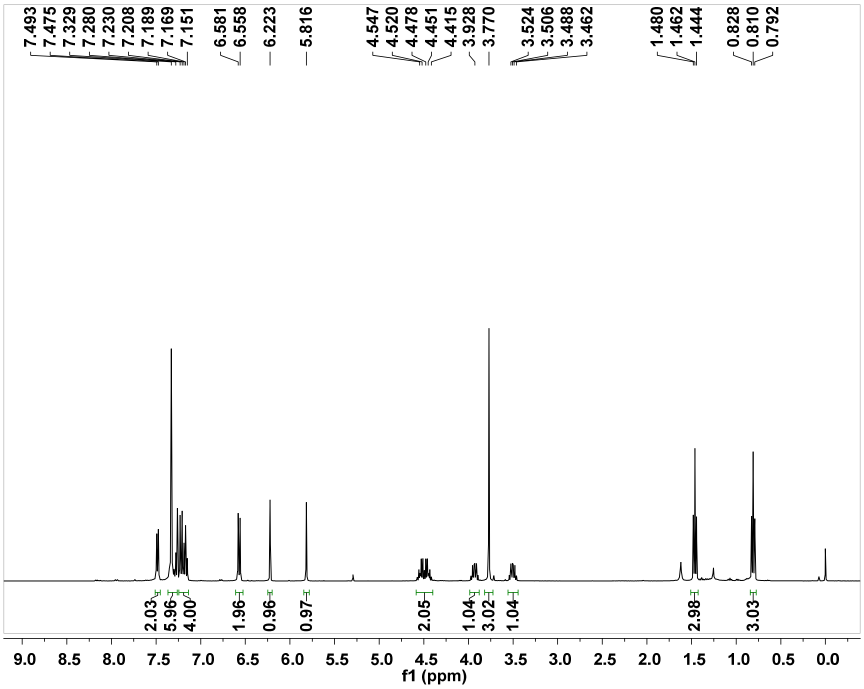

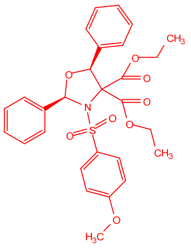

Current Data Parameters

F2 - Acquisition Parameters
DATE: 2015-05-17T01:39:59
PULPROG: zg30
TD: 32768
Solvent: CDCl3
NS: 16
DS: undefined
SWH: 8223.7 Hz
AQ: undefined
TE: 295.4 C

======== CHANNEL f1 ========
NUC1: 1H
P1: 9.93 usec
SFO1: undefined MHz

F2 - Processing Parameters
SI: 65536
DC: 0.05
LB: 0.30 Hz
First Point: 0.50
FT: Hyper Quadrature
Phase: Manual
Ph0: -265.46
Ph1: 14.39

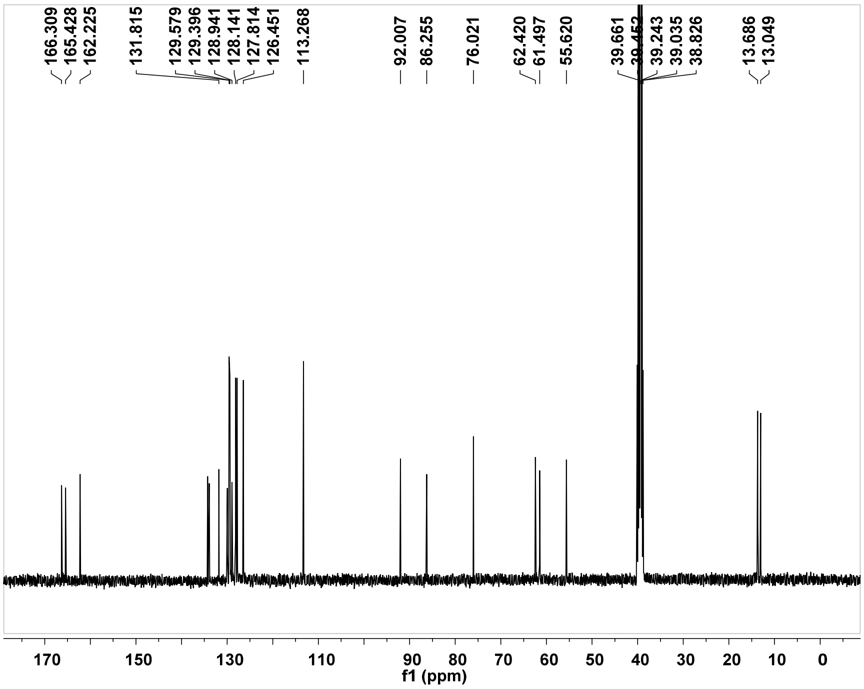

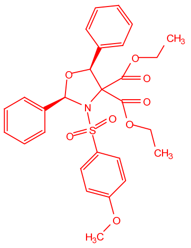

Current Data Parameters

F2 - Acquisition Parameters
DATE: 2015-05-20T03:53:59
PULPROG: zgpg30
TD: 32768
Solvent: DMSO
NS: 512
DS: undefined
SWH: 24038.5 Hz
AQ: undefined
TE: 296.2 C

======== CHANNEL f1 ========
NUC1: 13C
P1: 9.63 usec
SFO1: undefined MHz

F2 - Processing Parameters
SI: 65536
DC: 0.05
LB: 1.00 Hz
First Point: 0.50
FT: Hyper Quadrature
Phase: Manual
Ph0: -69.11
Ph1: 76.73

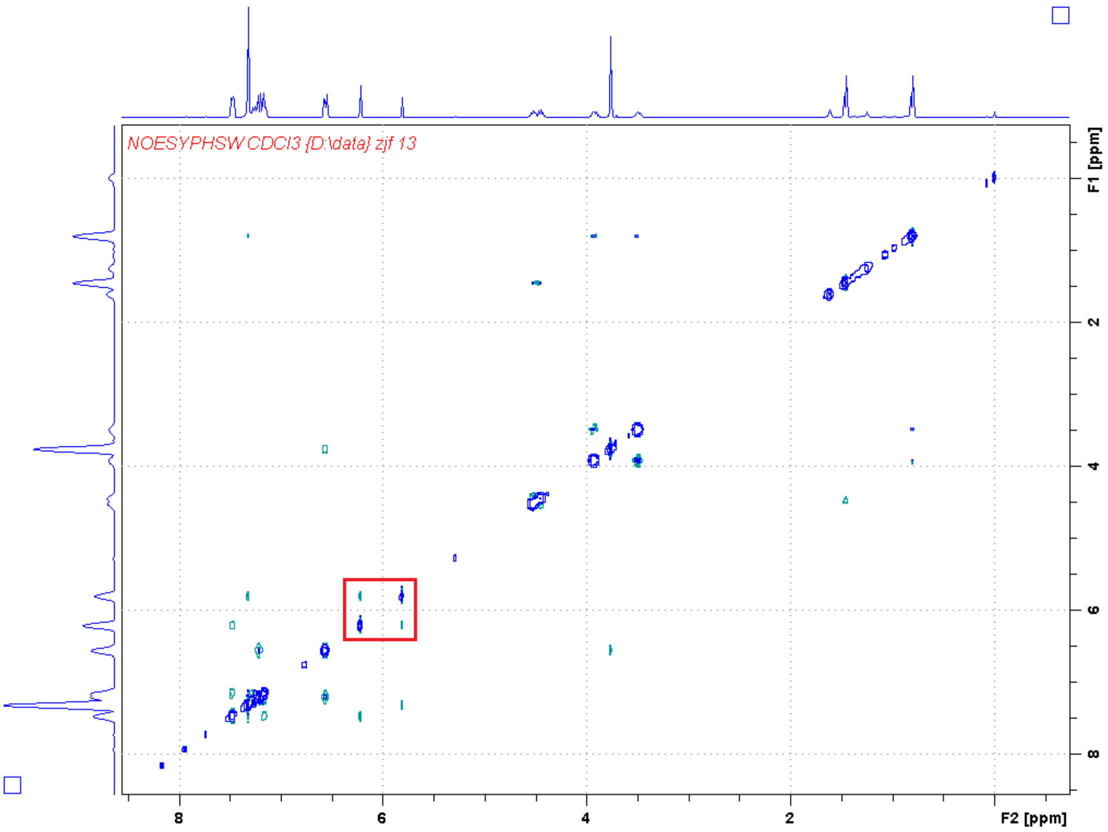
NOESYPHSW CDCl3 {D:\data} zjf 13
F1 [ppm]
F2 [ppm]
8
6
4
2
2
4
6
8

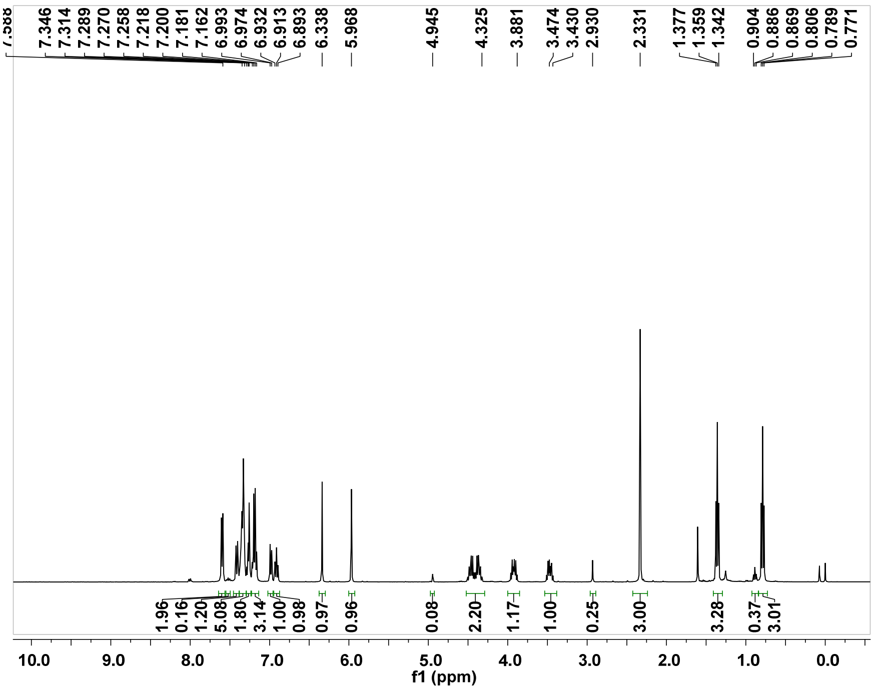

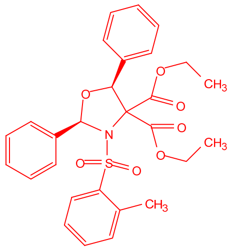

Current Data Parameters

F2 - Acquisition Parameters
DATE: 2015-05-23T16:23:25
PULPROG: zg30
TD: 32768
Solvent: CDCl3
NS: 16
DS: undefined
SWH: 8223.7 Hz
AQ: undefined
TE: 295.1 C

======== CHANNEL f1 ========
NUC1: 1H
P1: 9.93 usec
SFO1: undefined MHz

F2 - Processing Parameters
SI: 65536
DC: 0.05
LB: 0.30 Hz
First Point: 0.50
FT: Hyper Quadrature
Phase: Manual
Ph0: -261.15
Ph1: 16.99

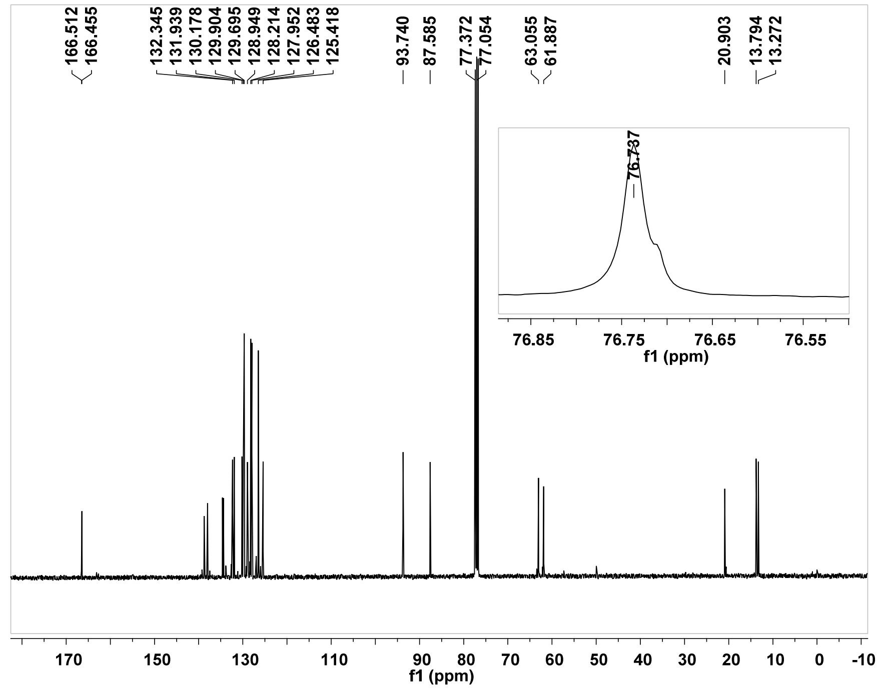

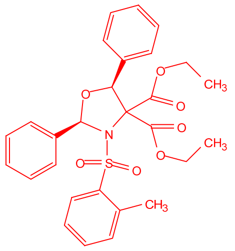

Current Data Parameters

F2 - Acquisition Parameters
DATE: 2015-05-23T18:40:41
PULPROG: zgpg30
TD: 32768
Solvent: CDCl3
NS: 1024
DS: undefined
SWH: 24038.5 Hz
AQ: undefined
TE: 295.8 C

======== CHANNEL f1 ========
NUC1: 13C
P1: 9.63 usec
SFO1: undefined MHz

F2 - Processing Parameters
SI: 65536
DC: 0.05
LB: 1.00 Hz
First Point: 0.50
FT: Hyper Quadrature
Phase: Manual
Ph0: -52.77
Ph1: 52.67

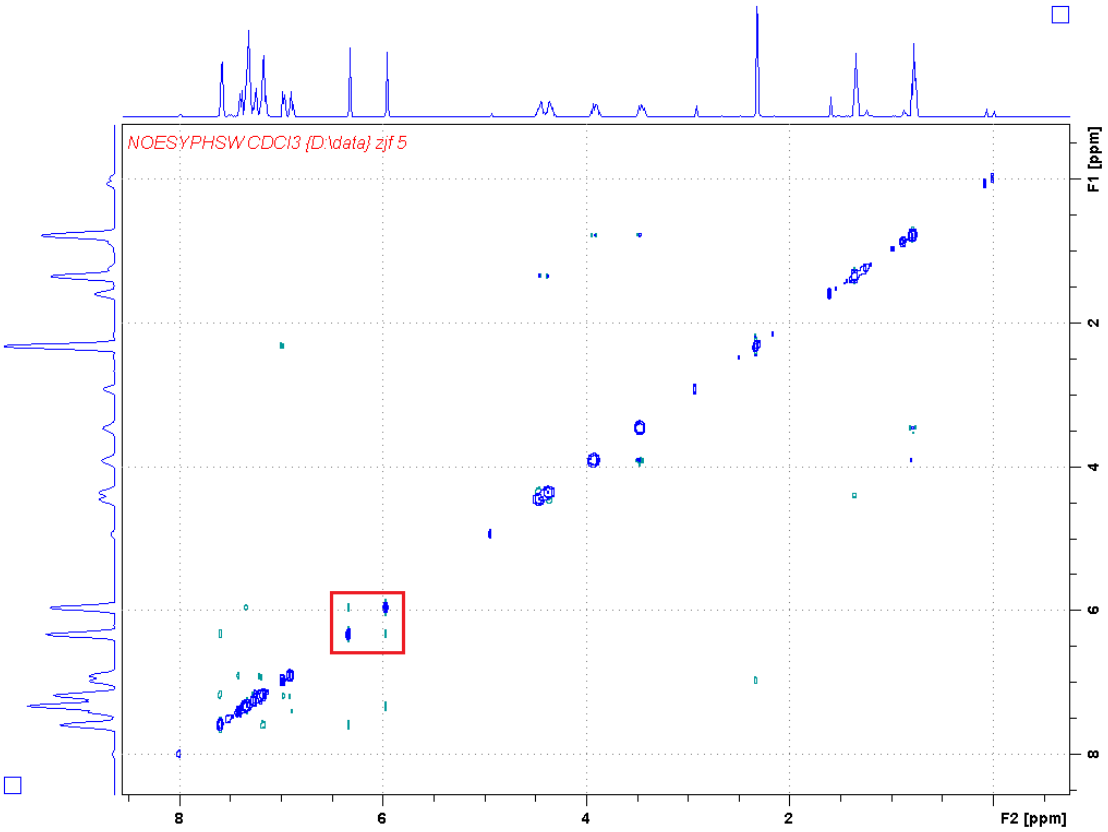
NOESYPHSW CDCl3 {D:\data} zjf 5
F1 [ppm]
F2 [ppm]
8
6
4
2

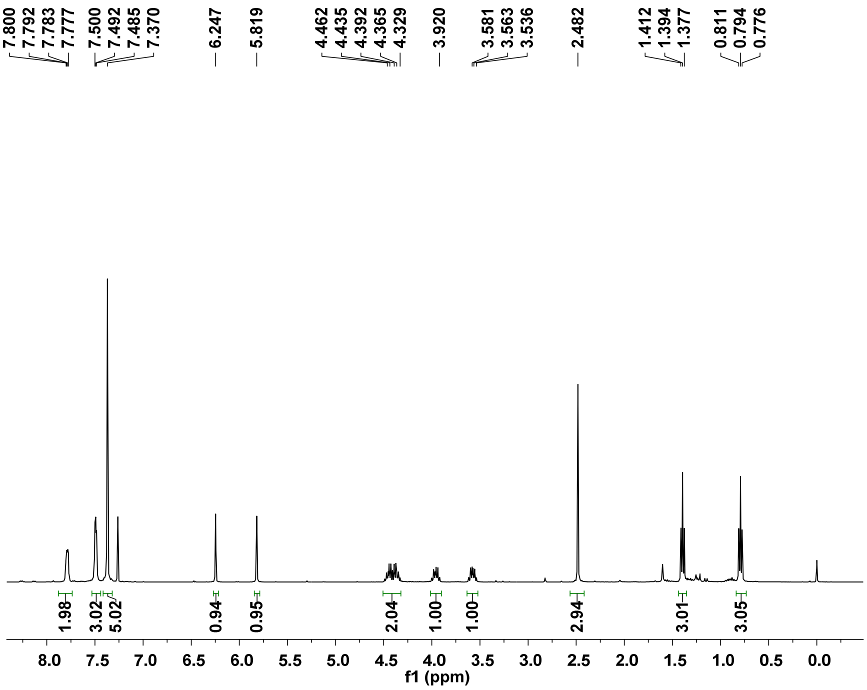

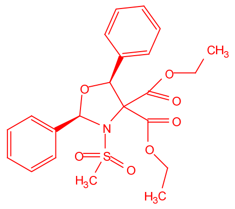

Current Data Parameters

F2 - Acquisition Parameters
DATE: 2014-12-11T16:21:58
PULPROG: zg30
TD: 32768
Solvent: CDCl3
NS: 32
DS: undefined
SWH: 8223.7 Hz
AQ: undefined
TE: 293.5 C

======== CHANNEL f1 ========
NUC1: 1H
P1: 9.93 usec
SFO1: undefined MHz

F2 - Processing Parameters
SI: 65536
DC: 0.05
LB: 0.30 Hz
First Point: 0.50
FT: Hyper Quadrature
Phase: Manual
Ph0: 93.18
Ph1: 15.03

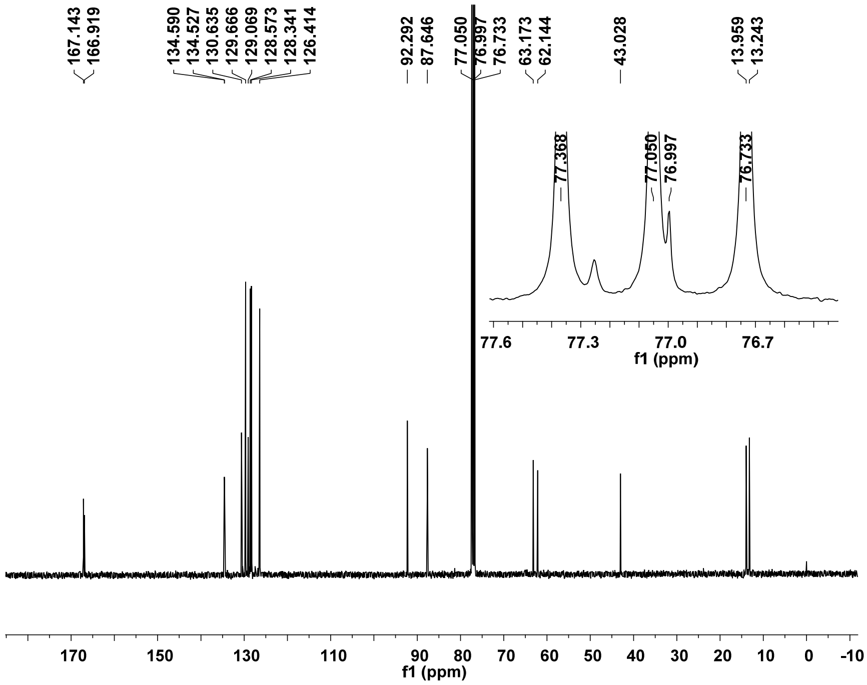

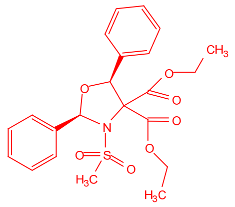

Current Data Parameters

F2 - Acquisition Parameters
DATE: 2014-12-11T23:20:52
PULPROG: zgpg30
TD: 32768
Solvent: CDCl3
NS: 1024
DS: undefined
SWH: 24038.5 Hz
AQ: undefined
TE: 294.2 C

======== CHANNEL f1 ========
NUC1: 13C
P1: 9.63 usec
SFO1: undefined MHz

F2 - Processing Parameters
SI: 65536
DC: 0.05
LB: 1.00 Hz
First Point: 0.50
FT: Hyper Quadrature
Phase: Manual
Ph0: -68.48
Ph1: 71.76

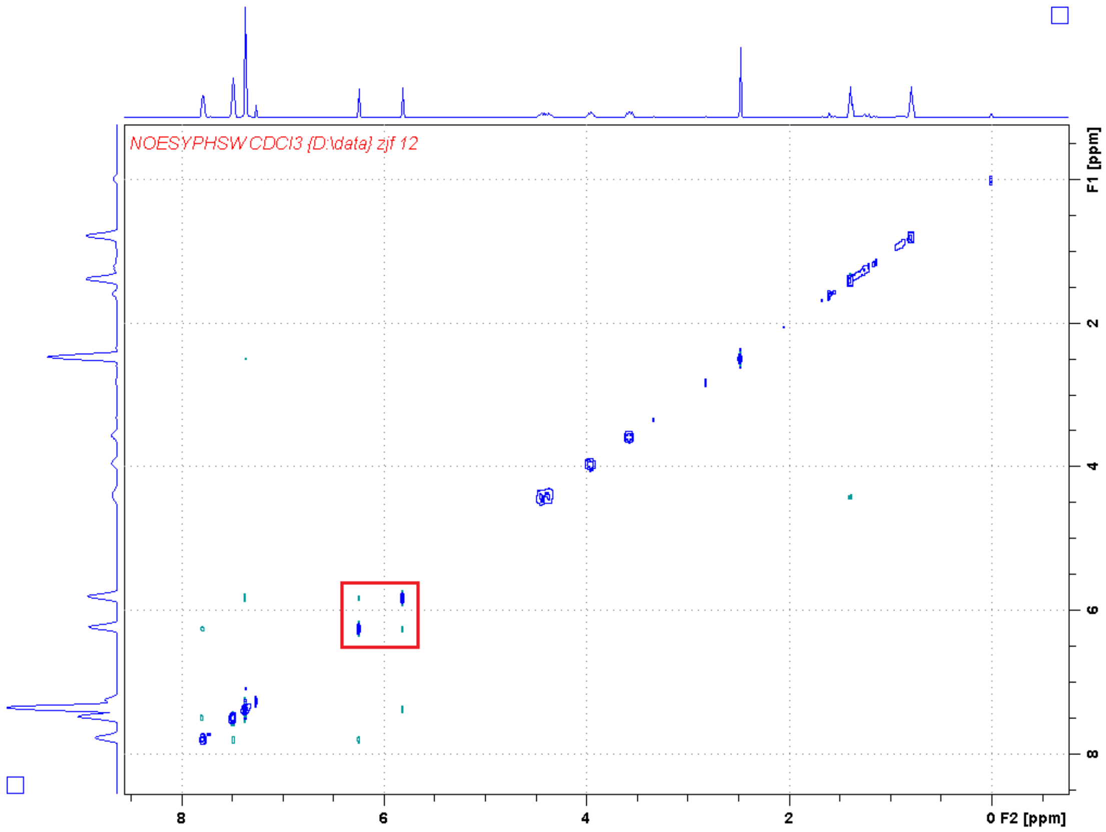

NOESYPHSW CDCl3 {D:\data} zjf 12
F1 [ppm]
2
4
6
8
8
6
4
2
0 F2 [ppm]

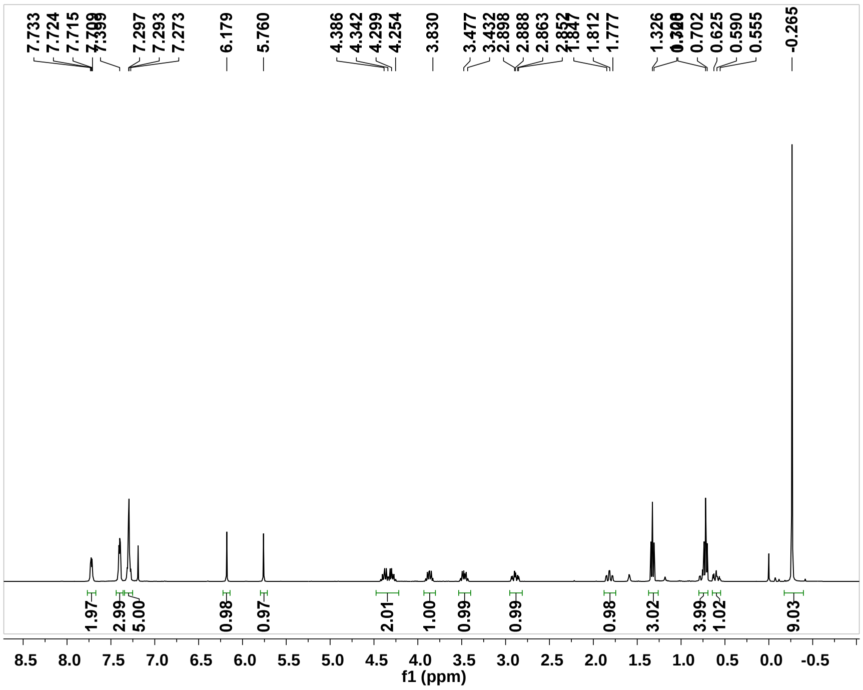

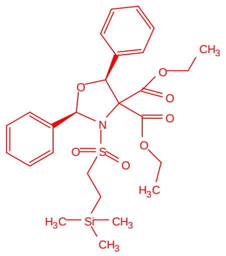

Current Data Parameters

F2 - Acquisition Parameters
DATE: 2015-05-27T23:59:27
PULPROG: zg30
TD: 32768
Solvent: CDCl3
NS: 32
DS: undefined
SWH: 8223.7 Hz
AQ: undefined
TE: 295.5 C

======== CHANNEL f1 ========
NUC1: 1H
P1: 9.93 usec
SFO1: undefined MHz

F2 - Processing Parameters
SI: 65536
DC: 0.05
LB: 0.30 Hz
First Point: 0.50
FT: Hyper Quadrature
Phase: Manual
Ph0: -260.15
Ph1: 17.69

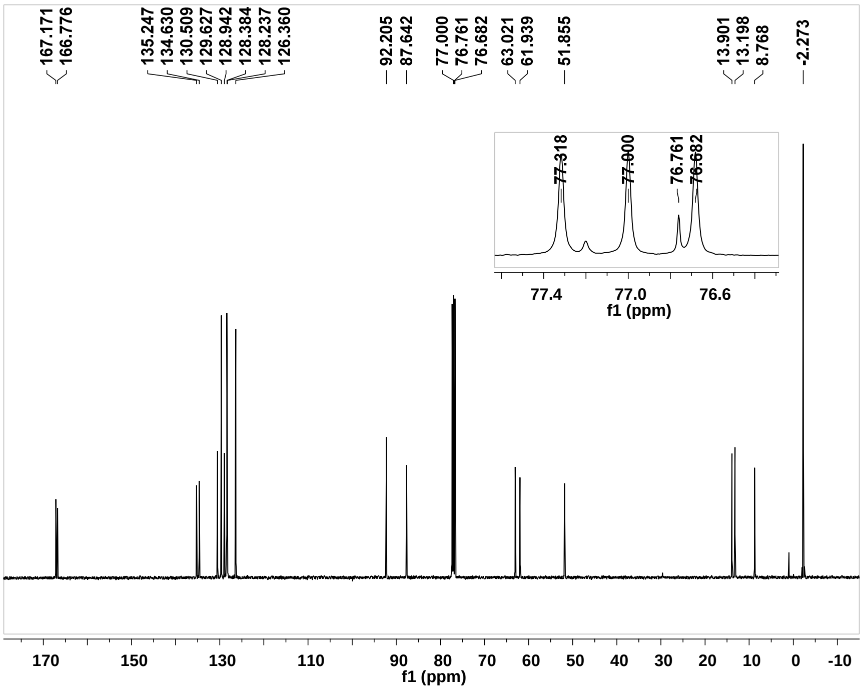

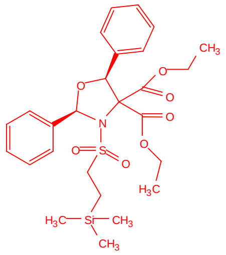

Current Data Parameters

F2 - Acquisition Parameters
DATE: 2015-05-28T00:58:52
PULPROG: zgpg30
TD: 32768
Solvent: CDCl3
NS: 1024
DS: undefined
SWH: 24038.5 Hz
AQ: undefined
TE: 295.7 C

======== CHANNEL f1 ========
NUC1: 13C
P1: 9.63 usec
SFO1: undefined MHz

F2 - Processing Parameters
SI: 65536
DC: 0.05
LB: 1.00 Hz
First Point: 0.50
FT: Hyper Quadrature
Phase: Manual
Ph0: -59.42
Ph1: 54.93

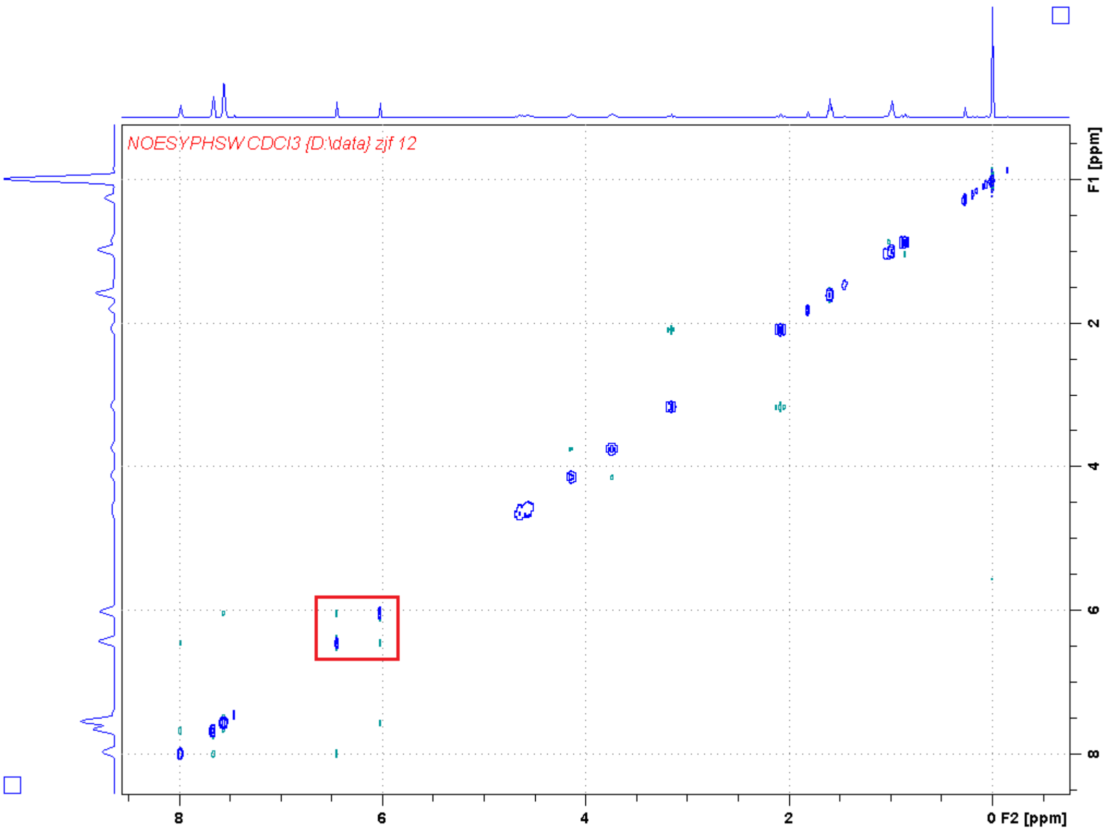
NOESYPHSW CDCl3 {D:\data} zjf 12
F1 [ppm]
F2 [ppm]
8
6
4
2
0

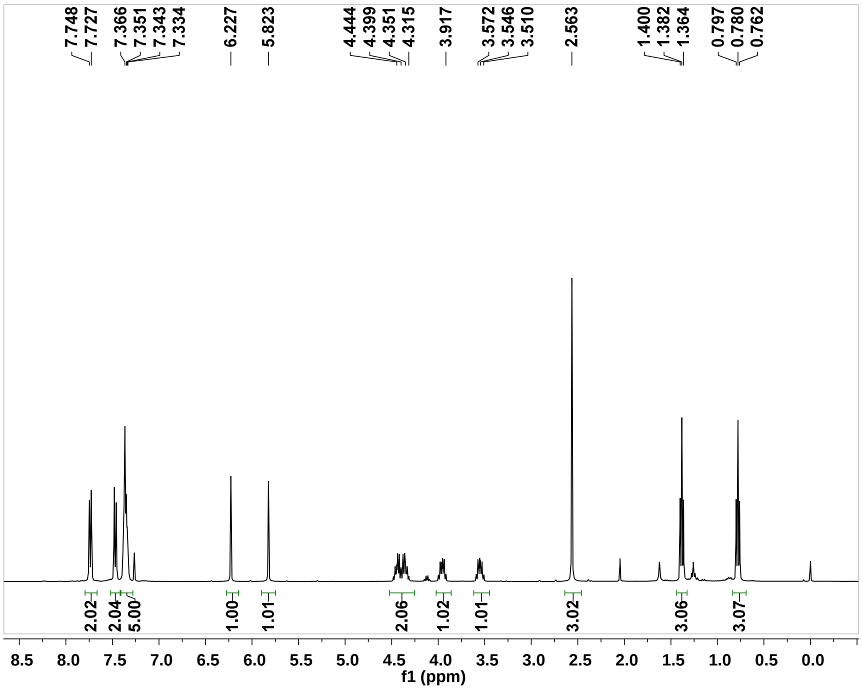

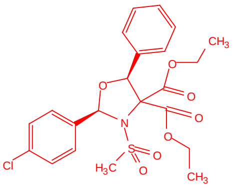

Current Data Parameters

F2 - Acquisition Parameters
DATE: 2015-02-16T14:41:27
PULPROG: zg30
TD: 32768
Solvent: CDCl3
NS: 32
DS: undefined
SWH: 8223.7 Hz
AQ: undefined
TE: 293.5 C

======== CHANNEL f1 ========
NUC1: 1H
P1: 9.93 usec
SFO1: undefined MHz

F2 - Processing Parameters
SI: 65536
DC: 0.05
LB: 0.30 Hz
First Point: 0.50
FT: Hyper Quadrature
Phase: Manual
Ph0: 96.72
Ph1: 17.55

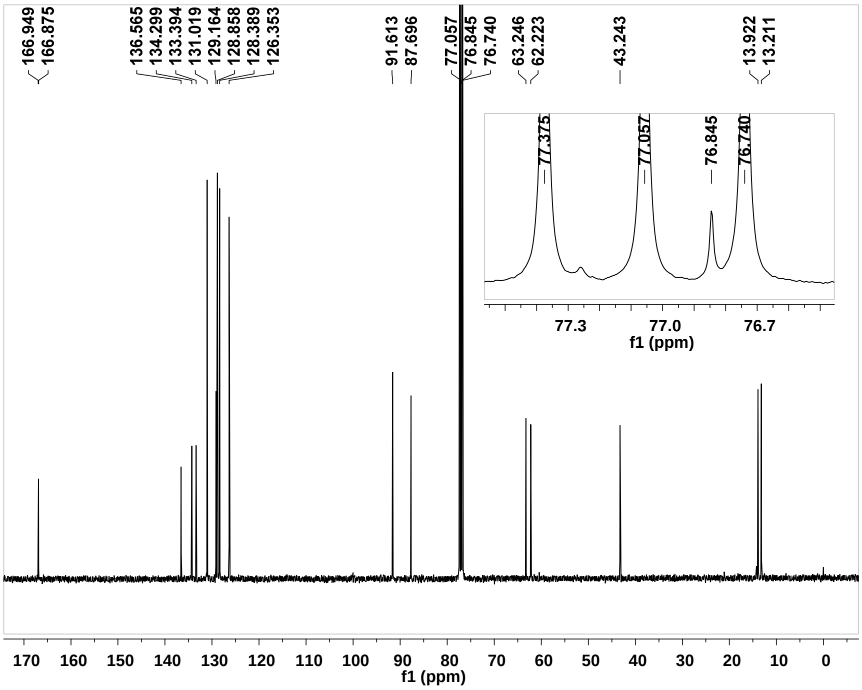

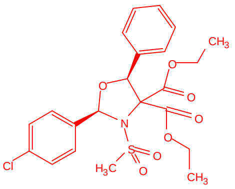

Current Data Parameters

F2 - Acquisition Parameters
DATE: 2015-02-16T16:31:57
PULPROG: zgpg30
TD: 32768
Solvent: CDCl3
NS: 1024
DS: undefined
SWH: 24038.5 Hz
AQ: undefined
TE: 293.9 C

======== CHANNEL f1 ========
NUC1: 13C
P1: 9.63 usec
SFO1: undefined MHz

F2 - Processing Parameters
SI: 65536
DC: 0.05
LB: 1.00 Hz
First Point: 0.50
FT: Hyper Quadrature
Phase: Manual
Ph0: -60.66
Ph1: 60.35

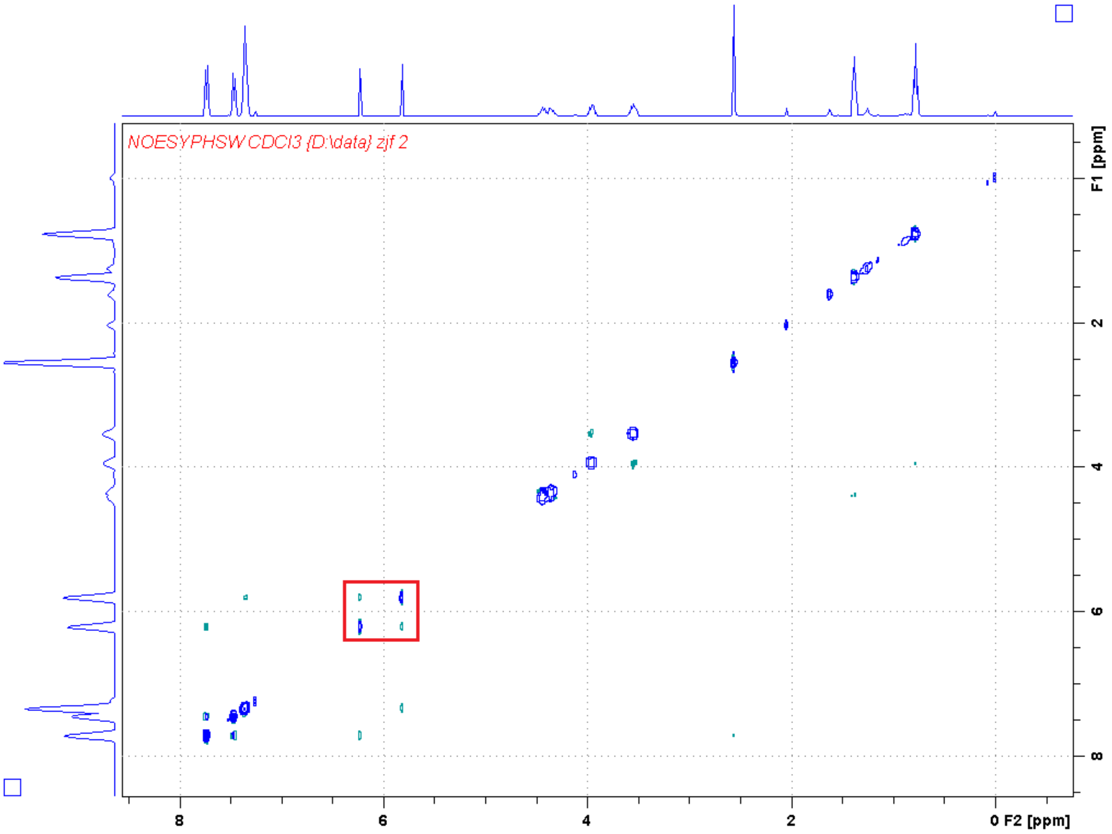

NOESYPHSW CDCl3 {D:\data} zjf 2
F1 [ppm]
2
4
6
8
8
6
4
2
0 F2 [ppm]

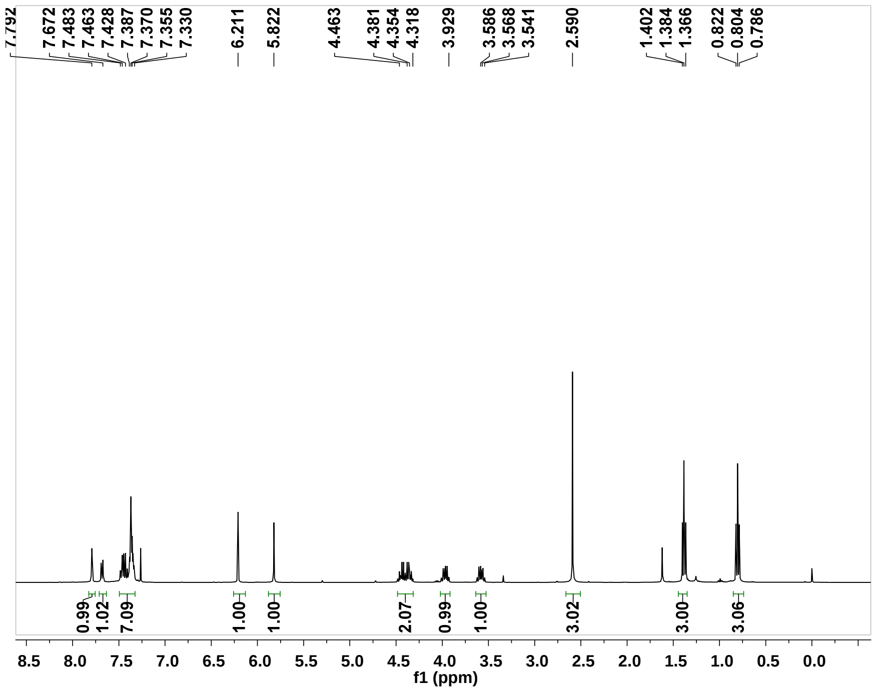

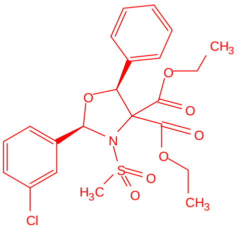

Current Data Parameters

F2 - Acquisition Parameters
DATE: 2015-04-02T10:05:50
PULPROG: zg30
TD: 32768
Solvent: CDCl3
NS: 32
DS: undefined
SWH: 8223.7 Hz
AQ: undefined
TE: 295.3 C

======== CHANNEL f 1 ========
NUC1: 1H
P1: 9.93 usec
SFO1: undefined MHz

F2 - Processing Parameters
SI: 65536
DC: 0.05
LB: 0.30 Hz
First Point: 0.50
FT: Hyper Quadrature
Phase: Manual
Ph0: 94.69
Ph1: 12.79

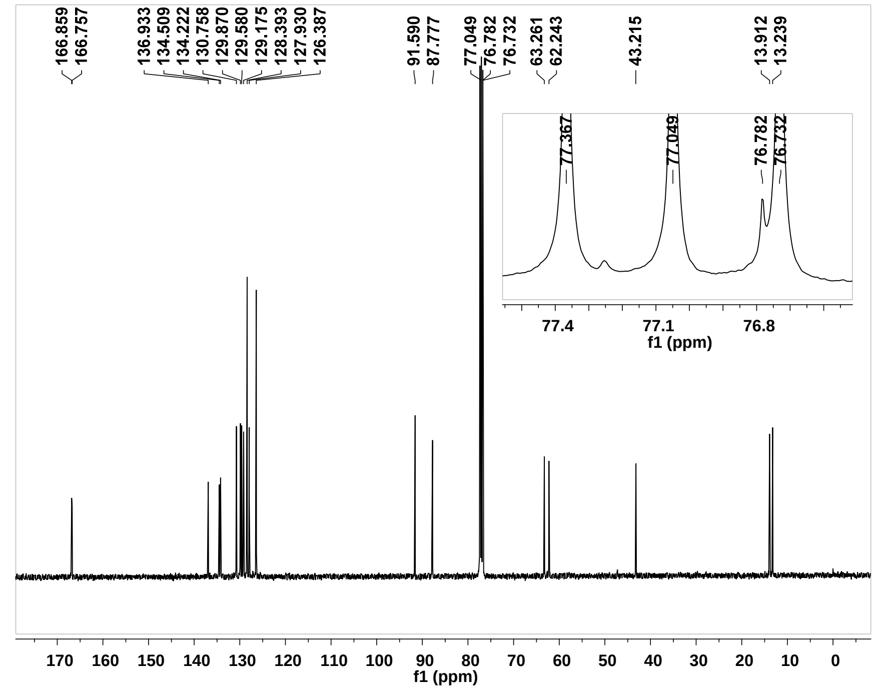

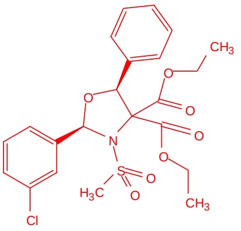

Current Data Parameters

F2 - Acquisition Parameters
DATE: 2015-04-02T11:56:19
PULPROG: zgpg30
TD: 32768
Solvent: CDCl3
NS: 1024
DS: undefined
SWH: 24038.5 Hz
AQ: undefined
TE: 295.7 C

======== CHANNEL f 1 ========
NUC1: 13C
P1: 9.63 usec
SFO1: undefined MHz

F2 - Processing Parameters
SI: 65536
DC: 0.05
LB: 1.00 Hz
First Point: 0.50
FT: Hyper Quadrature
Phase: Manual
Ph0: -59.59
Ph1: 50.89

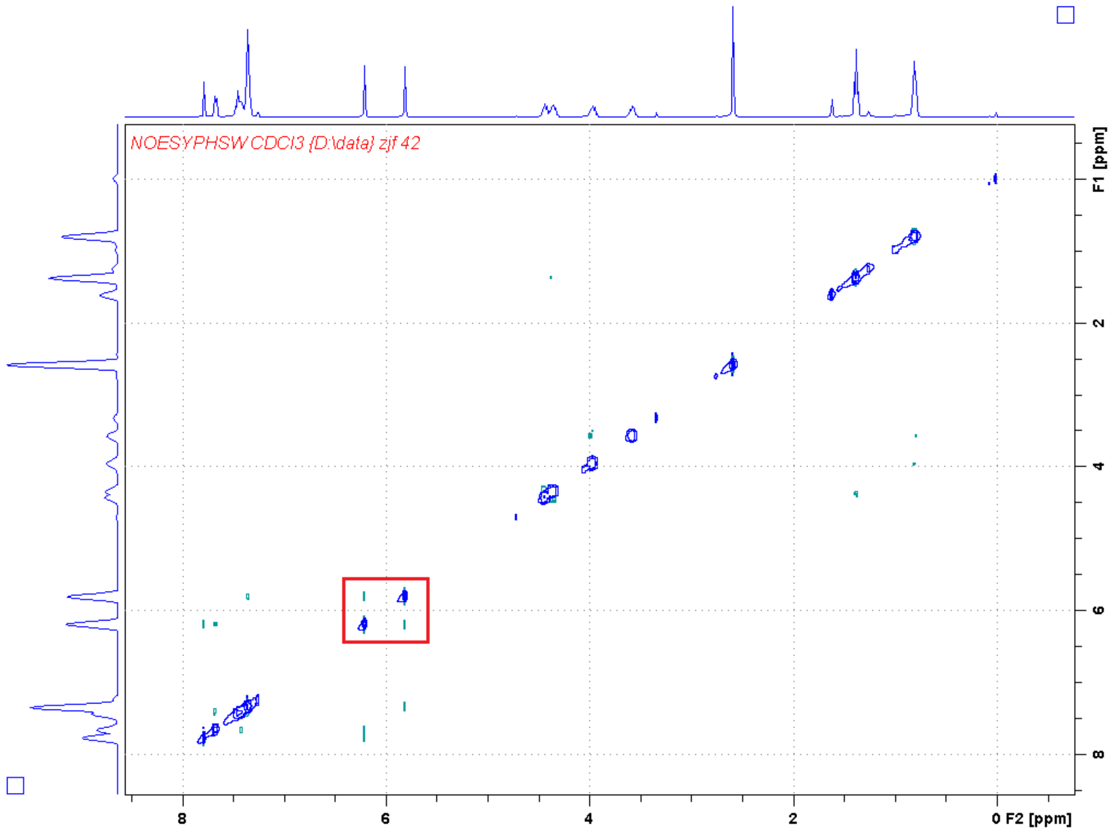

NOESYPHSW CDCl3 {D:\data} zjf 42
F1 [ppm]
8
6
4
2
0 F2 [ppm]

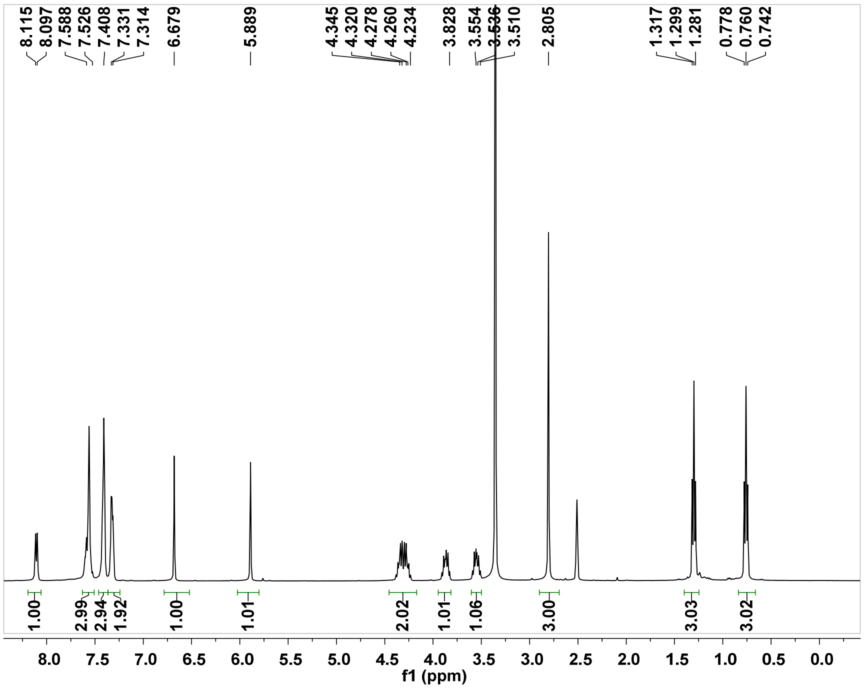

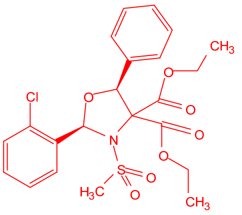

Current Data Parameters

F2 - Acquisition Parameters
DATE: 2015-04-28T11:06:21
PULPROG: zg30
TD: 32768
Solvent: DMSO
NS: 32
DS: undefined
SWH: 8223.7 Hz
AQ: undefined
TE: 297.8 C

======== CHANNEL f1 ========
NUC1: 1H
P1: 9.93 usec
SFO1: undefined MHz

F2 - Processing Parameters
SI: 65536
DC: 0.05
LB: 0.30 Hz
First Point: 0.50
FT: Hyper Quadrature
Phase: Manual
Ph0: 86.99
Ph1: 12.57

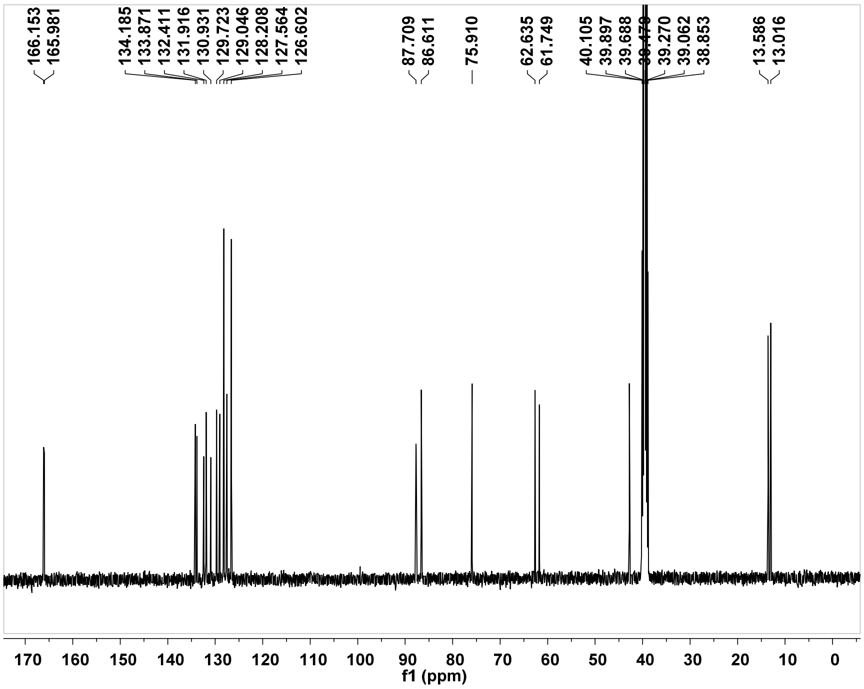

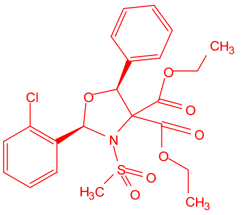

Current Data Parameters

F2 - Acquisition Parameters
DATE: 2015-04-28T12:16:16
PULPROG: zgpg30
TD: 32768
Solvent: DMSO
NS: 1024
DS: undefined
SWH: 24038.5 Hz
AQ: undefined
TE: 297.5 C

======== CHANNEL f1 ========
NUC1: 13C
P1: 9.63 usec
SFO1: undefined MHz

F2 - Processing Parameters
SI: 65536
DC: 0.05
LB: 1.00 Hz
First Point: 0.50
FT: Hyper Quadrature
Phase: Manual
Ph0: -66.51
Ph1: 63.87

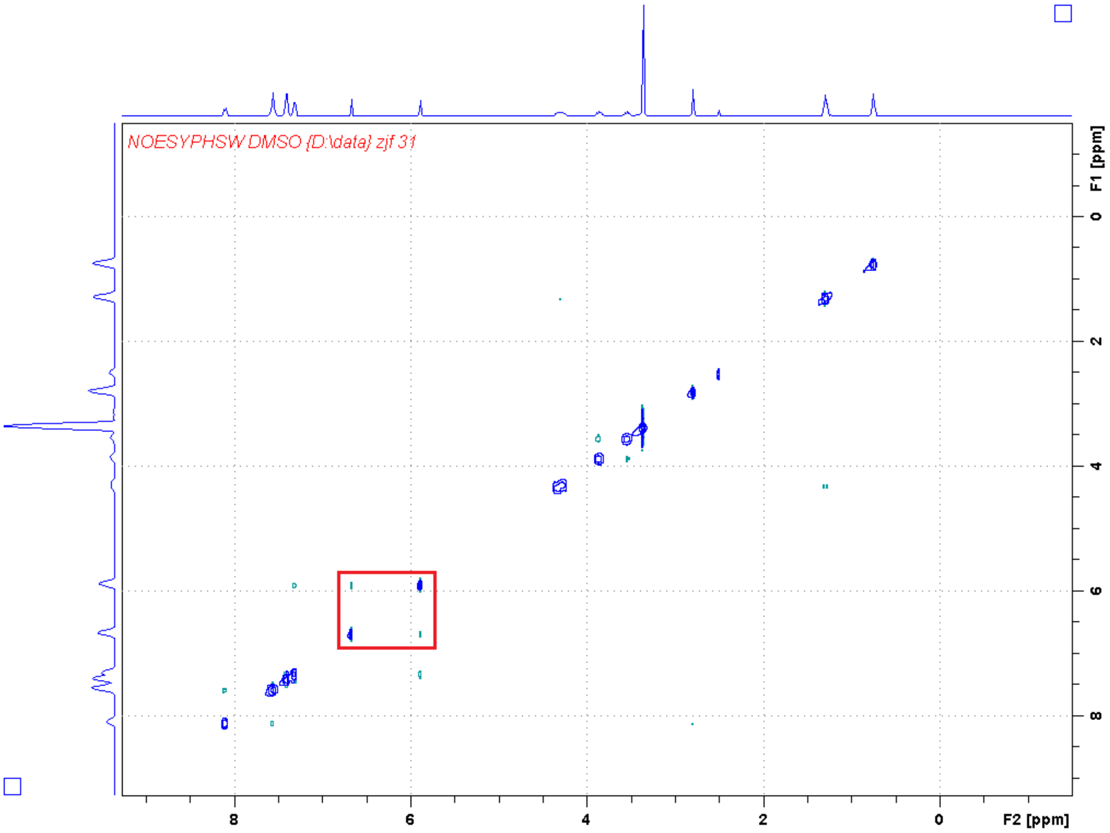

NOESYPHSW DMSO {D:\data} zjf 31
F1 [ppm]
0
2
4
6
8
8
6
4
2
0
F2 [ppm]

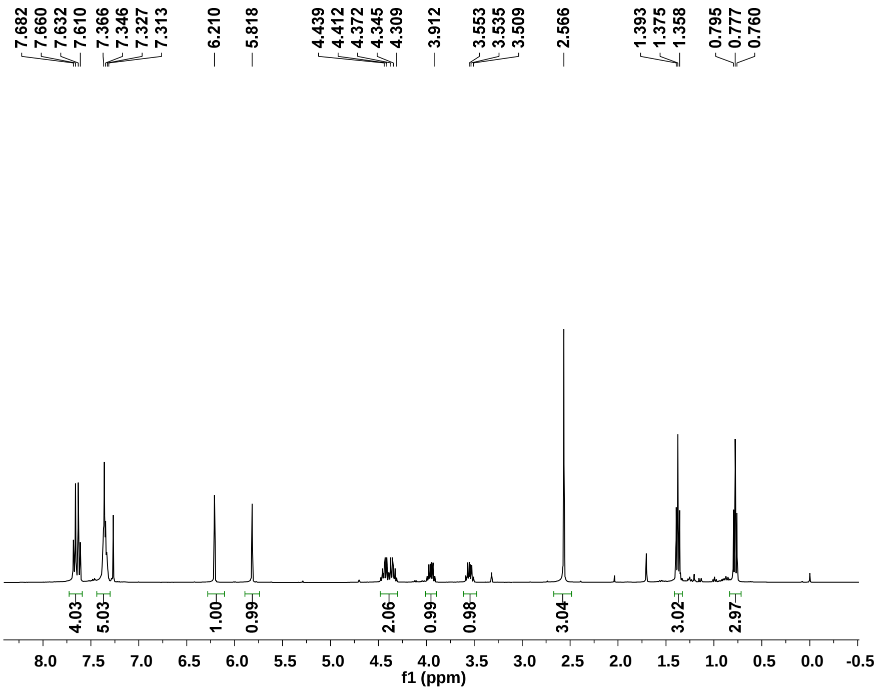

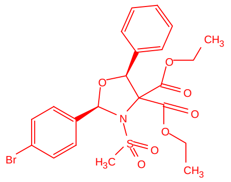

Current Data Parameters

F2 - Acquisition Parameters
DATE: 2015-07-15T05:15:33
PULPROG: zg30
TD: 32768
Solvent: CDCl3
NS: 16
DS: undefined
SWH: 8223.7 Hz
AQ: undefined
TE: 0 C

======== CHANNEL f1 ========
NUC1: 1H
P1: 9.93 usec
SFO1: undefined MHz

F2 - Processing Parameters
SI: 65536
DC: 0.05
LB: 0.30 Hz
First Point: 0.50
FT: Hyper Quadrature
Phase: Manual
Ph0: 94.75
Ph1: 15.88

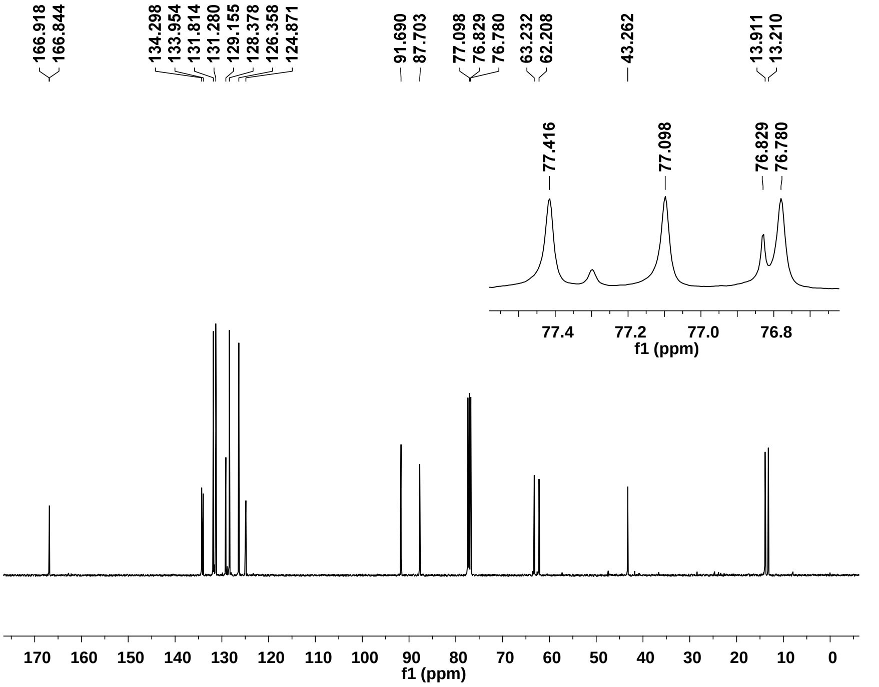

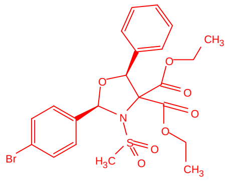

Current Data Parameters

F2 - Acquisition Parameters
DATE: 2015-07-15T07:11:07
PULPROG: zgpg30
TD: 32768
Solvent: CDCl3
NS: 1024
DS: undefined
SWH: 24038.5 Hz
AQ: undefined
TE: 0 C

======== CHANNEL f1 ========
NUC1: 13C
P1: 9.63 usec
SFO1: undefined MHz

F2 - Processing Parameters
SI: 65536
DC: 0.05
LB: 1.00 Hz
First Point: 0.50
FT: Hyper Quadrature
Phase: Manual
Ph0: -58.39
Ph1: 63.44

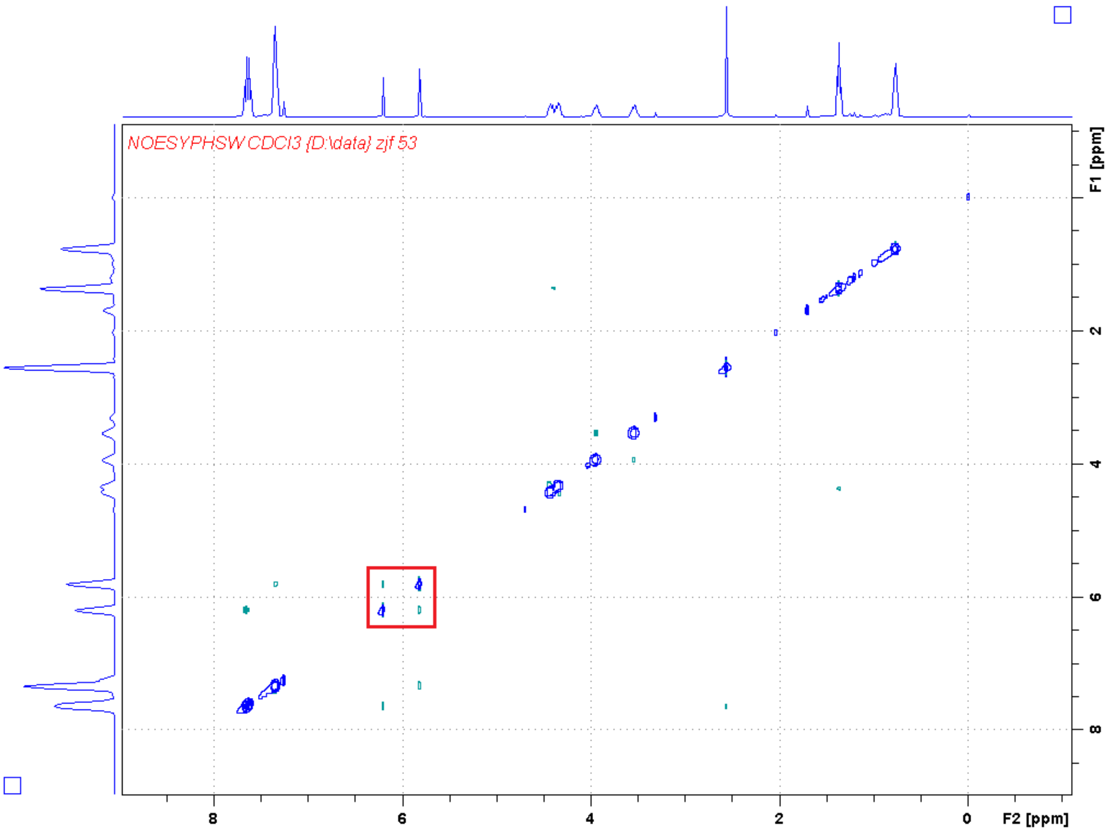

NOESYPHSW CDCl3 {D:\data} zjf 53
F1 [ppm]
F2 [ppm]

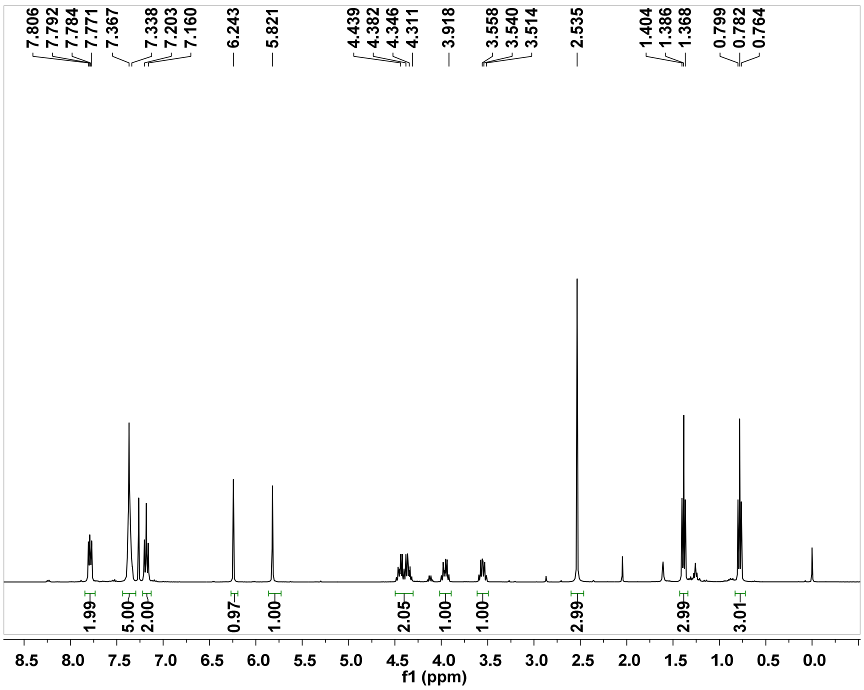

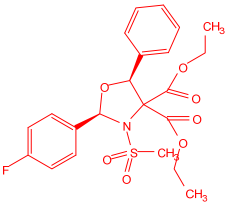

Current Data Parameters

F2 - Acquisition Parameters
DATE: 2015-02-16T20:32:08
PULPROG: zg30
TD: 32768
Solvent: CDCl3
NS: 32
DS: undefined
SWH: 8223.7 Hz
AQ: undefined
TE: 293.5 C

======== CHANNEL f1 ========
NUC1: 1H
P1: 9.93 usec
SFO1: undefined MHz

F2 - Processing Parameters
SI: 65536
DC: 0.05
LB: 0.30 Hz
First Point: 0.50
FT: Hyper Quadrature
Phase: Manual
Ph0: 96.64
Ph1: 17.09

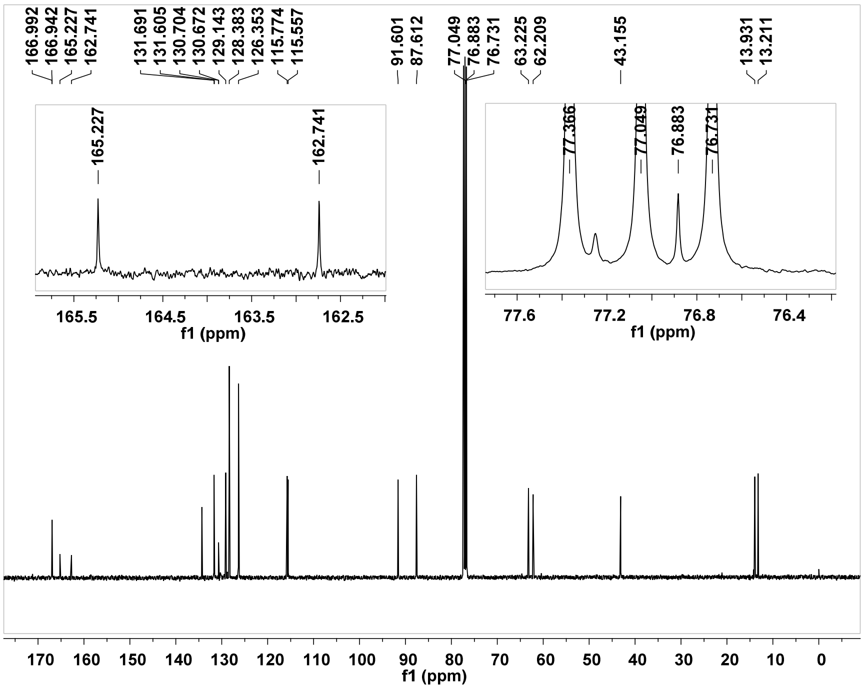

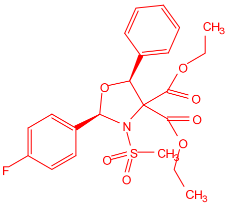

Current Data Parameters

F2 - Acquisition Parameters
DATE: 2015-02-16T22:22:39
PULPROG: zgpg30
TD: 32768
Solvent: CDCl3
NS: 1024
DS: undefined
SWH: 24038.5 Hz
AQ: undefined
TE: 293.8 C

======== CHANNEL f1 ========
NUC1: 13C
P1: 9.63 usec
SFO1: undefined MHz

F2 - Processing Parameters
SI: 65536
DC: 0.05
LB: 1.00 Hz
First Point: 0.50
FT: Hyper Quadrature
Phase: Manual
Ph0: -72.22
Ph1: 64.41

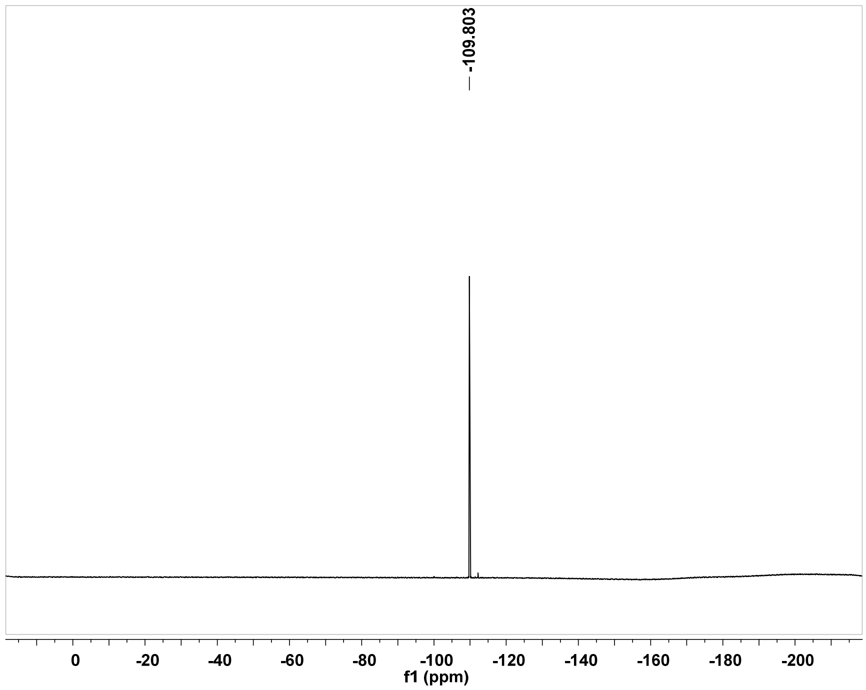

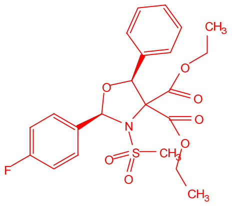

Current Data Parameters

F2 - Acquisition Parameters
DATE: 2015-02-16T17:48:23
PULPROG: zgflqn
TD: 65536
Solvent: CDCl3
NS: 16
DS: undefined
SWH: 89285.7 Hz
AQ: undefined
TE: 293.6 C

======== CHANNEL f1 ========
NUC1: 19F
P1: 12.75 usec
SFO1: undefined MHz

F2 - Processing Parameters
SI: 131072
DC: 0.05
LB: 0.30 Hz
First Point: 0.50
FT: Hyper Quadrature
Phase: Manual
Ph0: -30.79
Ph1: 0.00

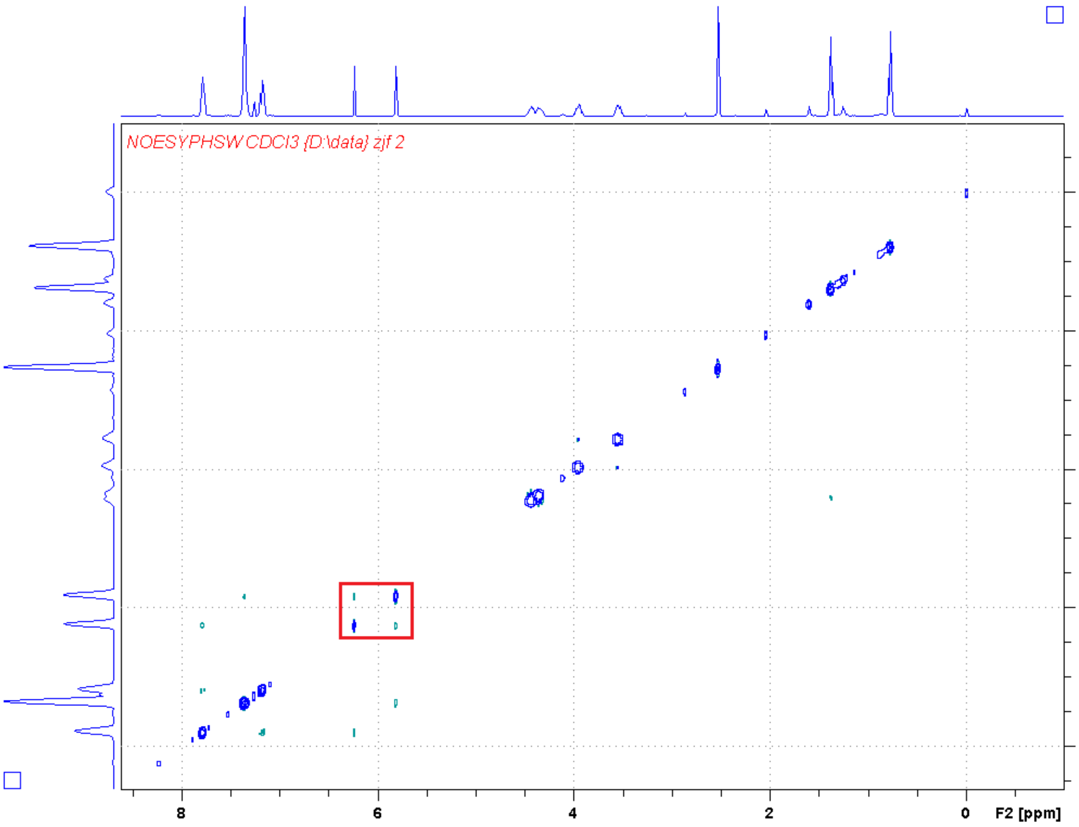

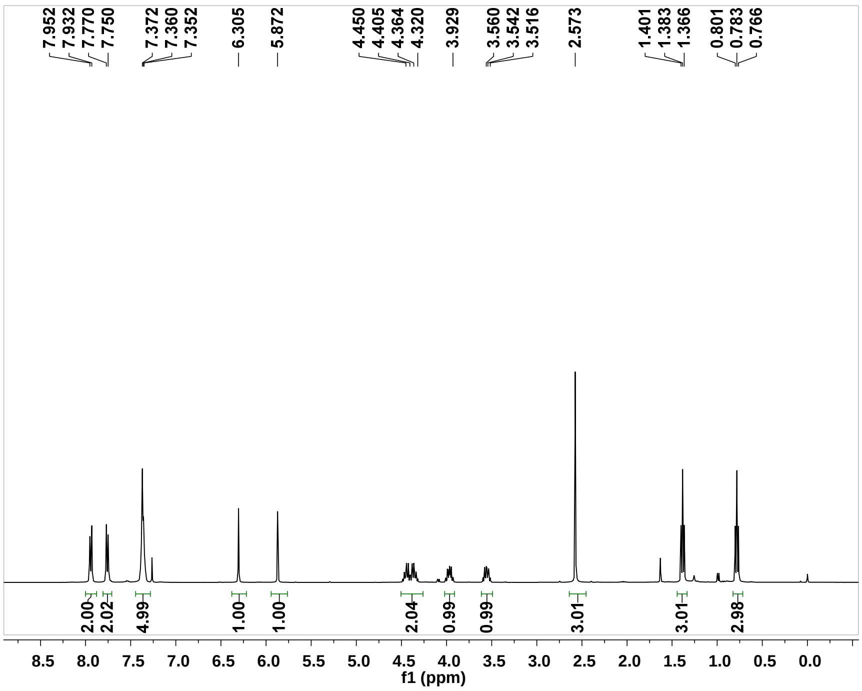

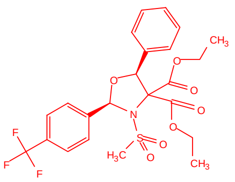

Current Data Parameters

F2 - Acquisition Parameters
DATE: 2015-04-14T01:47:38
PULPROG: zg30
TD: 32768
Solvent: CDCl3
NS: 32
DS: undefined
SWH: 8223.7 Hz
AQ: undefined
TE: 294.5 C

======== CHANNEL f1 ========
NUC1: 1H
P1: 9.93 usec
SFO1: undefined MHz

F2 - Processing Parameters
SI: 65536
DC: 0.05
LB: 0.30 Hz
First Point: 0.50
FT: Hyper Quadrature
Phase: Manual
Ph0: 97.61
Ph1: 16.82

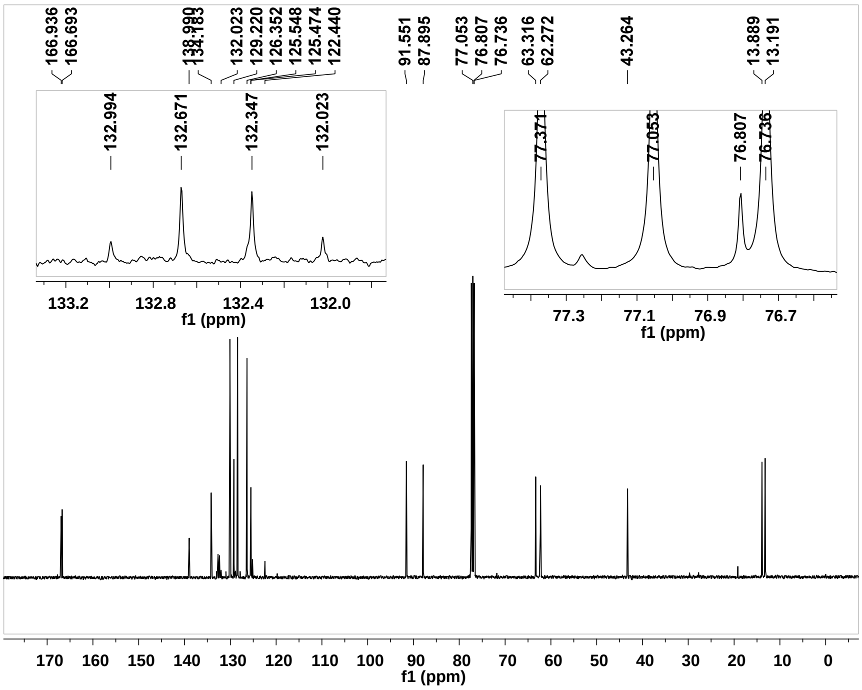

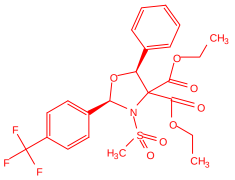

Current Data Parameters

F2 - Acquisition Parameters
DATE: 2015-04-14T03:39:53
PULPROG: zgpg30
TD: 32768
Solvent: CDCl3
NS: 1024
DS: undefined
SWH: 24038.5 Hz
AQ: undefined
TE: 294.7 C

======== CHANNEL f1 ========
NUC1: 13C
P1: 9.63 usec
SFO1: undefined MHz

F2 - Processing Parameters
SI: 65536
DC: 0.05
LB: 1.00 Hz
First Point: 0.50
FT: Hyper Quadrature
Phase: Manual
Ph0: -64.26
Ph1: 62.28

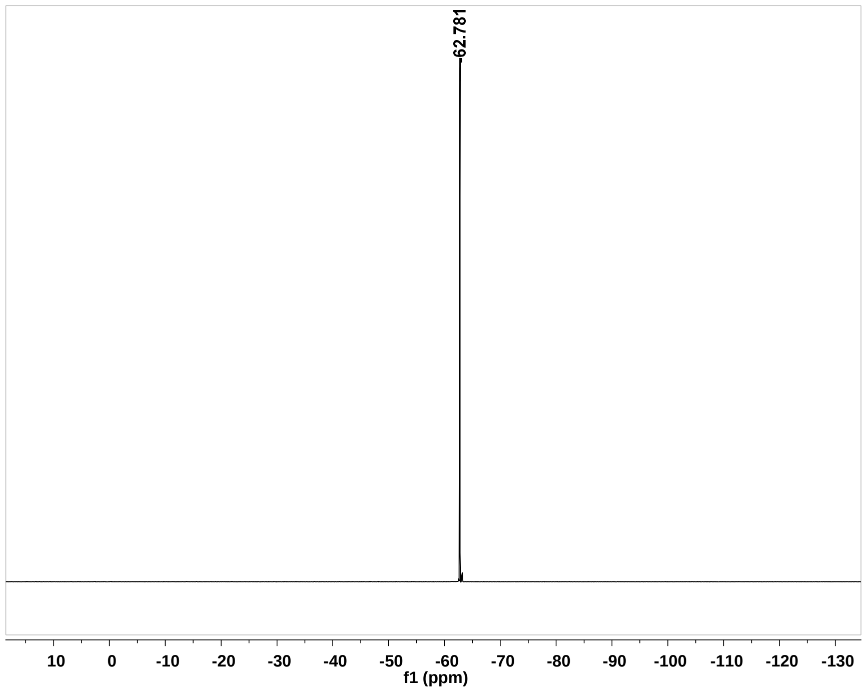

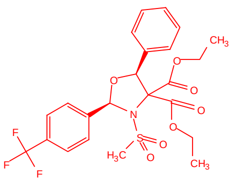

Current Data Parameters

F2 - Acquisition Parameters
DATE: 2015-03-20T00:27:32
PULPROG: zgflqn
TD: 65536
Solvent: CDCl3
NS: 16
DS: undefined
SWH: 89285.7 Hz
AQ: undefined
TE: 294.2 C

======== CHANNEL f1 ========
NUC1: 19F
P1: 12.75 usec
SFO1: undefined MHz

F2 - Processing Parameters
SI: 131072
DC: 0.05
LB: 0.30 Hz
First Point: 0.50
FT: Hyper Quadrature
Phase: Manual
Ph0: -115.49
Ph1: 0.00

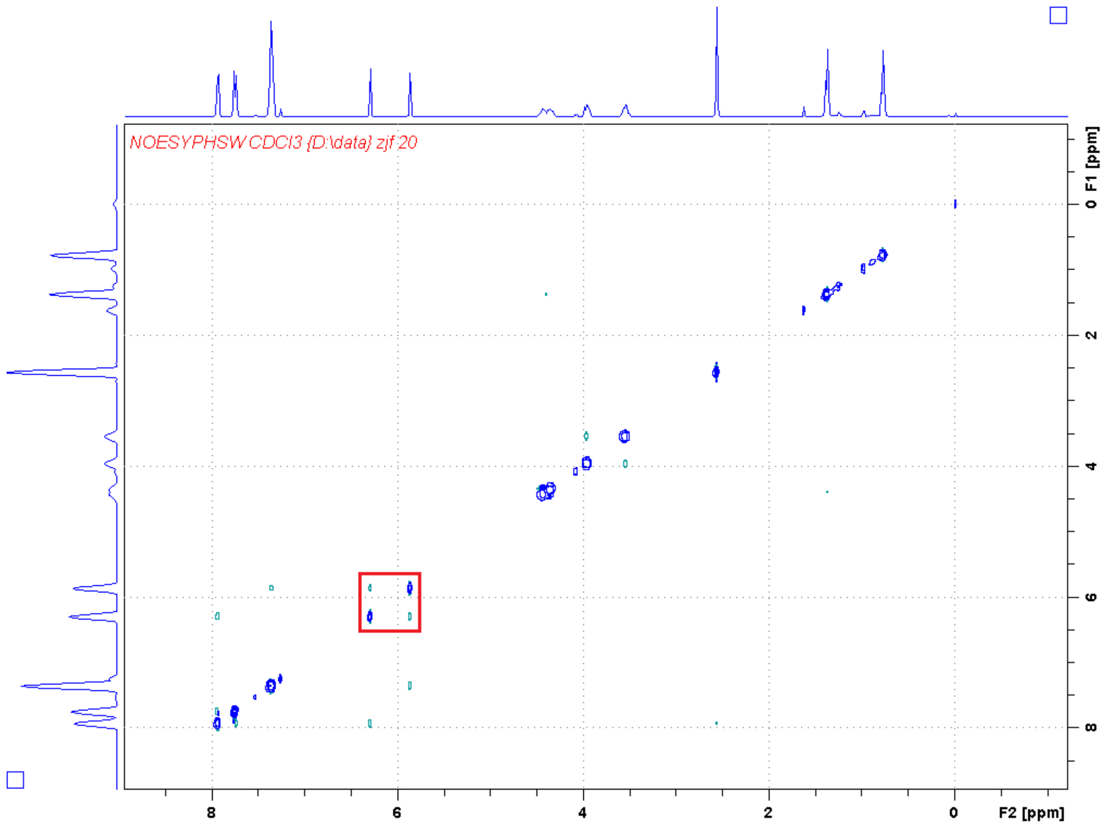

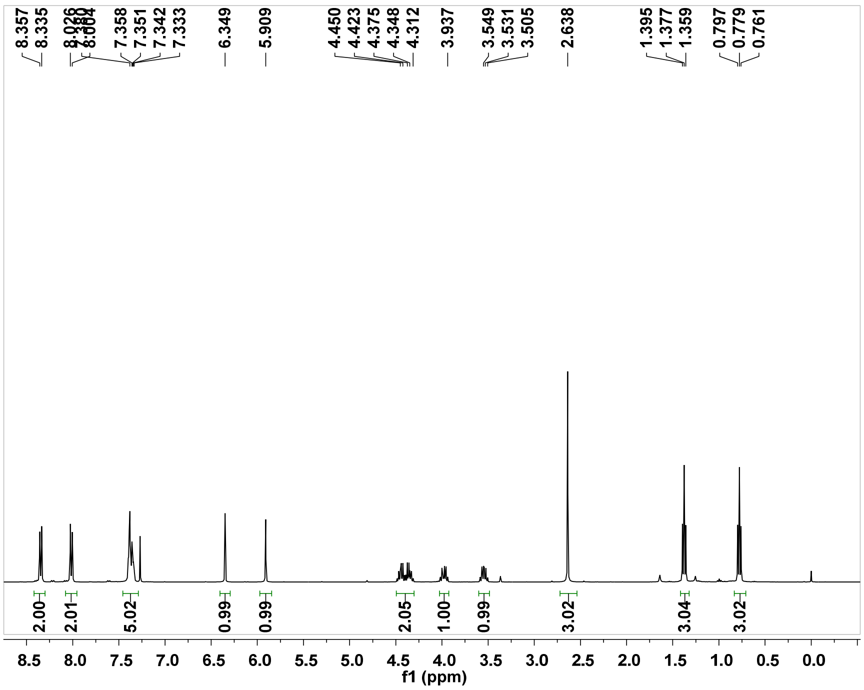

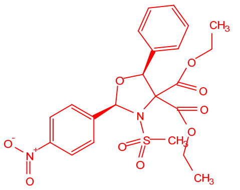

Current Data Parameters

F2 - Acquisition Parameters
DATE: 2015-03-20T18:24:57
PULPROG: zg30
TD: 32768
Solvent: CDCl3
NS: 32
DS: undefined
SWH: 8223.7 Hz
AQ: undefined
TE: 294.2 C

======== CHANNEL f1 ========
NUC1: 1H
P1: 9.93 usec
SFO1: undefined MHz

F2 - Processing Parameters
SI: 65536
DC: 0.05
LB: 0.30 Hz
First Point: 0.50
FT: Hyper Quadrature
Phase: Manual
Ph0: 98.02
Ph1: 17.09

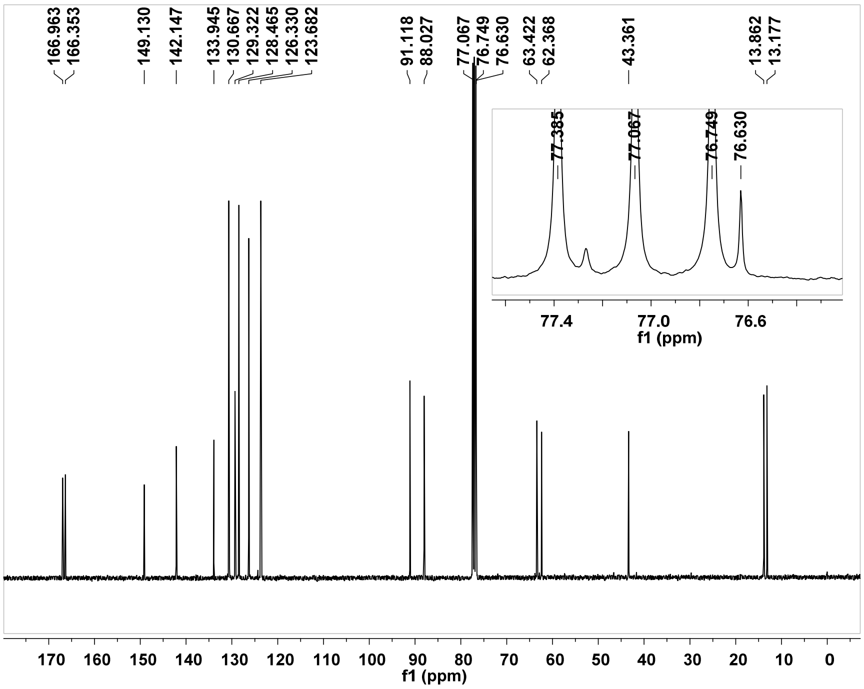

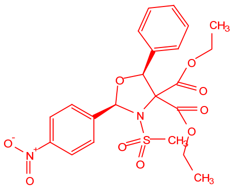

Current Data Parameters

F2 - Acquisition Parameters
DATE: 2015-03-20T19:24:25
PULPROG: zgpg30
TD: 32768
Solvent: CDCl3
NS: 1024
DS: undefined
SWH: 24038.5 Hz
AQ: undefined
TE: 294.5 C

======== CHANNEL f1 ========
NUC1: 13C
P1: 9.63 usec
SFO1: undefined MHz

F2 - Processing Parameters
SI: 65536
DC: 0.05
LB: 1.00 Hz
First Point: 0.50
FT: Hyper Quadrature
Phase: Manual
Ph0: -63.21
Ph1: 64.34

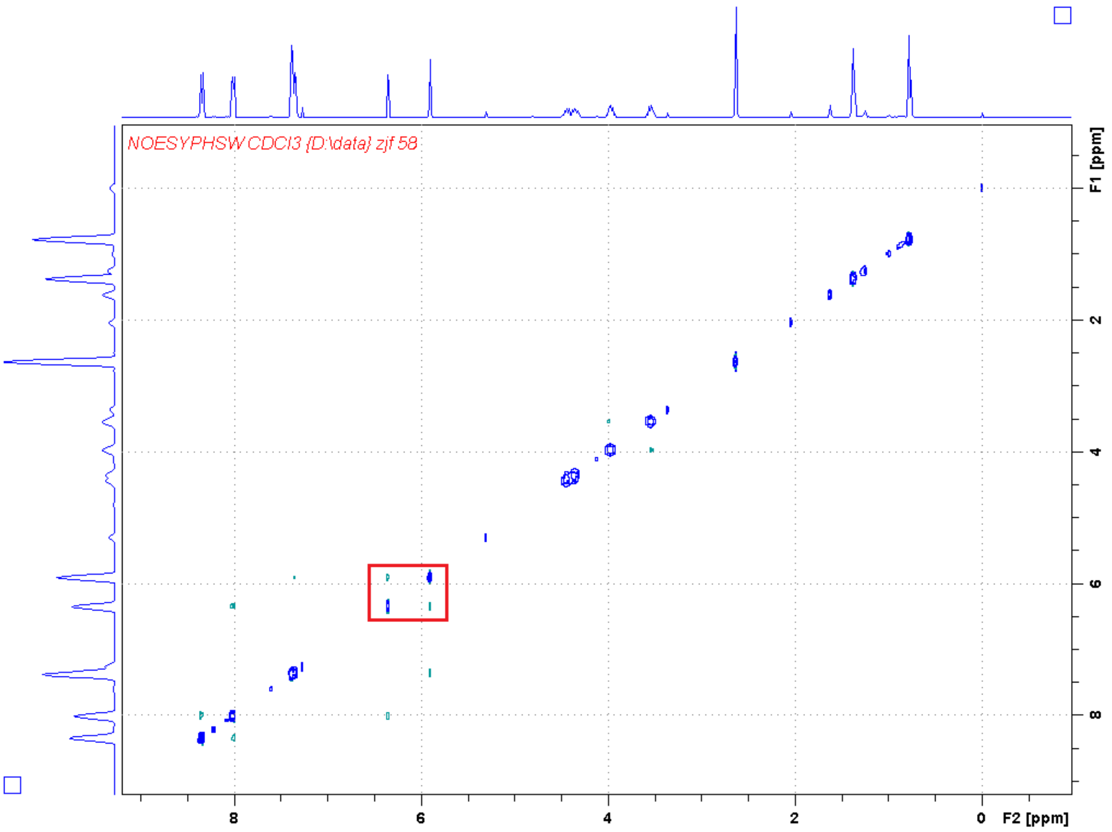
NOESYPHSW CDCl3 {D:\data} zjf 58
F1 [ppm]
F2 [ppm]
8
6
4
2
0

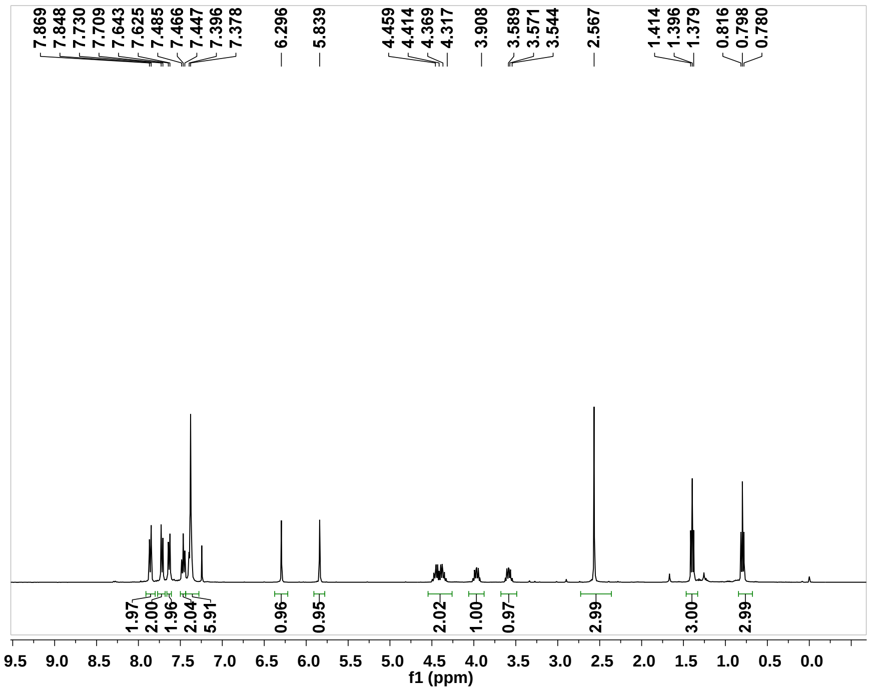

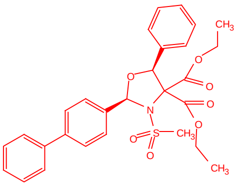

Current Data Parameters

F2 - Acquisition Parameters
DATE: 2015-03-13T17:19:41
PULPROG: zg30
TD: 32768
Solvent: CDCl3
NS: 4
DS: undefined
SWH: 8223.7 Hz
AQ: undefined
TE: 294.1 C

======== CHANNEL f 1 ========
NUC1: 1H
P1: 9.93 usec
SFO1: undefined MHz

F2 - Processing Parameters
SI: 65536
DC: 0.05
LB: 0.30 Hz
First Point: 0.50
FT: Hyper Quadrature
Phase: Manual
Ph0: 95.30
Ph1: 14.78

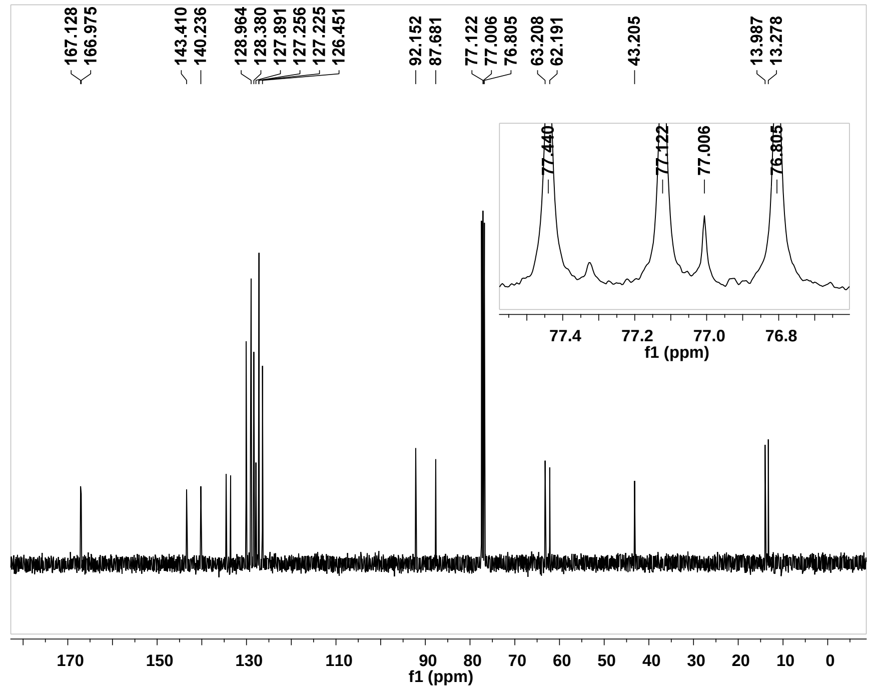

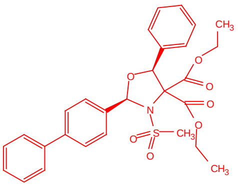

Current Data Parameters

F2 - Acquisition Parameters
DATE: 2015-03-13T17:21:36
PULPROG: zgpg30
TD: 32768
Solvent: CDCl3
NS: 16
DS: undefined
SWH: 24038.5 Hz
AQ: undefined
TE: 294.2 C

======== CHANNEL f 1 ========
NUC1: 13C
P1: 9.63 usec
SFO1: undefined MHz

F2 - Processing Parameters
SI: 65536
DC: 0.05
LB: 1.00 Hz
First Point: 0.50
FT: Hyper Quadrature
Phase: Manual
Ph0: -58.49
Ph1: 53.86

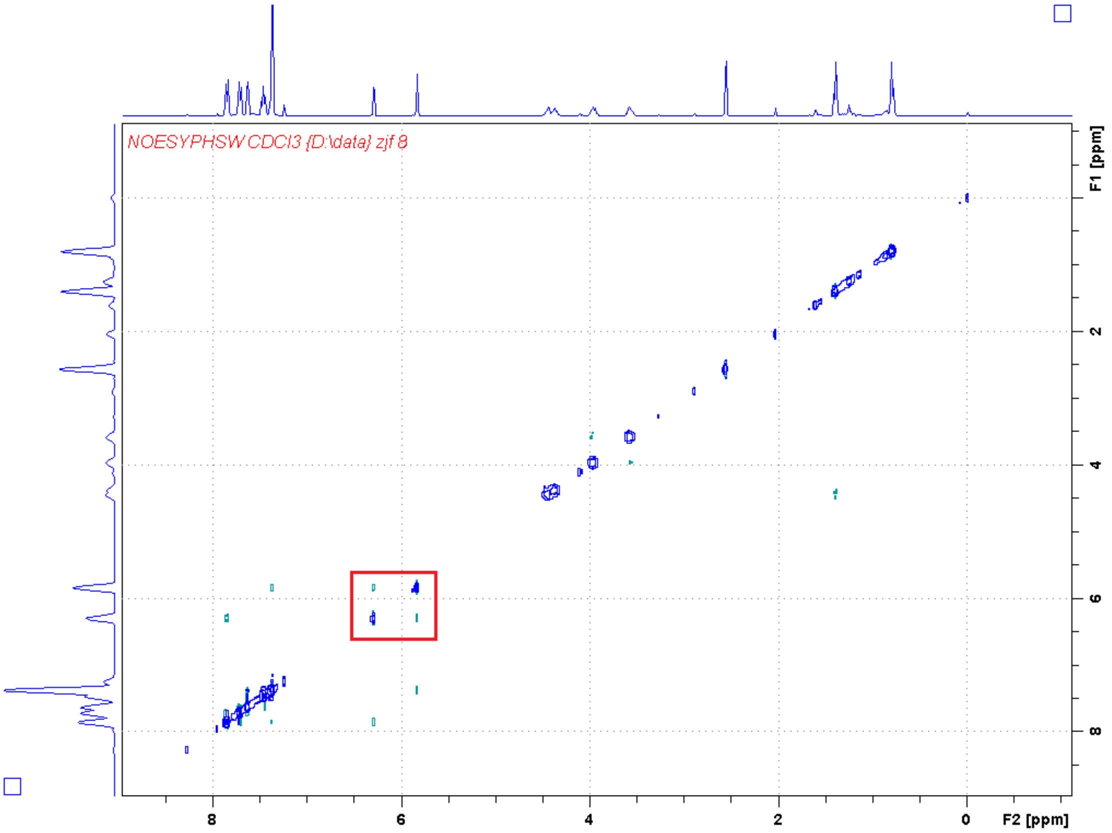
NOESYPHSW CDCl3 {D:\data} zjf 8
F1 [ppm]
F2 [ppm]
8
6
4
2
0

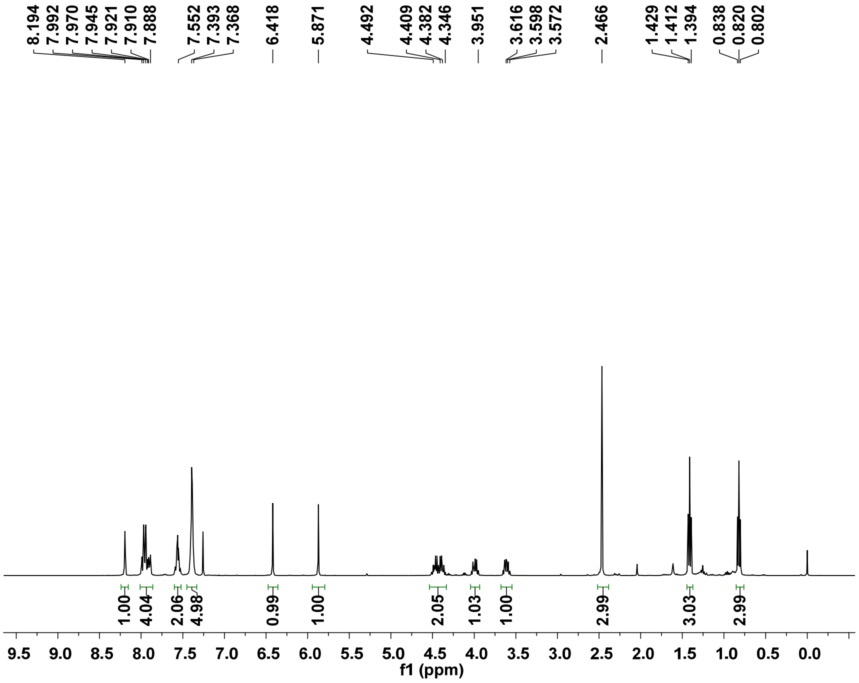

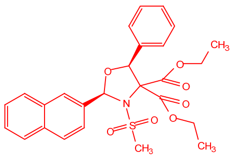

Current Data Parameters

F2 - Acquisition Parameters
DATE: 2015-02-03T00:08:14
PULPROG: zg30
TD: 32768
Solvent: CDCl3
NS: 32
DS: undefined
SWH: 8223.7 Hz
AQ: undefined
TE: 293.9 C

======== CHANNEL f1 ========
NUC1: 1H
P1: 9.93 usec
SFO1: undefined MHz

F2 - Processing Parameters
SI: 65536
DC: 0.05
LB: 0.30 Hz
First Point: 0.50
FT: Hyper Quadrature
Phase: Manual
Ph0: 95.35
Ph1: 18.03

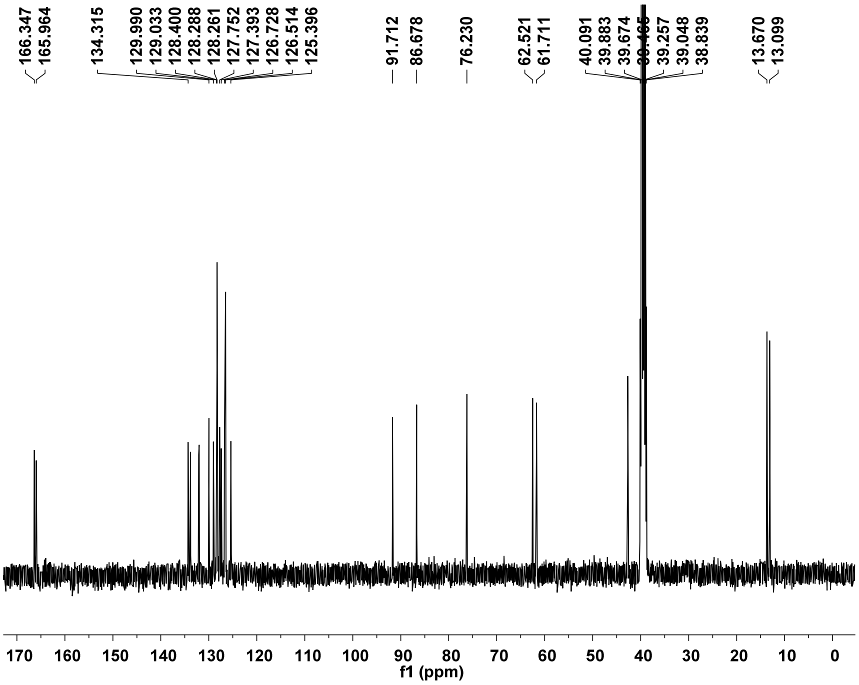

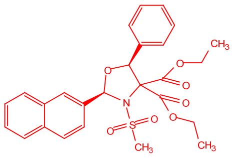

Current Data Parameters

F2 - Acquisition Parameters
DATE: 2015-05-31T04:19:54
PULPROG: zgpg30
TD: 32768
Solvent: DMSO
NS: 256
DS: undefined
SWH: 24038.5 Hz
AQ: undefined
TE: 296.1 C

======== CHANNEL f1 ========
NUC1: 13C
P1: 9.63 usec
SFO1: undefined MHz

F2 - Processing Parameters
SI: 65536
DC: 0.05
LB: 1.00 Hz
First Point: 0.50
FT: Hyper Quadrature
Phase: Manual
Ph0: -64.21
Ph1: 65.76

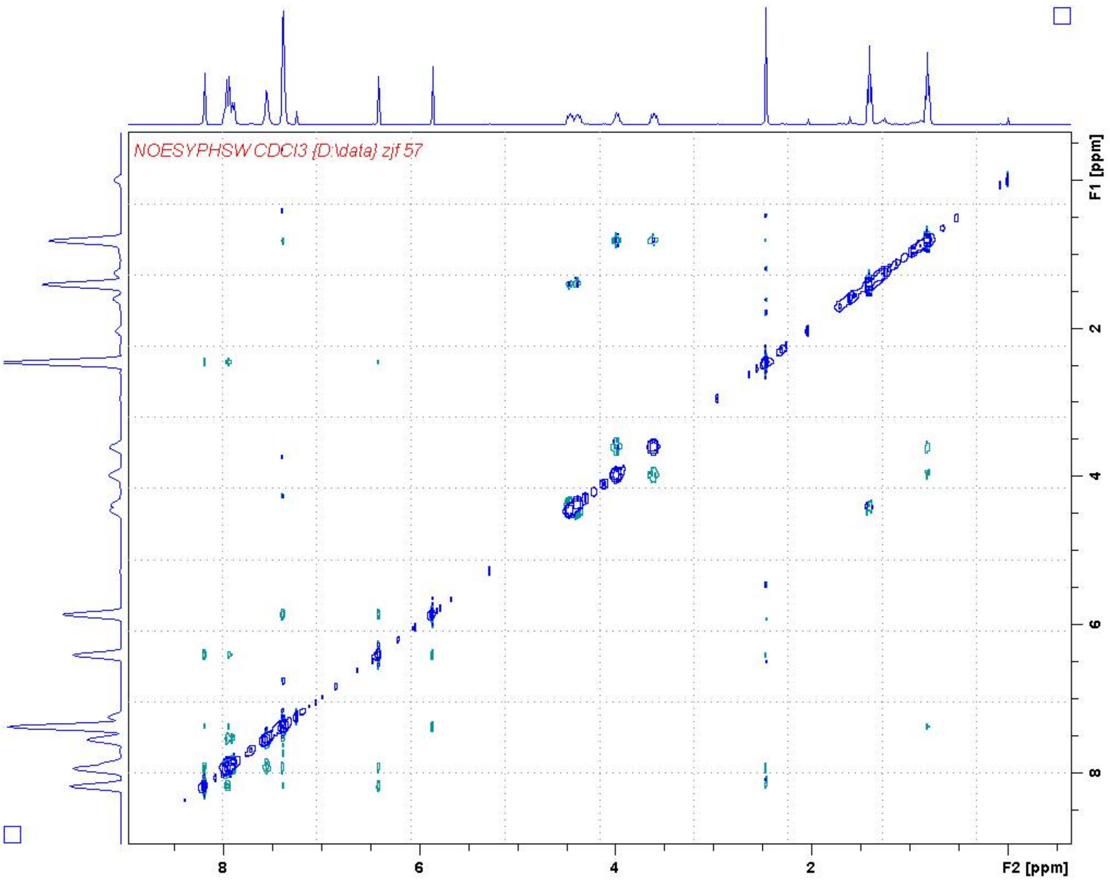

NOESYPHSW CDCl3 {D:\data} zjf 57
F1 [ppm]
F2 [ppm]
8
6
4
2
2
4
6
8

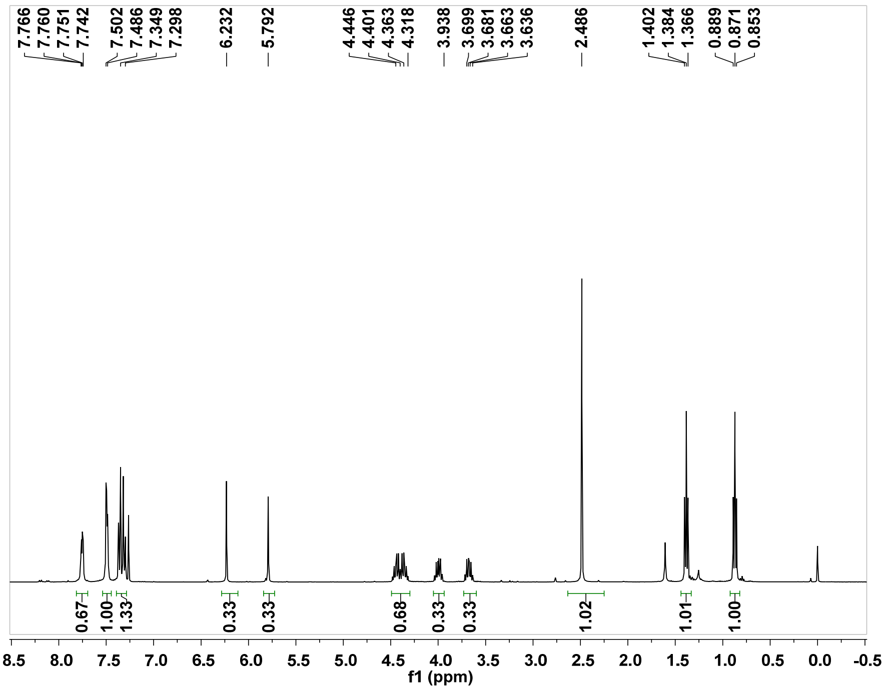

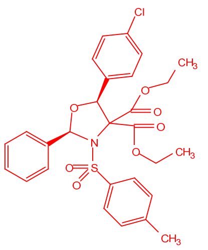

Current Data Parameters

F2 - Acquisition Parameters
DATE: 2015-03-30T11:12:10
PULPROG: zg30
TD: 32768
Solvent: CDCl3
NS: 32
DS: undefined
SWH: 8223.7 Hz
AQ: undefined
TE: 294.6 C

======== CHANNEL f1 ========
NUC1: 1H
P1: 9.93 usec
SFO1: undefined MHz

F2 - Processing Parameters
SI: 65536
DC: 0.05
LB: 0.30 Hz
First Point: 0.50
FT: Hyper Quadrature
Phase: Manual
Ph0: 98.45
Ph1: 16.28

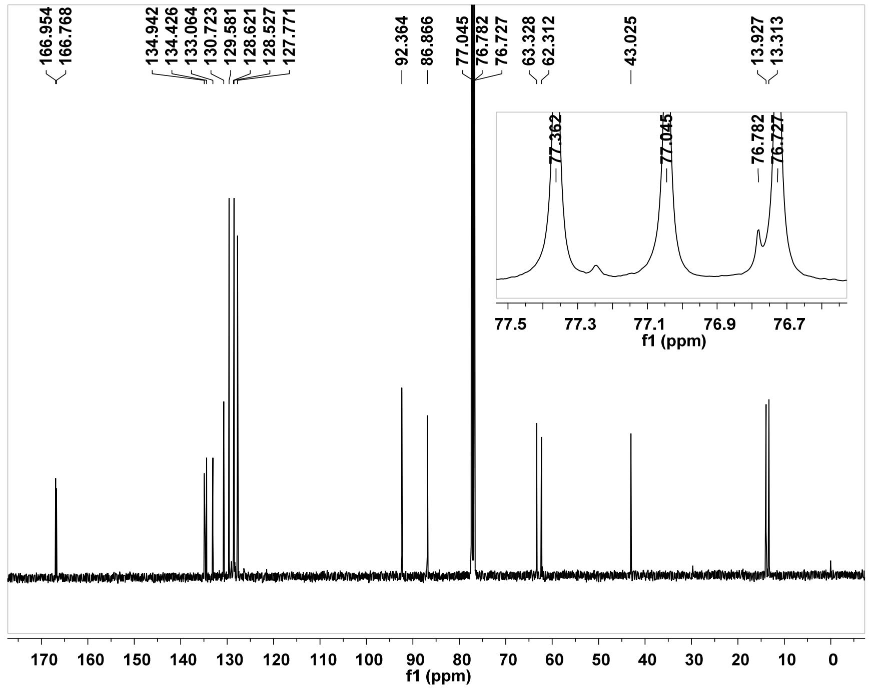

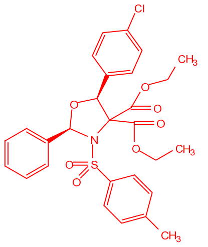

Current Data Parameters

F2 - Acquisition Parameters
DATE: 2015-03-30T13:02:57
PULPROG: zgpg30
TD: 32768
Solvent: CDCl3
NS: 1024
DS: undefined
SWH: 24038.5 Hz
AQ: undefined
TE: 295.1 C

======== CHANNEL f1 ========
NUC1: 13C
P1: 9.63 usec
SFO1: undefined MHz

F2 - Processing Parameters
SI: 65536
DC: 0.05
LB: 1.00 Hz
First Point: 0.50
FT: Hyper Quadrature
Phase: Manual
Ph0: -66.63
Ph1: 56.62

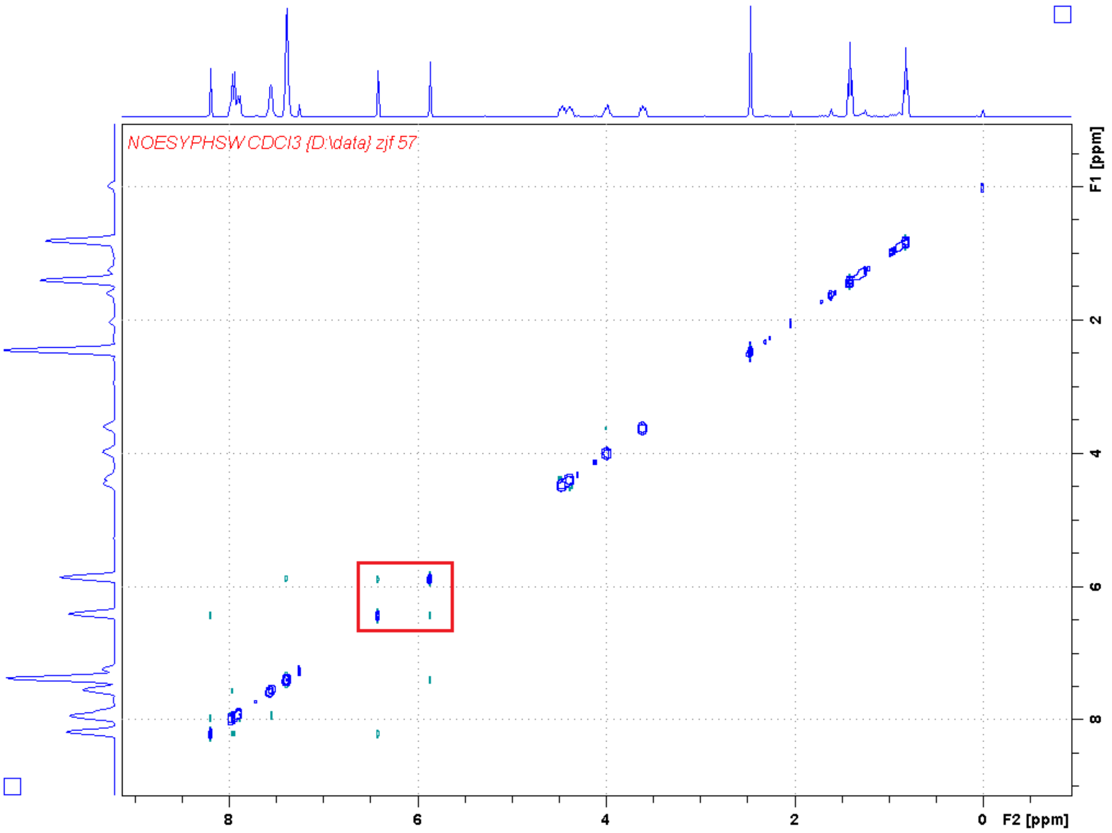

NOESYPHSW CDCl3 {D:\data} zjf 57
F1 [ppm]
F2 [ppm]
8
6
4
2
0

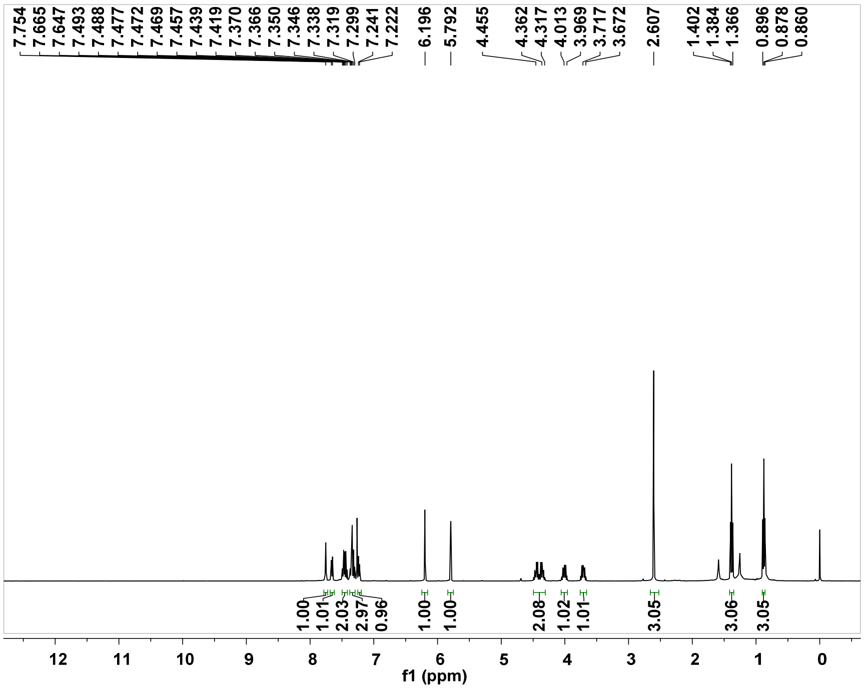

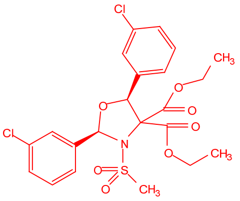

Current Data Parameters

F2 - Acquisition Parameters
DATE: 2015-05-05T18:08:30
PULPROG: zg30
TD: 32768
Solvent: CDCl3
NS: 16
DS: undefined
SWH: 8223.7 Hz
AQ: undefined
TE: 295.3 C

======== CHANNEL f1 ========
NUC1: 1H
P1: 9.93 usec
SFO1: undefined MHz

F2 - Processing Parameters
SI: 65536
DC: 0.05
LB: 0.30 Hz
First Point: 0.50
FT: Hyper Quadrature
Phase: Manual
Ph0: -262.23
Ph1: 10.12

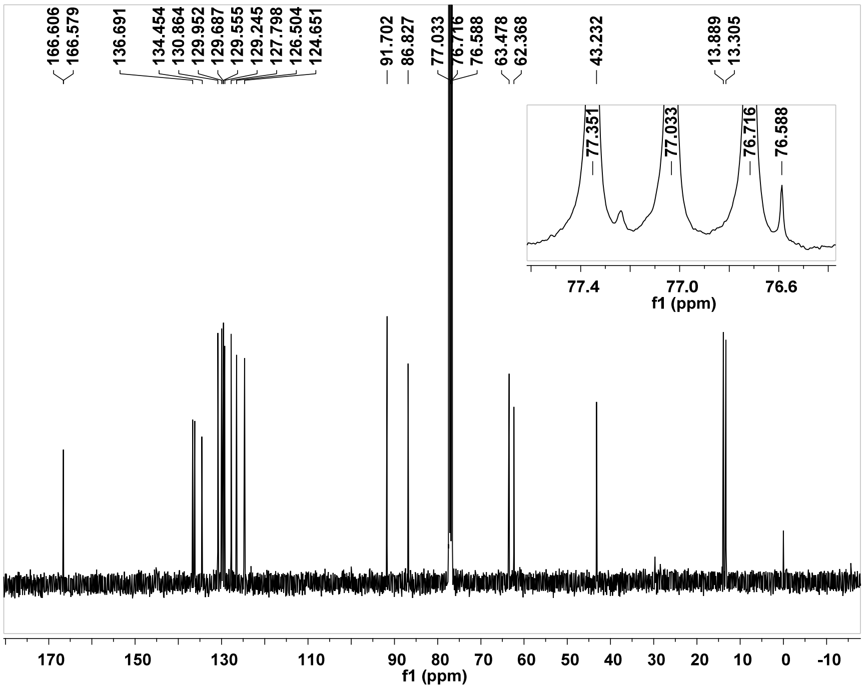

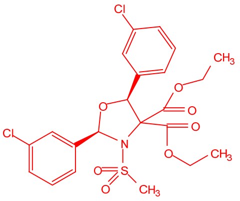

Current Data Parameters

F2 - Acquisition Parameters
DATE: 2015-05-05T20:04:12
PULPROG: zgpg30
TD: 32768
Solvent: CDCl3
NS: 1024
DS: undefined
SWH: 24038.5 Hz
AQ: undefined
TE: 295.6 C

======== CHANNEL f1 ========
NUC1: 13C
P1: 9.63 usec
SFO1: undefined MHz

F2 - Processing Parameters
SI: 65536
DC: 0.05
LB: 1.00 Hz
First Point: 0.50
FT: Hyper Quadrature
Phase: Manual
Ph0: -48.29
Ph1: 46.88

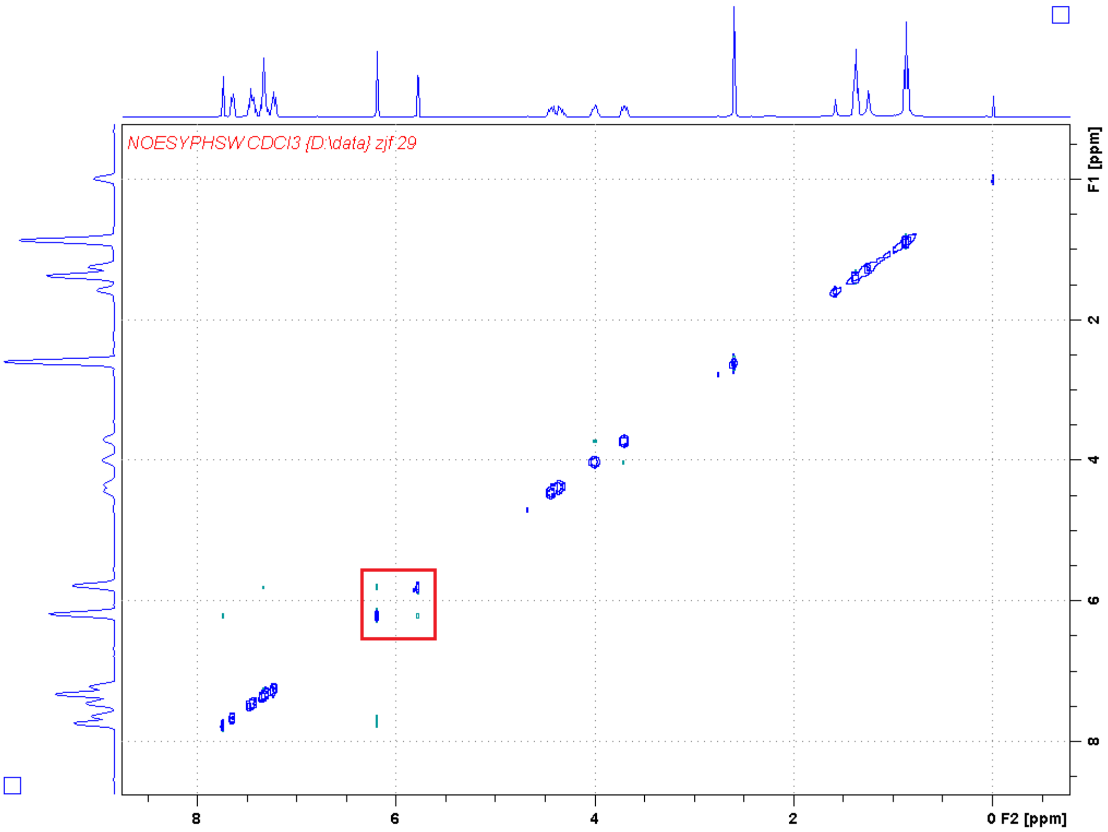
NOESYPHSW CDCl3 {D:\data} zjf 29
F1 [ppm]
F2 [ppm]
8
6
4
2
0

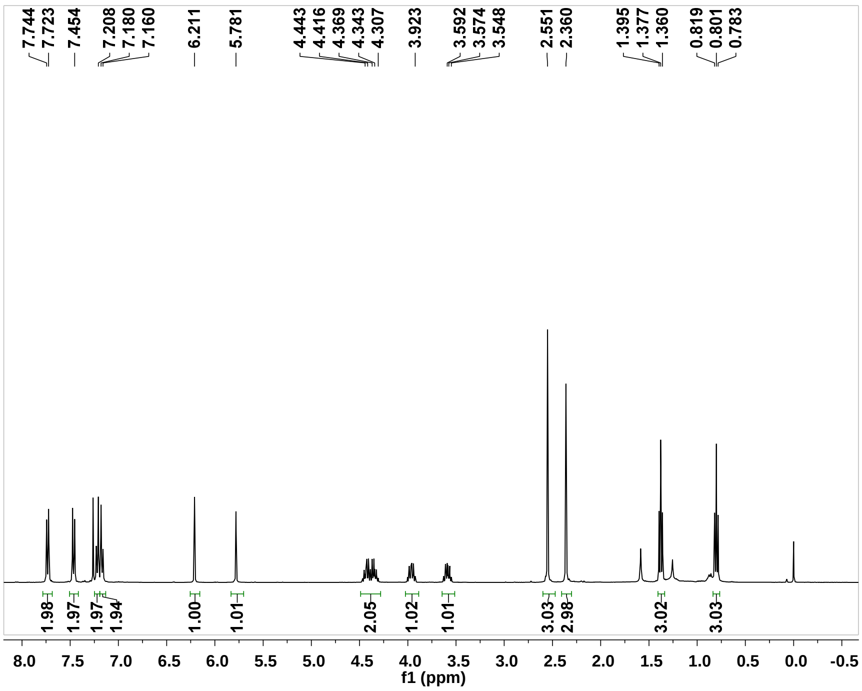

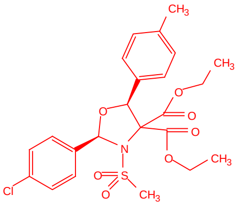

Current Data Parameters

F2 - Acquisition Parameters
DATE: 2015-05-01T10:36:09
PULPROG: zg30
TD: 32768
Solvent: CDCl3
NS: 32
DS: undefined
SWH: 8223.7 Hz
AQ: undefined
TE: 295.3 C

======== CHANNEL f1 ========
NUC1: 1H
P1: 9.93 usec
SFO1: undefined MHz

F2 - Processing Parameters
SI: 65536
DC: 0.05
LB: 0.30 Hz
First Point: 0.50
FT: Hyper Quadrature
Phase: Manual
Ph0: 91.87
Ph1: 17.59

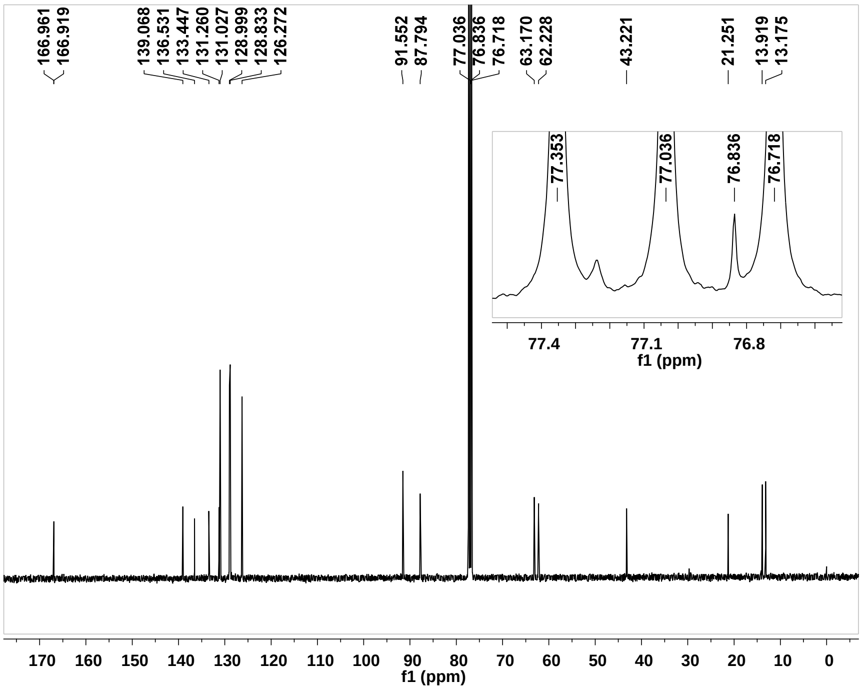

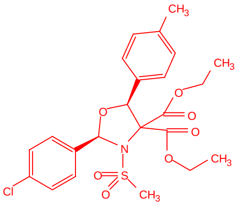

Current Data Parameters

F2 - Acquisition Parameters
DATE: 2015-05-01T20:38:38
PULPROG: zgpg30
TD: 32768
Solvent: CDCl3
NS: 1024
DS: undefined
SWH: 24038.5 Hz
AQ: undefined
TE: 295.6 C

======== CHANNEL f1 ========
NUC1: 13C
P1: 9.63 usec
SFO1: undefined MHz

F2 - Processing Parameters
SI: 65536
DC: 0.05
LB: 1.00 Hz
First Point: 0.50
FT: Hyper Quadrature
Phase: Manual
Ph0: -61.33
Ph1: 53.29

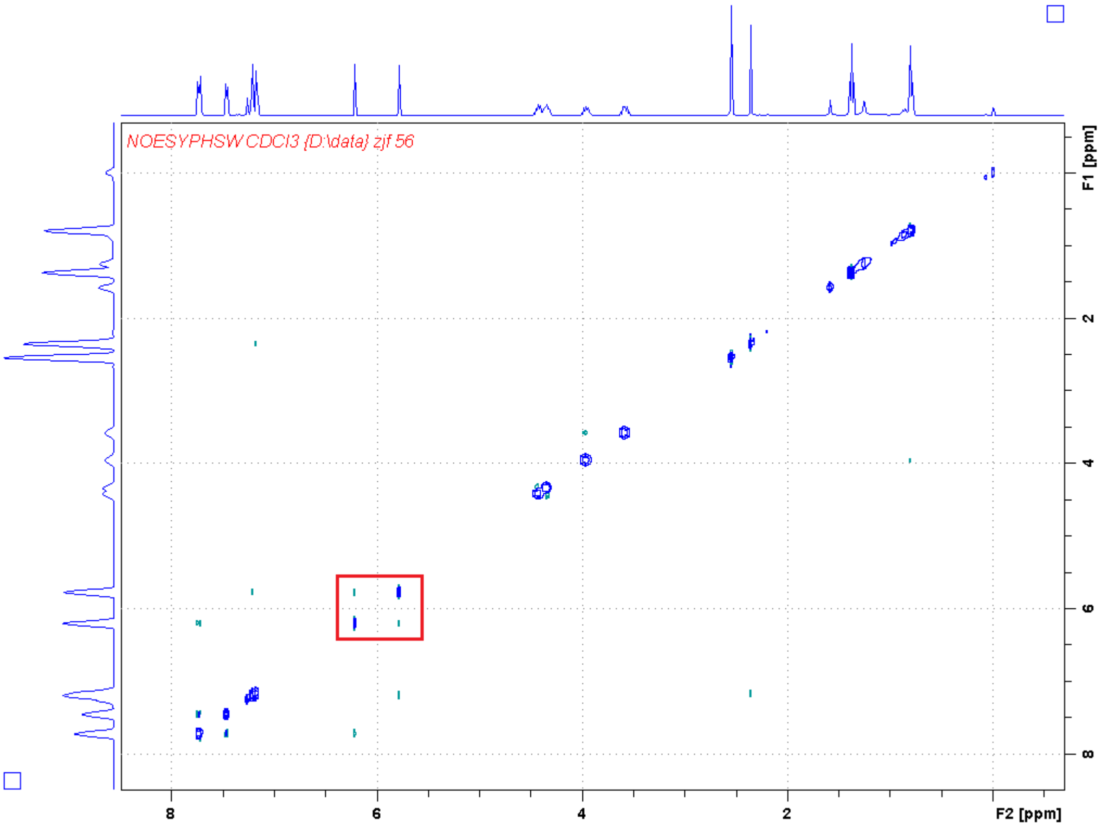
NOESYPHSW CDCl3 {D:\data} zjf 56
F1 [ppm]
F2 [ppm]
8
6
4
2

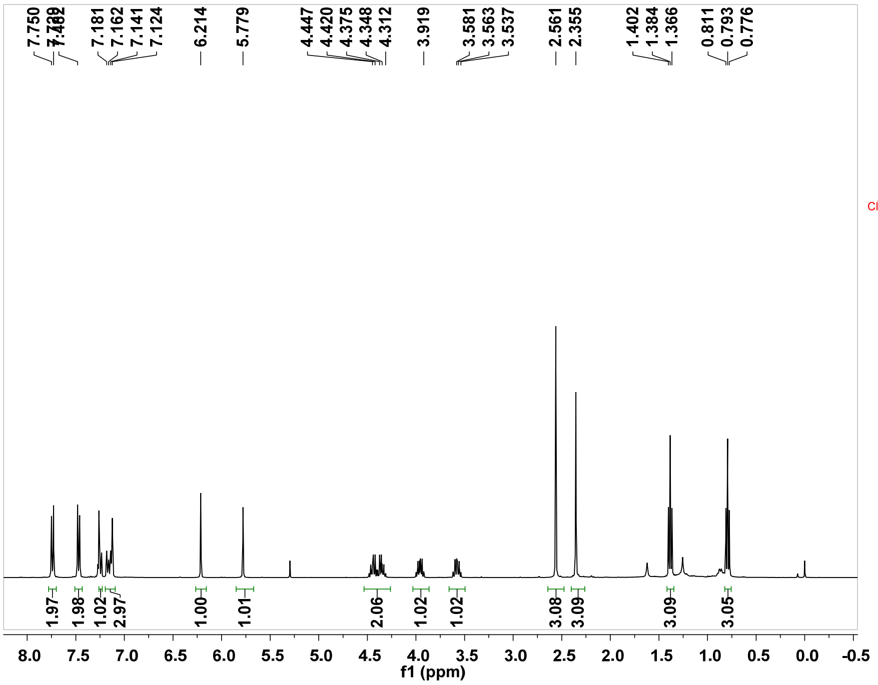

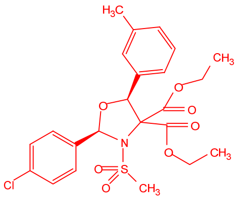

Current Data Parameters

F2 - Acquisition Parameters
DATE: 2015-05-04T14:16:05
PULPROG: zg30
TD: 32768
Solvent: CDCl3
NS: 32
DS: undefined
SWH: 8223.7 Hz
AQ: undefined
TE: 295.1 C

======== CHANNEL f1 ========
NUC1: 1H
P1: 9.93 usec
SFO1: undefined MHz

F2 - Processing Parameters
SI: 65536
DC: 0.05
LB: 0.30 Hz
First Point: 0.50
FT: Hyper Quadrature
Phase: Manual
Ph0: 94.44
Ph1: 18.50

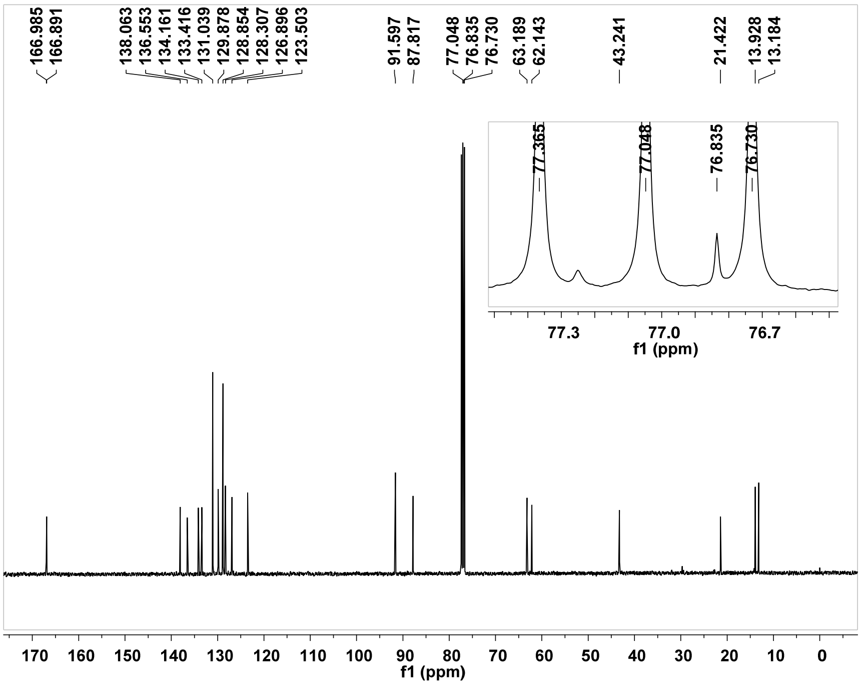

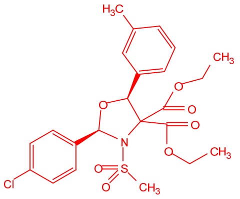

Current Data Parameters

F2 - Acquisition Parameters
DATE: 2015-05-04T15:15:31
PULPROG: zgpg30
TD: 32768
Solvent: CDCl3
NS: 1024
DS: undefined
SWH: 24038.5 Hz
AQ: undefined
TE: 295.4 C

======== CHANNEL f1 ========
NUC1: 13C
P1: 9.63 usec
SFO1: undefined MHz

F2 - Processing Parameters
SI: 65536
DC: 0.05
LB: 1.00 Hz
First Point: 0.50
FT: Hyper Quadrature
Phase: Manual
Ph0: -74.55
Ph1: 78.89

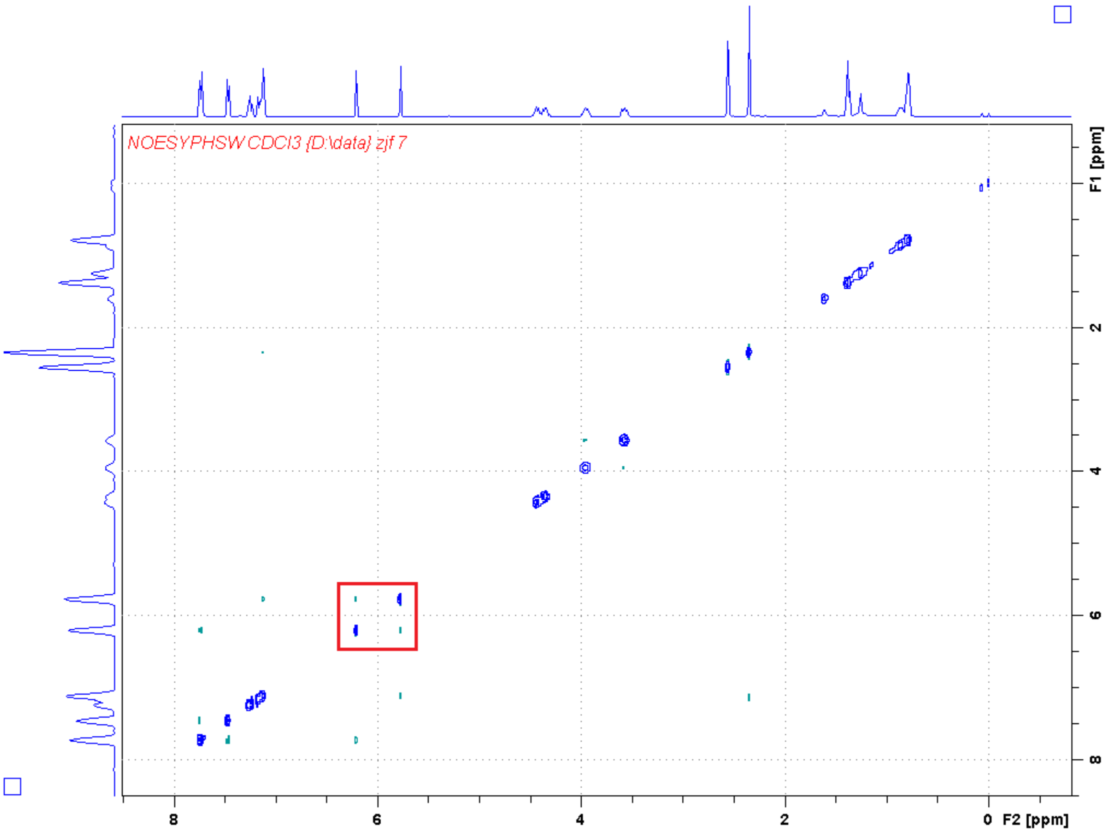
NOESYPHSW CDCl3 {D:\data} zjf 7
F1 [ppm]
F2 [ppm]
8
6
4
2
0

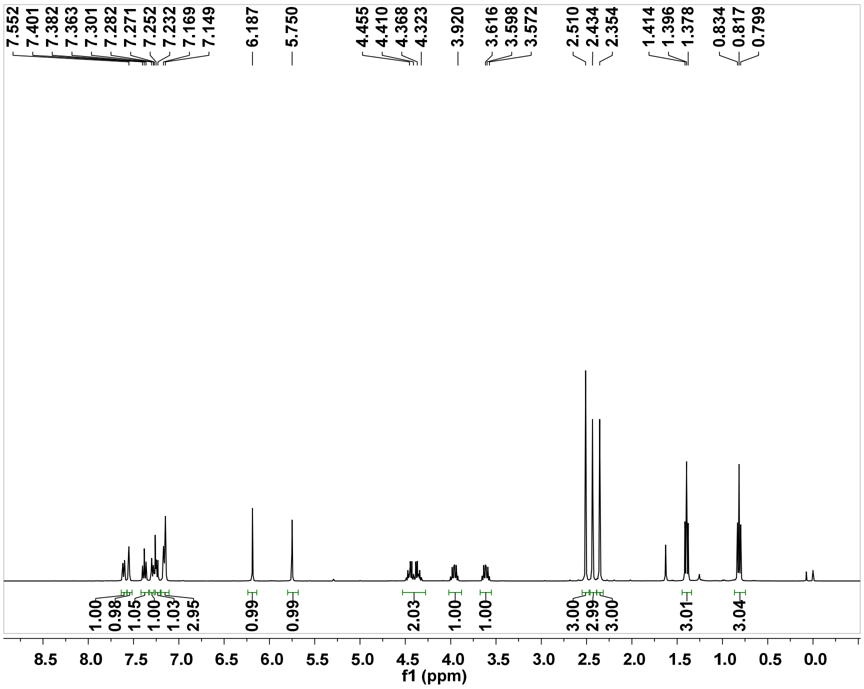

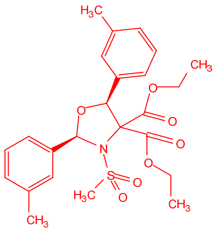

Current Data Parameters

F2 - Acquisition Parameters
DATE: 2015-06-06T19:34:47
PULPROG: zg30
TD: 32768
Solvent: CDCl3
NS: 16
DS: undefined
SWH: 8223.7 Hz
AQ: undefined
TE: 295.4 C

======== CHANNEL f1 ========
NUC1: 1H
P1: 9.93 usec
SFO1: undefined MHz

F2 - Processing Parameters
SI: 65536
DC: 0.05
LB: 0.30 Hz
First Point: 0.50
FT: Hyper Quadrature
Phase: Manual
Ph0: 93.89
Ph1: 19.48

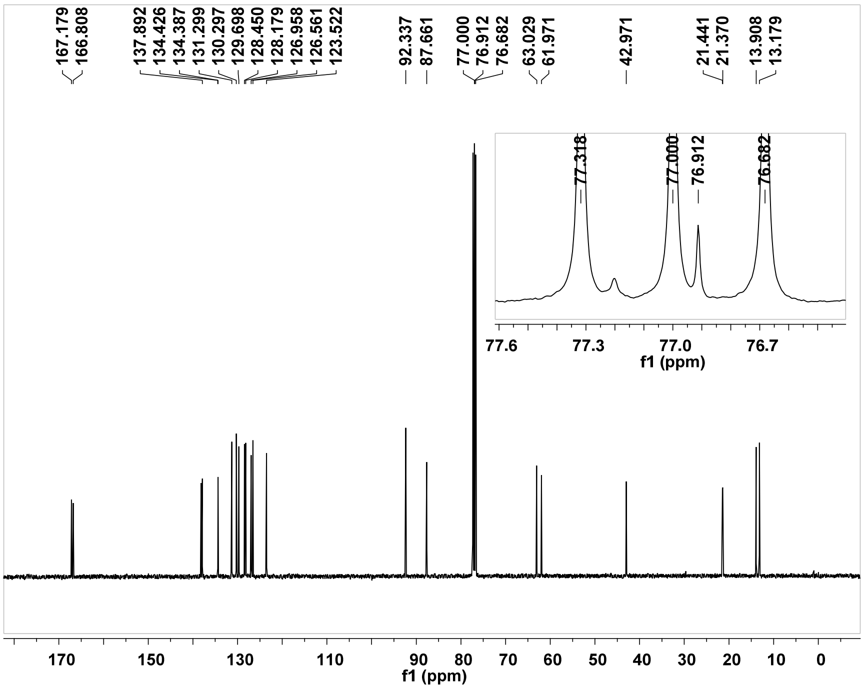

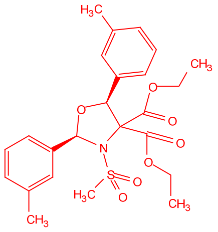

Current Data Parameters

F2 - Acquisition Parameters
DATE: 2015-06-06T21:30:28
PULPROG: zgpg30
TD: 32768
Solvent: CDCl3
NS: 1024
DS: undefined
SWH: 24038.5 Hz
AQ: undefined
TE: 295.7 C

======== CHANNEL f1 ========
NUC1: 13C
P1: 9.63 usec
SFO1: undefined MHz

F2 - Processing Parameters
SI: 65536
DC: 0.05
LB: 1.00 Hz
First Point: 0.50
FT: Hyper Quadrature
Phase: Manual
Ph0: -71.88
Ph1: 64.78

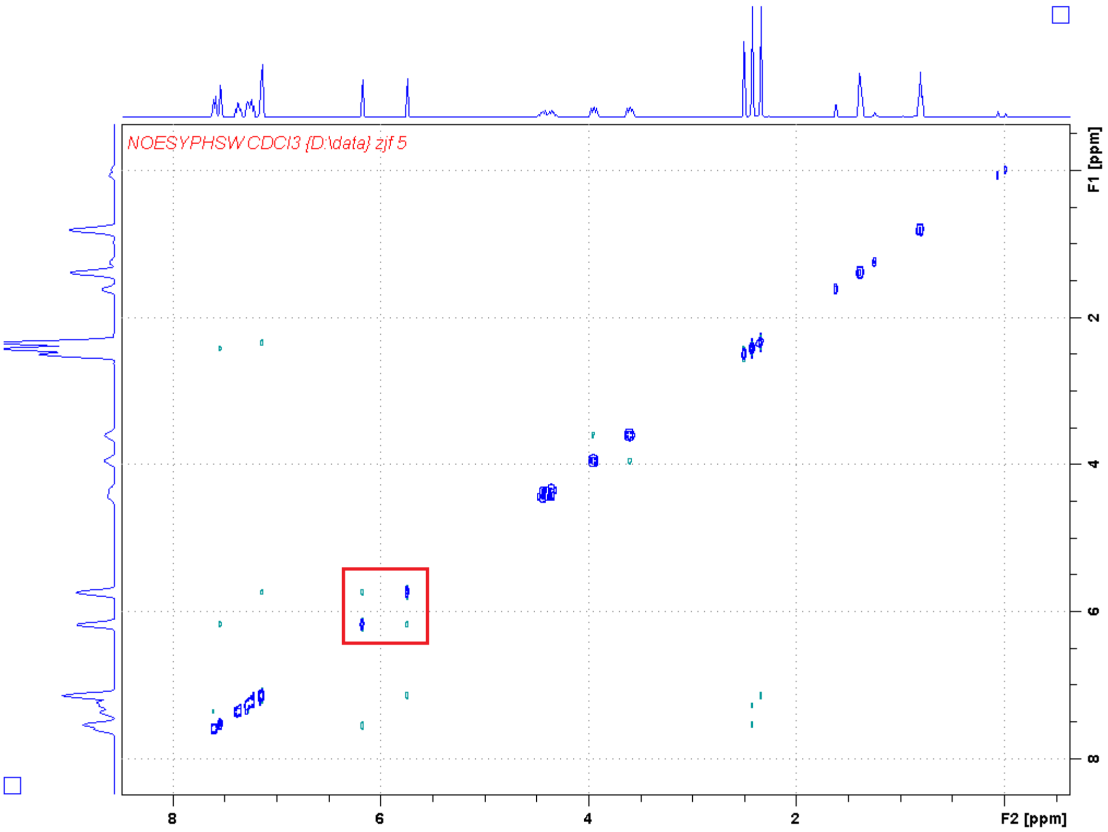

NOESYPHSW CDCl3 {D:\data} zjf 5
F1 [ppm]
F2 [ppm]
8
6
4
2

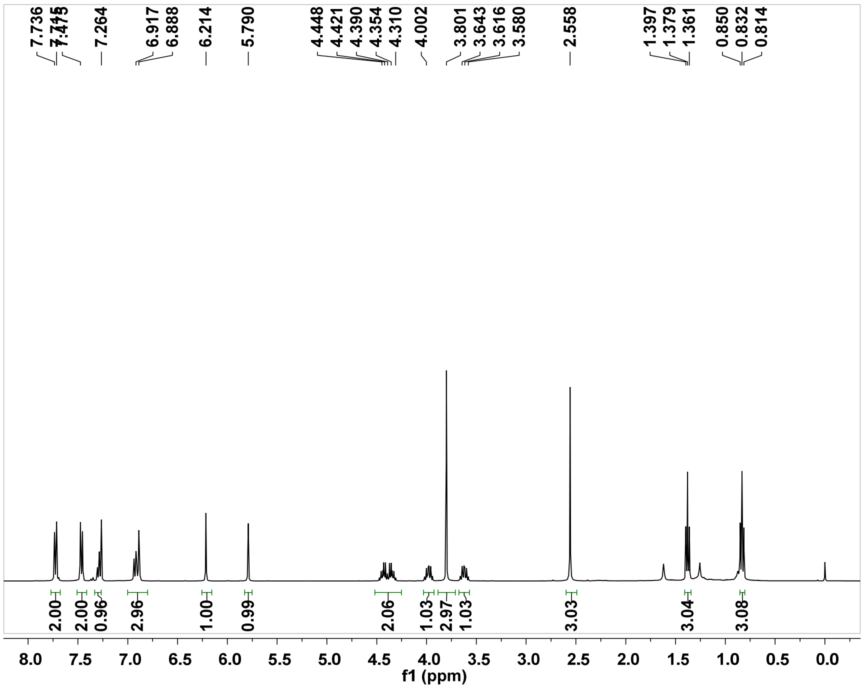

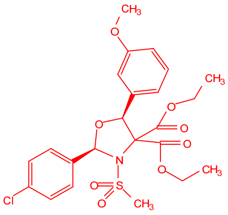

Current Data Parameters

F2 - Acquisition Parameters
DATE: 2015-05-08T02:11:56
PULPROG: zg30
TD: 32768
Solvent: CDCl3
NS: 32
DS: undefined
SWH: 8223.7 Hz
AQ: undefined
TE: 295.5 C

======== CHANNEL f1 ========
NUC1: 1H
P1: 9.93 usec
SFO1: undefined MHz

F2 - Processing Parameters
SI: 65536
DC: 0.05
LB: 0.30 Hz
First Point: 0.50
FT: Hyper Quadrature
Phase: Manual
Ph0: 94.52
Ph1: 14.14

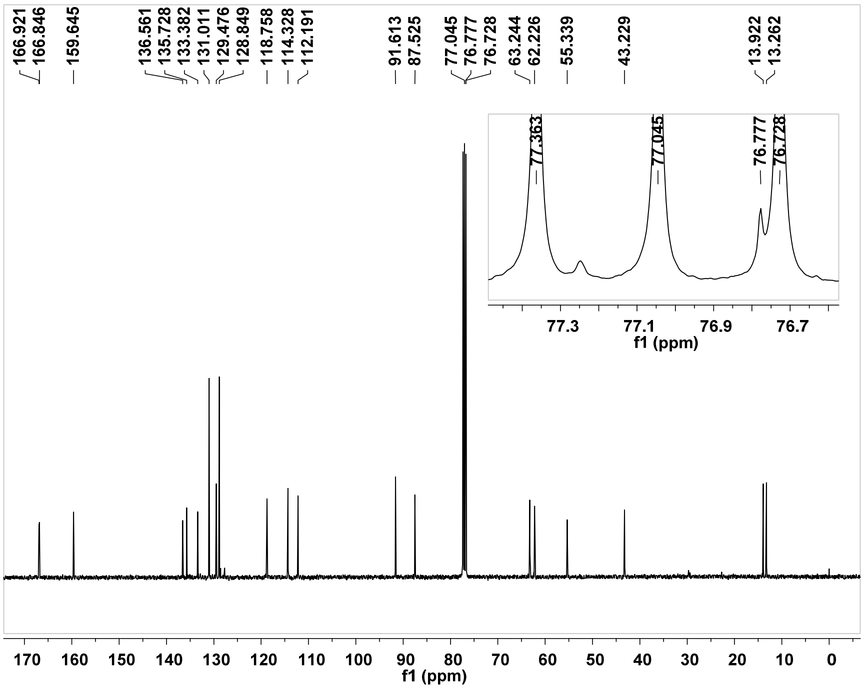

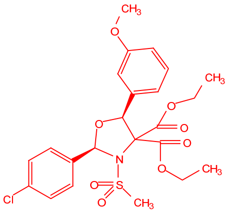

Current Data Parameters

F2 - Acquisition Parameters
DATE: 2015-05-08T04:04:12
PULPROG: zgpg30
TD: 32768
Solvent: CDCl3
NS: 1024
DS: undefined
SWH: 24038.5 Hz
AQ: undefined
TE: 295.9 C

======== CHANNEL f1 ========
NUC1: 13C
P1: 9.63 usec
SFO1: undefined MHz

F2 - Processing Parameters
SI: 65536
DC: 0.05
LB: 1.00 Hz
First Point: 0.50
FT: Hyper Quadrature
Phase: Manual
Ph0: -59.43
Ph1: 59.25

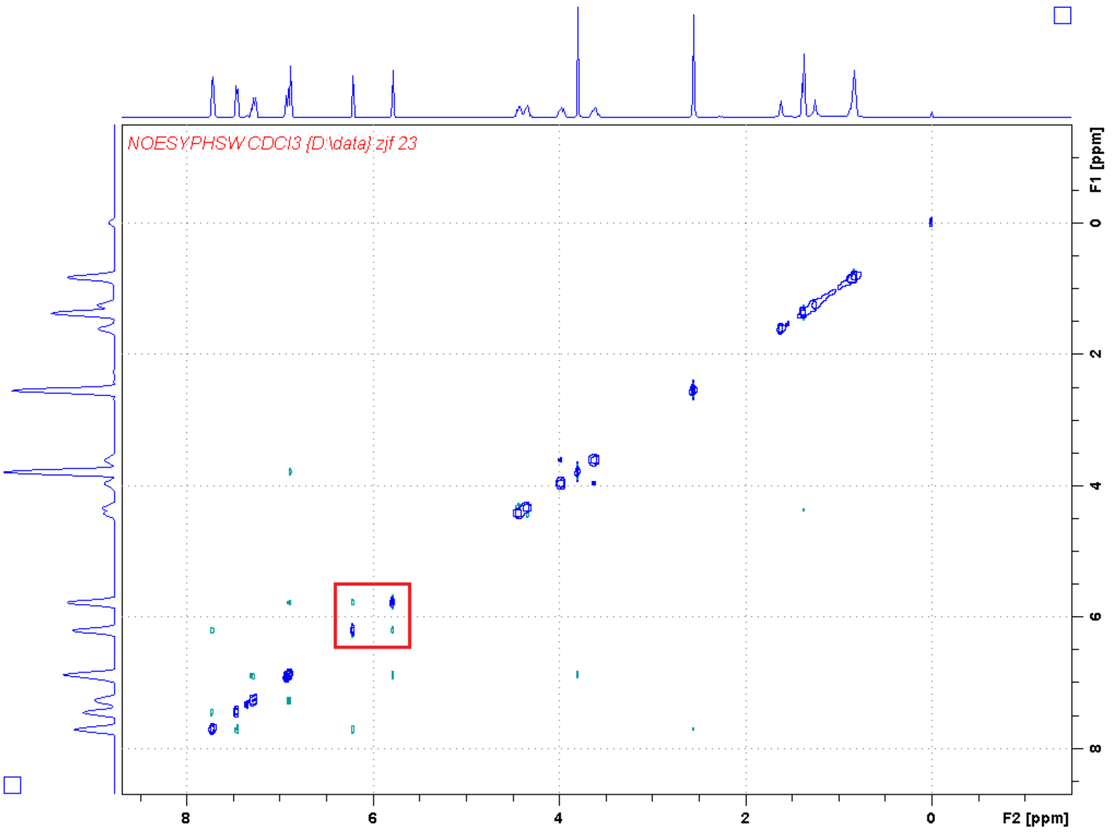

NOESYPHSW CDCl3 {D:\data} zjf 23
F1 [ppm]
F2 [ppm]
8
6
4
2
0

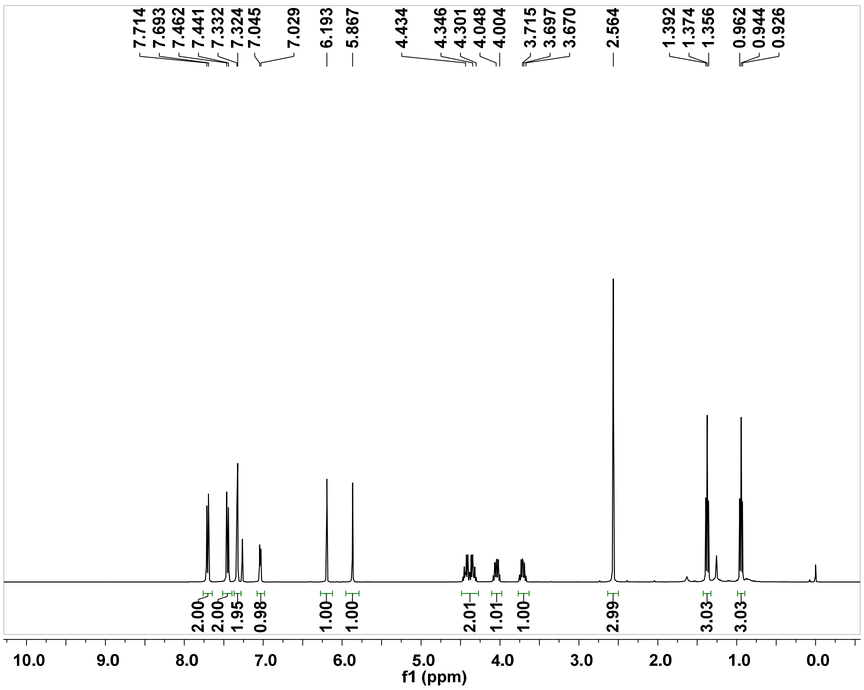

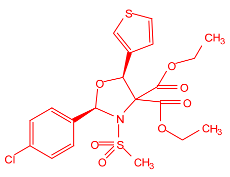

Current Data Parameters

F2 - Acquisition Parameters
DATE: 2015-05-13T12:40:00
PULPROG: zg30
TD: 32768
Solvent: CDCl3
NS: 16
DS: undefined
SWH: 8223.7 Hz
AQ: undefined
TE: 295.6 C

======== CHANNEL f1 ========
NUC1: 1H
P1: 9.93 usec
SFO1: undefined MHz

F2 - Processing Parameters
SI: 65536
DC: 0.05
LB: 0.30 Hz
First Point: 0.50
FT: Hyper Quadrature
Phase: Manual
Ph0: 95.23
Ph1: 17.70

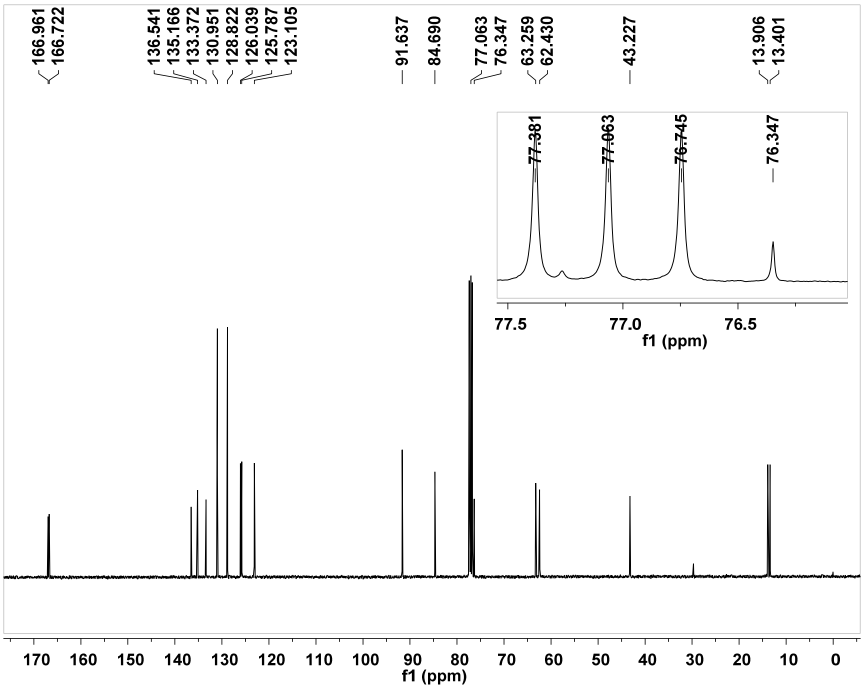

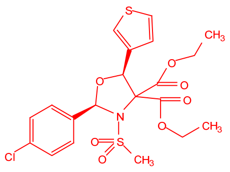

Current Data Parameters

F2 - Acquisition Parameters
DATE: 2015-05-13T14:35:41
PULPROG: zgpg30
TD: 32768
Solvent: CDCl3
NS: 1024
DS: undefined
SWH: 24038.5 Hz
AQ: undefined
TE: 295.8 C

======== CHANNEL f1 ========
NUC1: 13C
P1: 9.63 usec
SFO1: undefined MHz

F2 - Processing Parameters
SI: 65536
DC: 0.05
LB: 1.00 Hz
First Point: 0.50
FT: Hyper Quadrature
Phase: Manual
Ph0: -63.85
Ph1: 64.31

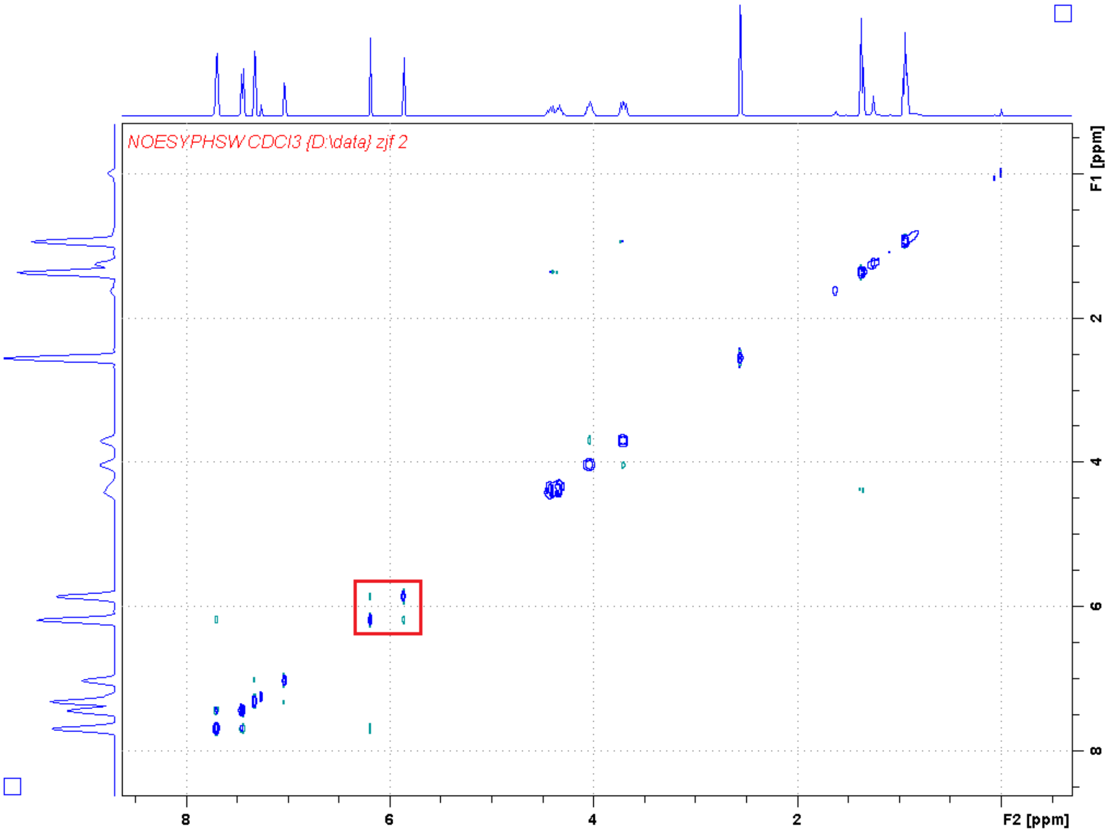

NOESYPHSW CDCl3 {D:\data} zjf 2
F1 [ppm]
F2 [ppm]
8
6
4
2
2
4
6
8

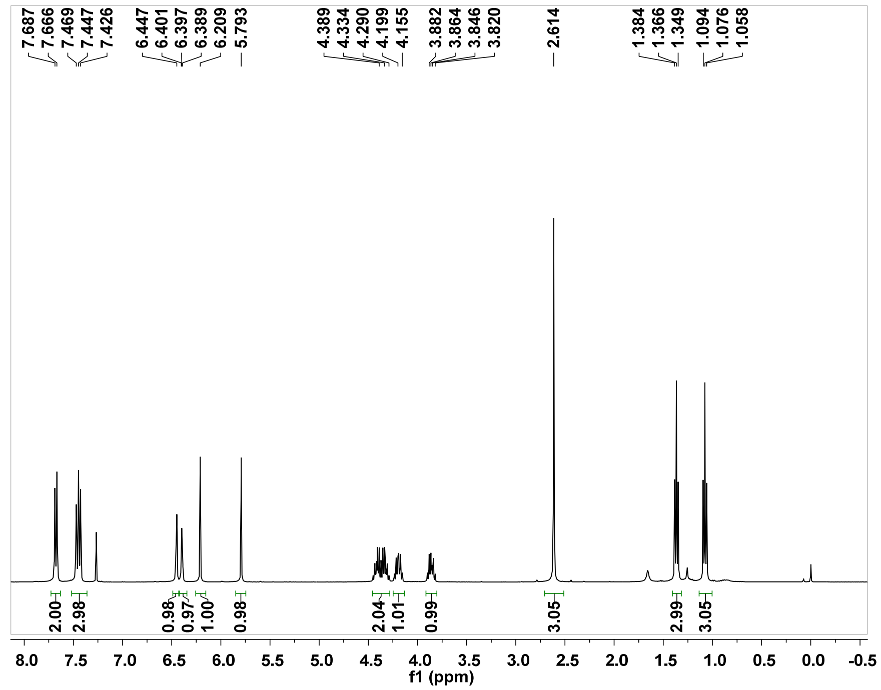

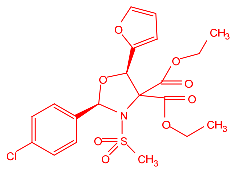

Current Data Parameters

F2 - Acquisition Parameters
DATE: 2015-05-15T15:36:25
PULPROG: zg30
TD: 32768
Solvent: CDCl3
NS: 16
DS: undefined
SWH: 8223.7 Hz
AQ: undefined
TE: 295.7 C

======== CHANNEL f 1 ========
NUC1: 1H
P1: 9.93 usec
SFO1: undefined MHz

F2 - Processing Parameters
SI: 65536
DC: 0.05
LB: 0.30 Hz
First Point: 0.50
FT: Hyper Quadrature
Phase: Manual
Ph0: -264.42
Ph1: 17.22

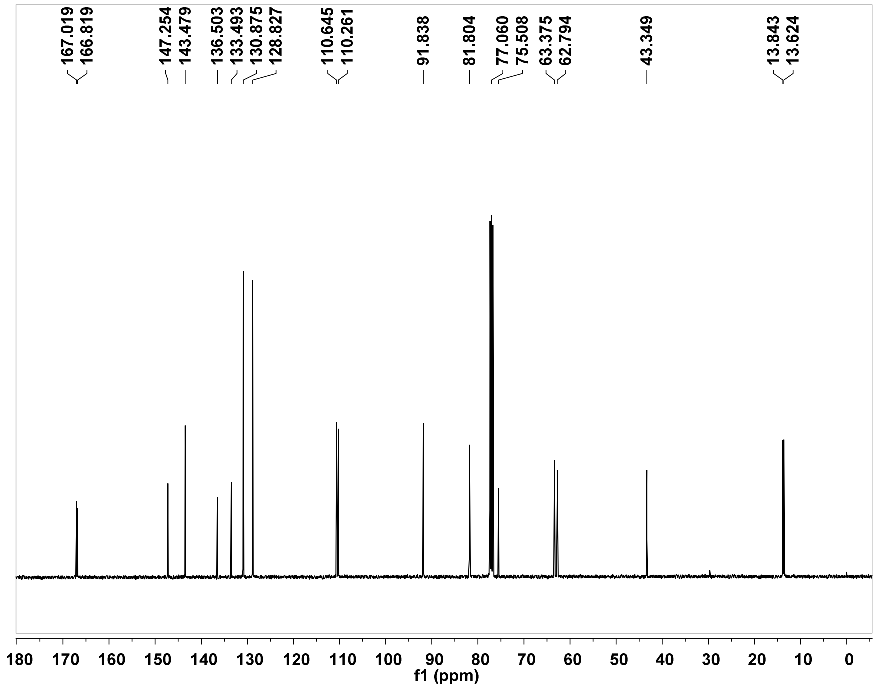

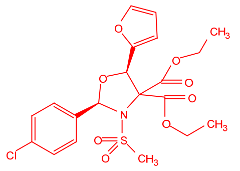

Current Data Parameters

F2 - Acquisition Parameters
DATE: 2015-05-15T17:32:09
PULPROG: zgpg30
TD: 32768
Solvent: CDCl3
NS: 1024
DS: undefined
SWH: 24038.5 Hz
AQ: undefined
TE: 296.4 C

======== CHANNEL f 1 ========
NUC1: 13C
P1: 9.63 usec
SFO1: undefined MHz

F2 - Processing Parameters
SI: 65536
DC: 0.05
LB: 1.00 Hz
First Point: 0.50
FT: Hyper Quadrature
Phase: Manual
Ph0: -66.30
Ph1: 59.71

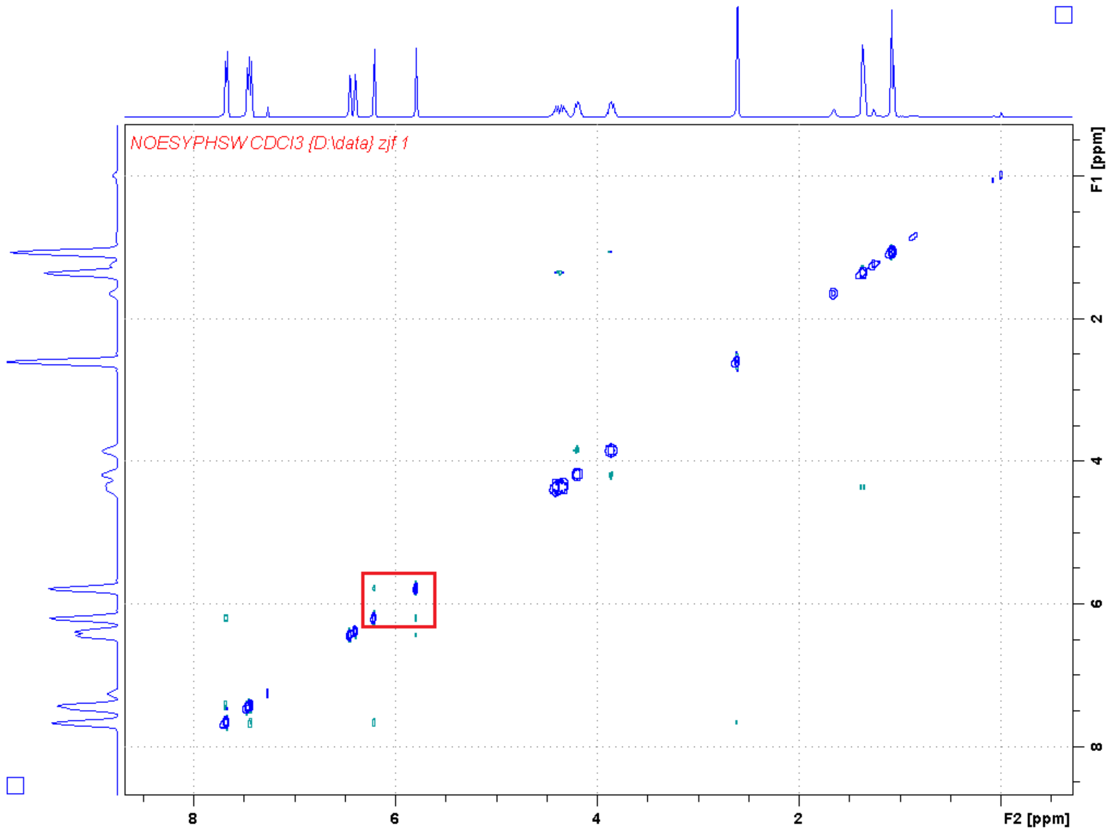

NOESYPHSW CDCl3 {D:\data} zjf 1
F1 [ppm]
F2 [ppm]

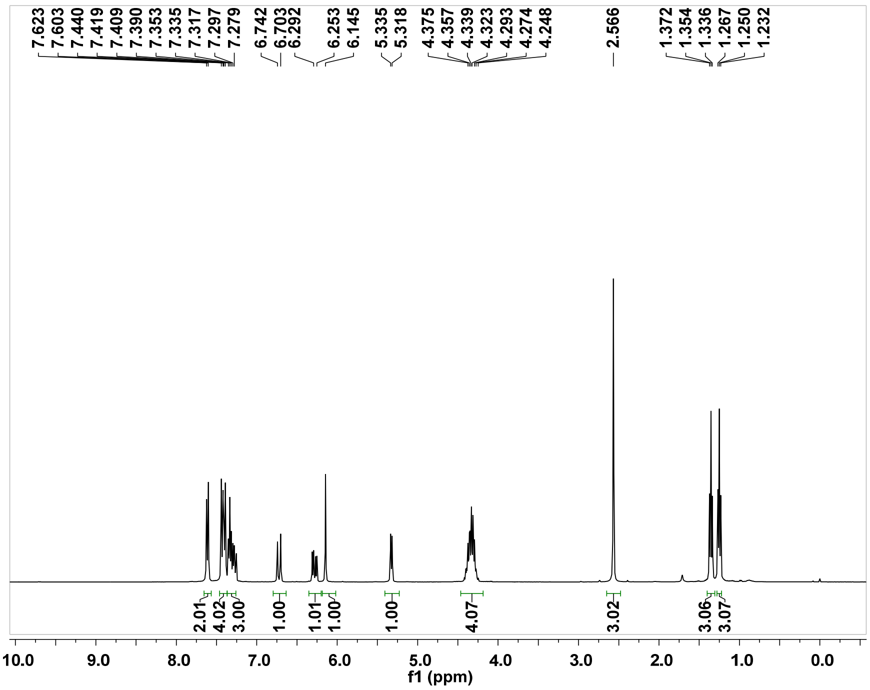

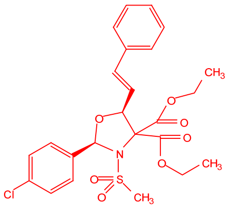

Current Data Parameters

F2 - Acquisition Parameters
DATE: 2015-05-09T16:46:55
PULPROG: zg30
TD: 32768
Solvent: CDCl3
NS: 16
DS: undefined
SWH: 8223.7 Hz
AQ: undefined
TE: 295.9 C

======== CHANNEL f1 ========
NUC1: 1H
P1: 9.93 usec
SFO1: undefined MHz

F2 - Processing Parameters
SI: 65536
DC: 0.05
LB: 0.30 Hz
First Point: 0.50
FT: Hyper Quadrature
Phase: Manual
Ph0: 94.52
Ph1: 18.71

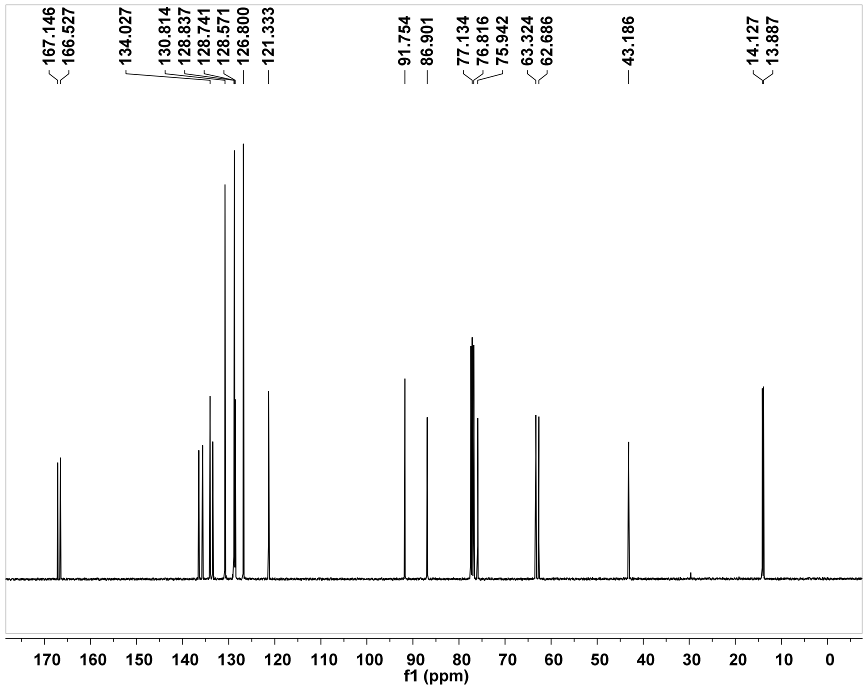

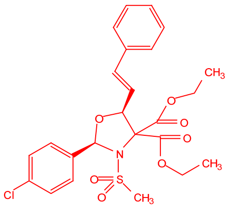

Current Data Parameters

F2 - Acquisition Parameters
DATE: 2015-05-09T18:42:31
PULPROG: zgpg30
TD: 32768
Solvent: CDCl3
NS: 1024
DS: undefined
SWH: 24038.5 Hz
AQ: undefined
TE: 296.3 C

======== CHANNEL f1 ========
NUC1: 13C
P1: 9.63 usec
SFO1: undefined MHz

F2 - Processing Parameters
SI: 65536
DC: 0.05
LB: 1.00 Hz
First Point: 0.50
FT: Hyper Quadrature
Phase: Manual
Ph0: -60.68
Ph1: 62.33

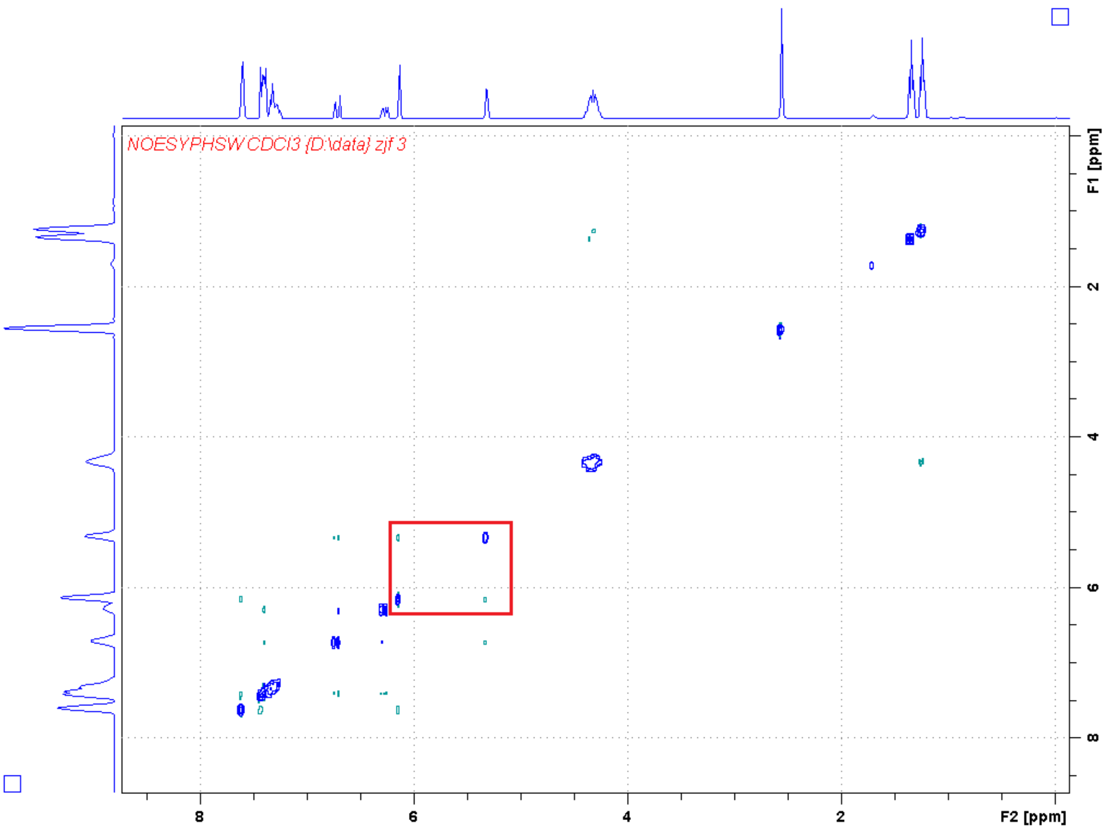
NOESYPHSW CDCl3 {D:\data} zjf 3
F1 [ppm]
F2 [ppm]
8
6
4
2
2
4
6
8

(O) Copies of CD Spectra

a. CD Spectra for the cycloadducts in CH_2Cl_2, (*2R*, *5S*)-**3sa** is an authentic sample.

(*2R*, *5S*)-**3sa**:

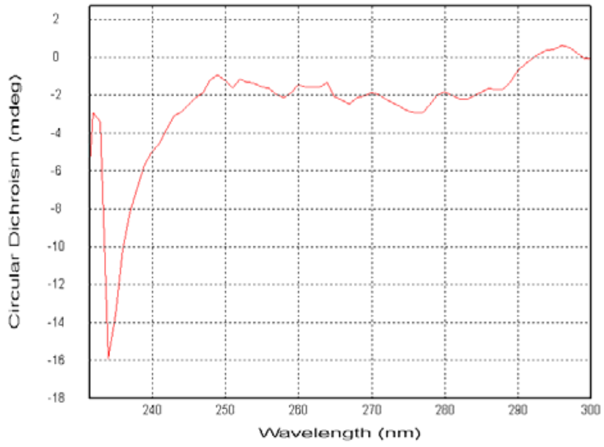

(*2R*, *5S*)-**3ba**:

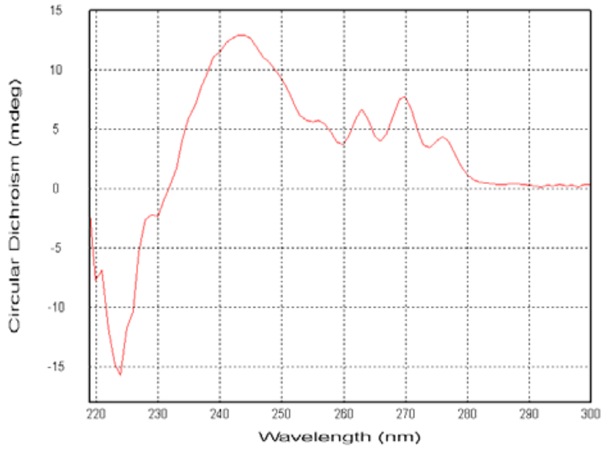

(*2R*, *5S*)-**3ca**:

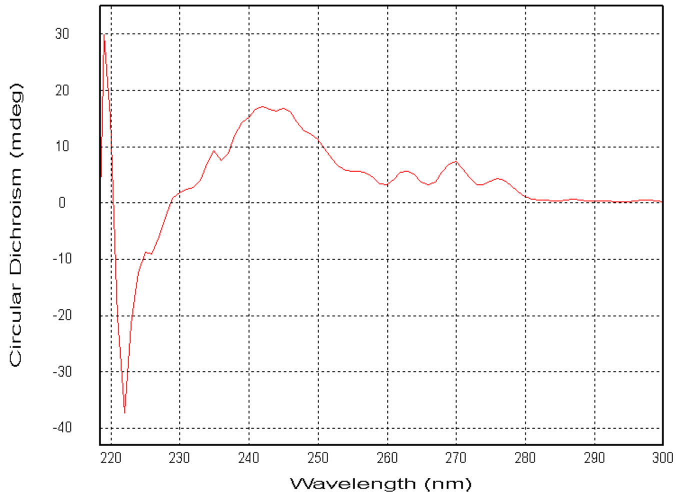

(*2R*, *5S*)-**3da**:

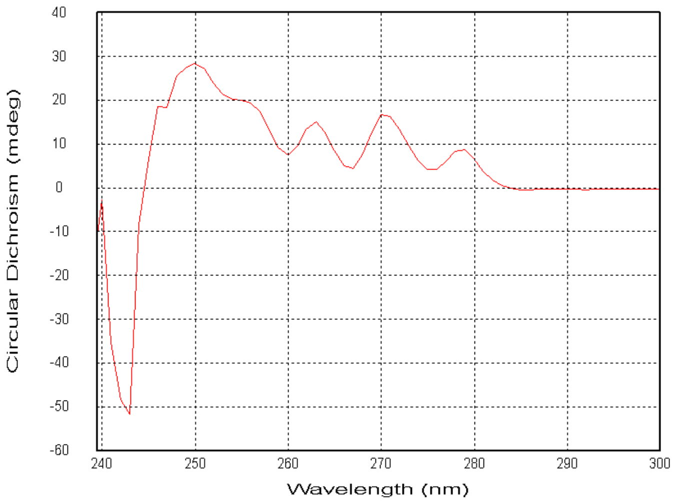

(*2R*, *5S*)-**3ia**:

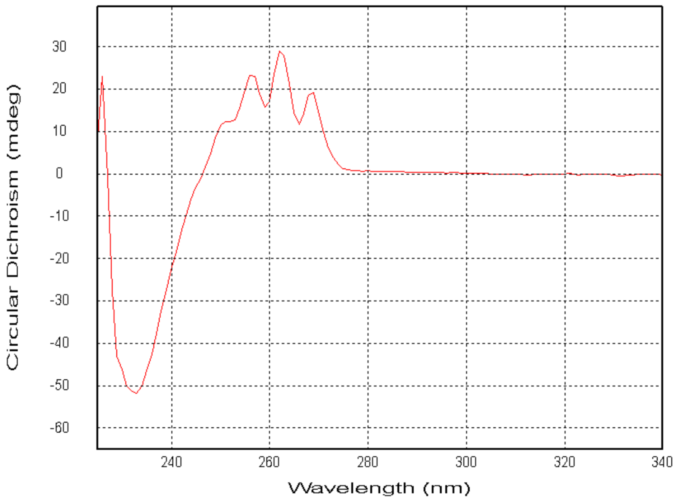

(*2R*, *5S*)-**3ja**:

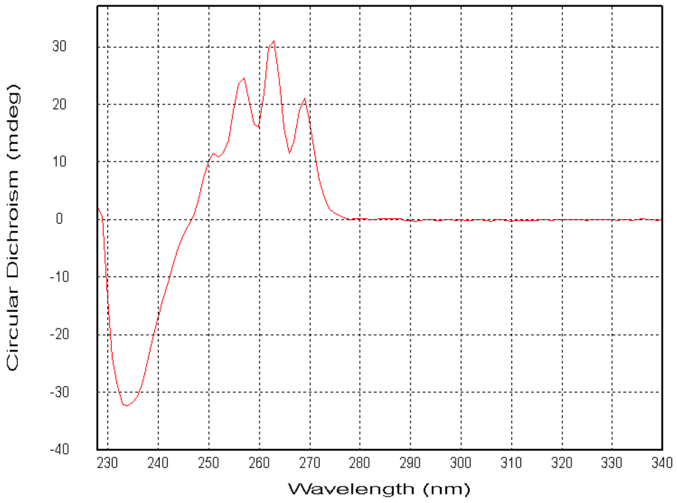

(*2R*, *5S*)-**3ka**:

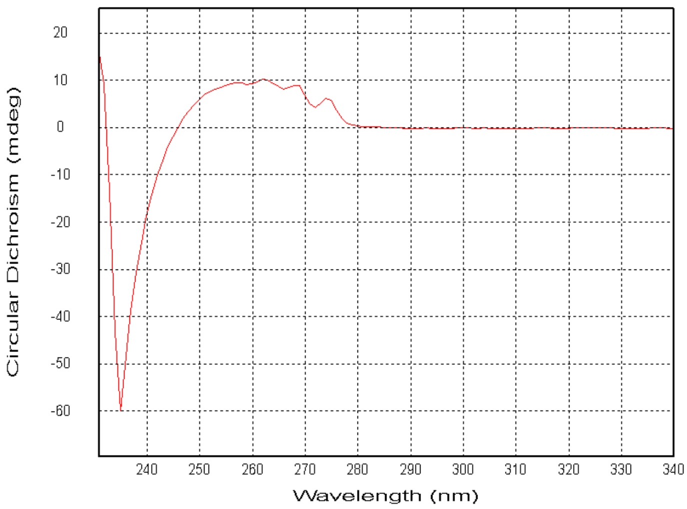

(*2R*, *5S*)-**3la**:

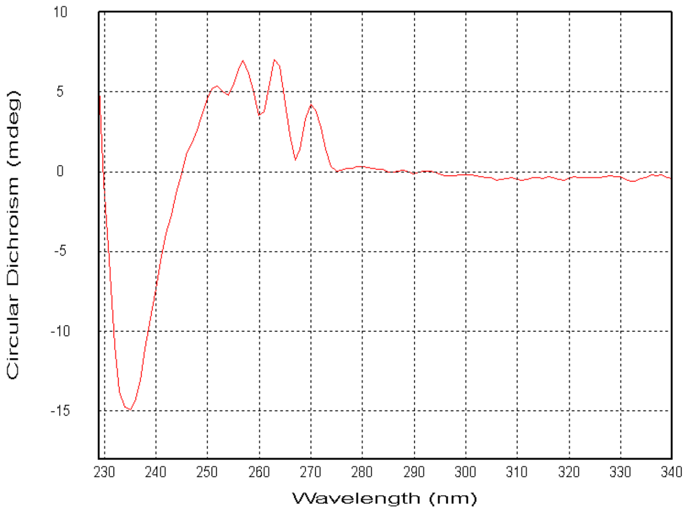

(*2R*, *5S*)-**3ma**:

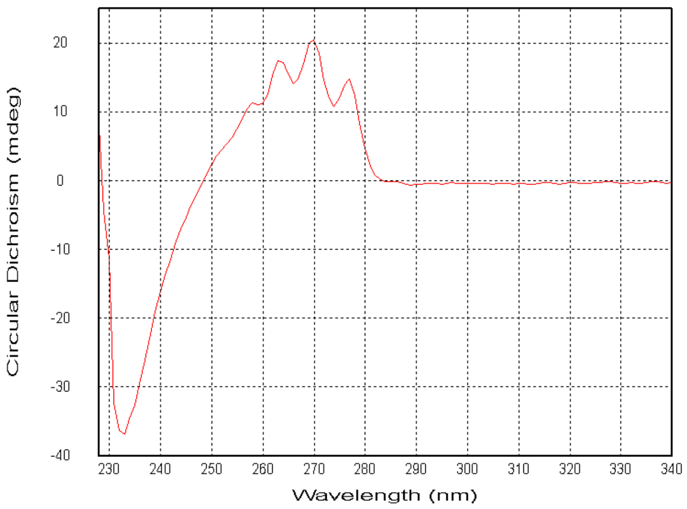

(*2R*, *5S*)-**3na**:

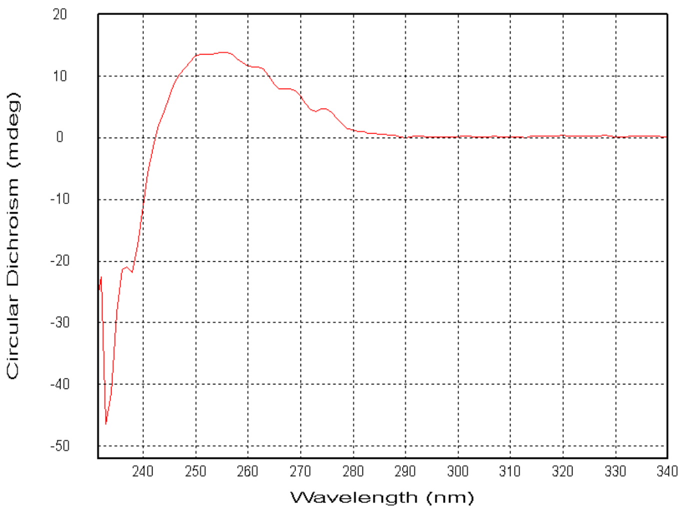

(*2R*, *5S*)-**3oa**:

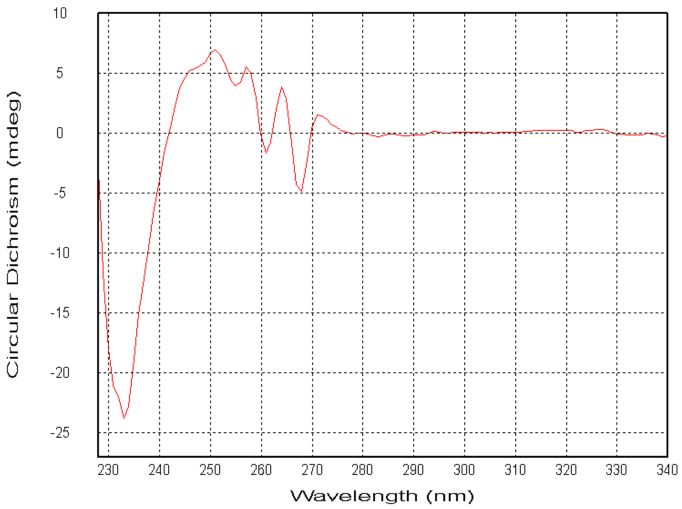

(*2R*, *5S*)-**3pa**:

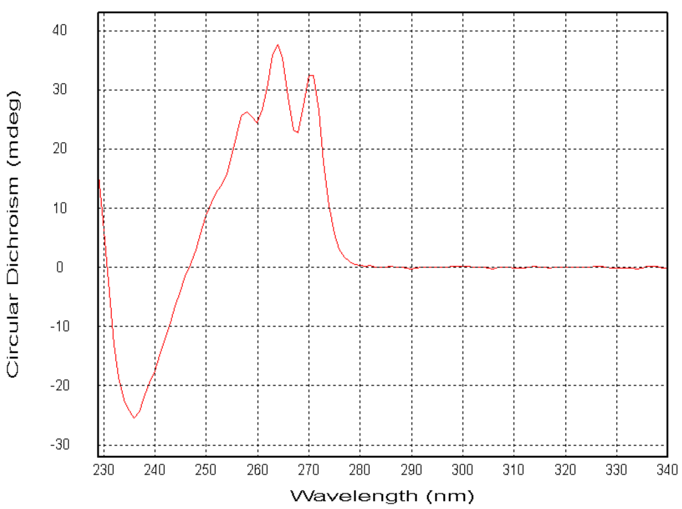

(*2R*, *5S*)-**3kd**:

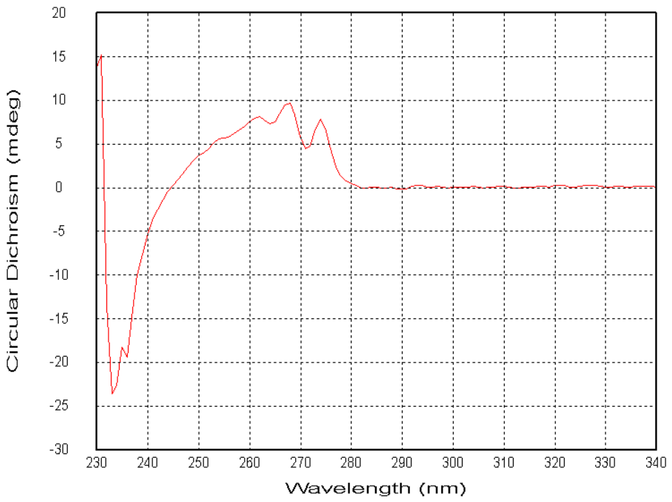

(*2R*, *5S*)-**3ke**:

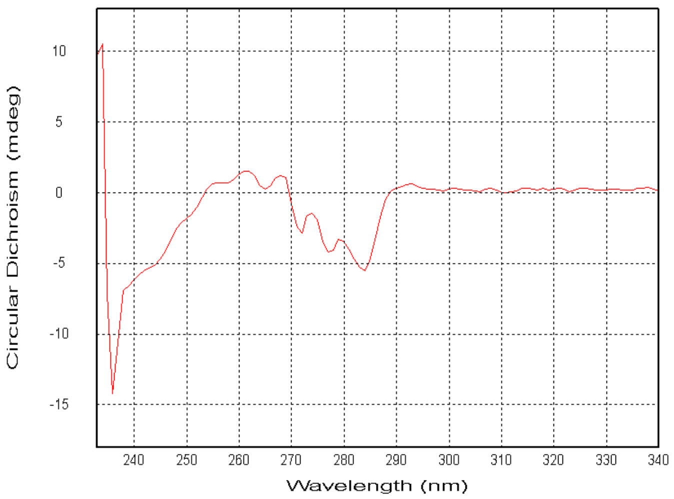

(*2R*, *5S*)-**3te**:

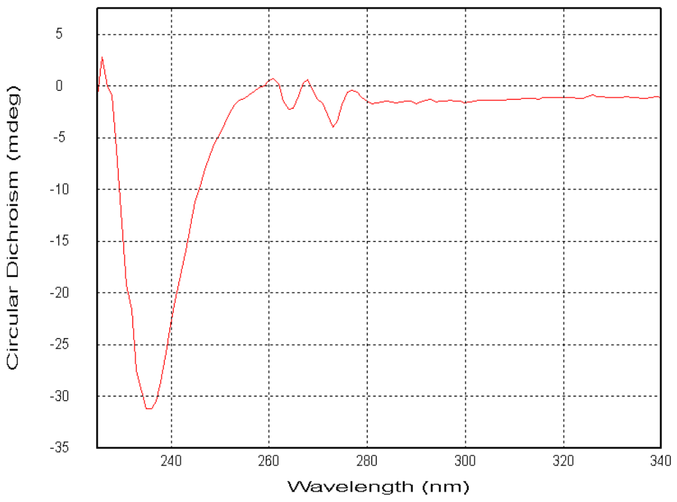

(*2R*, *5S*)-**3kf**:

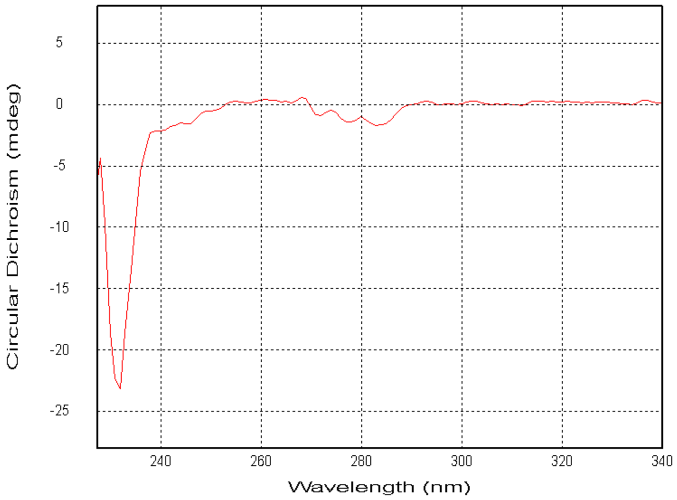

(*2R*, *5S*)-**3kg**:

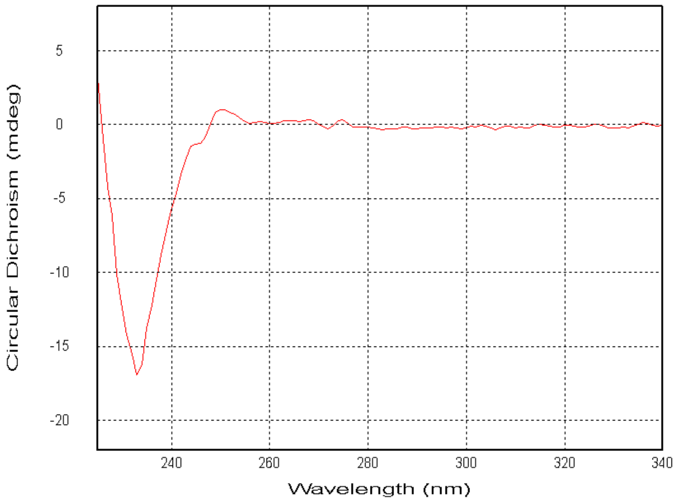

(*2R*, *5R*)-**3kh**:

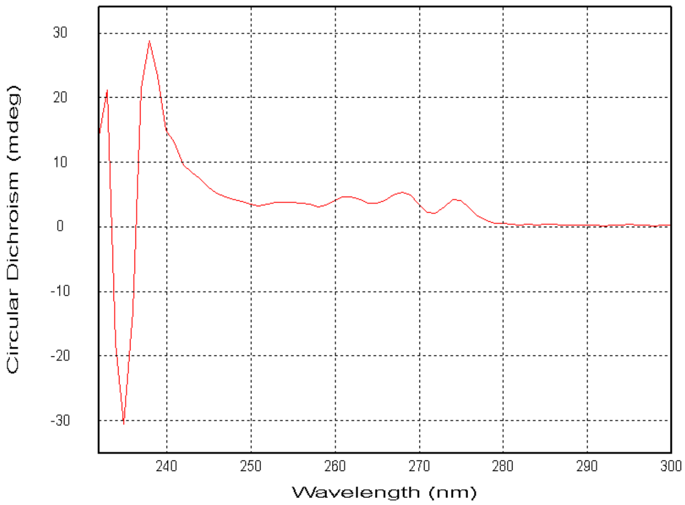

(*2R*, *5S*)-**3ki**:

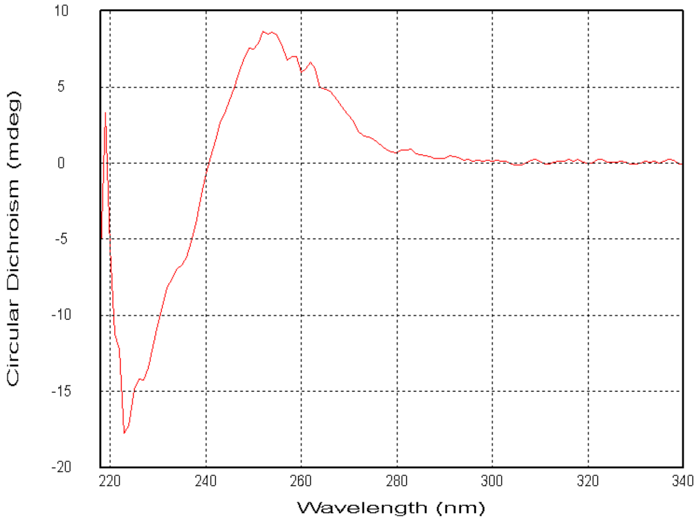

10
5
0
-5
-10
-15
-20
Circular Dichroism (mdeg)
220
240
260
280
300
320
340
Wavelength (nm)
